# Molecular recognition of organic ammonium ions in solution using synthetic receptors

**DOI:** 10.3762/bjoc.6.32

**Published:** 2010-04-06

**Authors:** Andreas Späth, Burkhard König

**Affiliations:** 1 Institut für Organische Chemie, Universität Regensburg, D-93040 Regensburg, Germany, Phone: +49-943-941-4576, Fax: +49-943-941-1717

**Keywords:** amino acids, ammonium ion, molecular recognition, synthetic receptors

## Abstract

Ammonium ions are ubiquitous in chemistry and molecular biology. Considerable efforts have been undertaken to develop synthetic receptors for their selective molecular recognition. The type of host compounds for organic ammonium ion binding span a wide range from crown ethers to calixarenes to metal complexes. Typical intermolecular interactions are hydrogen bonds, electrostatic and cation–π interactions, hydrophobic interactions or reversible covalent bond formation. In this review we discuss the different classes of synthetic receptors for organic ammonium ion recognition and illustrate the scope and limitations of each class with selected examples from the recent literature. The molecular recognition of ammonium ions in amino acids is included and the enantioselective binding of chiral ammonium ions by synthetic receptors is also covered. In our conclusion we compare the strengths and weaknesses of the different types of ammonium ion receptors which may help to select the best approach for specific applications.

## Introduction

**Figure 1:**
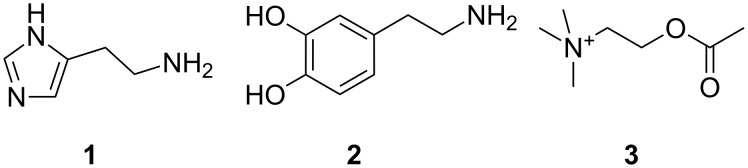
Biologically important amines and quaternary ammonium salts: histamine (**1**), dopamine (**2**) and acetylcholine (**3**).

The amino group is one of the most important functional groups in molecules of biological relevance. Examples of physiologically active amines (Figure 1) are histamine (**1**), dopamine (**2**) and quaternary ammonium ions, such as acetylcholine (**3**). Amino acids have amino groups like peptides and proteins. Under physiological conditions the amino group is usually protonated as an ammonium ion.

The interaction of small ammonium ion bearing compounds with protein receptors is important for biological signal transduction processes. As in all biological regulatory processes, selectivity of recognition is of key importance for subsequent steps and cellular response. An example is the binding of histamine (**1**) to the human H_1_ receptor, which results in lower blood pressure and dilatation of blood vessels or plays a primary role for allergic response [1–4]. The inhibition of biological processes is also addressed by molecular recognition involving amino acids and peptides: The antibiotic vancomycin binds selectively with its terminal lysyl-*R*-alanyl-*R*-alanine residues in bacterial cells through several hydrogen bonds [5]. Once it has bound to these particular peptides, they are no longer available for construction of the bacteria’s cell wall causing their cell death.

Malfunction of dopamine-responsive neurons has been implicated in a number of disease conditions including Parkinson’s disease [6]. The understanding of alkylammonium recognition in the dopamine (**2**) class of neurotransmitters is key to the development of tools to study these systems. Therefore the investigation of ammonium ion recognition is of considerable fundamental and practical interest [7,120].

Selective ligand-protein receptor binding relies typically on a number of specific interactions between two or more molecules. For the recognition of ammonium ions, three types of interactions, mostly acting simultaneously, are typically the most important:

1) Hydrogen bonds [8]

Hydrogen bonds are formed from the strongly polarized N^+^–H bonds to a free electron pair of an electronegative atom (O, N, F). Crystal structures mainly show a linear arrangement of the three atoms but bifurcated hydrogen bonds can also be observed [9]. If exposed to a competing solvent, a single hydrogen bond cannot contribute much binding energy. Gas phase energies range from 22 kJ/mol (neutral hydrogen bonds between water molecules) up to 163 kJ/mol (anionic F–H–F^−^ complex) [10]. Quaternary ammonium ions cannot be bound by hydrogen bonds.

2) Cation–π-interaction [11]

The first experimental evidence of interactions between cations and aromatic π-systems came from Kebarle et al. who showed that binding of potassium ions to benzene and water in the gas phase is of similar energy [9,12]. Ammonium–π-interactions were experimentally investigated in detail as well as by ab initio calculations and are mainly based on electrostatic interactions. The binding energies are between 42 and 92 kJ/mol in the gas phase. The cation–π-bond is an important motif for the recognition of quaternary ammonium ions. A relevant example is the binding of acetylcholine (**3**) in biological systems [13].

3) Ion pairs and salt bridges

Coulombic interaction attracts cations and anions. In salt bridges, additional hydrogen bonds are formed [14]. A typical example of a salt bridge is the ammonium ion carboxylate ion pair. The strength of cation–anion affinity depends on the distance, the polarity of the solvent and the ionic strength. When extrapolated to zero ionic strength, most coulombic interactions are around 8 kJ/mol [15]. In aqueous medium ion pair formation is primarily driven by entropy, not directly by coulombic forces [16]. The binding energy is, in general, independent of the geometry, polarizability of the ions or the formation of a salt bridge.

In addition, the selective recognition of ammonium ions depends on steric and molecular complementarity and the pre-organization [17] of interacting functional groups. As far back as 1890, Fischer suggested that enzyme–substrate interactions function like a “lock and key” between an initially empty host and a guest that exhibit molecular complementarity [18].

Today studies of non-covalent interactions, mainly by artificial model structures and receptors, have led to a far better understanding of many biological processes. Moreover, they are often the inspiration for supramolecular research, including self-assembly, mechanically-interlocked molecular architectures and molecular recognition in host–guest chemistry [19]. Analogous to biological systems, the formation and function of such supramolecular complexes occurs through a multiplicity of often difficult to differentiate non-covalent forces: Di- or polytopic receptors are used to enhance further the binding and selectivity with a binding mechanism that can be understood on the combined efforts of several non-covalent interactions such as hydrogen bonding, electrostatic interactions, hydrophobic interactions [20–22], cation–π interactions, π–π staking interactions [23–24] and steric complementarity [25]. The crucial interaction mechanisms have been comprehensively summarized [26–27]; basic rules for receptors and design have been outlined [28–29].

As in nature, molecular recognition can either be static – a complexation reaction with defined stoichiometry between a specific host and guest – or dynamic, where the binding of the first guest to the first binding site of a receptor affects the association constant of a second guest with a second binding site. Either positive allosteric binding – the first guest increases the association constant of the second guest – or negative allosteric binding – the first guest decreases the association constant with the second – can occur [30]. Positive allostery or co-operativity [31–32] is desireable for synthetic receptors. In most cases the host forms a cavity in which guest molecules are complexed as the “key” in the complementary binding site or an inclusion compound. This host pre-organization leads to a major enhancement of the overall energy of guest complexation. The binding is energetically favored: Both enthalpic – a less solvent accessible area leads to a less strongly solvated guest with fewer solvent-ligand bonds to break – as well as entropic – macrocycles [33] or cavities [34] being less conformationally flexible so losing fewer degrees of freedom upon complexation as a result of the reorganization energy already paid in advance in the synthesis.

In a few examples, guest molecules are enclosed on all sides by the receptor being “trapped” as in a cage forming clathrates [35]. Binding of the amino group to a planar surface of the receptor is found in metal complexes or metalla-porphyrins. The molecular environment and the solvent determine the stability of the assembly: competitive solvents building strong hydrogen bonds or having electrostatic and charge-transfer capabilities interfere with the ammonium ion binding and may even completely inhibit the complex formation. Recognition in water is especially a challenging topic of growing interest and has been recently reviewed [36].

Many types of synthetic ammonium ion receptors are available, ranging from crown ethers, calixarenes, porphyrins, cucurbiturils, cyclodextrins and cyclopeptides to tweezer ligands, sterically geared tripods and several types of metal complexes. The most important methods used for evaluating ammonium ion binding processes are direct absorption and emission measurements utilizing chromophores in the receptor or analyte molecule, displacement assays with suitable dyes, NMR titration experiments, isothermal titration calorimetry and transport through an organic phase monitored by HPLC, NMR [37–38] or UV–vis absorption [39].

## Review

### 1. Scope and limitations of this survey

Synthetic receptors for organic ammonium ions may help to understand better the individual contributions of the different forces involved in ammonium ion binding. In addition, they are valuable tools as chemosensors for the analytical detection of drugs or biogenic amines, most of which have chiral structures. Enantiomeric recognition is an essential process in living organisms and frequently involve ammonium ion compounds, especially in enzyme–substrate interactions [40], as well as in artificial systems, e.g., in separation science [41–44] and in the design of enzyme mimetics [45–49].

In this review, we discuss the different structures of ammonium ion receptors using typical examples from the recent literature. Where available, examples of enantioselective recognition of chiral ammonium ion guests will be covered. The recognition of guanidinium ions and metal cations [50] is not included. Ion pair recognition will be discussed briefly if it is relevant for ammonium ion recognition purposes. A comprehensive review on this topic has been published by Sessler et al. [51–53]. We also discuss the substance classes that have been mostly used in organic ammonium ion recognition: crown ethers, calixarenes [54], cyclodextrins [55–57], cucurbiturils, porphyrins, phosphonate based receptors, tripodal receptors, tweezer ligands, clefts, cyclopeptides and metal complexes. We have not included rotaxanes [58–64], catenanes [58,65–68], spherands [69], cryptophanes [70–72] as well as switching devices [73–75], self assembly systems [76–84] or carcerands [85–87] because these structures are less frequently used for organic ammonium ion binding, or their binding is based on similar interactions as in the previously noted receptor classes. Comprehensive information on the recognition properties of the compounds is available in the cited literature. We will start every chapter with a short discussion of fundamental properties such as selectivity and complementarity. Beginning with structurally simple examples we will increase complexity to higher substituted moieties and combinations of recognition sites to ditopic or oligomeric receptor types of the class. Synthetic receptors bearing binding sites from different compound classes are classified by their amine recognition moiety.

We present selected results covering complexation, solvent extraction and transport of organic ammonium ions in solution, thus excluding polymer [88–92] or other solid phase [93–95] materials and gas phase measurements, without attempting to cover all available references. Representative molecules for application in ion selective electrodes (ISE) [96] are briefly discussed. Unfortunately, the scope of the review cannot cover the topic of artificial receptors for organic ammonium ions comprehensively. It is rather the intention to illustrate the scope and the limitations of a binding motif with typical examples.

### 2. Crown ethers

This chapter discusses recent reports on ammonium ion recognition using crown ethers and their derivatives. Firstly, the properties of the substance class is illustrated by simple examples followed by more complex crown ethers and related systems. The next part discusses molecules capable to differentiate enantiomeric ammonium ions, followed by receptors for diammonium ions, such as ditopic crown ether compounds. Finally, we discuss the simultaneous recognition of ammonium ions and a second functional group as, for example, in amino acids.

#### 2.1. Ammonium ion binding by simple crown ethers

**Figure 2:**
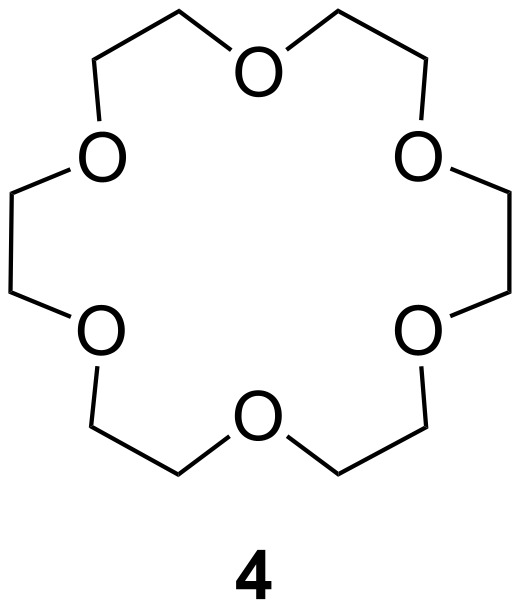
Crown ether 18-crown-6.

In his first publication, Petersen [97], who discovered the compound class and later received the Nobel Prize for it, mentioned the use of crown ethers for the recognition of ammonium ions [98]. Later, after extensive studies on *tert*-butyl ammonium thiosulfate and different crown ethers, Cram [99] and co-workers concluded that two factors are important to achieve high binding constants [100]: The principle of complementary binding sites must be fulfilled. Receptor and guest binding sites should be in close proximity – complementary geometry and fit without generating steric strain. Secondly, receptors which are suitably pre-organized for guest binding will lead to the more stable complex. Crown ether ammonium ion binding occurs by hydrogen bonding between oxygen atoms (or nitrogen, sulfur or other free electron pair in hetero crown ethers) and N^+^–H bonds [101]. The cyclic arrangement leads to a pre-organization of the host [102], whereby selectivity is determined by the ring size. Primary ammonium ions are complexed with highest affinity by 18-crown-6 derivatives [9] (Figure 2).

Table 1 summarizes exemplarily the affinity of benzyl ammonium chloride and 18-crown-6 in several solvents for comparison with other examples found in this review. The data given were determined by isothermal titration calorimetry [9].

**Table 1:** Binding constants of 18-crown-6 and benzyl ammonium chloride in several solvents.

Solvent	log *K*

water	1.44
methanol	4.22; 4.43^a^
isopropanol	4.14
*n*-octanol	3.25
dimethylformamide	2.50
dimethylsulfoxide	1.34

^a^Determined by ion-selective electrode.

**Figure 3:**
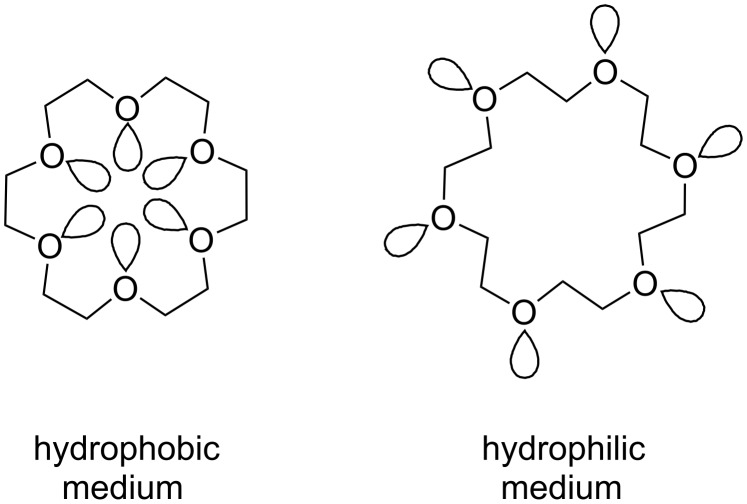
Conformations of 18-crown-6 (**4**) in solvents of different polarity.

These data show that crown ethers bind ammonium ions in different solvents which compete for hydrogen bonds such as dimethylsulfoxide, a very good hydrogen bond acceptor, and water, which is a poorer hydrogen bond acceptor than methanol, but very good hydrogen bond donor. Solvents competing in the intermolecular bond formation result in lower binding constants. Additionally, the binding ability is strongly affected by the polarity of the solvent [103]. The conformation of crown ethers in non-polar organic solvents reflects a “droplet of water in oil” with the lone pairs pointing to its interior in advantageous manner for ion co-ordination (Figure 3). In water, or generally speaking hydrophilic media, the lone pairs are oriented to the exterior. Upon guest co-ordination the crown ether has to be reorganized, which is energetically less favorable. Therefore, highest affinities for polar solvents are observed in methanol; in chloroform the values are even higher [104].

Table 2 shows the effect of the crown ethers size and constitution on the binding constant in methanol. The data were determined using an ion-selective electrode.

**Table 2:** Binding constants of three crown ethers to benzylammonium chloride in methanol.

Crown ether	Cavity size	Guest	log *K*

12-crown-4	120–150 pm	BnNH_3_Cl	0.80
15-crown-5	170–220 pm	BnNH_3_Cl	2.74
18-crown-6	260–320 pm	BnNH_3_Cl	4.43

**Figure 4:**
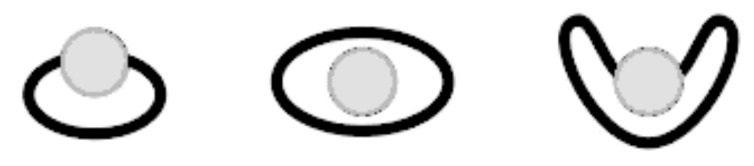
Binding topologies of the ammonium ion depending on the crown ring size.

Depending on the ratio of the crown ether ring size [103] and the diameter of the cation complex, different 1:1 topologies are observed reflecting differently strong co-ordination and complex stability (Figure 4) [105–106].

The ionic diameter of an ammonium ion is 286 pm, very similar to potassium ions with 266 pm. Important is, that ammonium ions prefer a tetrahedral and potassium ions need an octahedral co-ordination for strong binding. By reducing the co-ordination points (see **7b**) [107] or changing the co-ordination sphere, the selectivity of a coronand system can be directed towards ammonium ion binding.

**Figure 5:**
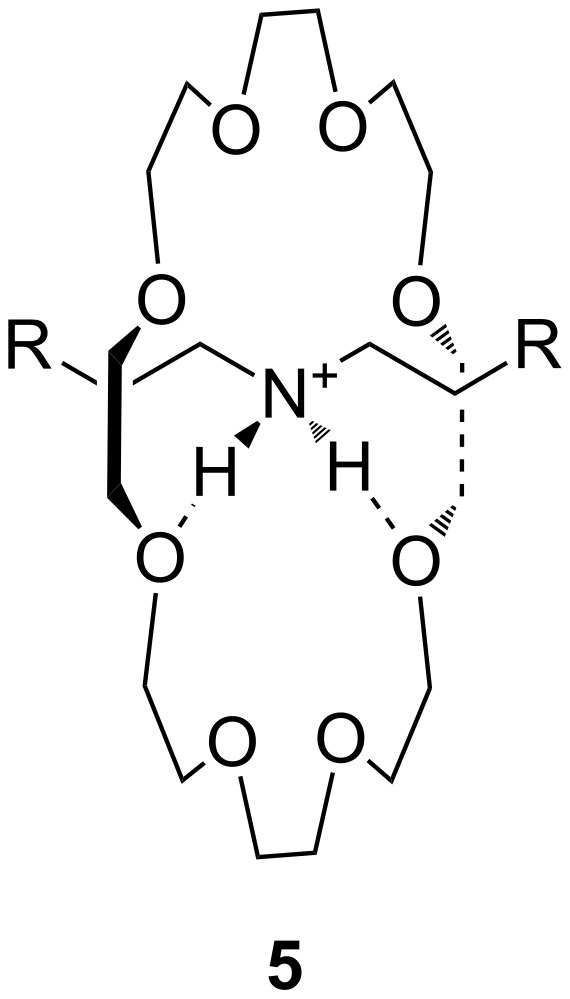
A “pseudorotaxane” structure consisting of 24-crown-8 and a secondary ammonium ion (**5**); R = Ph.

18-Crown-6 type structures typically show the highest affinity for primary ammonium ions, while secondary ammonium ions prefer larger crown ethers [108]. The secondary ammonium ion slips through the crown ether ring forming “pseudorotaxane” like structures (Figure 5).

The structural variability of crown ethers is very large. This allows varying the ring size, introducing substituents and changing the donor sites from oxygen atoms, to nitrogen (azacrowns) or sulfur, or phosphorus or arsenic atoms. Crown ether oxygen atoms as the donor site prefer harder cations of main group elements as guests, while crown ethers with sulphur atoms at the donor site are particularly suitable for the complexation of softer transition metals, e.g. Ag^+^, Cu^2+^, Hg^2+^ [109].

**Figure 6:**
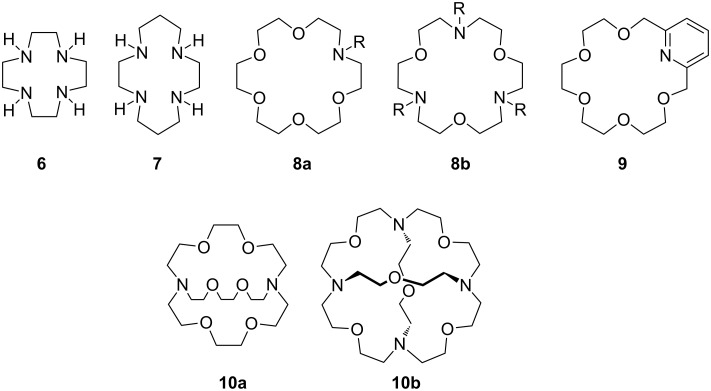
Typical examples of azacrown ethers, cryptands and related aza macrocycles.

Important heterocrowns (Figure 6) are macrocycles such as cyclens (**6**) and cyclams (**7**), which show excellent complexation properties towards transition metal ions [110]. Special classes of crown ethers are pyridino crowns (**9**), with one or more oxygen atoms replaced by pyridino moieties in the polyether chain, or azacrown ethers **8**, with a certain number of nitrogen atoms instead of oxygen in the macrocycle.

**Figure 7:**
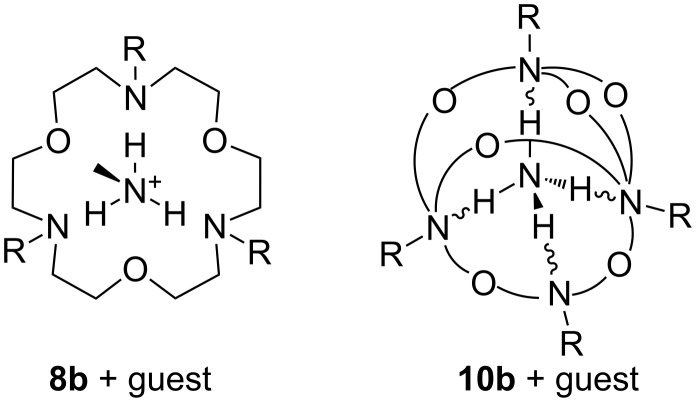
Binding of ammonium to azacrown ethers and cryptands [111–113].

A combination of both, triaza crown ether, with alternating nitrogen and oxygen atoms in the ring (**8b**), can be employed to enhance the selectivity for ammonium ions in comparison to potassium ions. It provides a sufficient number of binding sites for ammonium ions, but fewer for potassium ions compared to 18-crown-6 (Figure 7). The interaction is particularly advantageous when the number of complementary binding sites is maximal (**10b**).

Azacrown ethers with an additional side arm attached on the nitrogen of the macrocyclic ring may have, compared to the related parent crown ether, enhanced cation-binding. Crown ethers with linear or branched heteroatom-containing podand arms – depending on the connection point either *N*-pivot or *C*-pivot lariat ethers – exhibit increased guest specificity [106,114]. This argument holds for polyether compounds with two podand arms, bibraccial lariat ethers. Bridging the ring with the arm leads to cryptands, bicyclic (**10a**) or polycyclic (**10b**) crown ethers [115]. If the moiety is “tricyclic closed” via the two nitrogen atoms, the resulting cryptand **10a** permits cation encapsulation [116] (Figure 7). On inclusion in the cavity of the cryptand, the guest is shielded by three or more polyether bridges. As a result of this encapsulation, cryptands form more stable complexes than coronands (*K*_a_ = 10^6^ for NH_4_^+^ in methanol at 25 °C). In addition, solution thermodynamics of amino acids with **4** and **10a** confirm these facts [117].

Macrotricyclic cryptand **10b** exhibits a substantial enhancement in ammonium vs. potassium ion selectivity in comparison to crown and azacrown ethers, as determined by both calorimetric [104] and NMR studies [118]. The high selectivity over potassium ions has been attributed to the tetrahedral binding site geometry that favors complexation of the tetrahedral ammonium ion over that of the spherically symmetrical potassium ion, underlining the particular importance of hydrogen bonding and symmetry considerations in the design of ammonium ion recognition sites. Differences between these types of ligands also show up in the kinetics of complex formation. The conformationally rigid cryptands complex slower than coronands and these in turn are slower than the flexible podands. In contrast to crown ethers, the three dimensional cryptands display peak selectivity in cation binding. The cavities are more rigid and unable to adapt to bind cations that are too small or too large for the cavity.

The large body of published work on crown ether synthesis [119] and crown ether ammonium ion binding [120] cannot be covered comprehensively in this review, and therefore we refer the reader to recent overviews. Very recent publications of cryptands for ammonium ion recognition are rare. Crown ethers and azacrowns are widely used, and we will therefore focus on these two moieties. An excellent review covering concepts, structure and ammonium ion binding of crown compounds is available [121]. For the highly dynamic motion of 18-crown-6 in complexation/decomplexation processes [122–123] and an interesting closer view on the binding of ammonium ions to 18-crown-6 and its competition with potassium ions [124] we refer the reader to the articles of Schalley and Kimura.

In the following we discuss recent examples of ammonium ion binding compounds which contain crown ether substructures but are more complex in structure than the parent compounds.

#### 2.2. Ammonium ion binding by more complex crown ethers

**Figure 8:**
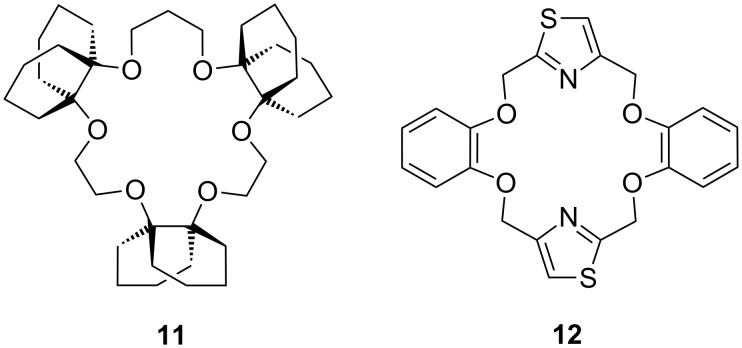
A 19-crown-6-ether with decalino blocking groups (**11**) and a thiazole-dibenzo-18-crown-6-ether (**12**).

An ammonium ionophore with better sodium selectivity than the natural antibiotic nonactin was developed based on a 19-membered crown compound (**11**) (Figure 8). Increased selectivity for ammonium ions over smaller and larger cations [125] was achieved by the introduction of decalino subunits which prevent a folding of the receptor to coordinate smaller cations and add bulkiness to block larger cations from entering the cavity. This compound was found to exhibit a high ammonium ion selectivity over K^+^, similar to nonactin, and over Na^+^ [log *K*_NH4+, K+_ = −1.0 (nonactin −1.0), log K_NH4+, Na+_ = −3.5 (nonactin −2.6) [126]] in an ion selective electrode (ISE). It had an almost Nernstian response (58.1 mV/decade) in the range 5 × 10^−6^–10^−1^ M ammonium ion activity, reflecting a similar detection limit as nonactin.

Similarly, Kim et al. investigated the use of a thiazole containing dibenzo-18-crown-6 derivative (**12**) as an ammonium ionophore (Figure 8) in an ISE sensor and reported a strongly enhanced selectivity for ammonium ions over sodium ions, and a slightly higher selectivity vs. potassium ions in comparison to nonactin [127] [log *K*_NH4+, K+_ = −1.3 (nonactin −1.0), log *K*_NH4+, Na+_ = −3.9 (nonactin −2.6) [126]]. This ionophore exhibited a similar detection limit of ~3 × 10^−6^ M compared to nonactin (1 × 10^−6^ M) [128] in an ISE sensor format. This design was primarily based on size-fit factors. In addition, the aromatic units increase rigidity and the thiazoles provide hydrogen bonding sites.

**Figure 9:**
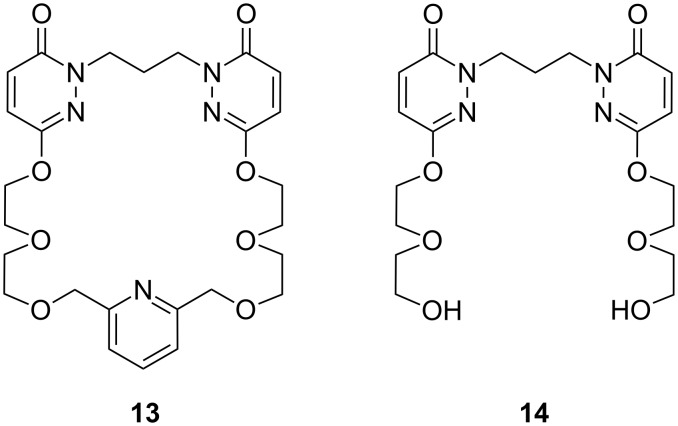
1,3-Bis(6-oxopyridazin-1-yl)propane derivatives **13** and **14** by Campayo et al.

Campayo et al. examined acyclic compounds containing the 1,3-bis(6-oxopyridazin-1-yl)propane and the corresponding heteroaromatic macrocycles containing pyridine units [129] (Figure 9). The cyclic receptor **13** is a most effective carrier of ammonium ions (ν = 57 μM h^−1^) and exhibits an excellent selectivity for NH_4_^+^ in relation to three metal cations investigated (NH_4_^+^/Na^+^ = 9.2, NH_4_^+^/K^+^ = 9.5, NH_4_^+^/Ca^2+^ = 11.8). The acyclic intermediate **14** shows efficient carrier properties for NH_4_^+^ ions and excellent selectivity in NH_4_^+^ transport in relation to K^+^ (NH_4_^+^/K^+^ = 73), which was almost seven times higher than that for nonactin [126]. An impressive selectivity in relation to Ca^2+^ (NH_4_^+^/Ca^2+^ = 146) was also observed. The formation of a pseudocavity by intramolecular hydrogen bonding in **14** and contribution to the binding of the host’s oxyimino part were suggested by molecular modeling of the ammonium complex.

In ammonium ions, where hydrogen atoms arereplaced by organic residues, the substituent will influence the binding. The co-ordination of primary ammonium ions salts with varying steric demand was investigated. The sensing ability of fluorescently labelled 1,10-diaza-18-crown-6 (**16**) was compared to the analogous monoaza-18-crown-6 coumarin sensor (**15**) [130]. The co-ordination experiments were monitored both by fluorescence and ^1^H NMR spectroscopy in CH_2_Cl_2_/CDCl_3_/CD_3_OD 90/9/1 v/v/v %. According to the NMR titrations, sensor **15** shows the highest affinity, two orders of magnitude greater than that of **16a** (Table 3). The stoichiometry of the complexes with *n*-butylammonium perchlorate was established as 1:1 in all cases. For ammonium salts of increased steric demand, the binding values generally decrease.

**Table 3:** Binding constants of **15** and **16**.

Perchlorate of	log *K*_ass_ (**15**)	log *K*_ass_ (**16a**)	log *K*_ass_ (**16b**)

*n*-butylamine	6.0	3.5	4.5
*tert*-butylamine	4.6	2.8	4.5
neopentylamine	5.2	2.8	5.1

**Figure 10:**
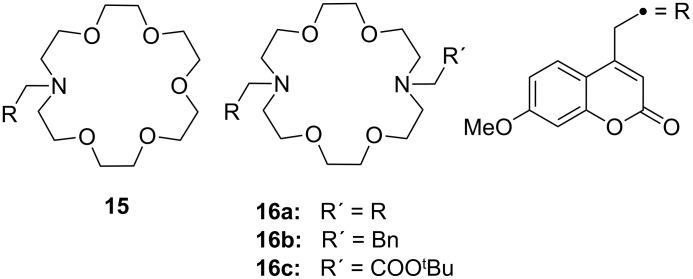
Fluorescent azacrown-PET-sensors based on coumarin.

The 18-crown-6 based PET sensors output was linked to the changes in the sensors’ conformational dynamics on complexation (Figure 10). The fluorescence enhancements upon guest addition of the diaza compounds **16** (140 to 170 fold) were three to four times higher than that of the monoaza receptor **15** (only 40 fold increase). The changes in the conformational mobility of these sensors induced by guest binding have a profound effect on their signaling.

#### 2.3. Enantioselective recognition of chiral ammonium ions by crown ethers

Chiral ammonium salts are found in many biologically active molecules. The enantioselective discrimination of such molecules is of interest as the biological properties of enantiomers may differ [131]. Since Cram et al. synthesized BINAP-crown ethers, which were the first enantioselective receptors for primary organoammonium salts [132] leading to a novel separation technique [133], a great number of attempts have been made to distinguish chiral ammonium ions by chiral crown ethers [134]. Amino acids and their derivatives are of particular interest [131]. Chiral macrocyclic ethers and their derivatives are typical receptors for enantioselective recognition of primary organoammonium salts [135–144]. Recent examples will be discussed.

**Figure 11:**
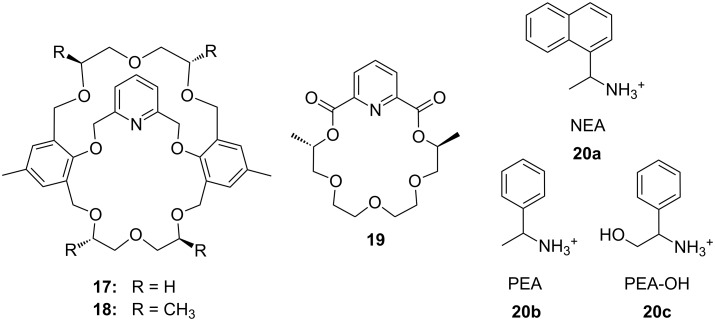
Two different pyridino-cryptands (**17** and **18**) compared to a pyridino-crown (**19**); chiral ammonium ions as guests (**20a–c**).

Pyridino crown receptors were extensively studied for this purpose by Huszthy et al. [145] and Izatt, Bradshaw and co-workers [131,146]: An achiral (**17**) and a chiral pyridine-based macrobicyclic cleft (**18**) were prepared [147] and compared to pyridine-18-crown-6 without the additional podand bridge (**19**) [148] (Figure 11). Compound **17** formed complexes in CH_3_OH/CHCl_3_ (1:1, v/v) with primary ammonium salts with binding strengths around 10^3^ M^−1^ as evidenced by a significant change in the ^1^H NMR spectrum. The strong intermolecular binding observed is attributed to the 3-point hydrogen bonding of the ammonium hydrogen atoms to the pyridine nitrogen atom and two of the oxygen atoms within the ring [149]. Binding strengths for **18** are slightly higher than for **17**. Compared to (*S,S*)-**19**, macrobicyclic (*S,S,S,S*)-**18** shows an improved stereoselective recognition towards NEA (1-naphthyl-ethyl ammonium salt, **20a**) in its three-dimensional cavity. A large difference in stabilities between the complexes of (*R*)- and (*S*)-NEA with (*S,S,S,S*)-**18** (Δlog *K*_ass_ = 0.85) is observed in a 2:8 (v/v) EtOH/C_2_H_4_Cl_2_ solvent mixture, while the Δlog *K*_ass_ value for (*R*)- and (*S*)-NEA interactions with (*S,S*)-**19** is 0.46 in the same solvent mixture. This high degree of enantiomeric recognition was attributed to an increase in molecular rigidity by introducing a second macrocyclic ring in the monocyclic pyridino crown ligand. Positive values of entropy changes for **18**-NEA interactions, as compared to **19**-NEA interactions (which show negative values of entropy changes) suggest a smaller conformational change of ligand **18** during complexation.

**Figure 12:**
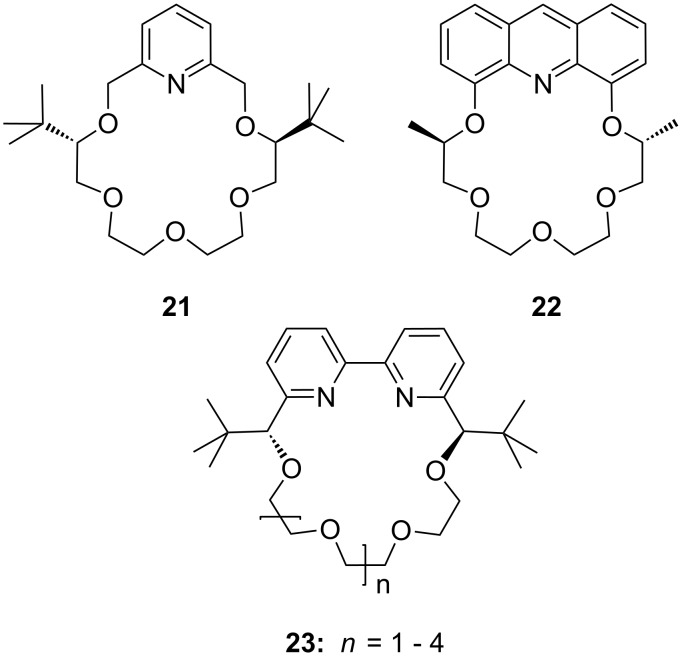
Pyridino-18-crown-6 ligand (**21**), a similar acridino-18-crown-6 ligand (**22**) and a structurally related bispyridyl (bpy)-18-crown-6 receptor **23**.

Pyridino crown systems proved to be advantageous for enantiodiscrimination in the extensive studies of Izatt and Bradshaw. Other groups employed the principle for the preparation of other chiral receptors (Figure 12): A series of enantiomerically pure chiral pyridino-18-crown-6 ligands were prepared by Samu et al. [150] and their ability to act as enantioselective hosts for primary ammonium salts was demonstrated with the two enantiomers of NEA [151]. The equilibrium constants were measured in a CD_3_OD/CDCl_3_ mixture by NMR spectroscopy. The best example (*R,R*)-**21** (R = ^t^Bu) shows a four times higher log *K*_ass_ for the *S*-enantiomer over the *R*-enantiomer of the guest, being more selective as the former examples, but a weaker binder (log *K*_ass_ < 10^3^ M^−1^).

Structurally similar acridino-18-crown-6 ligands like **22** were studied by the same group and the association process between ligands and organic ammonium ions monitored by changes in their photophysical properties in acetonitrile [152]. With the enantiomerically pure (*R,R*)-ligand good binding and enantiodiscrimination in favor of the *S*-enantiomers of PEA (**20b**) [150] (*K*_ass_ = 2.3 × 10^6^ M^−1^) and NEA (*K*_ass_ = 1.7 × 10^6^ M^−1^) over the corresponding *R*-enantiomers (*K*_ass_ = 4.4 × 10^5^ M^−1^ and *K*_ass_ = 3.4 × 10^5^ M^−1^, respectively) was observed.

This optically active dimethylacridino-18-crown-6 ether (*R,R*)-**22** showed higher enantioselectivity towards NEA (**20a**) and PEA (**20b**) than its comparable pyridino analogue (*S,S*)-**21** (R = Me instead of ^t^Bu) [152]. The higher enantioselectivity was rationalized by the stronger π–π-interaction of the extended π-system of the acridine unit and the more rigid conformation of host molecule. An interesting application was demonstrated by Lakatos: Molecule **22** was attached to a silica gel surface to produce a stationary phase for enantioseparation of racemic protonated primary arylalkyl amines [153].

Comparable enantioselectivities with a stronger co-ordinating ligand can be achieved using a crown ether bearing a bispyridyl (bpy) unit in the ring (**23**). A series of these *C*_2_-symmetric 2,2-bipyridine-containing crown macrocycles have been developed by Lee et al. [154] who studied their enantiomeric recognition properties towards a number of amino acid derivatives and chiral organic ammonium salts using UV–vis and NMR methods. The macrocycles were found to be strong chelating agents for primary organic ammonium salts with binding affinities *K*_ass_ up to 4.8 × 10^5^ M^−1^ in CH_2_Cl_2_ with 0.25% CH_3_OH. The bpy-crown macrocycle with *n* = 1, reflecting the pseudo 18-crown-6 type structure, exhibited the best properties and the highest enantioselectivity towards the *S*-enantiomer of phenylglycine methyl ester hydrochloride with a *K*(*S*) to *K*(*R*) ratio of 2.1 (ΔΔG_0_ = −1.84 kJ mol^−1^). The Job’s plot analysis supported the 1:1 stoichiometry of the host–guest complex. An analysis of the structure–binding relationship showed that the aromatic subunit and the ester group of the ammonium guests are both important for achieving high enantioselectivity.

**Figure 13:**
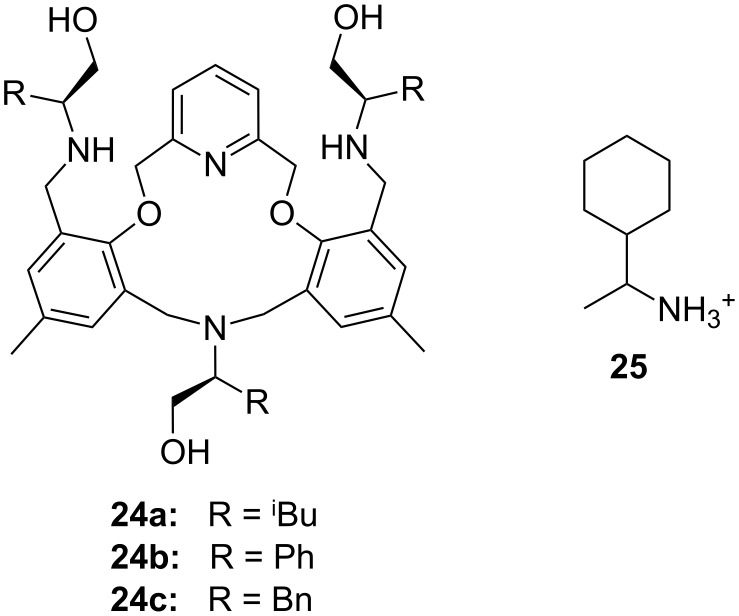
Ciral pyridine-azacrown ether receptors **24**.

The enantiomeric recognition of a different pyridino crown type ligand bearing aminoalcohol subunits on the exterior (Figure 13) were investigated by UV titration in chloroform [155]. The hosts formed very stable 1:1 complexes with α-phenylethylamine hydrochloride (**20b**) and α-cyclohexylethylamine hydrochloride (**25**) with relatively similar binding constants (10^4^ M^−1^) as calculated by a modified Benesi–Hildebrand equation. A preference for enantiomers with an (*S*) absolute configuration for both amine salts was found: Host **24a** bearing isobutyl groups shows an enantiomer recognition factor of 2.0 and 5.0 (*K_S_/K_R_*), which corresponds to approximately 33% and 67% *ee* for **20b** and **25**, respectively. For the host bearing a phenyl residue (**24b**) similar factors of 2.1 and 5.0 (*K_S_/K_R_*) corresponding to approximately 36% and 67% *ee* for **20b** and **25**, were observed. With the benzyl substituted moiety (**24c**) a far weaker discrimination was found. Hydrogen bonding of the alcohols combined with π–π staking, π–charge interaction and steric complementarity were assumed to be responsible for the enantioselective recognition.

**Figure 14:**
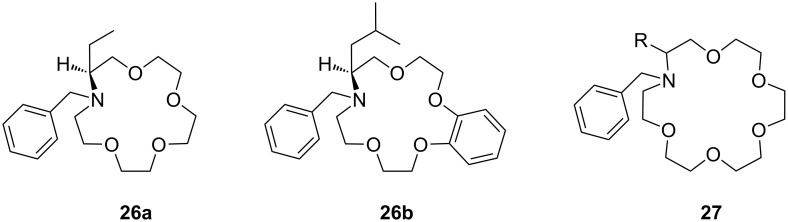
Chiral 15-crown-5 receptors **26** and an analogue 18-crown-6 ligand **27** derived from amino alcohols.

Even better enantioselectivities than with pyridino crowns were observed with chiral azacrown compounds (Figure 14), but the binding constants were for comparable cases approximately one order of magnitude lower. Togrul et al. [156] and Turgut et al. [157] examined several chiral monoaza-15-crown-5 ethers based on chiral aminoalcohols and investigated the effect of the substituent at the stereogenic centre on the enantioselectivity. The benzocrown derivative of *S*-leucinol and the 15-crown-5 prepared from (*R*)-(−)-2-amino-1-butanol were found to be the most effective examples [158]. Both molecules show enantioselectivity towards (*R*)-**20b** perchlorate compared to (*S*)-**20b** perchlorate [151]: The aggregate was for **26b** 4.76 times more stable for the *R*-enantiomer than with the *S*-form (ΔΔG_0_= −1.73 kJ mol^−1^; *K*_ass,R_ = 9.8 × 10^4^ dm^3^ mol^−1^, *K*_ass,S_ = 2.2 × 10^4^ dm^3^ mol^−1^). In the case of **26a** they observed a ratio of *K_R_/K_S_* = 4.46 (ΔΔG_0_= −3.7 kJ mol^−1^; *K*_ass,R_ = 9.5 × 10^3^ dm^3^ mol^−1^, *K*_ass,S_ = 4.8 × 10^3^ dm^3^ mol^−1^).

Enantiomeric recognition of chiral primary ammonium perchlorate salts was investigated with analogous chiral mono aza-18-crown-6 derivatives such as **27** [159]. For the isobutyl compound (**27**, R = ^i^Bu), the host exhibited the highest binding constant and the best enantiomeric selectivity ability towards 1-phenylethylammonium perchlorate isomers (**20b**): The complex with the *R*-isomer (*K*_a_ = 3.3 × 10^4^ dm^3^ mol^−1^) was 2.5 times more stable than the one with the *S*-configuration (*K*_a_ = 1.3 × 10^4^ dm^3^ mol^−1^) [158].

**Figure 15:**
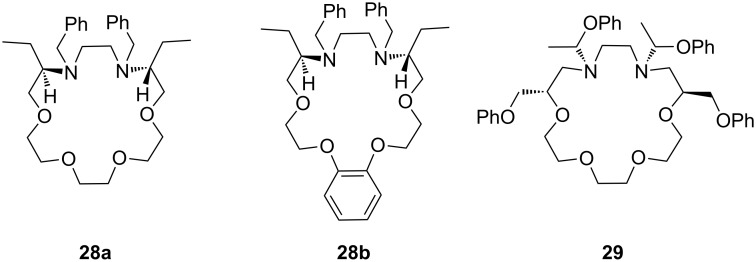
*C*_2_-symmetric chiral 18-crown-6 amino alcohol derivatives **28** and related macrocycles.

Turgut et al. investiged the corresponding *C*_2_-symmetric chiral diaza-18-crown-6 ethers **28a** and **28b** derived from chiral (*R*)-(−)-2-amino-1-butanol [160] (Figure 15). The association constants, measured by UV–vis spectroscopy in methanol/chloroform solvent mixture, revealed for *S*-, *R*-Ala-OMe hydrochloride the highest value for both macrocycles (*K*_a_ = 1.5 × 10^4^ dm^3^ mol^−1^) as calculated by a modified Benesi–Hildebrand equation, but without pronounced chiral discrimination. The highest enantioselectivity was observed in the case of Trp-OMe hydrochloride (*K*_R_/*K*_S_ = 12.5) with a binding strength in the same order of magnitude as observed for the alanine ester. This was the highest factor reported to date for such systems. The authors reasoned that steric and π–π-interactions with the crowns phenyl substituents are the decisive factor for the enantioselective recognition.

Recently, Turgut et al. reported a comparable series of *C*_2_-symmetric chiral aza crown ether macrocycles (**29**) based on (*S*)-3-phenyloxy-1,2-propanediol and (*S*)-1-methyl-1,2-propanediol for the enantiomeric recognition of amino acid ester derivatives [161]. The four similar macrocycles have been shown to be complexing agents for primary organic ammonium salts by ^1^H NMR titration. The best example, the depicted host **29**, exhibited enantioselective bonding toward the *R*-enantiomer of phenylalanine methyl ester hydrochloride with *K*_R_/*K*_S_ of 6.87 in CDCl_3_ with 0.25% CD_3_OD. The binding constants were far lower as in the former examples.

**Figure 16:**
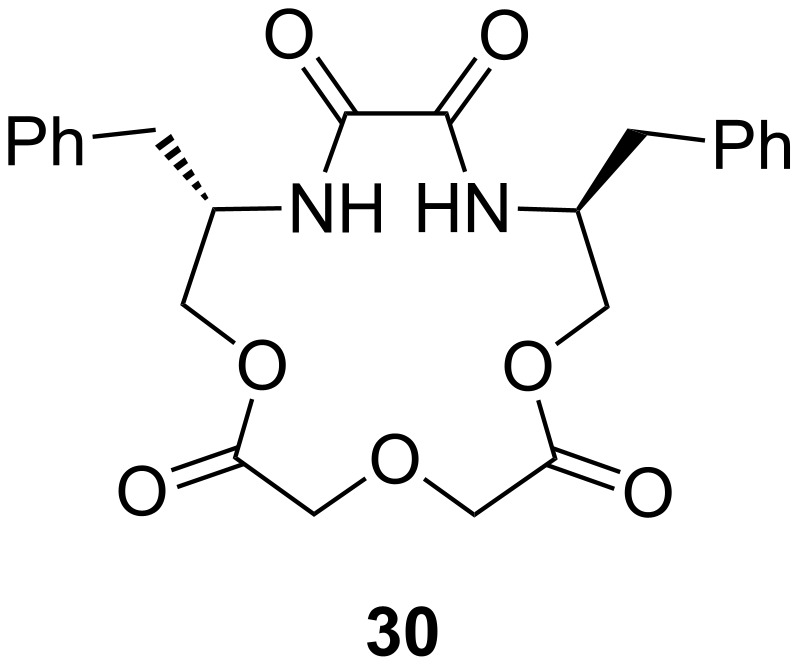
Macrocycles with diamide-diester groups (**30**).

Related macrocycles **30** with diamide-diester groups derived from dimethyloxalate and amino alcohols (Figure 16) also showed a considerable binding affinity and enantiomeric discrimination of aromatic amine salts [162]. The binding properties were evaluated by ^1^H NMR titration in acetonitrile. For the (*R,R*)- and (*S,S*)-configurated host with a phenyl residue, the highest differences in the *K*_ass_ values were observed: (*R*)-NEA and (*S*)-NEA (**20a**) [151] to (*S,S*)-**30** and (*R,R*)-**30** (R = Ph) show ratios of *K*_S_/*K*_R_ = 5.55 and *K*_R_/*K*_S_ = 3.65, respectively. A general tendency for the host to include the guests with the same absolute configuration was found. The amide and ester groups ensure a high rigidity of the host. The highest binding constant of 7.8 × 10^3^ M^−1^ was found for the complex of phenyl substituted (*R,R*)-**30** with the *R*-enantiomer of the guest.

**Figure 17:**
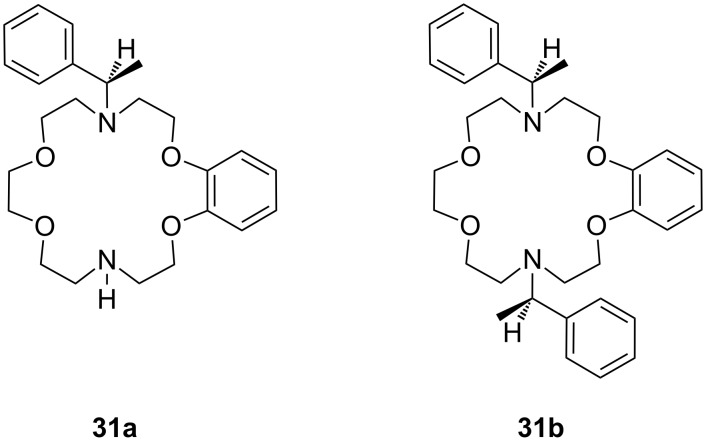
*C*_2_-symmetric chiral aza-18-crown-6 ethers (**31**) with phenethylamine residues.

Chiral side arms derived from phenethylamine attached to diaza-18-crown-6 ethers **31** (Figure 17) effectively enable the molecular recognition of aromatic amino acid potassium and sodium salts [163] as shown in the selectivity order Phe > Thr > Ala. The ability of the crown ethers to co-ordinate to the salts was investigated using UV–vis titration in a solution of acetonitrile/water (50:1). The highest affinities of 4 × 10^4^ M^−1^ were obtained with the monoaromatic ring system **31a** for the potassium salt of *S*-Phe. The cavity of the macrocycle plays an important role in recognition: A dibenzo substitution on the diazacrown ether may close the cavity due to steric hindrance of the arene units on the ring and the resulting π–π-interaction between the two aromatic moieties on the ring. However, π–stacking interactions between the aromatic moiety and aromatic part of the amino acid contributes to the overall binding strength of the receptor.

**Figure 18:**
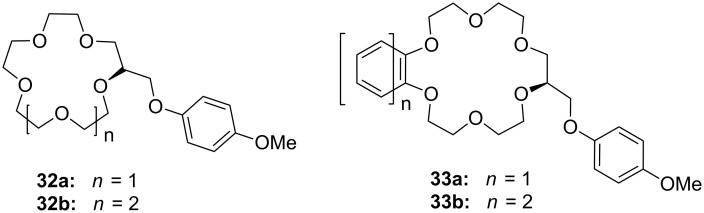
Chiral C-pivot *p*-methoxy-phenoxy-lariat ethers.

In transport experiments, chiral lariat ethers (Figure 18) show an increased flux of amino acids or their carboxylate salts and enantiomeric discrimination (Table 4): With preference for the *R*-enantiomers, the benzo- and naphtho-18-crown-6 **33a** and **33b** generally revealed a larger flux of the aromatic amino acids or their salts than hosts **32a** and **32b** [164]. This was attributed to a strong π–π stacking interaction. The highest flux values and enantiomeric selectivities were obtained for the *R*-enantiomers of tyrosine and its potassium salt. The more pronounced enantioselectivity of tyrosine may be explained by hydrogen bonding and the favorable π–π interaction between the hosts’ side arm and the aromatic moiety of guests. The higher enantioselectivity of potassium salts in comparison to other salts was explained by apical-π or a sandwich-type supramolecular complex due to the larger size of the ion.

**Table 4:** Fluxes and enantiomeric selection behavior of substance class **32** and **33**.

	**32a**		**32b**		**33a**		**33b**	
	*f*_72_×10^8^ (mol m^−2^ s^−1^)	α_T_	*f*_72_×10^8^ (mol m^−2^ s^−1^)	α_T_	*f*_72_×10^8^ (mol m^−2^ s^−1^)	α_T_	*f*_72_×10^8^ (mol m^−2^ s^−1^)	α_T_

*S*-Tyr	3.05	13.7	11.01	3.5	7.96	4.9	2.56	15.5
*R*-Tyr	41.87		38.04		38.73		39.81	
*S*-Tyr K^+^	4.62	8.3	10.81	3.5	7.18	5.2	2.75	14.1
*R*-Tyr K^+^	38.34		37.65		37.45		38.83	

**Figure 19:**
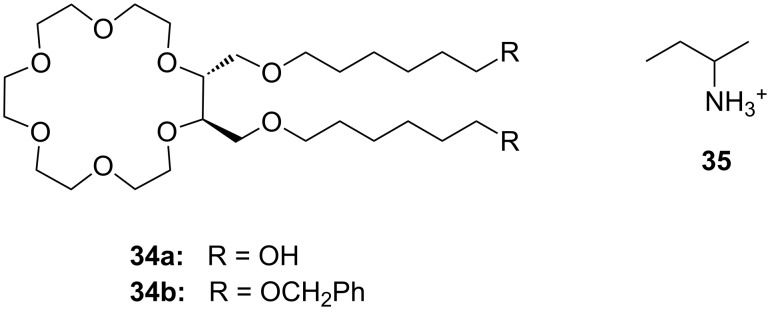
Chiral lariat crown ether **34**.

The approach to introduce chirality for a similar function by the introduction of C-pivot podand arms (Figure 19), resulting in stereogenic centres, was presented by Colera et al. [165]. The properties of the compounds were evaluated with two different chiral alkylammonium picrates, (+)-(*S*)- and (−)-(*R*)-**35** (AmI) and (+)-(*R*)- and (−)-(*S*)-**20b** (AmII) in acetonitrile. The ligands (*R,R*)-**34b** and (*R,R*)-**34a** showed enantioselective binding: (*R,R*)-**34b** favored (*R*)-AmI over (*S*)-AmI and (*R*)-AmII over (*S*)-AmII by a Δlog *K*_ass_ of 2.06 and 3.23, respectively. Similar results were observed with (*R,R*)-**34a** with Δlog *K*_ass_ = 2.64 and 2.43 for AmI and AmII. These results indicated that the presence of the phenyl rings in ligand (*R,R*)-**34b** not only gives rise to higher complexation constants with (*R*)-AmII than with (*R*)-AmI (log *K*_ass_ = 5.42 and = 4.61, respectively) but also increases the enantioselective recognition. In addition, racemic aqueous solutions of the ammonium salts have been enriched in the *R*-enantiomer after extraction experiments, with the best results obtained for (±)-AmII with an *ee* of 33%.

It is difficult to compare the results of the previous examples since their properties were investigated in different solvent mixtures and by different methods. However, this underlines the versatility of the systems published: For different conditions and separation problems several approaches are available.

A general trend is observable: 18-crown-6-systems reveal higher binding constants then 15-crown-5-systems, due to the better size fit of the guest ion. Aromatic substituents lead to better recognition and enantiomeric excess (up to 70%) with aromatic guests such as NEA (**20a**) or phenylglycinol (**20c**). For tryptophan (**81b**) the best results were achieved with selection factors of one enantiomer over the other up to 13 fold, corresponding to over 90% *ee*. This is explained by π–π-interactions.

Besides chiral substituents on the crown ether ring, chiral groups in the ring can be employed for enantioselection of guest ions: Stoddart determined the stability of complexes of D-mannitol based crown ethers with ammonium cations by NMR spectroscopy [166]. In another example fructopyrano-crown ethers with different ring sizes were employed [167].

**Figure 20:**
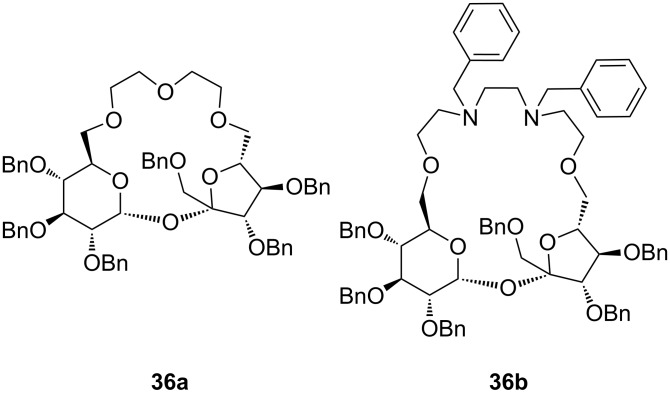
Sucrose-based chiral crown ether receptors **36**.

The chiral azacoronands **36a** and **36b** based on sucrose (Figure 20) display high enantioselectivity in the complexation of phenylethylammonium chlorides [168]. The stability constants of these receptors in acetone towards ammonium cations (NMR titration of NH_4_SCN) were 560 M^−1^ for **36a** and 230 M^−1^ for **36b** [169]. In NMR titration experiments in chloroform the receptors showed the preferential complexation of the (*S*)-ammonium salt with the highest value (*K*_ass_ = 1244 M^−1^) for the complex of compound **36a** with α-phenylethylammonium chloride. The complex with the (*R*)-amine was of lower affinity (*K*_a_ = 837 M^−1^, *K_S_*/*K_R_* = 1.84). Although the stability constants of **36b** with the (*S*)-amine were lower than for **36a** (*K*_ass_ = 945 M^−1^), it has interesting complexing abilities: The macrocycle did not complex the (*R*)-enantiomer of α-phenylethylamine. In all cases a Job’s plot confirmed a 1:1 stoichiometry of the aggregates.

**Figure 21:**
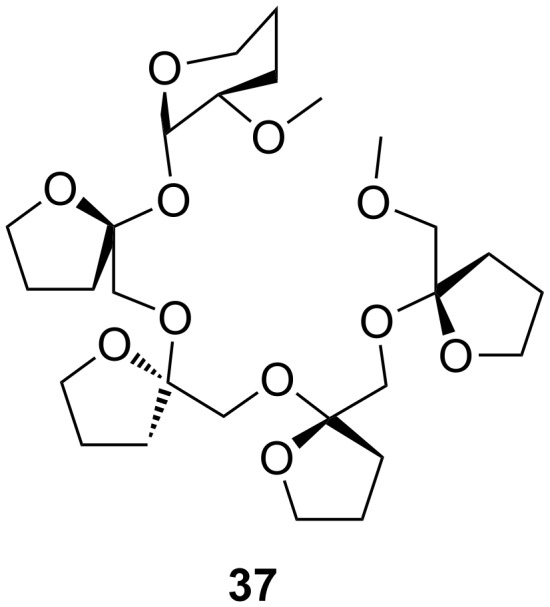
Permethylated fructooligosaccharide **37** showing induced-fit chiral recognition.

The use of cyclodextrin type structures in chiral discrimination is well documented [170–173]. In a recent example (Figure 21), Shizuma and Sawada demonstrated a high degree of chiral discrimination between amino acid ester salts with a permethylated fructooligosaccharide (pentasaccharide) by an induced-fitting chiral recognition mechanism with amino acid ester salts [174]: ValOPr***_i_*** gave *I_R_*/*I_S_*_-D_*_n_* = 0.14 corresponding to ΔΔ*G*_enan_ = 1.2 kcal mol^−1^ with *S*-selectivity and PheOPr***_i_*** led to *I_R_*/*I_S_*_-D_*_n_* = 0.18 corresponding to ΔΔ*G*_enan_ = 1.0 kcal mol^−1^, also with *S*-selectivity. It was assumed that a pseudo-18-crown-6-ring structure surrounding the ammonium ion was formed by the acyclic methylated pentasaccharide in the complexation. The chiral discrimination was ascribed to the steric effect of the fructofuranose rings of the pentasaccharide and the substituent of a given amino acid ester salt (complexation-induced selectivity). The binding ability of compound **44** in solution (CHCl**_3_**) was determined by UV–vis spectrometry using a picrate anion probe. This is one of the rare examples of podands used for enantioselective recognition.

The pioneering work on this topic was carried out in the 1970s by Cram et al. [135] who studied the chiral recognition ability of binaphtol based chiral macrocycles using the picrate salt extraction method [175].

**Figure 22:**
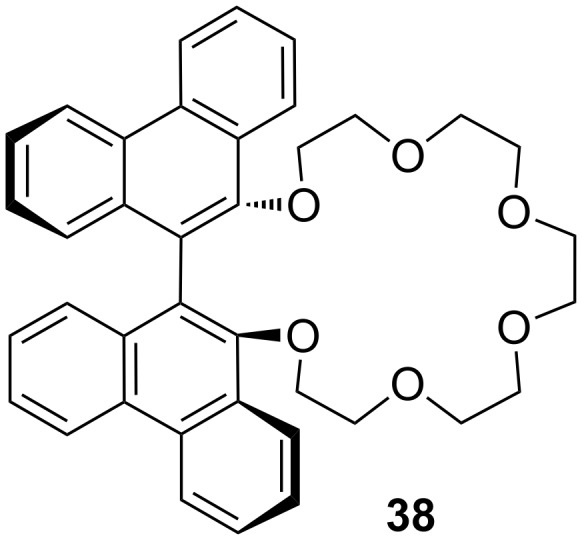
Biphenanthryl-18-crown-6 derivative **38**.

Many examples of chiral receptors have been reported, which exhibit chiral recognition towards cations derived from phenylethylamine. The biphenanthryl-18-crown-6 derivative **38** presented by Yamamoto et al. [176] (Figure 22) displayed one of the highest enantioselectivities towards one enantiomer of phenylethylamine hydrochloride as was demonstrated by liquid/liquid extraction experiments [the respective *ee* values are 42% (*R*) and 45% (*S*)].

**Figure 23:**
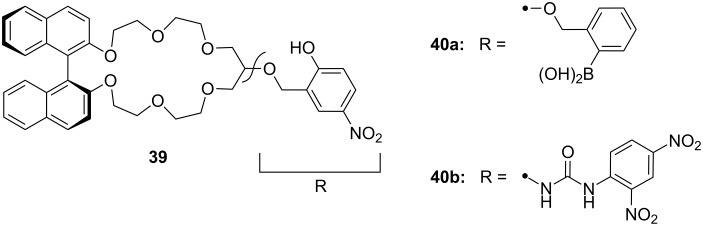
Chiral lariat crown ethers derived from binol by Fuji et al.

Fuji et al. [177] have developed the related chiral lariat crown ether **39** (Figure 23). Its phenolic hydroxyl group converts basic amines into ammonium ions, which are bound more tightly. A salt bridge between the ammonium and the phenolate ions supports the binding process. From UV and NMR titration experiments, the authors derive binding constants for hexylamine of 14 M^−1^ in THF and >10^5^ M^−1^ in DMSO. This is surprising, because an increased ability of the solvent to act as a hydrogen bond acceptor typically leads to decreased binding constants. A significant contribution of the phenolate-ammonium salt bridge or from π–cation interactions is likely. The best enantioselective binding of chiral ammonium ions was observed using phenylglycinol: The *R*-enantiomer (*K*_ass_ = 30 M^−1^) was bound preferentially over the *S*-enantiomer (*K*_ass_ = 9 M^−1^) by a factor of 3.2 in a methanol/acetonitrile solvent mixture.

The authors expanded their approach with two similar binaphthyl crown recognition systems containing phenylboronic acid **40a** and 2,4-dinitrophenylurea **40b** as lariat parts [178] (Figure 23). Host **40a** had 30% extraction efficiency for γ-aminobutyric acid (GABA) in solid–liquid extraction in DMSO, but showed only much lower selectivities for α-amino acids: Boc-*R*-Lys-OH (18.5%), Boc-*S*-Lys-OH (14.1%) and H-*R*-Asp-NH_2_ (8.2%), H-*S*-Asp-NH_2_ (4.3%). The chromogenic host **40b** discriminated amino acids by their length. After extraction, the color of the solvent changed from colorless to yellow due to increased absorbance around 460 nm. The extent of the color change correlates with the affinity for the guest amino acid. ω-Aminohexanoic acid produced the most significant change. Although the color change is visible to the naked eye, the maximum amount extracted (3%) was small.

**Figure 24:**
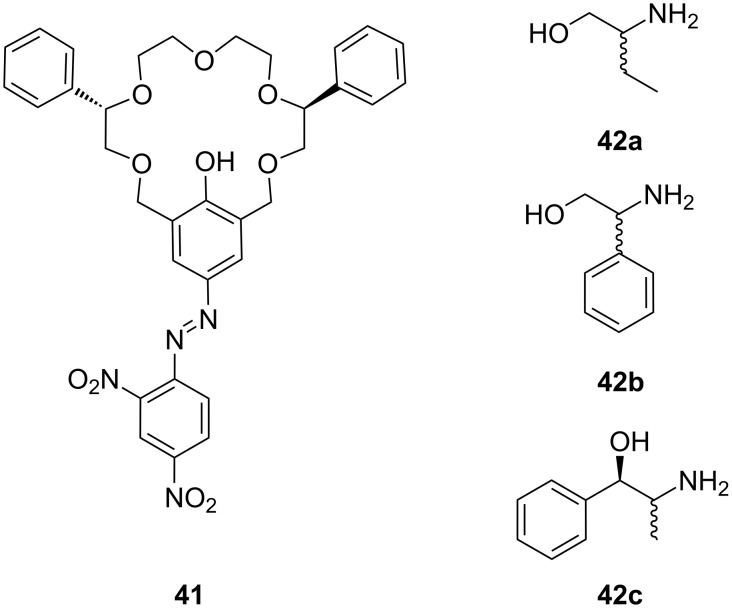
Chiral phenolic crown ether **41** with “aryl chiral barriers” and guest amines.

Homochiral phenolic crown ethers with “aryl chiral barriers” (Figure 24) were investigated and published in 1998 by the group of Naemura [179]. This system displayed, on investigation by UV–vis spectroscopy in chloroform, a good enantiodiscrimination ability in favor of (*R*)-phenylalaninol with an Δ_R-S_ΔG = 6.4 kJ mol^−1^. In succession, Steensma et al. investigated thermodynamic data and conditions for chiral separation of amines and amino alcohols [180]. The azophenolic crown ether was a versatile and a highly enantioselective host for their chiral separation by reactive extraction. Transport from a basic aqueous solution of the racemic mixture in CH_2_Cl_2_ and toluene was followed by UV–vis titration. Compound **41** showed the highest affinity for phenylglycinol (**42b**) with association constants of *K*_ass_ = 1.5 × 10^5^ M^−1^ in CH_2_Cl_2_ and *K*_ass_ = 8.0 × 10^4^ M^−1^ in toluene with a 10 fold higher binding constant to the *R*-enantiomer. In addition, norephedrine (**42c**) and 2-aminobutanol (**42a**) could be separated in an acceptable ratio. The extractant could be reused for further chiral separations without loss of activity or selectivity. Ammonium ion binding by chiral azophenol crowns and of diamines by bisazophenol crown ethers has been summarized in a special review [181].

#### 2.4. Di- and tritopic crown ether receptors for the recognition of bis- and tris-ammonium ions

**Figure 25:**
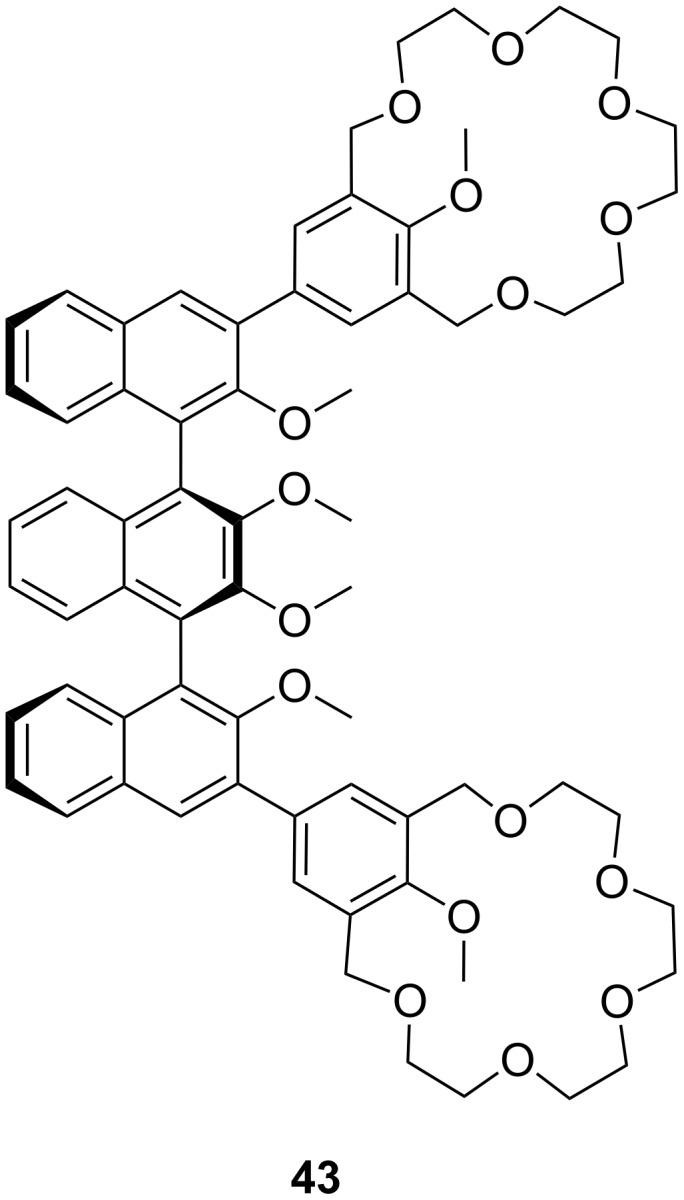
Chiral bis-crown receptor **43** with a meso-ternaphthalene backbone.

Fuji et al. investigated a ditopic receptor **43** to distinguish between the length of α,ω-diamines (Figure 25). The receptor consists of a meso-ternaphthalene backbone and two crown ether rings [182]. Receptor **43** preferably binds and transfers the di-picrate of 1,9-diaminononane and 1,10-diaminodecane from an aqueous solution to CHCl_3._

**Figure 26:**
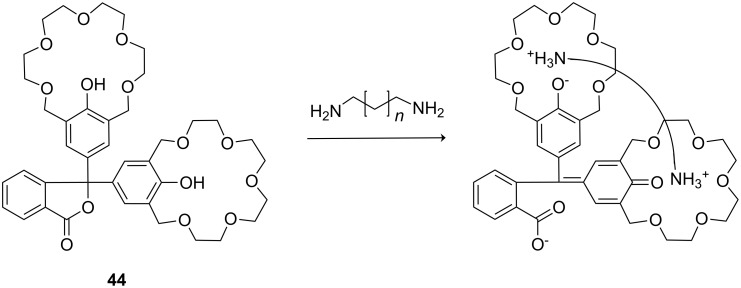
Chromogenic pH-dependent bis-crown chemosensor **44** for diamines.

The group also reported a colorimetric approach for recognition of such guests (Figure 26), a phenolphthalein core substituted with two crown ether moieties [183]. On amine binding, the phenolic hydroxyl groups are deprotonated, which leads to lactone ring opening and the formation of a colored quinone conjugated carboxylate structure. The chemosensor discriminated terminal diamines by length: 1,8-diaminooctane (*K*_ass_ = 1270 M^−1^) and 1,9-diaminononane (*K*_ass_ = 2020 M^−1^) showed the highest binding constants in methanol. Diamines with an alkyl chain length shorter than five carbons were not bound.

Investigation of the stoichiometry of the aggregate formation led to a value of 1.2 to 1.3, because one diamine is bound by the two crown ethers and a second diamine is recruited as the ammonium counter ion of the carboxylate. Addition of an excess of *N*-ethylpiperidine as base established the expected stoichiometry of the aggregate as 1:1. Control experiments with *N*-ethylpiperidine and phenolphthalein without crown ether moieties confirmed the ammonium ion crown ether interaction as being essential for the color response. Unprotected dipeptides showed an affinity to compound **44** if amino groups were present within a suitable distance, for example, as found in dipeptides with a C-terminal Lys. Lys-Lys (*K*_ass_ = 1020 M^−1^) and Gly-Lys (*K*_ass_ = 930 M^−1^) showed the highest affinity constants in methanol/water 10:1 [184].

**Figure 27:**
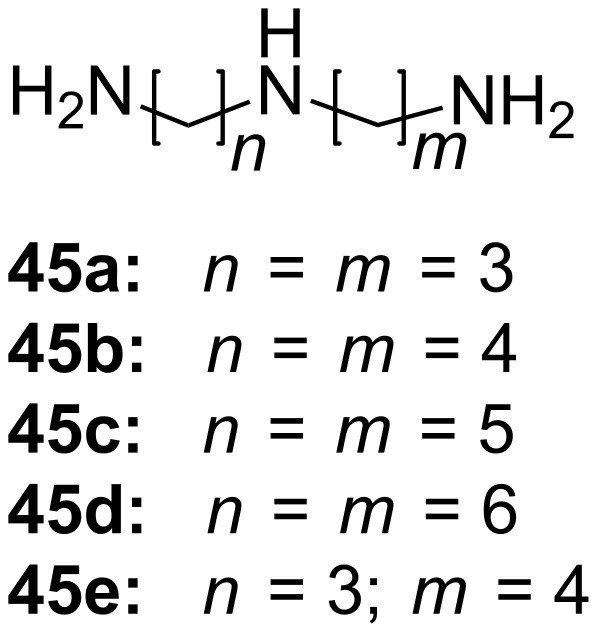
Triamine guests for binding to receptor **44**.

The same host (**44**) is able to signal the length of a linear triamine in a similar manner. Triamines **45a**–**45c** and spermidine (**45e**) (Figure 27) developed a bright purple color by forming complexes with the host in a 1:1 ratio with the inner imino group capturing the carboxylate after lactone ring opening. The color develops over a limited temperature range and therefore can be also used as a visible index of temperature. The association constants (*K*_ass_) as well as molar absorption coefficients (ε) were determined by UV–vis titration. For triamine **45c** thermodynamic parameters Δ*H* = −127.4 ± 6.3 kJ mol^−1^ and Δ*S* = −362.8 ± 21.3 J mol^−1^ K^−1^ were obtained, and temperature dependent measurement of the association constants were measured (*K*_ass_ = 14870 ± 880 M^−1^, ε = 5100 ± 30 at 15 °C; *K*_ass_ = 2270 ± 30 M^−1^, ε = 5080 ± 20 at 25 °C; *K*_ass_ = 1090 ± 10 M^−1^, ε = 4980 ± 10 at 30 °C). Both *K*_ass_ and ε reach maximum values with triamine **45c**.

**Figure 28:**
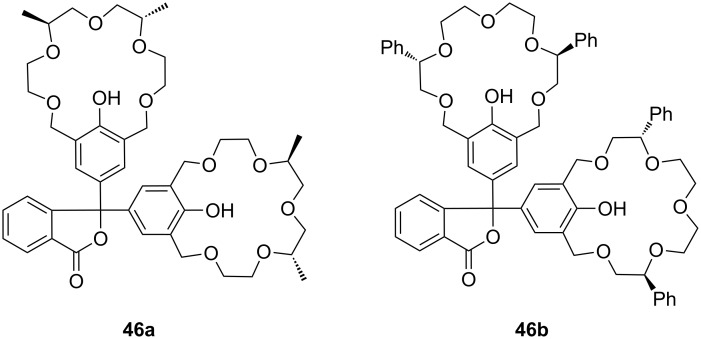
Chiral bis-crown phenolphthalein chemosensors **46**.

Based on this phenolphthalein skeleton, the host was later further developed for use in visual enantiomeric discrimination [185] (Figure 28). Various types of chiral host molecules were examined for their enantioselective color effect in complexation with chiral amino acid derivatives in methanol solution. The methyl substituted compound (*S*,*S*,*S*,*S*)-**46a** showed a particularly prominent selectivity for the alanine amide derivatives with 1,5-pentane diamine and 1,6-hexane diamine: A combination of methyl substituted host (*S*,*S*,*S*,*S*)-**46a** with the *R*-enantiomers developed a purple color, whereas no color development was observed with *S*-enantiomers. When Ala-1,6-hexane diamines with different optical purities were added to the host **46a** solution, a linear relationship was observed between the absorbance (*λ*_max_ = 574 nm) and the *ee* of the added guest. The phenyl substituted compound (*S*,*S*,*S*,*S*)-**46b** showed an even more intensive color change induced by a wide range of (*S*)-α-amino alcohols compared to the corresponding (*R*)-α-amino alcohols. The function, mechanisms and applicability of phenolphthalein crown systems have been recently summarized by Tsubaki [186].

**Figure 29:**
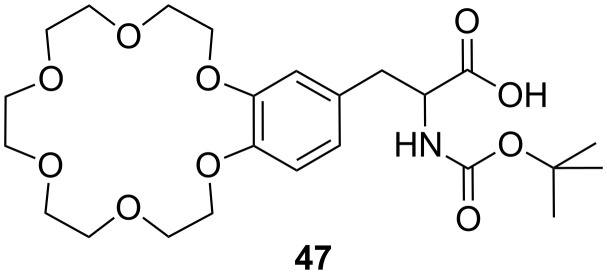
Crown ether amino acid **47**.

Ditopic receptors can consist of two or more crown ether amino acids. The group of Voyer reported crown ether based receptors for diamino and diammonium alkanes [187]. They used crown ether amino acid (CEAA) **19** (Figure 29), which was incorporated twice into an oligo Ala peptide chain.

The receptor structure was modified by varying of the number of Ala residues between the crown ether amino acids from one to three: Boc-Ala-Ala-CEAA-(Ala)_1-3_-CEAA-Ala-*^n^*Pr. 1,9-Diaminononane was found to be the diamine with highest affinity for all three sequences among all tested diaminoalkanes from C_2_ to C_9_. The binding constants were derived from picrate extraction [188] from water into chloroform with 2 × 10^10^ M^−1^ as the highest binding constant. However, binding constants determined by extraction methods may have larger errors [189] and the binding process includes a phase boundary transition. Therefore, binding constants cannot be compared to other systems investigated in homogeneous solutions. Surprisingly, despite the difference in crown ether spacer length in the Voyer’s and Fuji’s systems, both preferentially bind 1,9-diaminononane. To match the distance of the phenolphthalein system, the CEAA units must be connected directly. This indicates that the actual binding conformation of the bis-crown ether-diammonium ion aggregates may be more complex under the experimental conditions. Recently, they reported the application of a similar peptide forming an α-helical amphiphilic peptide nanostructure with cytolytic activity. A potential use of these peptide nanostructures is as pro-drugs that may be activated by a specific proteolytic enzyme to target selectively and destroy undesirable cells [190].

**Figure 30:**
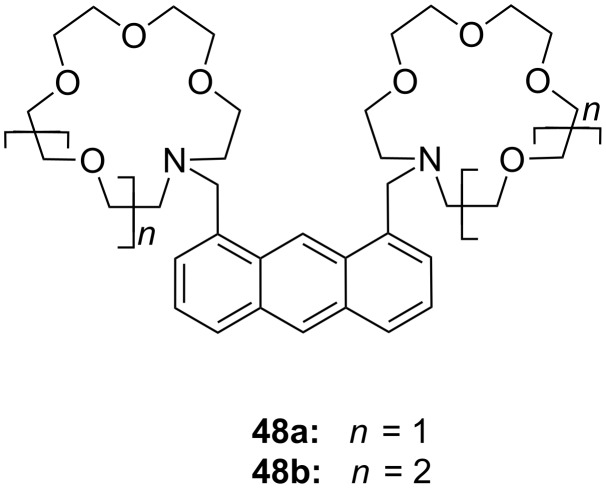
Luminescent receptor **48** for bis-alkylammonium guests.

Kim et al. reported two bis(azacrown)anthracene derivatives **48a** and **48b** (Figure 30) for the recognition and detection of alkyldiammonium ions in ethanol or in a chloroform/methanol mixture (9:1) based on the PET principle [191]. The fluorescence of the anthracene function is quenched by the free electron pairs of the nitrogen atoms. When hydrogen bonds are formed by both nitrogen atoms to the bis-ammonium guests, the photoinduced electron transfer (PET) is inhibited and the system shows an enhanced fluorescence. The binding was dependent on the chain length between the two cations, displaying a maximum stability in the case of the protonated 1,3-diaminopropane. for the bis(aza-15-crown-5) chemosensor **48a** the following binding constants were observed: *K*_ass_ = 4412 M^−1^ for *n* = 3; *K*_ass_ = 272 M^−1^ for *n* = 4; *K*_ass_ = 35 M^−1^ for *n* = 5; *K*_ass_ = 98 M^−1^ for *n* = 6. Compound **48b** showed a similar selectivity towards the guests.

**Figure 31:**
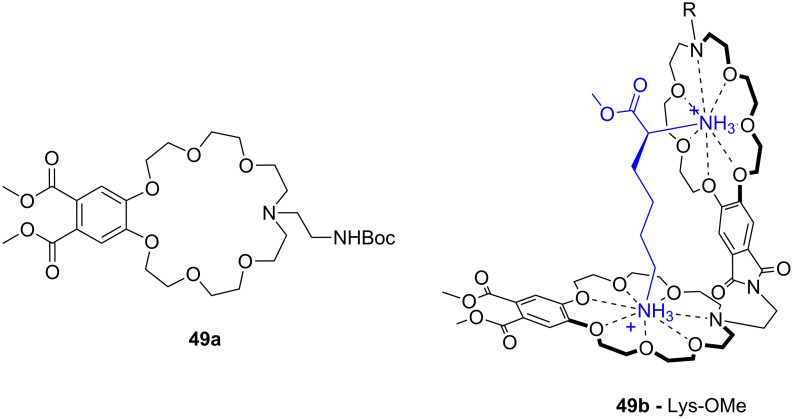
Luminescent CEAA (**49a**), a bis-CEAA receptor for amino acids (**49b**) and the structure of lysine binding.

König et al. combined both principles. They investigated luminescent crown ether amino acid (CEAA) dipeptide (**49b**) (Figure 31) which showed high affinity for ammonium ions with the binding processes signalled by an increase in their emission [192]. In contrast to Voyer’s system, the crown ether moieties are the central part of the CEAA enabling the synthesis of linear receptors. Both crown ether parts in the ditopic receptor bound independently to mono-ammonium guests with similar affinities than monomeric CEAAs. A bis-ammonium guest, such as lysine methyl ester, was co-operatively bound with a higher affinity (log *K*_ass_ = 4.3 for the phthalimide containing part and log *K*_ass_ = 4.7 for the phthalate ester containing part in methanol). The binding affinity increased more than 100 fold in comparison to a single receptor CEAA. The affinity of the bis-CEAA to bis-ammonium ions is distance dependent, which made it possible to distinguish between isomeric small peptides containing a lysine residue in different positions. Peptides with *N*-terminal lysine showed the highest affinity to **49b**. The binding events of the crown ether groups can be monitored independently by changes of their specific emission properties.

**Figure 32:**
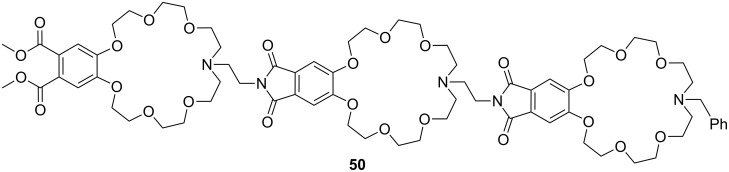
Luminescent CEAA tripeptide for binding small peptides.

The approach was extended to linear tris-CEAA receptors (**50**) for di-lysine peptides [193] (Figure 32). The additional chromophore leads to a stronger emission, which becomes visible to the naked eye, but the extension from bis- to tris-crown ethers does not lead to an increase of ammonium binding affinities as demonstrated by emission titration. Compared to **49b**, comparable binding constants for di-lysine-guests in methanol (log *K*_ass_ = 4.5) and in buffered aqueous solution (log *K*_ass_ = 2.5) are achieved with **50**. The flexible structure of the extended crown ethers and their peptidic guest molecules is a likely rational for the observation: the limited pre-organization of the extended receptor binding sites prohibits an additive or co-operative action of the intermolecular interactions, and illustrates the importance of well balanced entropy and enthalpy contributions in the design of synthetic receptors.

**Figure 33:**
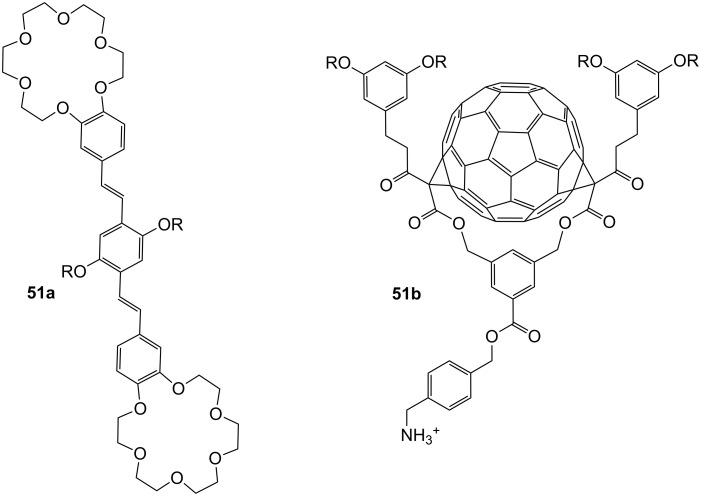
Bis crown ether **51a** self assembles co-operatively with C_60_-ammonium ion **51b**.

More unusual, but demonstrating the wide scope of ammonium ion recognition with crown ethers, are systems which utilize guest self assembly for enhancement of binding strength. The assembly of the C_60_–ammonium cation **51b** with the oligophenylenevinylene derivative bearing two crown ether moieties **51a** (Figure 33) led to the co-operative formation of the 2:1 complex as a result of intramolecular fullerene-fullerene interactions [194]. High stability constants in dichloromethane (log *K*_1_ = 5.6 by luminescence titration and log *K*_2_ = 6.5 by UV absorption) were reported, but due to the small spectral changes upon binding, the binding constants obtained had high errors. The observation was also supported by electrospray mass spectrometry. The co-operative recognition process could be shown by fluorescence quenching experiments: The stability of the supramolecular *syn*-complex is significantly higher than that of its corresponding anti-complex. The combination of several weak interactions such as π–π-stacking and hydrophobic associations between the two C_60_ units was proposed to explain the stronger co-ordination and its ability to self-aggregate.

**Figure 34:**
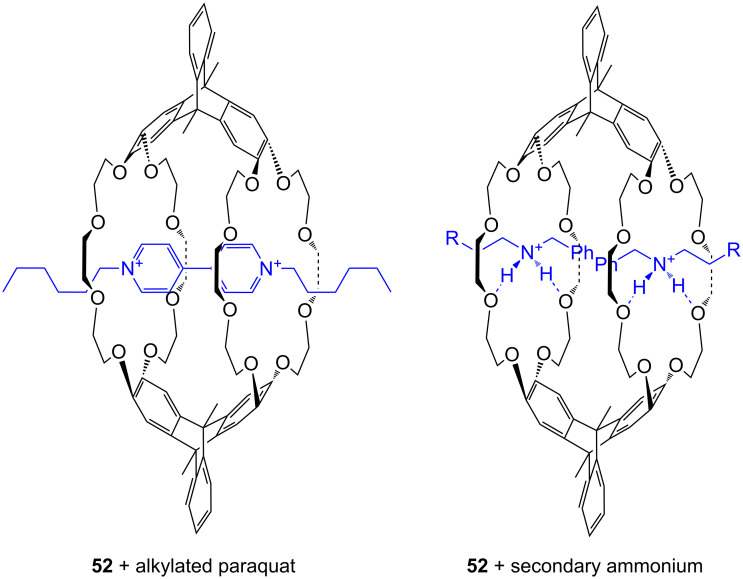
Triptycene-based macrotricyclic dibenzo-[24]-crown-8 ether host **52** and guests.

With larger crown ethers (24-crown-8 and above) secondary amines or pyridylium ions can also be recognized. Such an approach for ditopic crown receptors with enhanced guest selectivity was presented by Chen [195]. A triptycene-based macrotricyclic host **52** containing two dibenzo-[24]-crown-8 moieties (Figure 34) selectively forms stable 1:1 or 1:2 complexes with different functionalized paraquat derivatives and secondary ammonium salts (*K*_ass_ ~ 10^3^–10^4^ M^−1^ in acetonitrile/chloroform). These guest-dependent complexation modes have been confirmed by 2D NMR experiments and X-ray crystallographic analysis. Alkyl substituted paraquat derivatives thread the lateral crown cavities of the host to form 1:1 complexes in chloroform/acetonitrile 1:1 (2–4 × 10^3^ M^−1^) [196]. The host forms a 1:2 complex with two 9-anthracylmethylbenzylammonium salts (R = 9-anthracyl) in the same solvent (*K*_1_ = 8.0 × 10^3^ M^−1^ and *K*_2_ = 1.2 × 10^3^ M^−1^), in which the two 9-anthracyl groups were selectively positioned outside the central cavity. The competing complexation of the host and two different guests, the hexyl-substituted paraquat derivative and a dibenzylammonium salt, can be controlled by the addition of acid or base.

Paraquat and its derivatives are widely used in crown ether rotaxanes and several recent examples of crown ether [197–202] or cryptand [203–205] complexes with paraquat have been described. Such complexes are not within the scope of this review and the interested reader is referred to the literature cited above.

#### 2.5. Crown ether ammonium ion receptors with appended binding sites for other functionalities than ammonium

**Figure 35:**
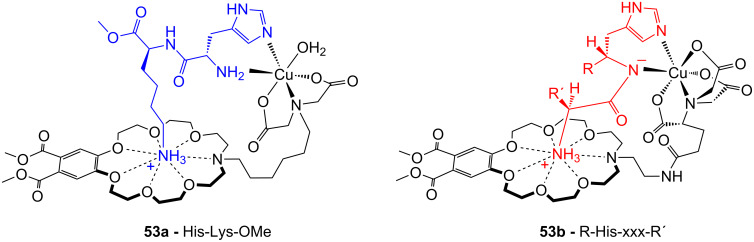
Copper imido diacetic acid azacrown receptor **53a** and the suggested His-Lys binding motif; a copper imido triacetic acid azacrown receptor **53b** and the target binding area (R = COO^−^, CONHCH_2_COO^−^, CONHCH_2_COOCH_3_, CONHCH_2_CONHCH_2_CONH_2_; R′ = H, CH_3_,CH_2_-CH(CH_3_)_2_, CH_2_CH_2_CONH_2_).

Crown ether receptors with appended moieties for the binding of different functionalities in addition to the ammonium ion have been reported. The combination of the luminescent ammonium-binding crown ether (**49a**) with a pendant copper imido diacetic acid complex (Figure 35) with an imidazole-co-ordinating site led to receptor **53a**, which co-ordinates peptides bearing both functional groups with high affinity in buffered aqueous solution [206]. An increase in emission intensity, visible to the naked eye, signals the guest binding: The response is triggered by the ammonium ion binding to the crown ether unit, which is in water only possible intramolecularily within the assembly. Compound **53** does not respond to the presence of an ammonium group, even in large excess. In the case of His-Lys-OMe a 1:1 complex with a molar binding constant of log *K*_ass_ = 4.2 is observed. The receptor was applied for the selective detection of small peptides containing *N*-terminal histidine or histidine (**81e**) among all other natural α-amino acids at physiological conditions.

In succession, the combination of a copper(II)-NTA complex with the benzocrown ether led to a receptor (**53b**) (Figure 35) that preferably binds to specific histidine-glycine peptide sequences under physiological conditions [207]. Nearly micromolar affinities were observed for Gly-Gly-His (log *K*_ass_ = 5.8) and Gly-His-Gly (log *K*_ass_ = 5.8) by emission titration in HEPES-buffered (pH 7.5) aqueous solution. In tetrapeptides, the recognition motif R′-xxx-HGG was identified, in which the *N*-terminal amino acid residue may vary (R′-xxx = Leu, Ala, Gly, Gln). Only the *N*-terminal amino group triggered an emission signal; the ammonium moiety of a lysine side chain did not.

Besides metal complexes, which will be discussed in detail in a later chapter, urea, thiourea and charged binding sites such as quaternary ammonium ions or guanidines are often employed as second anchoring functionalities for amino acids.

**Figure 36:**
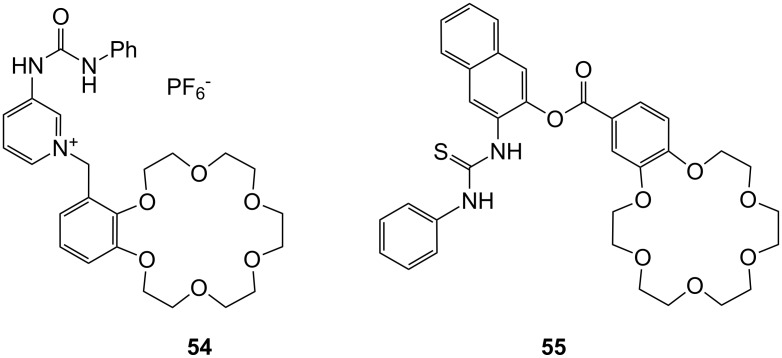
Urea (**54**) and thiourea (**55**) benzo crown receptor for transport and extraction of amino acids.

Receptor **54** binds to zwitterionic amino acids via a combination of urea-carboxylate and crown ether-ammonium hydrogen bonding (Figure 36), and thus efficiently transports them across a CHCl_3_ liquid membrane [208]. The binding properties of **54** were also examined by solid-liquid and liquid-liquid extraction experiments. The amounts of amino acids extracted into the chloroform phase were determined by the ^1^H NMR. In comparison to similar compounds devoid of one of these functional groups, receptor **54** efficiently extracted amino acids with non-polar side chains such as Phe, Ile, Leu, and Trp into CHCl_3_. The overall transport efficiencies (Phe > Trp > Ile > Leu > Val >> Ala > Ser >> Asp, His) were consistent with the extraction results (Phe > Ile > Leu > Val > Ala >> Ser, Asp, His, Tyr). No preference for aromatic amino acids over aliphatic amino acids was observed in extraction and transport experiments; no binding constants were however, reported.

A recent example by Costero et al. employed a comparable heteroditopic ligand in the solid-liquid extraction of ω-amino acids into DMSO solutions (Figure 36). The prepared ligand contained thiourea or amide groups for anion recognition [209]. Compound **55** was found to be an efficient solid-liquid extractant for lysine (**81c**) as well as 4-aminobutanoic, 5-aminopentanoic and 6-aminohexanoic acids, with the highest value recorded for 4-aminobutanoic acid (GABA). The simultaneous complexation of the anionic and cationic moieties by the ligand gave rise to extraction values much higher than those obtained with equimolar mixtures of the corresponding monotopic ligands. The introduction of a *para*-nitro group in the phenylthiourea made the extraction process much faster.

**Figure 37:**
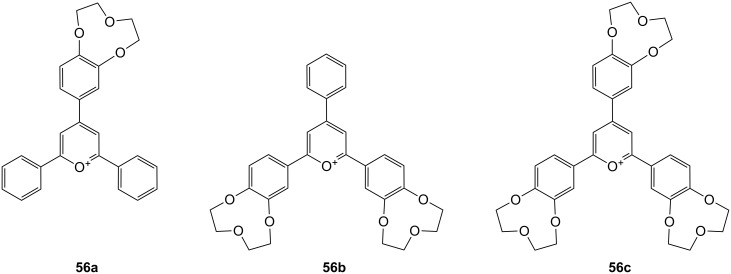
Crown pyryliums ion receptors **56** for amino acids.

The molecular recognition of *S*-amino acids such as asparagine, glutamine, lysine (**81c**) and arginine (**81d**) with crown pyrylium ions **56a** to **56c** (Figure 37) as receptors was examined by Moghimi et al. [210–211]. Their receptors use a two point binding of the guest: Ion pairing for the two oppositely charged carboxylate anion and pyrylium cation, and hydrogen bonding between crown ethers and the amino acid terminal NH’s. The terminal NH_2_ to COOH distance of *S*-asparagine is best matched when the crowns are located in the *ortho*-position of the receptors, **56b** (*K*_ass_ = 1290 ± 60 M^−1^) and **56c** (*K*_ass_ = 1740 ± 90 M^−1^). The distance in *S*-asparagine and *S*-glutamine is not long enough for interaction with **56a**. The binding properties were evaluated by fluorimetric titration in methanol.

**Figure 38:**
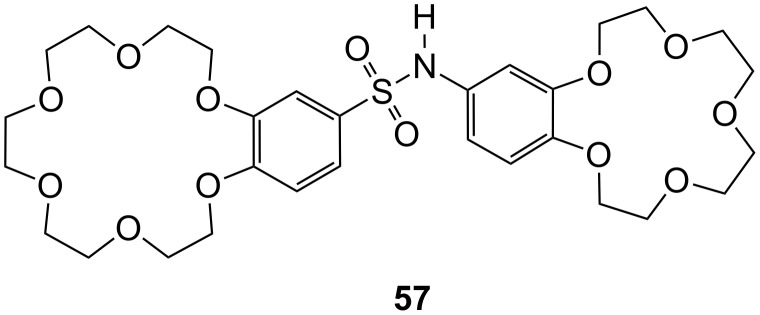
Ditopic sulfonamide bridged crown ether receptor **57**.

A different receptor type **57** for zwitterionic amino acids was described by Barboiu et al. [212]. Simultaneous complexation of the ammonium moiety of the amino acid by the benzo-18-crown-6 cavity and of the sodium ion in the benzo-15-crown-5 cavity (Figure 38) induces charge interactions of the carboxylate moiety with Na^+^-15-crown-5 and π–π-stacking interactions between the aromatic ring of phenylalanine (**81a**) and the aromatic moieties of **57**. The membrane transport mechanism of phenylalanine (**81a**) through a bulk liquid membrane was achieved and monitored as a function of the co-transported alkali cation.

**Figure 39:**
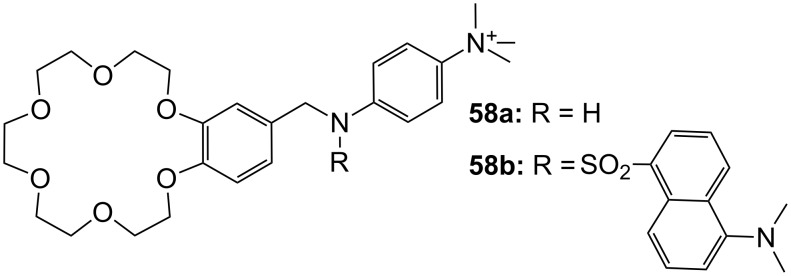
Luminescent peptide receptor **58**.

Schneider and Hossain [213] investigated the crown ether **58** (structurally related to the Voyer compound **47**) for peptide binding in water (Figure 39). Here, a peralkylated ammonium group interacts with the peptides carboxylate, whilst the primary ammonium ion is bound by the benzo crown ether. The bridging amine can be functionalized by a luminescent dansyl group as in **58b** to allow facile optical detection of the binding event and supplies additional hydrophobic interactions to aromatic peptide side chains. Several di- and tripeptides were tested with compound **58a**: Triglycine showed the highest binding affinity in water (*K*_ass_ = 200 M^−1^) and methanol (*K*_ass_ = 13000 M^−1^) as determined by NMR titration. Fluorescence titrations with **58b** revealed the effect of hydrophobic or π–stacking interactions of the dansyl group. Tripeptides bearing an amino acid with aromatic side chain functionality, such as Trp, showed a significant increased affinity (*K*_ass_ = 2150 M^−1^ for Gly-Trp-Gly) to **58b** in water compared to triglycine (*K*_ass_ = 210 M^−1^).

**Figure 40:**
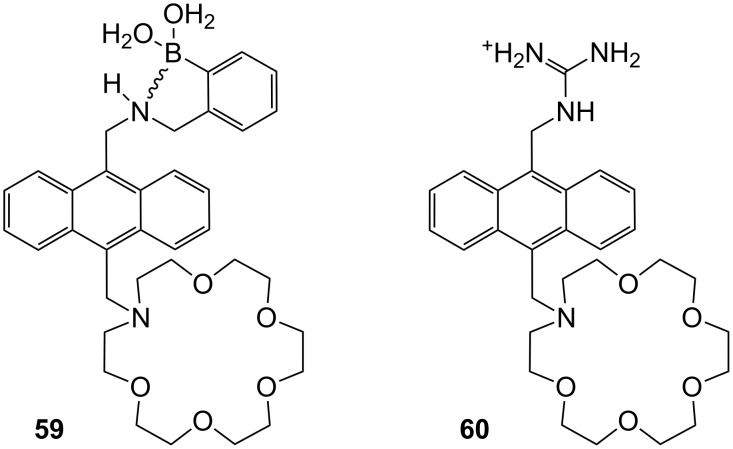
Luminescent receptor **59** for the detection of D-glucosamine hydrochloride in water/ethanol and luminescent receptor **60** for ω-amino acids.

Cooper and James prepared mono-aza-18-crown-6 ether **59** with a boronic acid binding site [214] (Figure 40). The additional interaction of boronic acid has been used to create a photoinduced electron transfer (PET) sensory system for saccharides. Binding studies were carried out in 33.2% (w/w) ethanol–water buffer, showing selective fluorescent enhancement with D-glucosamine hydrochloride (log *K*_ass_ = 3.31) at pH 7.18. In this medium, compound **59** showed no increase with D-glucose. For a fluorescent output both a diol and the ammonium group must be present in the guest. The increase in stability can be attributed to co-operative binding by the boronic acid and azacrown ether.

Guanidines are well known binders for oxoanions such as carboxylates [215]. A molecule similar to **59** was introduced for the recognition of amino acids by de Silva et al. [216] (Figure 40): Chemosensor **60** is capable of recognizing the distance between the two functional groups in methanol/water (3:2) at pH 9.5. Co-ordination of the carboxyl group to the guanidinium moiety of the receptor has a strong effect on the fluorescence output of the system. As in the former example, upon binding of the ammonium functionality in the crown ether the quenching by the PET of the nitrogen atom’s free electron pair disappears and an enhancement in the fluorescence of the anthracene is observed. 5-Aminopentanoic acid binds with *K*_ass_ = 84 M^−1^, while 3-aminopropanoic acid binds with only *K*_ass_ = 17 M^−1^. A limitation of the compound is its similar response to simple amines, e.g. propylamine (*K*_ass_ = 79 M^−1^).

**Figure 41:**
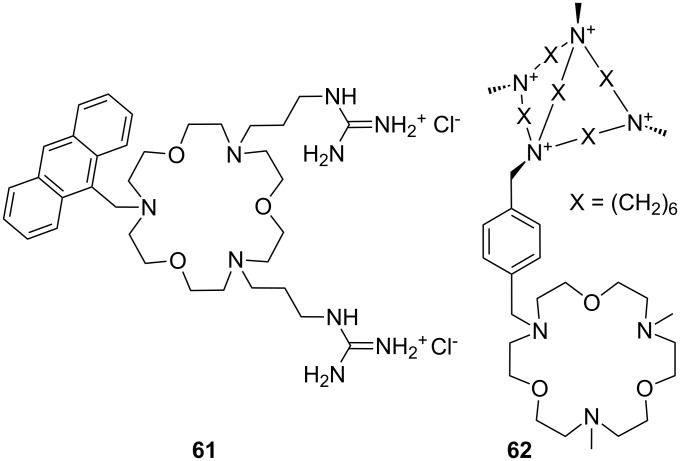
Guanidinium azacrown receptor **61** for simple amino acids and ditopic receptor **62** with crown ether and polyammonium macrocycle for GABA binding.

Suzuki et al. employed a similar approach for sensing amino acids in receptor **61**, which is based on tri-aza-18-crown-6 [217] (Figure 41). The ammonium-ion binding crown ether is substituted by two guanidinium groups interacting with carboxylates, and the luminescent anthracene moiety. Upon ammonium ion binding the quenching of the anthracene emission by PET is intercepted leading to an emission increase. The authors did not report binding constants, but described glycine, lysine (**81c**) and GABA (γ-aminobutyric acid) as preferred guests. The emission intensity increased upon addition of GABA to compound **61** in methanol/water 1:2 by a factor of 2.2.

A ditopic receptor **62** for the effective binding of zwitterionic GABA (γ-aminobutyric acid) was investigated by Schmidtchen [218] who combined triaza-18-crown-6 with a positively charged polyammonium macrocycle for the construction of the synthetic receptor (Figure 41).

**Figure 42:**
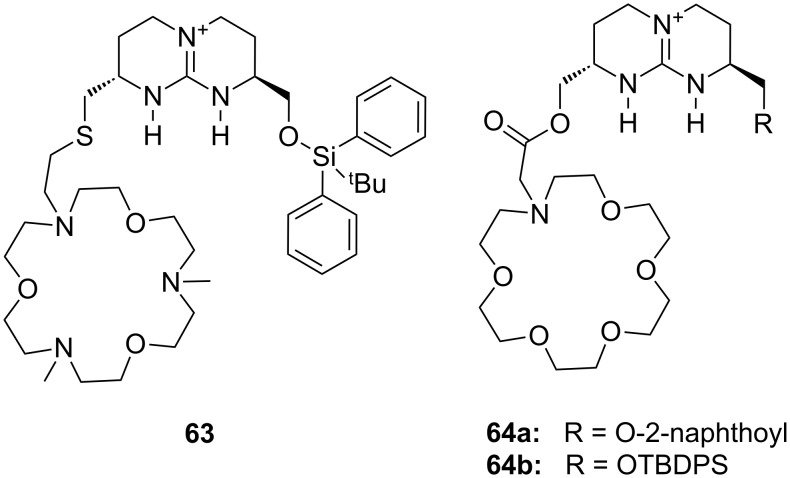
Chiral bicyclic guanidinium azacrown receptor **63** and similar receptor **64** for the enantioselective transport of simple amino acids into organic phases.

The same group described synthetic receptor **63** with bicyclic guanidinium and azacrown ether binding sites for amino acid zwitterions [219] (Figure 42). The chiral bicyclic guanidinium salt acts as strong anchor for the carboxylate and the triaza-crown ether binds the ammonium ion. The hydrophobic silyl ether provides additional interactions and facilitates the transfer of hydrophilic amino acid zwitterions into an organic phase. Quantification of the extraction process by radiometry revealed a 1:1 stoichiometry and suggests the zwitterion as the species undergoing phase transfer. Small hydrophilic (Ser, Gly), but no charged amino acids were extracted. Some enantioselectivity was observed in the transfer of phenylalanine (**81a**, 40% *ee*). In the case of **63** the order of decreasing extractability was Phe > Leu > Trp > Gly, Ser.

Comparable artificial carriers based on this bicyclic chiral guanidinium scaffold (Figure 42) attached to crown ethers (**64**) or lasalocid A were able to reach up to 80% enantiomeric excess in transport experiments for the separation mixtures of amino acid enantiomers under neutral conditions. Such chiral selectors for underivatized amino acids have been prepared, usually as the (*S*,*S*)-compounds, and evaluated by de Mendoza et al. [220]. Crown ethers were shown to be superior to lasalocid derivatives and amides were found to be better carriers than esters, though less enantioselective for transport across the bulk model membranes. Receptor **64a** proved to be the best “chiral selector”, followed by **64b**.

**Figure 43:**
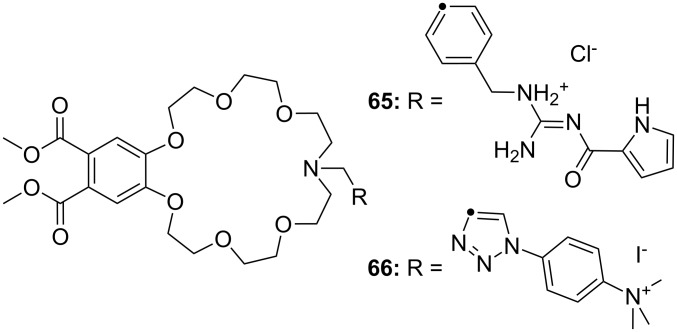
Receptors for zwitterionic species based on luminescent CEAAs.

CEAA **65** with appended guanidinium ions or quaternary ammonium side chains (Figure 43), as in **66**, were tested for amino acid recognition in aqueous methanol [221]. By following the binding events by fluorescence and UV–vis spectroscopy in methanol/water 9:1 (v/v), compound **65** showed selectivity for γ-aminobutyric acid (*K*_a_ = 1300 M^−1^) over ε-aminohexanoic acid, β-alanine and lysine (**81c**) at pH = 6.5. Compound **66** revealed a pronounced selectivity for (Gly)_3_ (*K*_a_ = 600 M^−1^) over (Gly)_2_, γ-aminobutyric acid and ε-aminohexanoic acid at pH 7.4. A 1:1 stoichiometry was always observed. Both receptors did not bind other amino acids.

The last examples presented in this chapter combine crown ether ammonium recognition with moieties for co-ordination or inclusion of non-polar side chains. Extended π-systems such as porphyrins, developing hydrophobic or stacking interactions, or carbohydrates and cyclodextrins, binding alkyl- and aryl chains by hydrophobic or van-der-Waals interactions, are discussed.

Cyclodextrins (**136**) [222–223], cyclic oligosaccharides of six (α), seven (β) or eight (γ) α-1→4 linked D-glucose units, can include non-polar guests such as alkyl chains or aromatic moieties in their hydrophobic interior mainly by van-der-Waals and hydrophobic interactions. Entropic effects play an important role: Complex formation leads to the release of high-energy water molecules from the cavity of cyclodextrins and is therefore entropically favorable. The selectivity depends principally on the steric fit, similar to the crown ethers.

**Figure 44:**
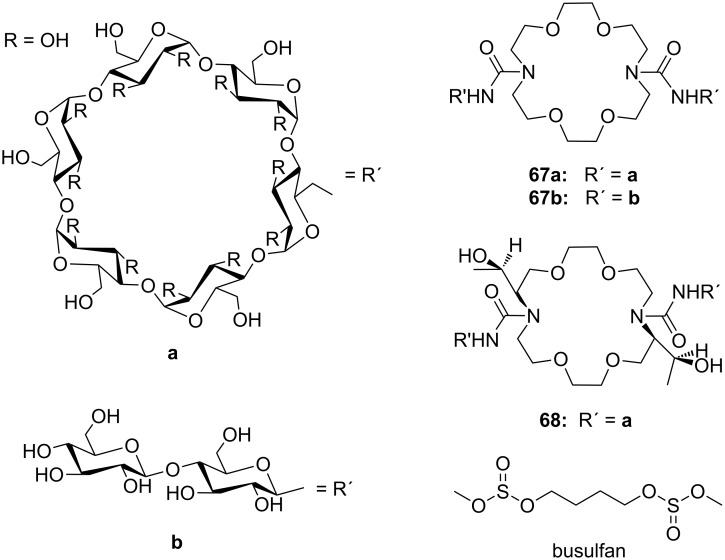
1,10-Azacrown ethers with sugar podand arms and the anticancer agent busulfan.

Combinations of a diaza-18-crown-6-ether with α-cyclodextrin- (**67a**, **68**) and celobiosyl- (**67b**) residues (Figure 44) bind efficiently *S*-arginine (**81d**), *S*-lysine (**81c**) and the anticancer agent busulfan [224]. The Job’s plots indicate 1:1 stoichiometries in all the complexes. Complexation constants (*K*_ass_) of ca. 4000 M^−1^ were estimated for [*S*-arginine/**68**], 5500 M^−1^ for [*S*-lysine/**68**], and 6000 M^−1^ for [*S*-arginine/**67b**] and 4500 M^−1^ for the [*S*-lysine/**67b**]. No significant differences between *S* and *R* series could be observed. Busulfan bound to all three ligands with the highest association constant of 1600 M^−1^ for **68** [225]. 2D NMR results clearly established that a similar mode of complexation is involved for both the amino acids and the anticancer agent: They are not embedded in the cyclodextrins cavity, but hydrogen bonded across the azacrown macrocycle to the urea functions.

**Figure 45:**
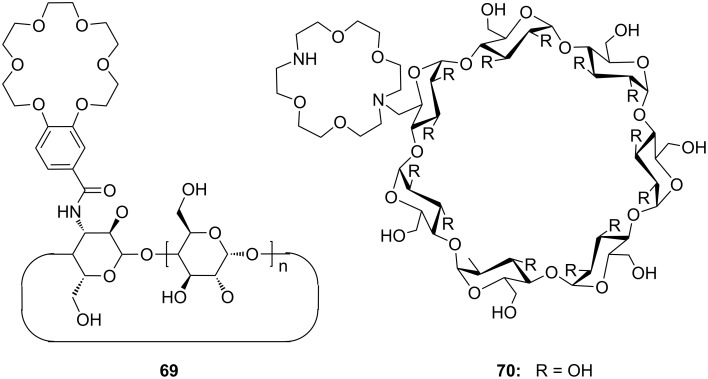
Benzo-18-crown-6 modified β-cyclodextrin **69** and β-cyclodextrin functionalized with diaza-18-crown-6 at primary face (**70**).

Another combination of crown ethers and sugars as ditopic receptors was described by Suzuki et al. who used a β-cyclodextrin derivative modified with benzo-18-crown-6 moiety (Figure 45) for the recognition of tryptophan (**81b**) in zwitterionic form in water [226]. The molecular recognition ability of **69** was improved by the co-operation of hydrophobic binding by the cyclodextrin cavity and the ammonium cation binding by the benzocrown moiety (188 M^−1^ vs. 31 M^−1^ for single side interaction). 2D ROESY experiments confirmed that the ammonium cation of Trp is located at the secondary hydroxy side of the cyclodextrin cavity and is recognized by the benzo-18-crown-6 moiety.

The association constant of ammonium ions with 18-crown-6 was reported to be 10–17 M^−1^ in water [106]. The β-cyclodextrin **70** functionalized with diaza-18-crown-6 at its primary face (Figure 45) showed a 7–10 fold enhanced binding affinity for aromatic ammonium ions in aqueous media compared to unmodified β-cyclodextrin [227]. Compared to **69**, this receptor reveals a binding constant in the same order of magnitude for an aromatic amine guest e.g. Trp. The point of attachment of the crown ether does not significantly alter the ammonium binding ability.

**Figure 46:**
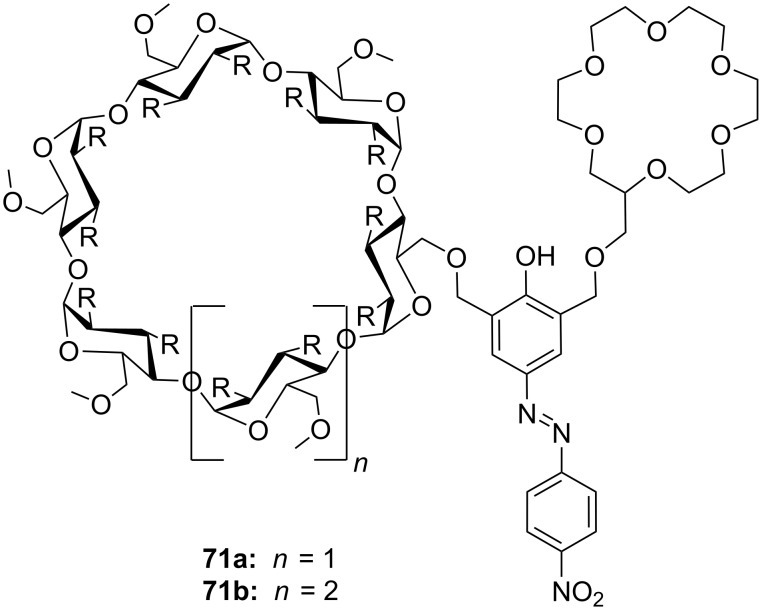
Receptors for colorimetric detection of primary and secondary ammonium ions.

A crown-appended permethylated α-cyclodextrin azophenol **71a** (Figure 46) showed a significant, distinguishable color change, observable with the naked eye, for primary and secondary amines. No change was evident in the case of tertiary amines, which is a similar analytic distinction as in the Hinsberg test [228]. The system was investigated by UV–vis spectrophotometry in chloroform. Association constants with primary amines were found to range from log *K*_ass_ = 4.2 to 4.8 and from log *K*_ass_ = 2.0 to 2.3 for secondary amines. The selective complexation is explained by H-bonding between the ammonium ion and oxygen atoms of the 18-crown-6 [229]. The hydrophobic interaction between the cyclodextrin and the lipophilic tail of the amine in combination with the acidity of the host molecule (p*K*_a_ = 5.6) assist the binding.

The studies were expanded by the related 18-crown-6 azophenol dye with permethylated β-cyclodextrin **71b** [230] (Figure 46). The binding of various amines was investigated by UV–vis spectrophotometry in chloroform. As before, the addition of primary and secondary amines shifted the absorbance maximum differently, from 380 nm (yellow) to 580 nm (violet) and 530 nm (pink), respectively with no change observable with tertiary amines. The log *K*_ass_ values are, compared to compound **71a**, generally 5 to 10% higher (4.25–4.95 for primary, 2.10–2.48 for secondary amines). The selectivity was calculated to be 60–720. Receptors which lack the crown ether moiety, changed from yellow (380 nm) to pink (500 nm) upon addition of amines, but with no selectivity and binding constants being one order of magnitude lower. NMR spectroscopy indicated the formation of 1:1 complexes and the inclusion of the alkyl chain in the cyclodextrin by a strong shift of the CH_2_-protons. In a competition experiment, *n*-propylamine was added to the chloroform solution of **71b** containing 2000 equiv of triethylamine. A small amount of *n*-propylamine was already known to result in a marked increase in absorption intensity, whilst in the case of the tertiary amine no spectral changes were observed.

The formation of efficient H-bond interactions of the ammonium ion to the oxygen atoms of the crown ether and their number, the hydrophobic interaction between the cyclodextrins and the lipophilic tail of the amine as well as the acidity of the host molecule determine the selectivity and binding strengths of these ditopic receptors.

The following examples involve crown ether–porphyrin conjugates. In these examples the ammonium ion binding takes place at the crown ether moiety. Ammonium ion binding using porphyrin based binding sites will be discussed later in this survey.

**Figure 47:**
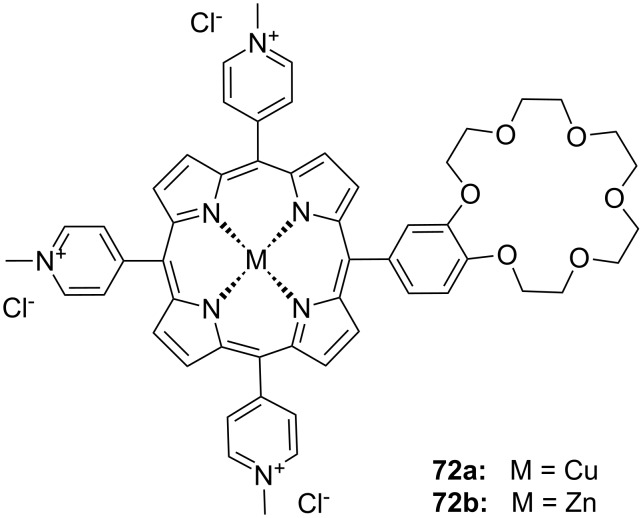
Porphyrine-crown-receptors **72**.

Schneider et al. described a water-soluble host compound with three pyridinium units and one spacer-connected benzocrown ether unit in the meso-positions of porphyrin and its Zn(II) or Cu(II) complexes [231] (Figure 47). They investigated the complexation constants of unprotected di-, tri- and tetrapeptides with the metal-free and the metalated hosts in water. Metalation led to small changes of the selectivities towards different peptides compared to the apo-derivative, with complexation constants in water of 10^5^ M^−1^ to 10^6^ M^−1^. One complex containing the tripeptide Gly-Gly-Phe was analyzed in detail by COSY, HSQC, HMBC, and NOESY NMR experiments and clearly indicated complexation of the ammonium ion in the crown and π–π-stacking interactions of the phenyl of Phe with the porphyrin. Peptides containing aromatic side chains were always bound better than the corresponding simple oligo-glycines. The titration curves showed isosbestic points, in line with the expected 1:1 complexes, which were supported by very good nonlinear least-squares fits to a 1:1 model.

**Figure 48:**
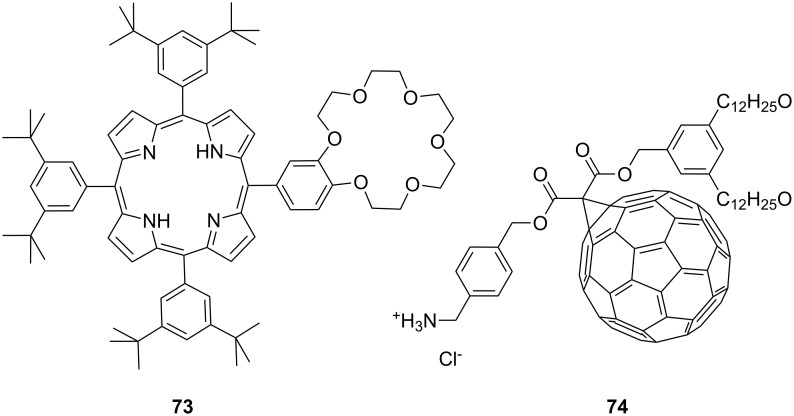
Porphyrin-crown ether conjugate **73** and fullerene-ammonium ion guest **74**.

Nierengarten et al. investigated the ability of a methanofullerene derivative with an ammonium subunit to form an aggregate with a porphyrin–crown ether conjugate (Figure 48) by NMR, UV–vis, electrospray mass spectrometry and luminescence experiments [232]. In addition to the ammonium–crown ether recognition, they found intramolecular stacking of the fullerene moiety to the porphyrin subunit. Due to this additional recognition element, the association constant for the aggregate was increased by two orders of magnitude when compared to the *K*_ass_ values found for the complexation of **74** with the crown ether (2100 M^−1^ in CDCl_3_). The value is consistent with association constants reported for associates resulting from ammonium–crown ether interactions [233].

The broad variability of crown ethers allows manifold adaption for specific tasks: A variety of crown ether receptors for co-operative recognition of ammonium moieties in diamines, for transport and effective enantioselective recognition of amino acids, as esters or in zwitterionic form have been described. Crown ethers have been widely used for the recognition of primary organoammonium compounds as found in amino acids, neurotransmitters such as GABA and other biological important molecules like dopamine (**2**).

### 3. Calixarenes, resorcinarenes and cavitands

Calixarenes are versatile host molecules for ammonium ions with unique structure and complexation properties. In this chapter we discuss approaches for ammonium ion recognition with calixarenes and related molecules. We will start our survey with simpler substitution patterns and proceed with more complex substituted calixarenes for enantiodiscrimination, for colorimetric assays and capped structures. Resorcinarenes and deeper cavities, ditopic receptors, and capsules are also included.

#### 3.1. Basic examples with simpler substitution pattern

**Figure 49:**
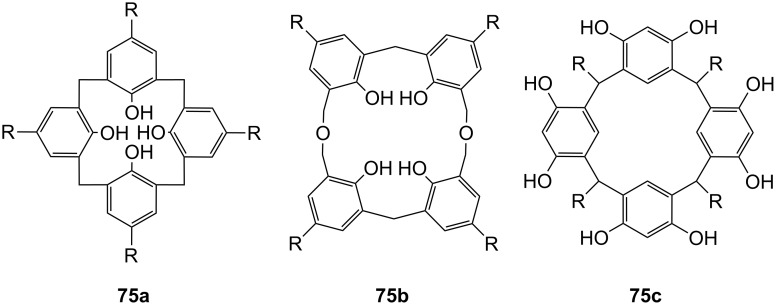
Calix[4]arene (**75a**), homooxocalix[4]arene (**75b**) and resorcin[4]arene (**75c**) compared (R = H, alkyl chain).

Calixarenes and resorcinarenes (**75)** (Figure 49) belong to the most versatile building blocks in supramolecular chemistry. Several books and reviews covering their synthesis, structural properties and applications have been published [234–236]. A variety of methods for the synthesis and functionalization of the macrocycles has been developed [237–238]. Likewise, the synthesis and application of resorcinarenes and O-alkylated derivatives have been comprehensively summarized [239]. Calixarenes, e.g., **75a** resemble a vase like (chalice) shape but are not completely rigid. They may form many conformational isomers by the rotation of the phenol units through the annulus, thus affording a large number of unique cavities of different size and shape. Homooxocalix[4]arenes (example **75b**, Figure 49) and their methyl esters are more recently studied examples [240]. Together with the structurally related resorcin[*n*]arenes (example **75c**) and calixpyrroles, calixarenes are used in a variety of applications, such as chromo- and fluorophores [241–242] for metal ion binding in solution [243–244], anion complexation [245–248] and binding of neutral guests [249], as potentiometric sensors [250–252] in ion selective electrodes [253–255] or as molecular switches [256]. The aromatic cavity of calixarenes is an excellent model for the investigation of cation–π-interactions [11,257–259].

**Figure 50:**
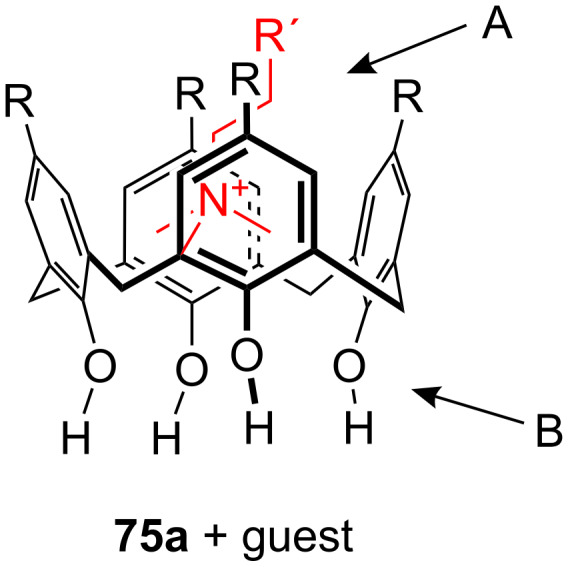
Calix[4]arene and ammonium ion guest (R = H, alkyl, OAcyl etc.), possible binding sites; A: co-ordination of cationic or neutral guests (cation–π-interaction), B: binding site for cationic guests (ion–dipole-interaction or H-bonding).

A calix[4]arene includes ammonium ions in its pre-organized cone cavity via electrostatic attraction between the positive charge of the guest and the electron rich faces of the aromatic rings (“cation–π-interaction”) (Figure 50) [260–262]. The inclusion of alkyl ammonium ions in the cavity of calixarenes is therefore reflected in a high field shift of the host signals in the ^1^H NMR spectrum. Based on the magnitude of the shifts of the different host signals, conclusions can be drawn on the preferred orientation of the guest in the cavity [263].

Gutsche et al. reported the complexation of aliphatic amines by alkylcalix[4]arene with a binding strength in the order of 10^4^ M^−1^ in acetonitrile [264–265]. The contribution of cation–π-interactions to the binding was demonstrated for several examples of complexes with quaternary ammonium [266–267] or tetraalkylammonium [261,268–269] salts in organic media. Proton transfer from OH-groups of the calixarene to the amine, followed by association and inclusion is a different binding situation: The guest is co-ordinated by a tripodal H-bonding [265–266,270–271]. The complexation behavior seems to be mainly determined by the conformational mobility of the calix. Control of the conformational properties of these macrocycles is crucial for their applications in supramolecular chemistry.

**Figure 51:**
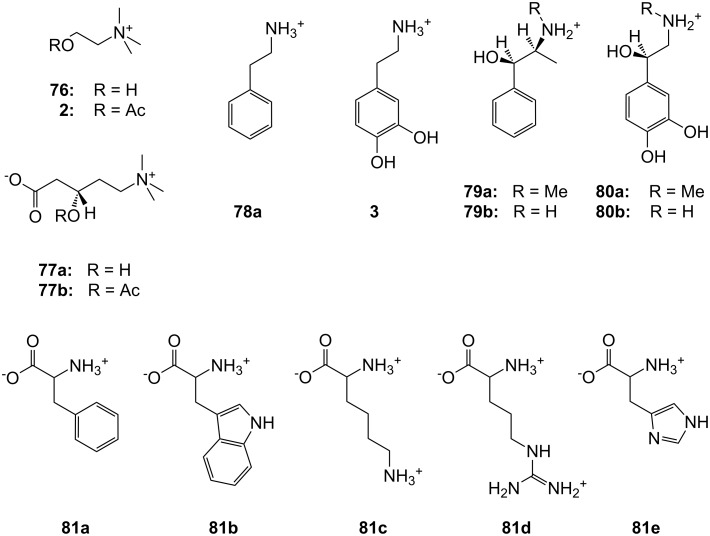
Typical guests for studies with calixarenes and related molecules.

Typical guests (Figure 51) in studies with calixarenes and resorcinarenes utilizing the explained modes of interaction are the physiologically relevant quaternary ammonium compounds choline (**76**), acetylcholine (**3**), carnithine (**77a**) and acetyl-carnithine (**77b**), as well as the salts of the aromatic amines 2-phenethylamine (**78a**), dopamine (**2**), ephedrine (**79a**), norephedrine (**79b**), adrenaline (**80a**) and noradrenaline (**80b**).

Additionally, amino acids and their derivatives are also bound by calixarenes, especially aromatic amino acids such as phenylalanine (**81a**) or tryptophan (**81b**) or the basic representatives, for example, lysine (**81c**), arginine (**81d**) and histidine (**81e**) (Figure 51), and peptides containing these residues. Similar to larger crown ethers (24-crown-8 and larger) or cyclodextrins, calixarenes may also be threaded to form rotaxane like structures. A common guest for this is paraquat. The reader is referred to the literature covering this topic [272–275]. We discuss now some recent examples in ammonium ion recognition with the calixarene class of receptors and focus on the recognition of these ammonium targets e.g. *N*-terminal peptide recognition, preferably in water and/or under neutral conditions. The binding of metal ions is not covered and has been already reviewed [243]. For detailed thermodynamic data we recommend the articles of Izatt et al. [146] and Namor et al. [276]. Recognition of biochemical targets was recently covered comprehensively by Ludwig [277]. Biros and Rebek have summarized the application of water soluble resorcinarenes for the recognition of ammonium ions in their recent review [278].

**Figure 52:**
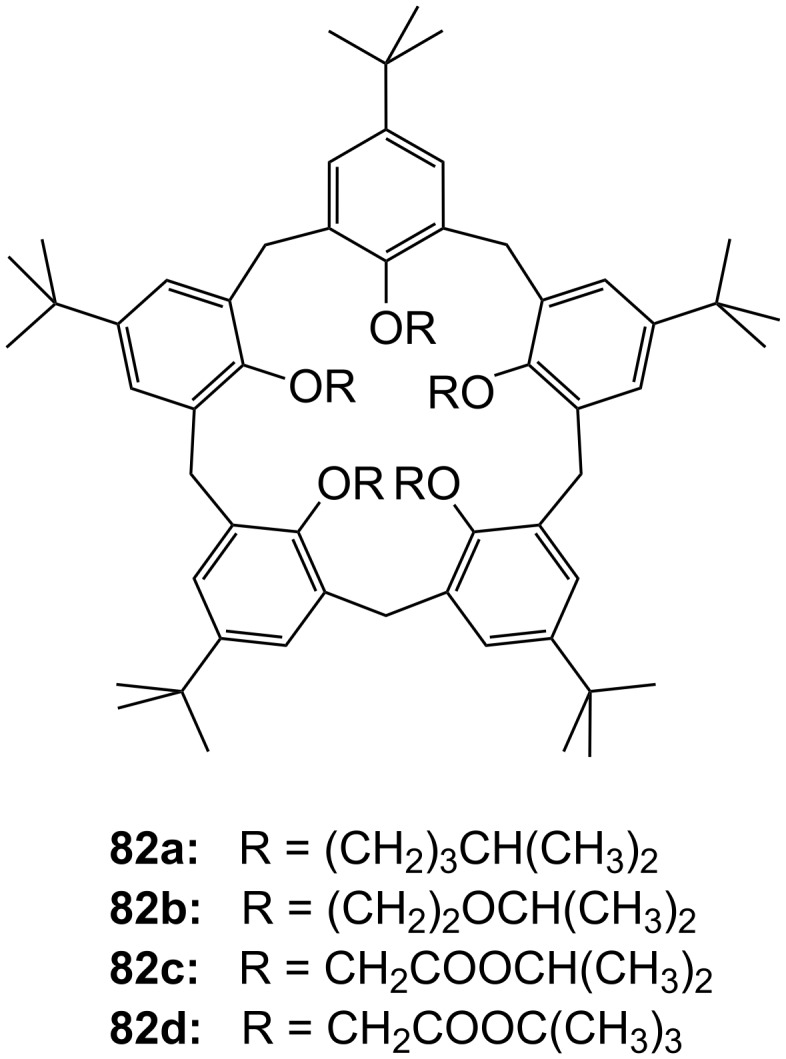
Lower rim modified *p*-*tert*-butylcalix[5]arenes **82**.

In the simplest case, only one side of the calixarene skeleton is substituted. For example, *p*-*tert*-butylcalix[5]arene **82** modified at the lower rim [279] (Figure 52) was investigated in CDCl_3_/CD_3_OD (9/1). The binding affinities of isomeric butylammonium picrate salts show high log *K*_ass_ values with the *n*-BuNH_3_^+^ ion ranging from 4.63 to 6.47, while other branched cations, such as *tert*-BuNH_3_^+^ give significantly lower values. The stability of the complexes generally decreased in the order: **82d** > **82a** > **82b** > **82c** for one given isomer, with the highest selectivity of calix[5]arenes **82a** and **82d** towards *n*-BuNH_3_^+^ ion. The presence of *tert*-butyl substituents on the upper rim is essential to force the molecule into a regular *C*_5v_ cone conformation and ensure selective inclusion of R-NH_3_^+^-ions. Receptors **82a** and **82d** formed 1:1 inclusion complexes only with Na-Ac-Lys-OMe hydrochloride and Lys-Gly-OMe dihydrochloride. In the latter the ε-butylenammonium group was recognized by the cavity and complexed in the presence of an unprotected α-ammonium group. The methyl ester hydrochlorides of the neurotransmitter γ-aminobutyric acid (GABA) and the related plasmin inhibitor ε-aminocaproic acid (ε-Ahx) [280] were also strongly included with degrees of complexation up to 80%.

**Figure 53:**
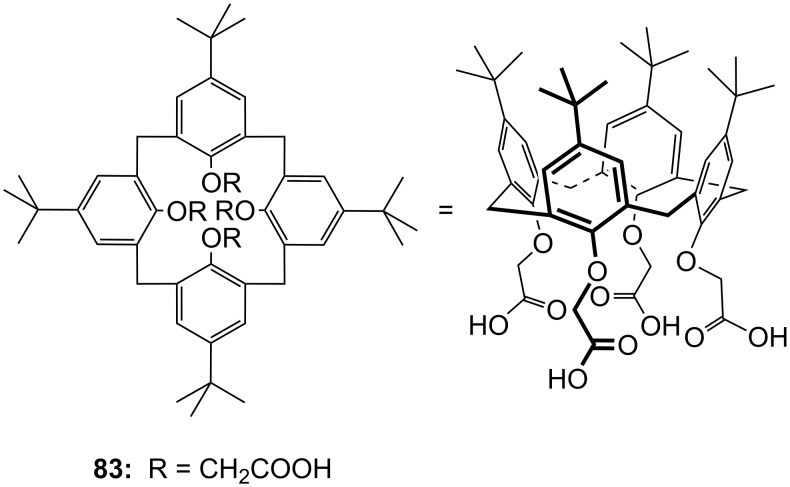
The first example of a water soluble calixarene.

Similar to the unsubstituted calixarenes such examples are only poorly soluble in water and polar substituents are required to increase water solubility. Several examples of water soluble calixarenes bearing phosphonate [281], amino acid [282] or neutral groups [283] at the upper rim have been reported already in the 1990s. Arduini et al. reported the first example of a water soluble calix[4]arene in the fixed cone conformation (Figure 53). It carries four carboxylate groups at the lower rim but shows no inclusion of neutral molecules in water [284].

**Figure 54:**
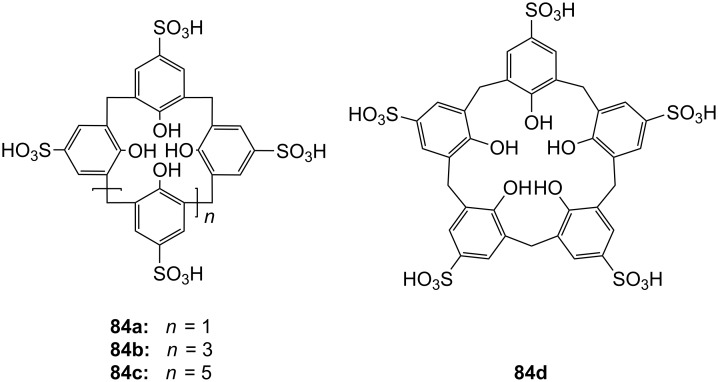
Sulfonated water soluble calix[*n*]arenes that bind ammonium ions.

Sulfonated calix[*n*]arenes (**84**, *n* = 4, 6, 8) [266] (Figure 54) have good water solubility. They complex trimethylanilinium cations (*K*_ass_ for *n* = 4 is 5600 M^−1^) and adamantlytrimethylammonium cations (*K*_ass_ for *n* = 4 is 21000 M^−1^) in water [285–286]. Studies by Gokel and Kaifer on the inclusion of ferrocene derivatives in water showed that calix[6]arene hexasulfonate (**84b**) is a good receptor for the complexation of a bulky trimethylammonium ion with a association constant of *K*_ass_ = 10930 M^−1^ [287].

Later, the investigated scope was expanded to the corresponding calix[5]arene (**84d**). The inclusion of tetramethylammonium and ditopic trimethylammonium cations was studied at neutral pH by ^1^H NMR and compared to the homologous tetrasulfonatocalix[4]arene (**84a**) [288]. The more flexible host exhibits a more efficient and selective complexation of ditopic methylammonium ions compared to the more pre-organized calix[4]arene receptors (**84a**). This is a rare case of molecular recognition by induced fit enhancing affinity and selectivity.

Utilizing the outstanding complexation properties of calixarenes for quaternary ammonium ions, the binding of acetylcholine (**3**) has attracted much interest due to its biological importance as a neurotransmitter. It has been shown, that the cationic ammonium group of acetylcholine (**3**) binds to the aromatic cavity of calixarenes through cation–π-interactions (see also later examples of **75c**, **115c**, **116, 117, 118** and **126a/c**).

**Figure 55:**
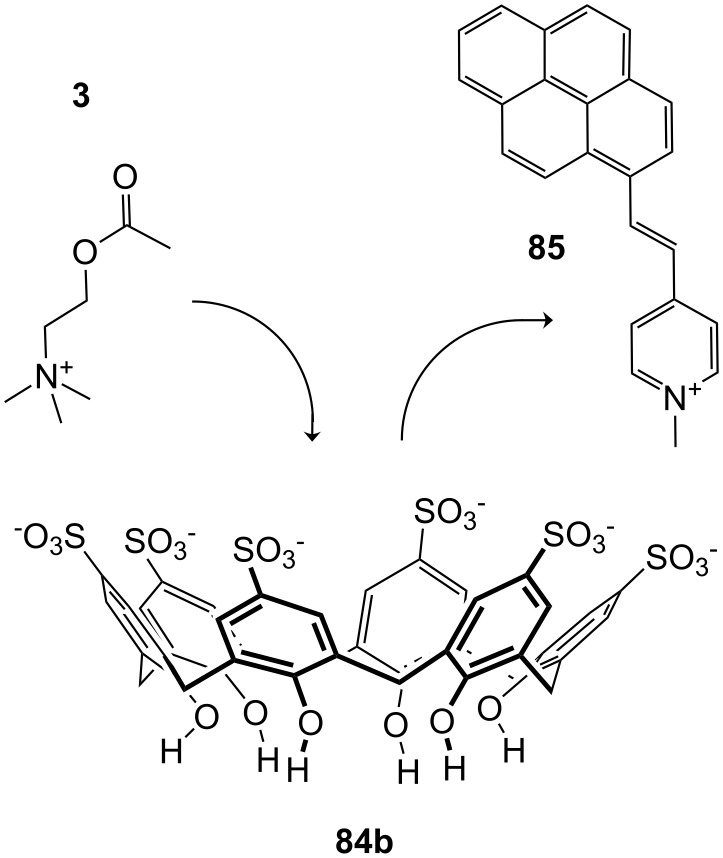
Displacement assay for acetylcholine (**3**) with a sulfonato-calix[6]arene (**84b**).

Compound **84b** was used to sense the presence of acetylcholine (**3**) in neutral aqueous or water/methanol solution. The sulfonatocalix[6]arene binds acetylcholine (**3**) in preference to primary and secondary amines, and allows the use of the pyrene indicator **85** in a displacement assay (Figure 55). Upon displacement of the fluorescent pyrene cation by **3**, the binding event is signalled by the increased fluorescence intensity of **85** in solution [289].

The affinity of the *p*-sulfonatocalix[*n*]arenes (**84**) (*n* = 4, 6, and 8) towards amino acids was also extensively investigated by ^1^H NMR [290–292], microcalorimetry [293] and HPLC-methods [294].

The *p*-sulfonatocalix[4]arenes formed 1:1 complexes more strongly with basic amino acids with *K*_ass_ values for Arg and Lys of 1520 and 740 M^−1^, respectively (phosphate buffer at pH 8), than with aliphatic or aromatic amino acids: Val, Leu, Phe, His, Trp, with *K*_ass_ values between 16 M^−1^ and 63 M^−1^ (phosphate buffer at pD 7.3) [292,295].

**Figure 56:**
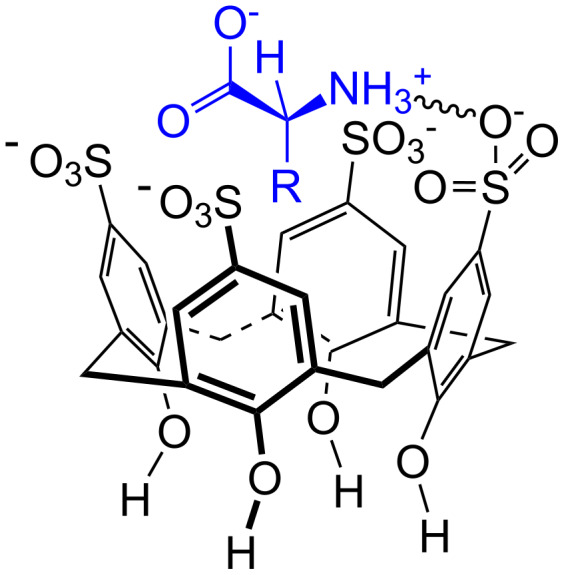
Amino acid inclusion in *p*-sulfonatocalix[4]arene (**84a**).

The basic amino acids arginine (**81d**) and lysine (**81c**) show strong electrostatic binding to calix[4]arene sulphonate at pH 5 (Figure 56). For higher calixarenes, only weak interactions at the faces of the flattened macrocycles occur. This binding is in contrast to the inhibition of protein–protein interactions by the calixarenes where the calix[6]arene and calix[8]arene sulfonates show much stronger effects [291].

Their application as glycosylaminoglycan (GAG) mimicry [296] was demonstrated by the binding thermodynamics towards certain di- and tripeptides bearing lysine (**81c**) or arginine residues in aqueous buffer at pH 8.0 [296]. Due to their key role in these peptide sequences present in GAG recognition sites, arginine (**81d**) and lysine (**81c**) were also used as guests in the titration microcalorimetry and NMR studies. The simple amino acids were bound with *K*_ass_ = 10^3^ dm^3^ mol^−1^. With the corresponding dipeptides there was a of 3 to 4 fold increase in binding, with the tripeptide of 5 to 8 fold increase was observed in comparison to Arg or Lys, respectively. More interaction sites were involved in their binding. Mixed Arg-Lys-peptides were bound more strongly and were sequence independent. The selectivity order (Arg > Lys > other amino acids) was retained in the peptides and was governed by hydrophobic interactions between the calixarene cavity and the aliphatic or aromatic guest moiety. The apolar part of the peptide inserts into the cavity.

**Figure 57:**
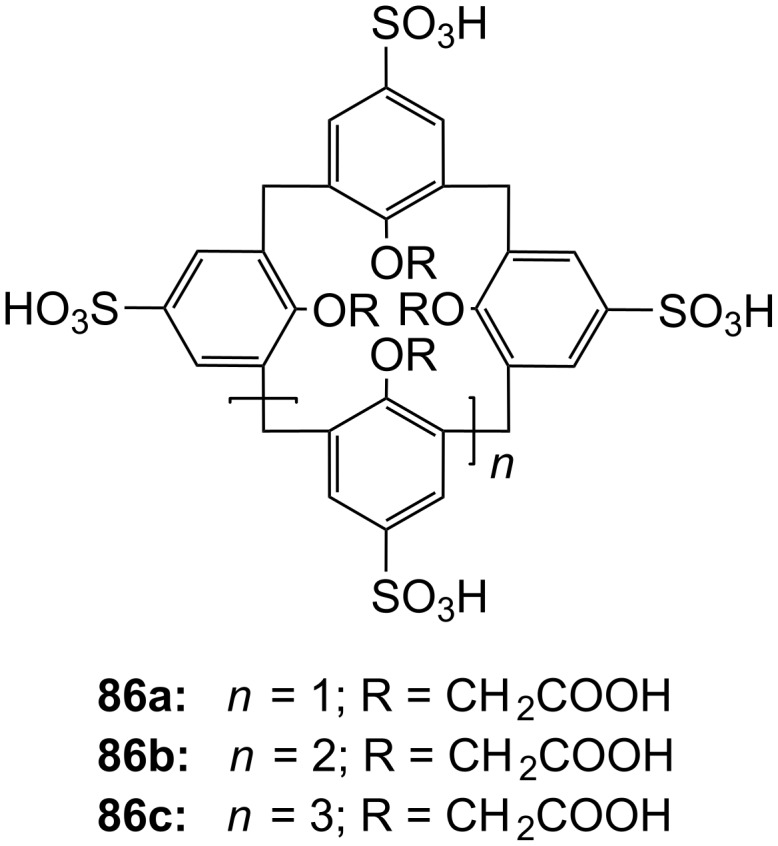
Calixarene receptor family **86** with upper and lower rim functionalization.

Ungaro et al. introduced sulfonate groups instead of the bulky *tert*-butyl groups in **83** [297], resulting in more flexible hosts [*n* = 1; X = H (**83**) and SO_3_H (**86a**); R = CH_2_COO^−^] (Figure 57). From compound **83** to **86a** a significant increase in log *K*_ass_ values for the binding of organic ammonium ions was observed: 1.7 and 3.3 for benzyl-NMe_3_^+^ or 1.7 and 3.4 for *p*-nitrobenzyl-NMe_3_^+^, respectively [298]. The inclusions were enthalpically driven and disfavored for entropy reasons.

**Figure 58:**
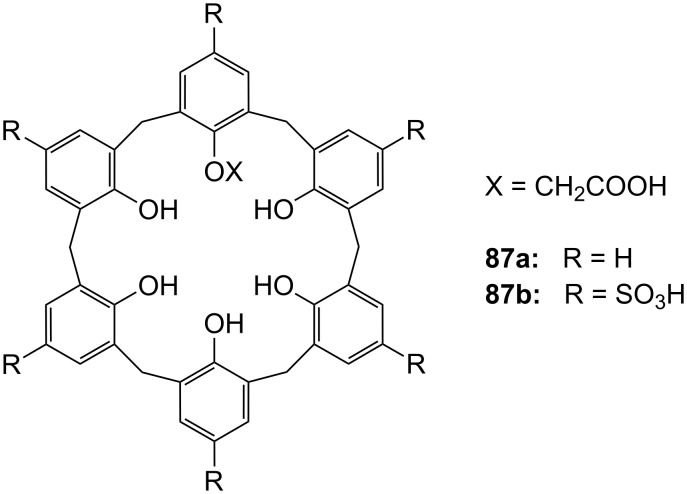
Calix[6]arenes **87** with one carboxylic acid functionality.

Calix[5]arenepentasulfonates (**86b**) bind trimethylammonium ions in water (pD 7.3) with association constants between 4.0 × 10^3^ and 1.3 × 10^5^ M^−1^. The alkylammonium group is completely immersed in the cavity [288]. The corresponding calix[6]arene (**86c**) binds a variety of amino acids in water. The highest binding affinities were found for aspartic acid, arginine (**81d**) and tryptophan (**81b**, *K*_ass_ = 4.1 × 10^3^ M^−1^, 3.6 × 10^3^ M^−1^ and 2.5 × 10^3^ M^−1^, respectively). Coleman et al. investigated a similar calix[6]arene with one carboxyl group at the lower rim (Figure 58) in amino acid recognition in water [299]. The selectivity changed in favor of asparagine (log *K*_ass_ = 3.82 for **87a** and 3.61 for **87b**). These most stable complexes resulted from the double H-bonding, which is known from carboxylate dimers. Similar contributions could be observed for arginine (**81d**) and lysine (**81c**). Additional π–π-interactions stabilized the complexes with aromatic amino acids; the hydroxy or thiol groups in cysteine and serine showed no effect on the complex stability. In summary, the 1:1 complex stability follows the following order: acidic > aromatic ~ basic > aliphatic ~ polar amino acids. The more polar compound **86b** binds non-polar guests weaker.

**Figure 59:**
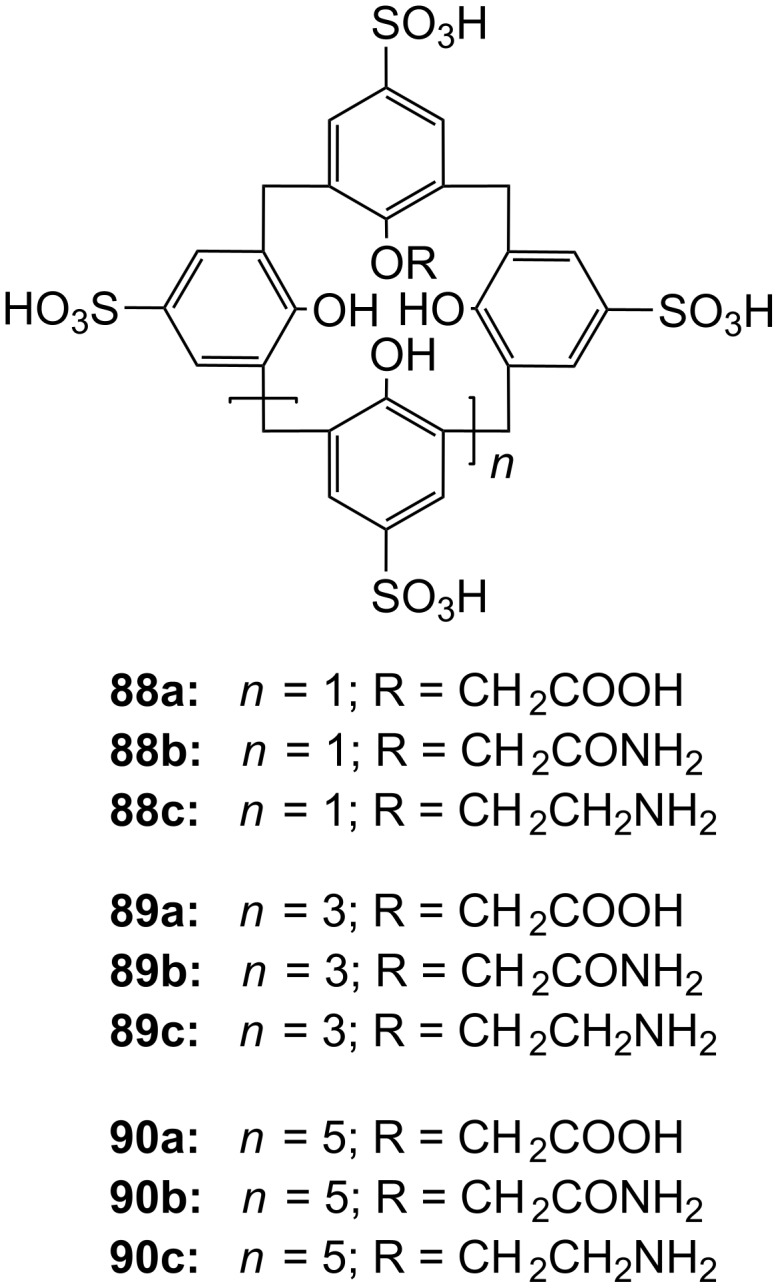
Sulfonated calix[*n*]arenes with mono-substitution at the lower rim systematically studied on their response to amino acids.

Consequently, da Silva and Coleman studied complexing properties of *p*-sulfonatocalix[*n*]arenes (*n* = 4, 6, 8) mono-functionalized at a phenolic oxygen (Figure 59) towards 11 amino acids by means of ^1^H NMR spectroscopy in unbuffered aqueous sodium hydroxide solution (pH 8.0) and compared them to the unsubstituted parent calixarenes [300]. In general, the receptors follow the trends discussed above: Arg and Lys, and sometimes His are bound more strongly than Gly, Ala, Leu, Pro, Phe and Trp. Receptors with acid functionality (**88a**, **89a** and **90a**) often show higher binding values for the basic amino acids. Especially noteworthy is the enhanced complexing ability for aspartic acid with *K*_ass_ values ranging from 2200 (**88b**) to 2500 M^−1^ (**90b**) for the amide functionalization, 2800 (**88a**) to 3200 M^−1^ (**90a**) for the acid functionality and, not surprisingly observing the highest values of 5600 M^−1^ (**88c**) to 5400 M^−1^ (**90c**) for the amine substitution pattern. Ser bound strongly to **88a** with *K*_ass_ = 3555 M^−1^ attributed to its additional hydrogen bonding site and the optimal fit.

**Figure 60:**
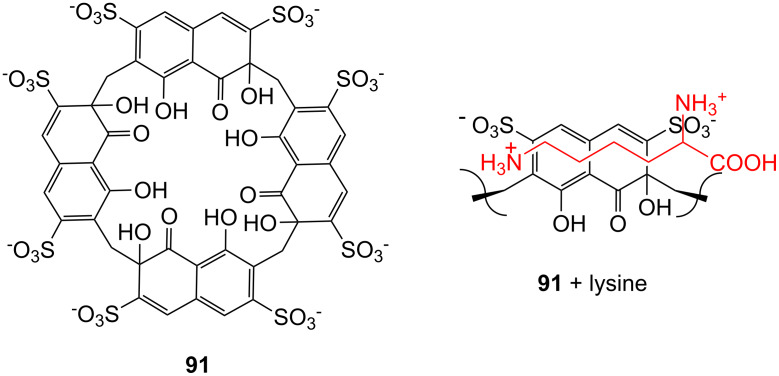
Cyclotetrachromotropylene host (**91**) and its binding to lysine (**81c**).

The formation of complexes between derivatized cyclotetrachromotropylene host (**91**) (Figure 60) and Ala, Asp and Lys in aqueous solution at pD 1.0 was also investigated [301]. For tetraalkylammonium ions, the hosts reveal the same stability trend as has been reported for the 1:1 complexes of *p*-sulfonatocalix[4]arene (**84a**). The *K*_ass_ values, reaching 2.7 × 10^4^ M^−1^ for the complexation of Et_4_N^+^ in D_2_O, are in the same order of magnitude as for **84a**. A similar behavior is observed for amino acids. The basic representative lysine (**81c**) is bound best in a 1:1 complex with the host with a *K*_ass_ value of 2.0 × 10^3^ M^−1^. The binding values for aspartic acid and alanine were substantially smaller (250 M^−1^ and 70 M^−1^, respectively).

The non-covalent phosphate–ammonium interaction not only plays a key role in living systems for many critical molecular recognition processes, it can also inspire the design of water-soluble artificial receptors.

**Figure 61:**
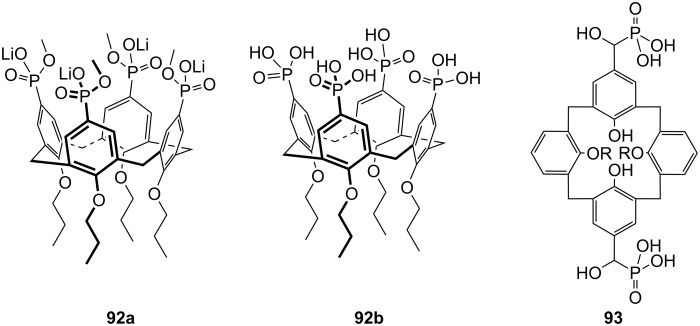
Calixarenes **92** and **93** with phosphonic acids groups.

The influence of phosphonic acids groups instead of sulfonate groups at the upper rim of calix[4]arenes has also been investigated. Witt et al. researched the complexation properties of water-soluble calix[4]arenes based cavitands (Figure 61) with (1*R,*2*S*)-(−)-ephedrine (**79a**), (1*R*,2*S*)-(−)-norephedrine (**79b**), (*R*)-(−)-noradrenaline hydrochloride (**80b**) and 2-phenylethylamine hydrochloride (**78a**) in phosphate buffer at pD 7.3 [302]. The host molecules were intended to mimic the adrenergic receptor. The participation of the calixarene hydrophobic cavity was confirmed and the structural requirements for the binding of the ammonium ion guests were investigated. The host compounds were able to form 1:1 complexes with an association constant *K*_ass_ of up to 145 M^−1^ (2-phenylethylamine hydrochloride (**78**)–(**92b**)). The aggregate stoichiometry was confirmed by a Job’s plot. For ammonium type guest, a stronger interaction is observed when phosphonic acids groups are attached at the upper rim (*K*_ass_ for **92b** > **92a**).

A similar receptor for amino acids was studied by Zielenkiewicz et al. who investigated the thermodynamics of distally substituted bis(dihydroxyphosphorylhydroxymethyl)calix[4]arene at the upper rim of racemic **93** (Figure 61) in the binding of several amino acids [303–304] and dipeptides [305] in methanol by isothermal titration calorimetry, NMR and UV–vis spectroscopy. Free amino acids as well as dipeptides gave strong 1:1 complexes. The complex stability correlates with the hydrophobicity of the amino acid residues and decreases with decreasing hydrophobicity: Ile > Leu > Val > Ala > Gly with log *K*_ass_ = 4.23 for Ile and 3.84 for Gly. Neutral aliphatic and aromatic amino acids were better bound than basic ones. The stability constants for dipeptides were in a similar range of 25000–45000 M^−1^, enthalpy changes in the range of −10.5 to −5.9 kJ mol^−1^ and −26.5 to −25.3 kJ mol^−1^ in the estimated Gibbs free energy, respectively. The complexation phenomenon was found to be driven by electrostatic interactions between the protonated *N*-terminal amino group of the guest and the calixarene phosphoryl groups.

Water soluble calix[4]arenes with one, two or four dihydroxyphosphoryl groups at the lower rim can form salts with (1*S*,2*R*)-(+)-ephedrine and 2-phenylethylamine hydrochloride [306]. The salts of these inherently chiral calixarene phosphoric acids with the chiral amines are easily separated into diastereomeric forms.

**Figure 62:**
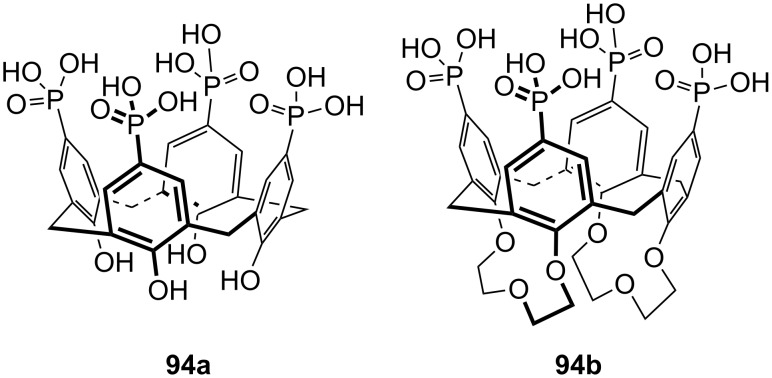
Calix[4]arene tetraphosphonic acid (**94a**) and a double bridged analogue (**94b**).

Based on the results of the former investigations, studies with **92b** were extended to amino acid derivatives and also compared to a series of calix[4]arene phosphonic acids [307]. The influence of the calixarenes conformation flexibility and its hydrophobic cavity shape dependent on the lower rim substitution pattern on the complexation process was monitored by ^1^H NMR spectroscopy in deuterated phosphate buffer at pD 7.3. Receptor **92b** did not show any remarkable selectivity towards the investigated amino acids methyl esters (*K*_ass_ = 10^2^ M^−1^). Only mixed 1:2 and 2:1 (host–guest) complexes were observed for compound **92b**. By contrast, compounds **94** (Figure 62) showed selectivity for basic amino acid methyl esters, i.e. Lys-OMe (*K*_ass_ (**94b**) = 170 M^−1^, *K*_ass_ (**94a**) = 600 M^−1^), Arg-OMe (*K*_ass_ (**94b**) = 120 M^−1^, *K*_ass_ (**94a**) = 600 M^−1^), and His-OMe (*K*_ass_ (**94b**) = 30 M^−1^, *K*_ass_ (**94a**) = 200 M^−1^) forming 1:1 complexes. More H-bonding sites increase the binding strength. Modification of the lower rim of the calix[4]arene skeleton by bridging ligands lowered the complexation ability of the more rigid molecule **93b** although its binding selectivity was preserved.

**Figure 63:**
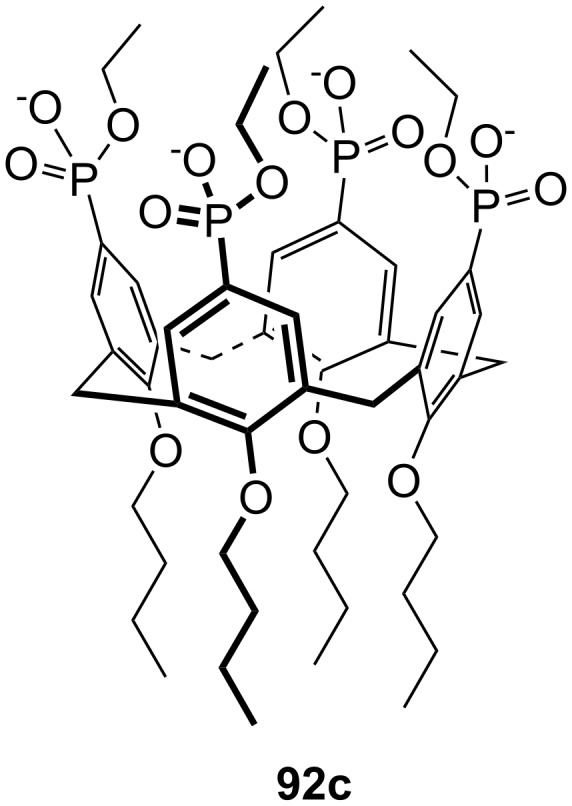
Calix[4]arene tetraphosphonic acid ester (**92c**) for surface recognition experiments.

Calixarene tetraphosphonate (**92c**) (Figure 63) was described as specific receptor for basic amino acids, with preference for arginine (**81d**). Binding constants in methanol ranged from 7.9 × 10^2^ M^−1^ for Ac-Lys-OMe (Lys, *K*_ass_ = 3 × 10^3^ M^−1^) to 1.9 × 10^4^ M^−1^ for Ts-Arg-OMe (Arg, *K*_ass_ = 7.9 × 10^2^ M^−1^). Consequently, this host molecule was used in lipid monolayers for recognition of peptides and basic protein surfaces in buffered aqueous solution [308–309] (HEPES), and the binding events monitored with the aid of a Langmuir film balance. Histone H1 and Cytochrome C were recognized in the range of 10^−8^ mol/L guest concentration [306].

Similar calix[4]arenes with α-aminophosphonic acid fragments at the upper or lower rim were described and their remarkable selectivity as carriers for zwitterionic aromatic amino acids in membrane transport reported [310].

**Figure 64:**
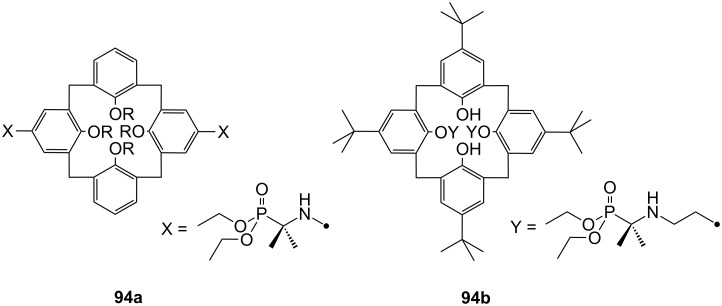
Calixarene receptors **95** with α-aminophosphonate groups.

By introducing these H-donor and H-acceptor groups in the host skeleton, it was shown that a calix[4]arene molecule binds hydrophilic amino acid zwitterions in its polar cavity: Two aminophosphonate groups at the lower rim (Figure 64) lead to selective transport of His over Phe, Tyr and Trp, while upper rim modification changes the selectivity towards Phe. In the later case the substituents can participate in complexation and recognize the aromatic side chains of amino acids. The selectivity of membrane transport for phenylalanine (**81a**) was enhanced 40 times over tryptophan (**81b**) (fluxes ratio for **95a** −7.3, for **95b** −4.9).

In addition, phosphorylated calixarenes have been used to bind uracils (*K*_ass_ up to 5.43 × 10^4^ M^−1^) in aqueous solvent mixtures [311–313]. Together with the examples **92** and **94**, a whole series of phosphonate substituted calixarenes for amino acids binding has been reported, which have proved to be more versatile than the *p*-sulfonatocalix[*n*]arenes and applicable at pH values closer to those found under physiological conditions. The binding constants for amino acids in water are of the same order of magnitude for both functionalizations, where comparable. The preference for basic amino acids is evident.

#### 3.2. More complex calixarenes: optical readout, enantiodiscrimination, bridges and caps

Calixarenes have been modified to exhibit special properties such as optical readout by chromophoric groups, enabling quick and easy monitoring of guest binding, or by groups supplying chirality for enantiodiscrimination. In addition, the cavity has been expanded or rigidified by bridges or even caps to improve binding properties. Often no sharp dividing line can be drawn between these concepts. We present now the current approaches, where we try to keep the direction, starting with optical readout systems, followed by calixarenes for chiral recognition and then go on to more complex systems ending with capped moieties with additional functionalities.

**Figure 65:**
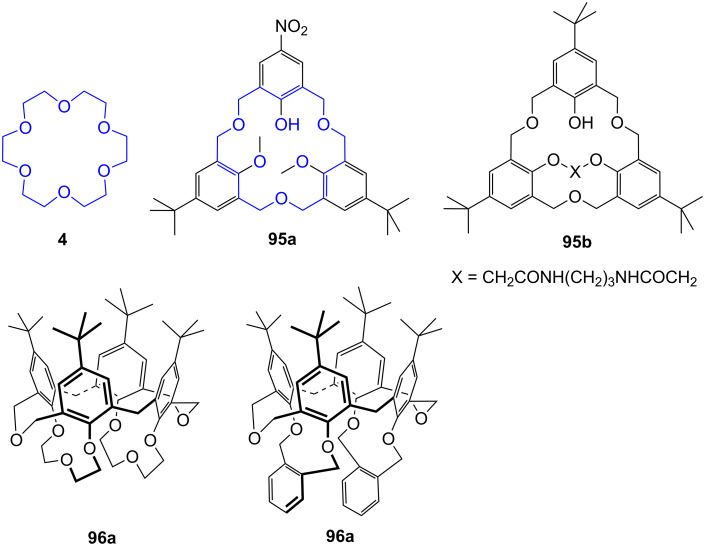
A bridged homocalix[3]arene **95** and a distally bridged homocalix[4]crown **96**.

Bridging of calixarenes and resorcinarenes with ethyleneglycol chains leads to calixcrowns and resorcinarene crowns, or even calixcryptands [314]. The synthesis, structure and fundamental properties of such systems have been reviewed [315]. We will point out their application in ammonium ion recognition in comparison to other calixarenes with selected examples. Related systems carry ether bridges in the calixarene ring (Figure 65). Such homocalixarenes are structurally similar to crown ethers (**4**) and can bind primary ammonium ions [316–320].

Two typical examples have been described by Chen et al. (**95**) [321] and Masci et al. (**96**) [322] (Figure 65). Compounds **95** show selectively binding ability towards linear primary alkylammonium ions from *n*-BuNH_3_^+^ to *n*-hexyl-NH_3_^+^ with the formation of 1:1 complexes in CDCl_3_/CD_3_CN and *K*_ass_ = 600 M^−1^. Compound **96a** binds the tetramethylammonium ion with *K*_ass_ = 280 M^−1^ in CDCl_3_.

**Figure 66:**
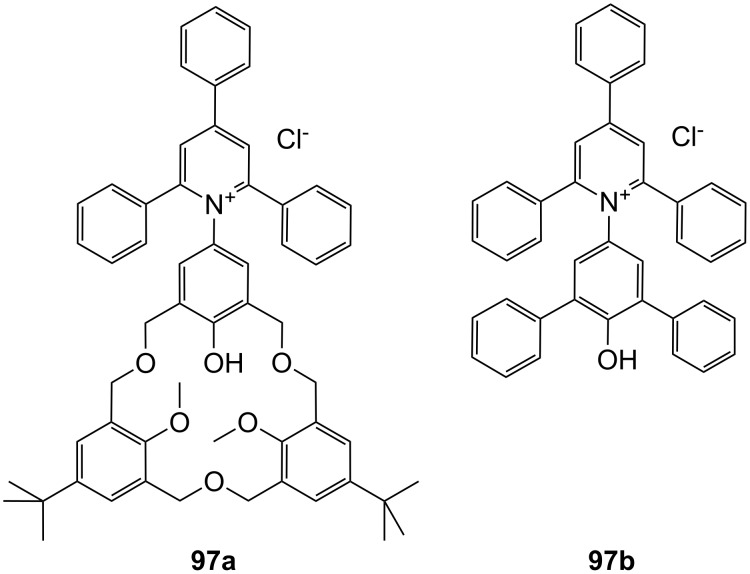
Homocalix[3]arene ammonium ion receptor **97a** and the Reichardt’s dye (**97b**) for colorimetric assays.

Homocalix[3]arene **97a**, reported by Tsubaki et al., consists of an 18-membered ring and six oxygen atoms available for cation co-ordination [323]. In addition, the molecule contains a Reichardts dye E_T_1 (**97b**) type pyridinium phenolate moiety (Figure 66), which becomes deprotonated upon ammonium ion binding. The resulting betaine structure shows long wavelength charge transfer absorption observable in the visible spectrum. Only compound **97a**, and not the dye E_T_1 (**97b**) itself, showed a color change upon addition of amines or an alkaline earth acetate. This confirms a binding process and excludes a simple deprotonation reaction as the origin of the color change. Due to steric reasons, primary amines are preferentially bound over secondary and tertiary amines. *N*-Butylamine showed a binding constant of 135 M^−1^ in DMSO.

**Figure 67:**
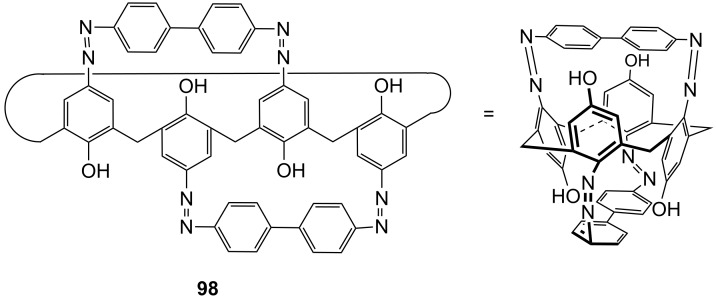
Chromogenic diazo-bridged calix[4]arene **98**.

Diazo-bridges in calix[4]arenes also allow distinguishing the binding of amines and diamines (or triamines) by color changes, caused by host–guest proton transfer [324]. Bisazobiphenyl-bridged chromogenic calix[4]arenes **98** (Figure 67) were employed as reagents for the visual discrimination of aliphatic and aromatic amines [325]. Various amines were added to **98** in DMSO resulting in distinct color changes. For instance, *tert*-butyl amine induced bathochromic shift of the absorption of 84 nm, whilst the addition of aromatic amines did not induce any color change or shift in the absorption maxima. The yellow color was restored upon acidification of a solution of the **98**-*tert*-butylamine complex. This indicated that the color change could be attributed to the ionization of hydroxyl groups of **98**. Conductometric titration gave further evidence: On addition of the guest, the conductivity continuously increased until it reached a plateau at equimolar concentration of amine.

In an earlier publication, Arduini et al. introduced short diethylene glycol bridges into calix[4]arene. The resulting derivative was successfully used for the cation–π-complexation study of methylammonium and tetramethylammonium ions [326]. When a crown ether moiety bridges a calix[4]arene at the lower rim it prefers primary ammonium ions over the isomeric derivatives (*n*-butyl >> *tert*-butyl) for steric reasons [327]; a similar selectivity was observed if two parallel crown-3 moieties at the lower rim are introduced in *p*-phenylcalix[4]arene [326] and the same order of preference was noted (i.e. *n*- >> *s*- > *tert*-butylamines) if two carboxymethoxy groups at the lower rim of a calix[4]arene are bridged by a crown-3 group [328].

**Figure 68:**
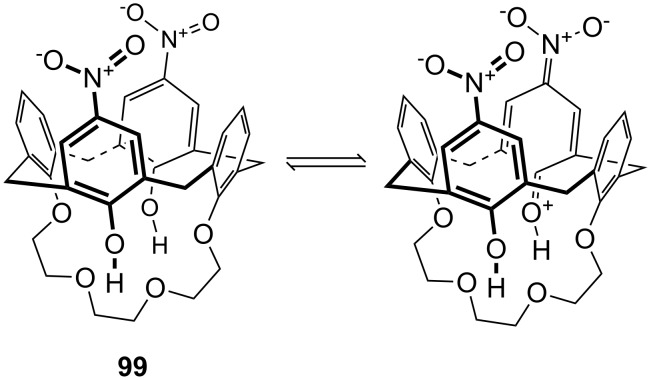
Calixarene receptor **99** by Huang et al.

The parent calix[4]arene was used by Huang to develop an amine receptor with optical readout. The dinitrated calix[4]arene is bridged by oligoethyleneglycol chains of different length (Figure 68) by the alkylation of the phenolic hydroxyl groups of the non-substituted arenes [329].

As in the previous examples, the binding of the amine by the resulting phenolate ion is crucial for the development of the color. Because of two phenols being deprotonable per calixarene, it is not surprising that the authors identified a 1:2 receptor to amine stoichiometry. For this class of receptors a clear preference for binding of primary amines over branched, secondary and tertiary guests was observed. For the depicted receptor they found the best binding properties with *n*-butylamine (*K* = 326 M^−1^) in chloroform.

Enantioselective analysis and separation of amino acids was addressed using chiral calixarene type macrocycles: A pseudo-*C*_2_-symmetrical homooxacalix[3]arene discriminates between chiral amino acids [138], whilst chiral calix[4]crown ethers were used for the binding of alkylammonium ions [330]. Amino acid esters were separated in liquid membrane transport experiments with an efficiency dependent on their hydrophobicity, with preference to *S*-Phe- and *S*-Trp- ester showing the highest flux [331].

**Figure 69:**
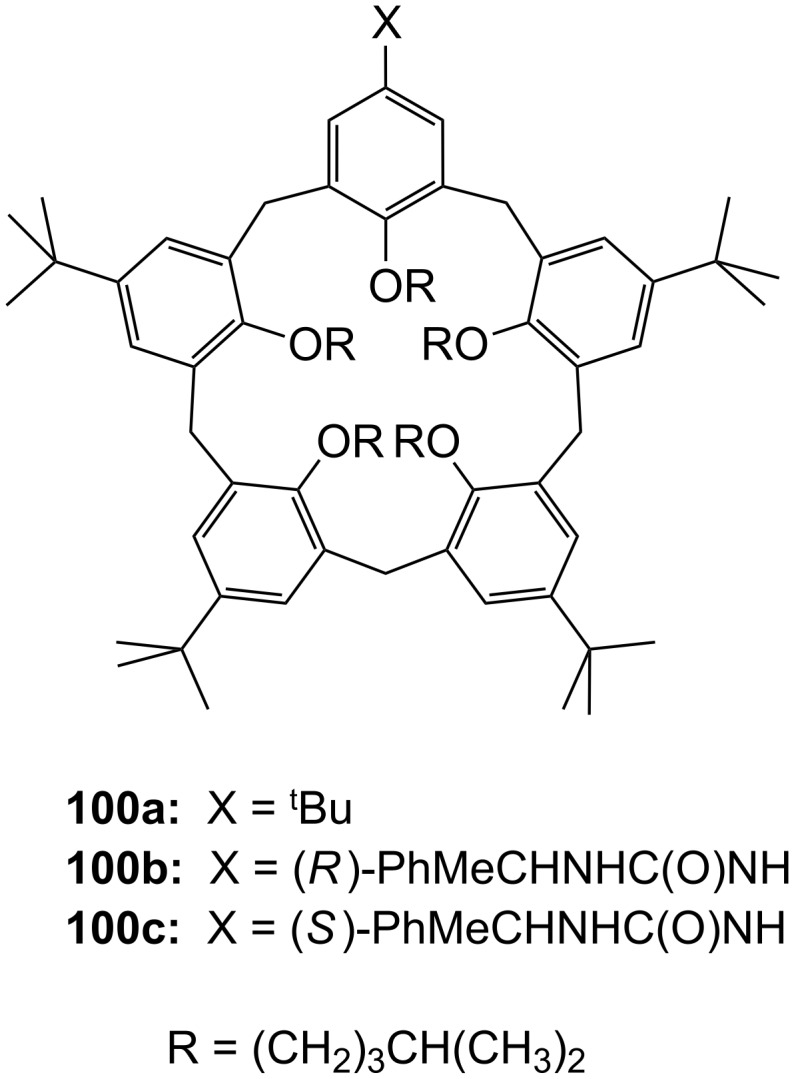
Calixarenes **100** reported by Parisi et al.

A calix[5]arene related to **82** for attempted enantiodiscrimination was reported by Parisi et al. [332]. Replacing the *tert*-butyl group (**100a**) by a urea functionality (**100b** and **100c**) on the upper rim (Figure 69) significantly improved the binding constants towards ammonium guests.

**Figure 70:**
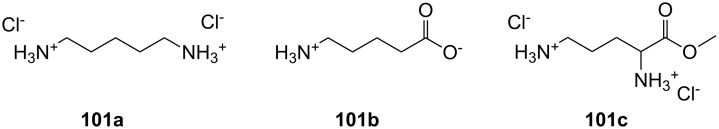
Guest molecules for inclusion in calixarenes **100**: DAP × 2 HCl (**101a**), APA (**101b**) and Lys-OMe × 2 HCl (**101c**).

The free rotation around the aromatic-*N*-(urea)-bond allows the urea unit to act as a hydrogen bond acceptor to bind ammonium ions and as a hydrogen bond donor for carboxylate binding. However, a comparison of the binding constants shows that carboxylate ions are bound more tightly. This is indicated by the difference between the binding of 1,5-diaminopentane dihydrochloride (DAP × 2 HCl, **101a**) and 5-aminopentanoic acid (APA, **101b**) (Figure 70, Table 5). The chirality of the receptors **100b** and **100c** did not lead to any enantiodifferentiation of chiral guest molecules.

**Table 5:** Binding constants of different guest molecules (**101**) with receptors **100** (NMR titration in C_2_D_2_Cl_4_/CD_3_OD 2/1).

Receptor	**101a**	**101b**	**101c**

**100a**	300 M^−1^	1070 M^−1^	43 M^−1^
**100b**	12820 M^−1^	16140 M^−1^	2240 M^−1^
**100c**	11860 M^−1^	16850 M^−1^	2190 M^−1^

**Figure 71:**
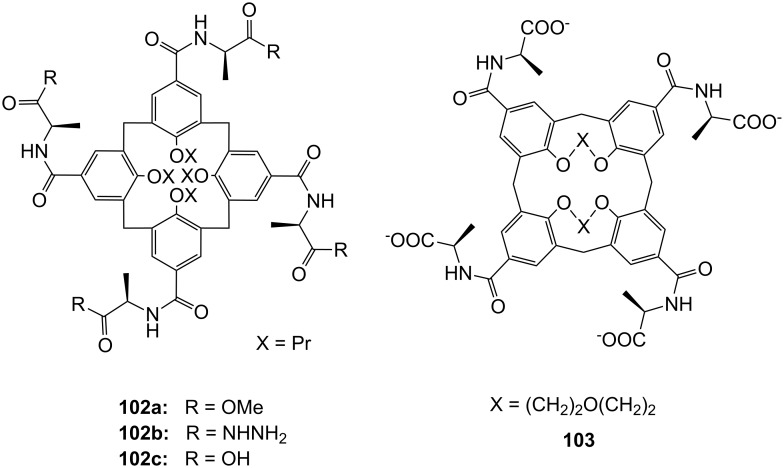
Different *N*-linked peptido-calixarenes open and with glycol chain bridges.

The inclusion properties of the chiral cone peptidocalix[4]arenes **102** with different conformation flexibility (Figure 71) towards aliphatic and aromatic amino acids and their methyl esters were investigated in D_2_O (pD 7.3, phosphate buffer) [333]. The authors compared the recognition properties towards α-amino acids and aromatic quaternary ammonium cations of **102c**, and the more rigid water soluble peptidocalix[4]arene **103** by ^1^H NMR titration experiments. The complexation occurred exclusively through the interaction of the calixarene cavity with the apolar groups of the guests [334].

Rigid receptor **103** with two di(ethylene glycol) units introduced in proximal positions at the lower rim of the calix[4]arene skeleton (Figure 71) was much more efficient than the flexible analogue in all complexation processes. Aromatic molecules were better bound than aliphatic ones with the highest association constants values *K*_ass_ = 110 and 620 M^−1^ for *S*-Trp and *S*-Trp-OMe, respectively [335]. The magnitude of log *K*_ass_ decreased with decreasing hydrophilicity (log *K*_ass_ in brackets): *R*-Trp-OMe, *S*-Trp-OMe (2.8) > *R*-Phe-OMe, *S*-PhGly-OMe, *S*-Phe-OMe (2.6) > *S*-Leu-OMe (2.5) > *S*-Val-OMe (2.3) > *S*-Tyr-OMe (2.2) > *S*-Ala-OMe, *S*-Trp (2.0) > *S*-Phe (1.8) > *S*-Tyr, *S*-Leu (<1.3) > Ala, Val, Gly. A similar behavior was noted on examining the pH dependence of the association constant between **103** and *S*-Phe-OMe: pH = 6.0 (*K* = 710 M^−1^), pH = 7.3 (*K* = 400 M^−1^) and pH = 8.0 (*K* = 220 M^−1^), corresponding to the decrease in the percentage of the protonated guest species. The hydrazides of these “*N*-linked-peptido-calixarenes” were able to extract complementary amino acids and dipeptides such as acetyl-*R*-alanine and acetyl-*R*-alanyl-*R*-alanine.

Introduction of chirality by the insertation of an amino acid into the ring of the calixarene moiety potentially enables enantiodiscrimination properties by the formation of diastereomeric complexes with racemic ammonium ions [336].

**Figure 72:**
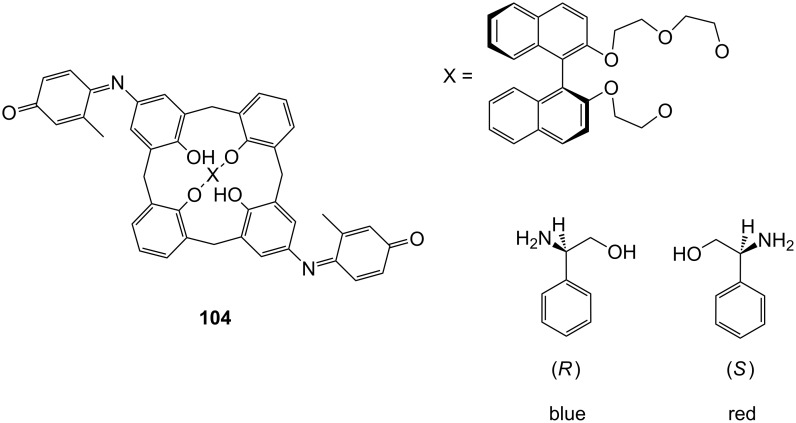
(S)-1,1′-Bi-2-naphthol calixarene derivative **104** published by Kubo et al.

For the visual discrimination between enantiomers, Kubo et al. synthesized a receptor (**104**) which undergoes a color change upon the binding of chiral substrates [337] (Figure 72). Upon binding of the enantiomers, two different bathochromic spectral shifts of the two chromophores attached to the binding cavity were observed, with significant optical response only for one enantiomer. The best strongest binding occurred with (*R*)-phenylalaninol salt in ethanol *K*_ass_ = 159 ± 16 dm^3^ mol^−1^. The formation of a 1:1 complex was confirmed by mass spectroscopy. Other amino acids enantiomers, such as the those from phenylglycine, were distinguishable with the system.

**Figure 73:**
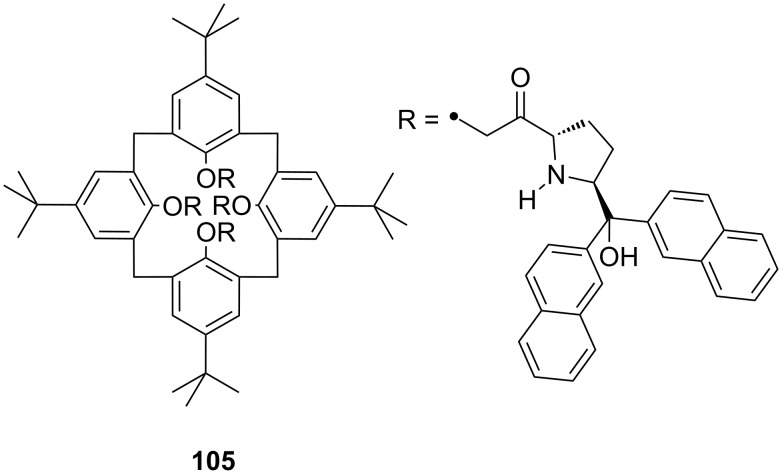
A chiral ammonium-ion receptor **105** based on the calix[4]arene skeleton.

Diamond et al. synthesized compound **105** to obtain a sensor (Figure 73) which discriminates enantiomers by hydrogen bonding interactions [338–339]. Without directly observable optical readout option, the fluorescence quenching of the receptor’s emission was investigated in chloroform (λ_ex_ 274 nm). Compound **105** shows some selectivity for (*R*)-1-phenylethylamine and also discriminates between the enantiomers of phenylglycinol in methanol.

**Figure 74:**
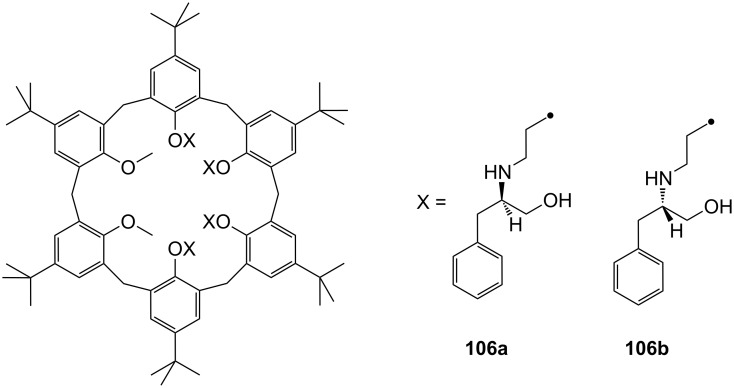
*R*-/*S*-phenylalaninol functionalized calix[6]arenes **106a** and **106b**.

*p*-*tert*-Butylcalix[6]arenes were modified with chiral amino alcohols (Figure 74) to achieve enantioselective binding of amino acids and amino alcohols [340]. The extraction properties of the two homochiral receptors **106a** and **106b** for some amino acid methyl esters and amino alcohols were studied by liquid–liquid extraction. The results show that these derivatives were excellent extractants for all the amino acids and amino alcohols, but only a weak or no chiral discrimination of the guests was found. Table 6 shows some selected results.

**Table 6:** Extraction abilities in % of receptors **106a** and **106b**.^a^

Receptor	**106a**	**106b**

*S*-Ala-OMe	91.4	84.3
*R*-Ala-OMe	89.1	89.6
*S*-Phe-OMe	90.3	87.2
*S*-Phe-OMe	90.7	82.5
*R*-Trp-OMe	87.5	85.4
*S*-Trp-OMe	93.2	89.8
*R*-phenylglycinol	92.3	83.5
*S*-phenylglycinol	72.5	87.6

^a^Extraction for 1 h from water with 2.0 × 10^−5^ M ammonium picrate to CH_2_Cl_2_; 25 °C

The inclusion of quaternary ammonium cations in the cavity of calixarenes with more enclosing substituents, has been extensively studied over the years in the gas phase, in solution and in the solid state [341–342]. The next step is to close the cavity from one side, to bridge or cap the moiety. Bridging of the upper rim of a calixarene may lead to altered selectivity and higher binding constants due to the pre-organized and fixed cavity.

**Figure 75:**
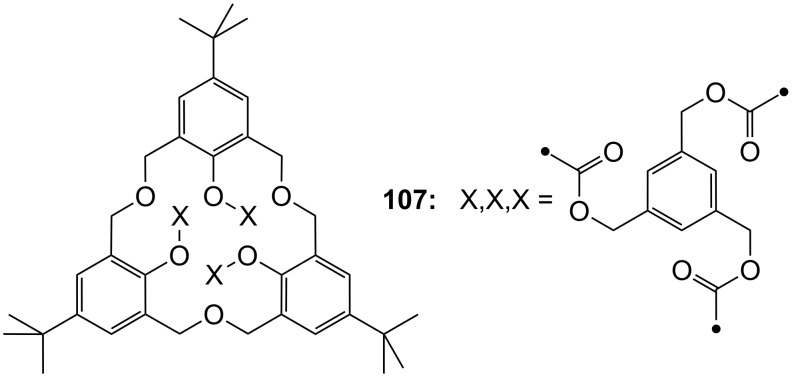
Capped homocalix[3]arene ammonium ion receptor **107**.

A triply bridged capped *C*_3_-symmetric hexahomotrioxacalix[3]arene **107** (Figure 75) exhibited high affinity (*K*_ass_ = 7.6 × 10^4^ M^−1^) for the *n*-butylammonium ion [343]. The association constant of receptor **107** with the picrate salt was determined in CH_2_Cl_2_/THF (99:1, v/v) by the Benesi–Hildebrand equation and exhibited a very well-defined linear shape for a 1:1 interaction.

**Figure 76:**
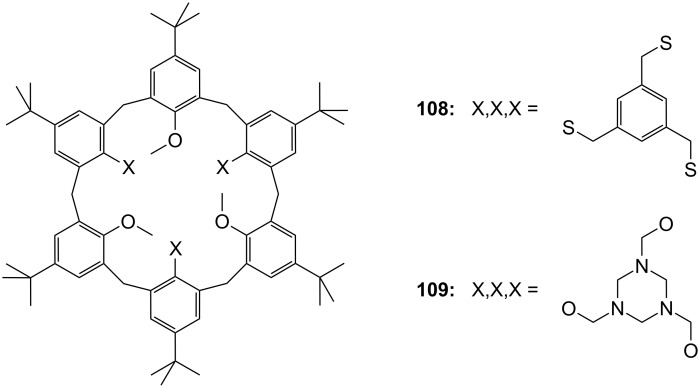
Two *C*_3_ symmetric capped calix[6]arenes **108** and **109**.

A three point connected thioether bridge led to a rigid calix[6]arene moiety (**108**) with *C*_3_ symmetry [344] (Figure 76). This pre-organization enabled better cation–π-interactions with the derivative **108** resulting in a 10–20 fold enhanced association constant for trimethylanilinium iodide (CD_2_Cl_2_, *K*_ass_ = 10^2^ dm^3^mol^−1^) compared to the reference compound hexamethoxy-*tert*-butylcalix[6]arene.

Compound **109** held rigidly in the cone conformation (Figure 76) displayed an exceptionally high affinity for small ammonium ions forming *endo*-complexes [345]. Extraction and competitive binding experiments gave values that were, at that time, the highest ever obtained with a calixarene-type host. The best affinity was observed for ethylammonium picrate (*K*_ass_ = 3.3 × 10^4^ M^−1^) with a more than 100 fold stronger association constant than butylammonium- and secondary ammonium ions. Quaternary ammonium ions were not complexed in chloroform. With the aid of X-ray diffraction, the authors identified the origin of the strong inclusion as contributions of hydrogen bonding to both, the aza cap and one phenolic unit of the calixarene, and to cationic as well as to CH–π-interactions between the ammonium ion and the aromatic walls of the host compound.

**Figure 77:**
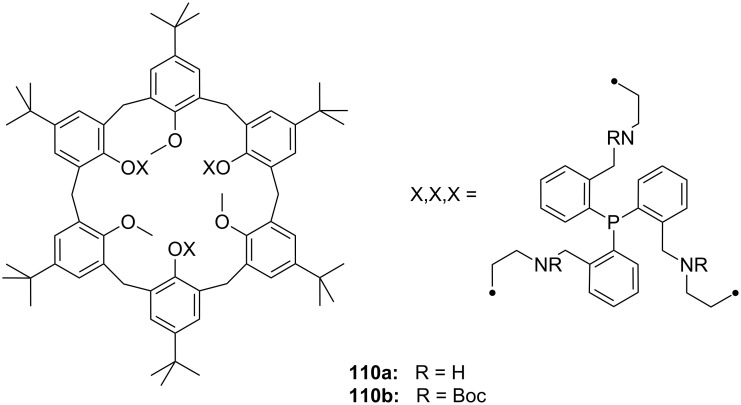
Phosphorous-containing rigidified calix[6]arene **110**.

A *C*_3_*_v_*-symmetrical calix[6]cryptand with a P,N-crypto cap was prepared leading to a pre-organized well-defined hydrophobic cavity open at the large rim (Figure 77). The free base of **110a** is able to complex cationic ammonium guests. ^1^H NMR studies showed that the methoxy substituents point towards the inside of the cavity.

**Figure 78:**
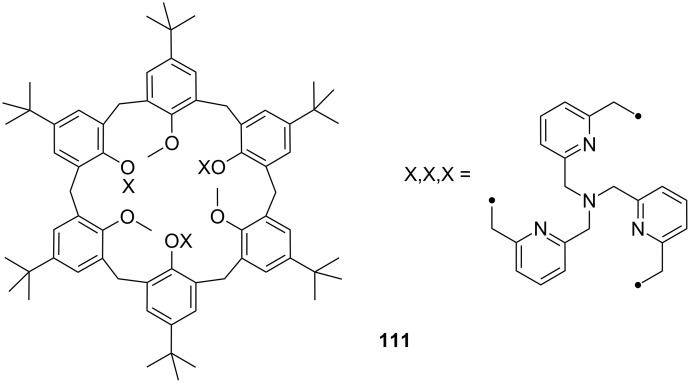
Calix[6]azacryptand **111**.

Reinaud et al. provided another example of synergistic combination of a polyaza and a calix[6]arene structure: Calix[6]tmpa **111** [346] (Figure 78). The compound behaved as a single proton sponge and appeared reluctant to undergo polyprotonation, unlike classical tris(2-pyridylmethyl)amine (tmpa) derivatives.

Calix[6]tmpa **111** and its sodium and protonated species display conformational properties that differ from the properties previously observed for other calix[6]-azacryptands: ^1^H NMR studies indicated that the ligand, as well as its complexes, adopt a flattened cone conformation probably due to the high steric constraint from the tmpa cap.

The monoprotonated derivative behaved as a good receptor for amines, leading to inclusion complexes, and as a good host for ammonium ions. Interestingly, it strongly binds sodium ions and neutral guest molecules, such as ureas, amides, or alcohols, co-operatively. Since it preferentially includes cyclic ureas, amides, or alcohols rather than primary amines, the group found the first example of a funnel complex binding an alkali-metal cation, comparable with related Zn^2+^ funnel complexes [347]. It displayed five fold selectivity in favor of propylammonium hydrochloride over the corresponding ethyl- and two fold selectivity over the butyl–guest in chloroform.

Even larger structures, based on this trimethoxy-calix[6]arene scaffold triple-bridged with a cyclotriveratrylen or connected to dimers via alkyl bridges, were applied for ammonium ion pair inclusion [348].

**Figure 79:**
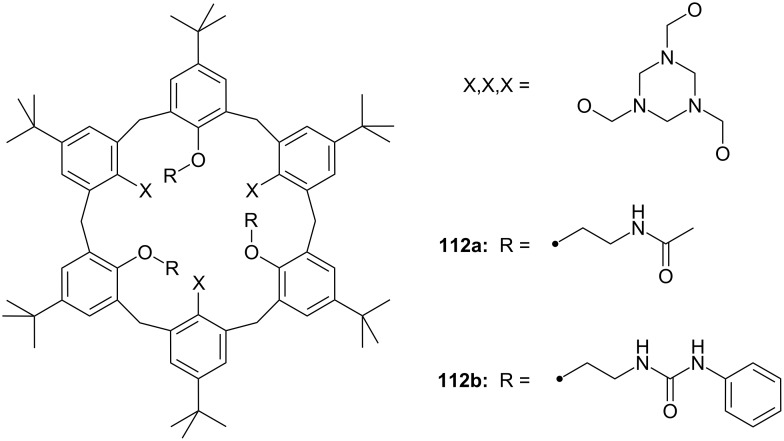
Further substituted calix[6]azacryptands **112**.

The use of such ditopic receptors and capped calixarenes with enhanced strength by ion-pair recognition has been an emerging field. In succession of the presented examples, a second generation of the hosts has been introduced [349]. These heteroditopic receptors (Figure 79) can bind ammonium ions or organic ion pair salts with a positive co-operativity [350]. The host–guest properties of receptors **112a** and **112b** toward the picrate and chloride salts of propylammonium ion were studied by ^1^H NMR spectroscopy and compared to **109**. No distinct binding constants were reported, but addition of 1 equiv of PrNH_3_^+^Pic^−^ to CDCl_3_ solutions of **112a** or **112b** led to the quantitative formation of the corresponding endocomplexes [**112a**·PrNH_3_^+^], Pic^−^ and [**112b**·PrNH_3_^+^], Pic^−^. With XCl, in comparison with [**109**·PrNH_3_^+^], Cl^−^, a much larger amount of [**112b**·PrNH_3_^+^], Cl^−^ was produced with less than 1 equiv of PrNH_3_Cl. This highlights that the simultaneous binding of the anion by the urea groups of the ditopic receptor **112b** enhances the endocomplexation of the ammonium ion and consequently a much larger binding constant should be observed compared to the first generation molecule **109**.

#### 3.3. Resorcinarenes and deeper cavities

**Figure 80:**
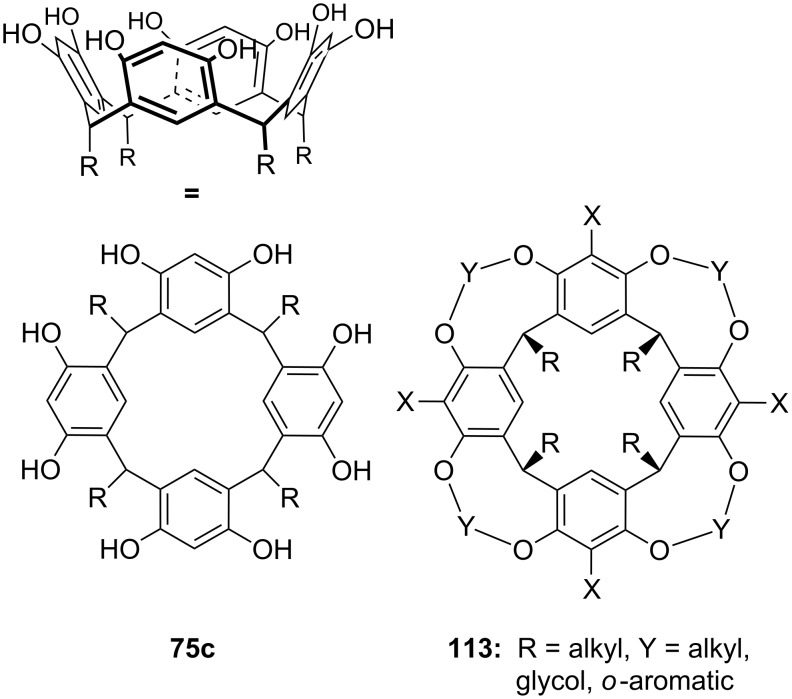
Resorcin[4]arene (**75c**) and the cavitands (**113**).

Resorcin[4]arene (**75c**) is a macrocycle with eight hydroxy groups at the upper rim, which form intramolecular H-bonds (Figure 80). Their interior is much smaller than that of cucurbituril. Resorcinarenes are versatile compounds for molecular recognition [351–353] and like calixarenes, they include guest molecules in the bowl-shaped cavity (cation–π-interaction).

The monomeric resorcinarene (**75c**) and its simple derivatives show recognition properties, but their shallow curvatures cannot provide sufficient surface contacts for selecting between targets. Nevertheless, they bind ammonium ions, choline (**76**), acetylcholine (**3**), and carnitine (**77a**) in protic solvents [354–357]. Larger guests such as DABCO can also be included [358–359]. Significant interactions to the ammonium ion can also occur via hydrogen bonds to the phenolic OH-groups. In unsubstituted resorcinarenes, these are preferably formed intramolecularily involving two neighboring OH groups of the host. For example, in dilute aqueous sodium hydroxide solution (pH 12–13) the tetraanionic structure, in which one hydroxyl group per aromatic moiety is deprotonated and stabilized by a strong intramolecular hydrogen bond, can bind tetralkylammonium ions in the 10^4^–10^5^ M^−1^ range [360].

**Figure 81:**
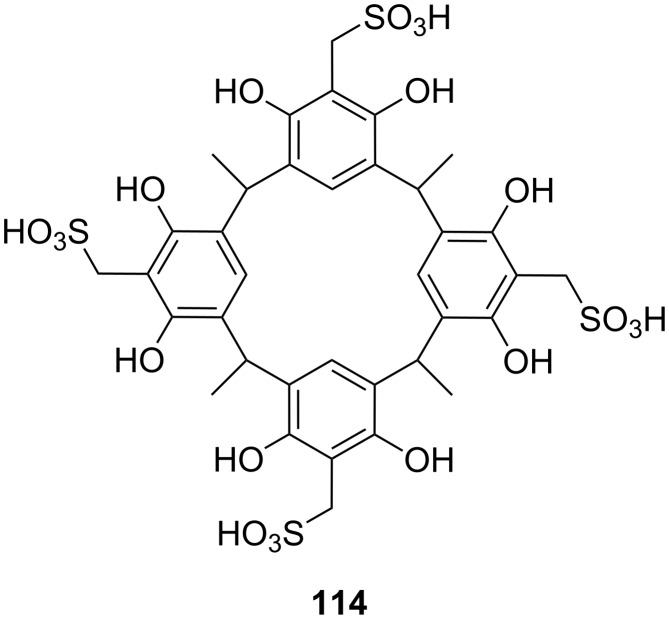
Tetrasulfonatomethylcalix[4]resorcinarene (**114**).

Similar to *p*-sulfonatocalix[*n*]arenes (**84**) tetrasulfonatomethylcalix[4]resorcinarene (Figure 81) forms complexes with amino acids in D_2_O (pD 7.2, phosphate buffer) [361]. The *K*_ass_ values for these complexes, estimated from ^1^H NMR experiments, decrease in the order Lys > Arg > Pro > Trp > Phe (with a maximum log *K*_ass_ of 3 for basic amino acids). No interactions with Asp, Asn, Thr, Leu, Met were observed.

**Figure 82:**
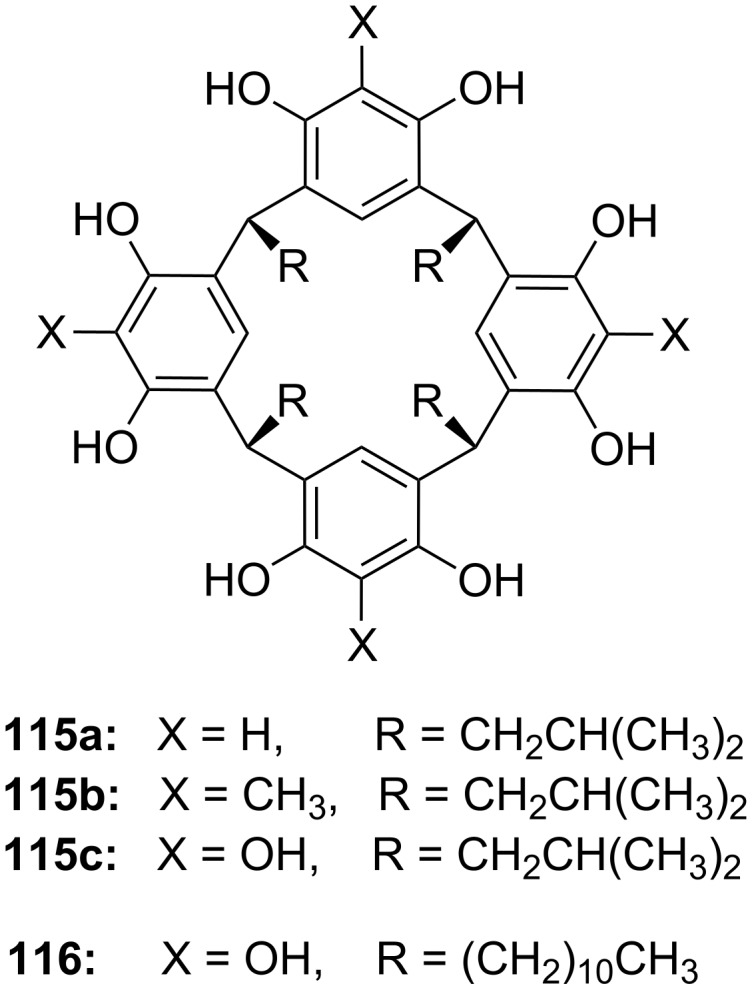
Resorcin[4]arenes (**115a/b**) and pyrogallo[4]arenes (**115c, 116**).

Only recently, the complexation properties of pyrogallo[4]arenes (**115c**) towards quaternary ammonium salts were compared with two resorcin[4]arenes (**115a/b**) [362] (Figure 82). The stability constants (*K*), standard free energy (ΔG_o_), enthalpy (ΔH_o_) and entropy changes (ΔS_o_) for the complexation of pyrogallol[4]arenes with ammonium cations were determined in ethanol by isothermal titration calorimetry. The binding strengths were in the order of 10^3^–10^4^ M^−1^ and generally 2 to 7 fold higher compared to the corresponding simple resorcinarenes. In the best example, diethyldimethylammonium and triethylmethylammonium ions were included in **115c** with *K*_ass_ = 6900 M^−1^ and 7500 M^−1^, respectively. The trends observed in the thermodynamic parameters for 1:1 and/or 1:2 host–guest complexations correspond to the systematic structural changes of the guest molecules. Molecular modeling calculations confirmed the results.

Similar pyrrogallol[4]arenes carrying long alkyl chains (**116**) were applied as amphiphilic receptors in an aqueous micelle system and their interaction with dopamine (**2**) and acetylcholine (**3**) studied by NMR methods [363].

**Figure 83:**
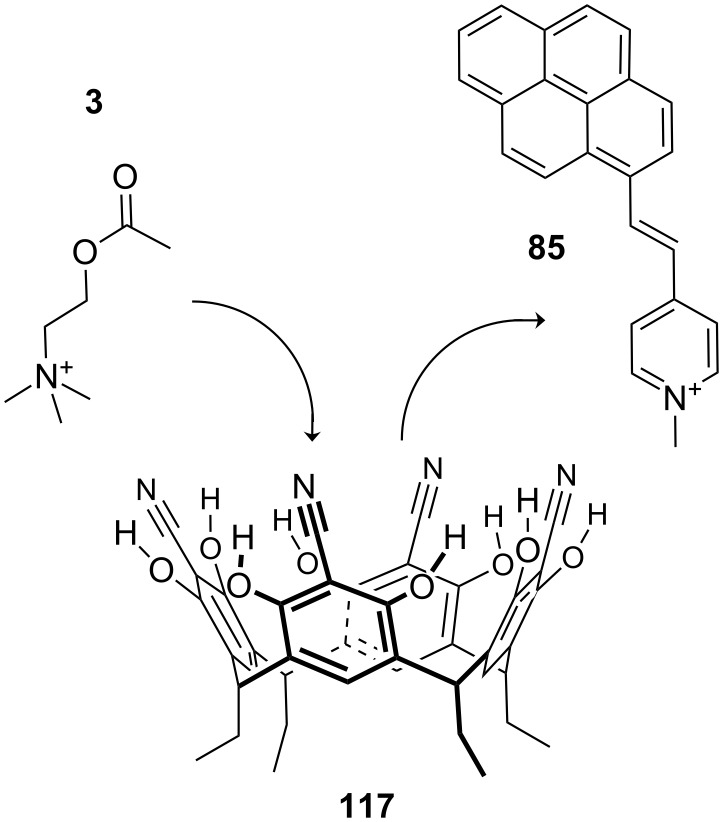
Displacement assay for acetylcholine (**3**) with tetracyanoresorcin[4]arene (**117**).

The inclusion of acetylcholine (**3**) in resorcinarene (**75c**) via multiple cation–π-interactions was proved by crystallography [355]. Not surprisingly, resorcinarenes were also employed in a fluorescent displacement assay (Figure 83) for acetylcholine (**3**). Similar to Shinkai’s study with *p*-sulfonatocalix[6]arene (**84b**), a tetracyanoresorcin[4]arene (**117**) in comparison to the parent compound **75c** (R = Et) was used as complex with indicator **85** [364]. The binding constants observed for acetylcholine (**3**) were 2 to 2.5 fold higher for the tetracyanoresorcin[4]arene (**117**). This was attributed to the larger contact area and a more suitable p*K*_a_ value of the resorcinarene in consequence to the strong electron withdrawing effect of the cyano groups. With increasing pH, acetylcholine (**3**) was bound more strongly by the receptors, with a *K*_ass_ of up to 10^6^ in phosphate buffer at pH 8.

**Figure 84:**
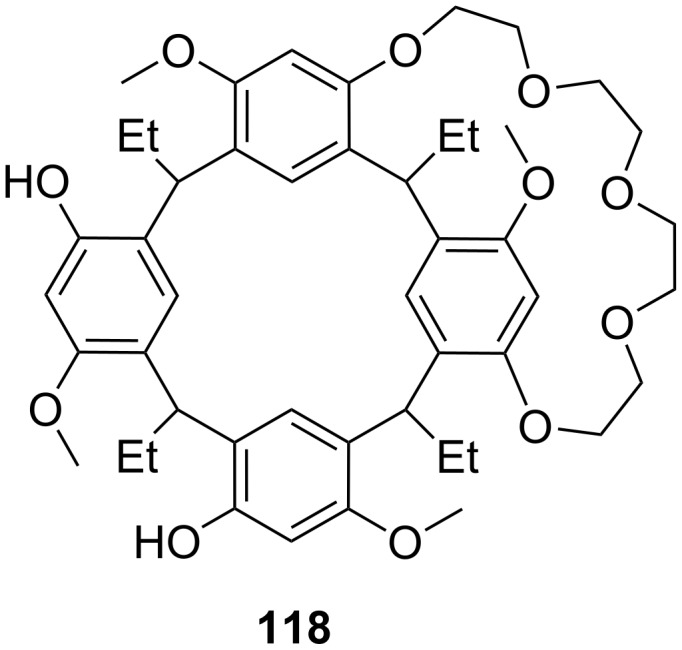
Tetramethoxy resorcinarene mono-crown-5 (**118**).

A mono-bridged resorcinarene host for acetylcholine (**3**) with tetramethoxy resorcinarene mono-crown-5 (**118**) was reported [365] (Figure 84). The dual nature of the cavity formed between the crown bridge at one end and the two hydroxyl groups at the other offers a better fit to acetylcholine (**3**) compared to the smaller tetramethylammonium cation. Acetylcholine (**3**) is able to interact with both the crown ether moiety and the free hydroxyl groups of receptor **118** simultaneously: the quaternary trimethylammonium group binds to the crown moiety through cation–O and cation–π-interactions, whereas hydrogen bonding interactions prevail between the acetate group and the hydroxyl part of the cavity. The binding of acetylcholine (**3**) to **118** was investigated by an ^1^H NMR titration technique in CDCl_3_ and showed 1:1 host–guest complex formation. The titration data indicated a stability constant of 150 M^−1^, which is 10–10^3^ orders smaller compared to the values found with acetylcholine complexes of resorcinarenes (**75c** and **117**), pyrogallolarenes (**115c** and **116**) or deep-cavitands (**126a/c**).

Following such a bridging approach, even deeper cavities (**113**) can be formed based on the structurally related resorcinarenes such as **75c** (Figure 80). By covalent bridging of the OH groups of two neighboring aromatic subunits by aromatic moieties, a resorcinarene can be made more rigid and the cavity formed can enclose guest molecules completely.

**Figure 85:**
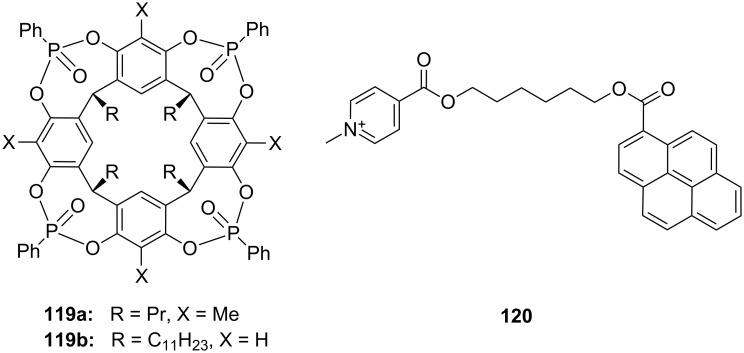
Components of a resorcinarene based displacement assay for ammonium ions.

One way of achieving this is the use of phosphonate-cavitands [366]. Following a similar principle as in the acetylcholine (**3**) displacement assays (**84b** or **117** + **85**) mentioned above, Prodi et al. reported a suitable protocol for the reversible complexation of methylammonium and methylpyridinium salts with the phosphonate cavitand **119** [367] (Figure 85). The *K*_ass_ values measured for the *N*-methyl complexes exceeded 10^7^ M^−1^ in dichloromethane. As displaceable guest they used compound **120**, consisting of a methylpyridinium unit as recognition moiety connected to a pyrene probe via a diester. In this molecule the cation–dipole interactions and CH_3_–π-interactions of the acidic +N–CH_3_ group with the π-basic cavity could be assisted in a synergistic manner by two simultaneous hydrogen bonds to the phosphonate groups. In the case of protonated secondary amines such as *N*-methyl-butylamine, a *K*_ass_ = 7.8 × 10^6^ M^−1^ was determined for **119a**.

**Figure 86:**
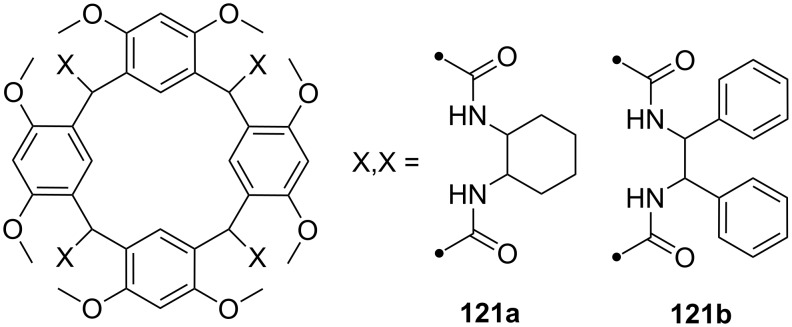
Chiral basket resorcin[4]arenas **121**.

As a different approach of cavity deepening, Botta, Speranza and colleagues presented both enantiomers of the two chiral basket resorcin[4]arenes **121a** and **121b** rigidified and doublespanned with 1,2-diaminocyclohexane and 1,2-diphenylethylenediamine bridges, respectively, in a flattened cone conformation [368] (Figure 86). Binding constants were not reported, but in several ESI-experiments the proton bonded diastereomeric complexes with amino acid guests exhibited a pronounced selectivity towards the enantiomers of tyrosine methyl ester and amphetamine. An additional kinetic study on the base-induced displacement of the guest revealed that the *S*-Tyr-OMe and *R*-amphetamine enantiomer was displaced faster from the heterochiral complex than from the homochiral one.

Cavitands [369] and carcerands [370] are additional examples of resorcin[4]arene based supramolecular host systems. Ideally, a synthetic receptor should provide a congruent surface and chemical complementarity to the target molecule. Cavitands (**113**) with (hetero-) arene linker between the resorcin[*n*]arene oxygen atoms, thus adding three or four walls to the resorcinarene skeleton, form a larger and deeper cavity than the according alkyl or glycol chain bridged homologues [279,371–373]. This not only increases the cavitand’s space but also increases the curvature. Non-functionalized resorcin[4]arenes are dominated by hydrogen bonding as driving force for complex formation and aggregation. For the latter cases, the resorcinol hydroxyl groups are functionalized and, therefore, π-interaction and electron donation become more important in their binding processes. Larger guests can be included, more surface capacitating cation–π-interaction is available and a stronger solvent shielding effect can be achieved. Thus, their binding properties and selectivities can be enhanced [374].

**Figure 87:**
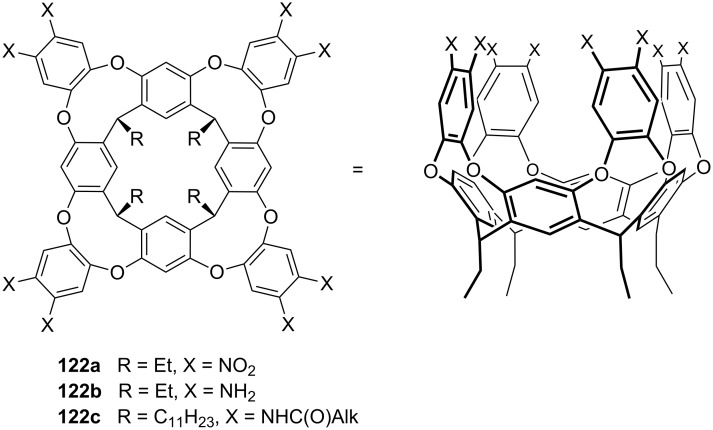
Resorcinarenes with deeper cavitand structure (**122**).

Two examples (Figure 87) of this were recently studied by Rebek et al. as a different concept for the molecular recognition of choline (**76**) and carnitine (**77a**). They enhanced the affinity and the selectivity by a better complementarity of size and shape instead of optimizing charge/charge attractions [374]. Specific cation–π attractions between the positive charge of the guest and the electron-rich aromatic surfaces of the host result in the formation of complexes with highly kinetic and thermodynamic stability. *R*-Carnitine (**77a**) is complexed with an association constant of 15000 ± 3000 M^−1^ reflecting the fact that its carboxyl and hydroxyl functions are well-positioned for hydrogen bonding to the amino groups at the rim of the host. Both choline hydrochloride (**76**) with 12000 ± 2400 M^−1^ and also tetramethylammonium chloride in DMSO with 22000 ± 4000 M^−1^ are bound tightly by **119a**. The molecule can be seen as a further development of the calixarene tetrasulfonate of Shinkai et al., which also had a very good affinity for choline (**76**) in water (log *K*_ass_ = 4.7), but was less selective.

**Figure 88:**
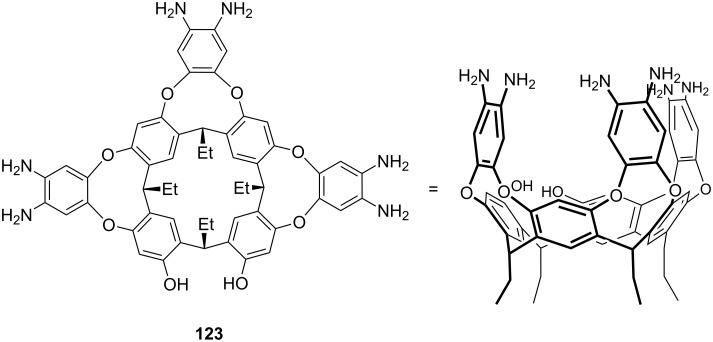
Resorcinarene with partially open deeper cavitand structure (**123**).

A comparable receptor molecule **123** (Figure 88) in a vase-like conformation was employed as supramolecular fluorescent sensor system for choline (**76**). The selectivity of the hybrid cavitand resorcin[4]arene receptor is explained by its enforced scoop-shaped cavity and multiple cation–π-interactions. Deprotonation in alkaline aqueous media afforded a negatively charged receptor which interacted more strongly by means of charge–charge attraction. NMR titration gave the stability constant of 0.1 × 10^2^ M^−1^ for **123** in DMSO with the tetramethylammonium chloride complex. The tetraethylammonium chloride was bound with a similar affinity, whilst the larger tetrapropylammonium chloride showed a sharp decrease in affinity. Choline (**76**) chloride was bound in pure DMSO with a *K*_ass_ of 80 M^−1^. In alkaline media (0.01 M KOH/DMSO) the stability constants for the complexes of tetramethylammoniumchloride and choline (**76**) hydrochloride were determined as 0.2 × 10^3^ and 0.1 × 10^3^ M^−1^, respectively. In dipolar aprotic solvents such as DMSO, the ammonium salt is recognized as a close contact ion pair. Consequently, the chloride may also interact with the receptor [375]. In protic solvents, such as methanol, **123** is a neutral species capable of forming thermodynamically stable complexes exclusively by cation–π and CH–π-interactions with ammonium cations which are complementary in size and shape.

**Figure 89:**
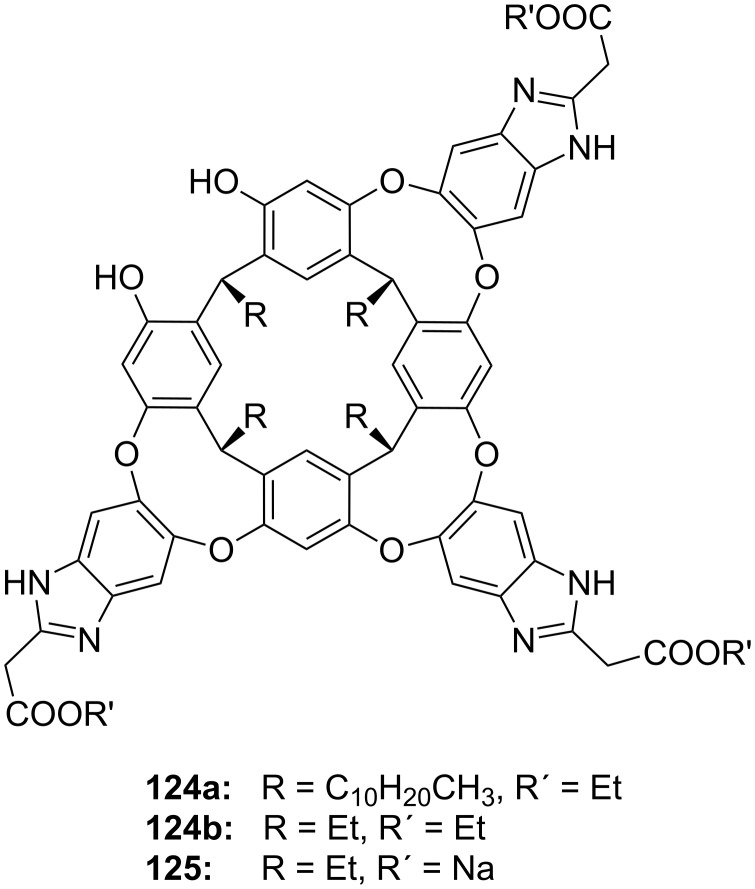
Water-stabilized deep cavitands with partially structure (**124**, **125**).

Rebek et al. reported a similar water-stabilized, deep cavitand (Figure 89) recognizing various amines and ammonium guests of different shapes. The absence of a fourth wall allows the binding of bulky ammonium groups [376]. In D_2_O saturated chloroform, **124a** strongly includes 1-aminoadamantane (*K*_ass_ = 1 × 10^3^ M^−1^) and carnitine (**77a**, *K*_ass_ = 2 × 10^3^ M^−1^) as measured by NMR titration methodology. Choline (**76**, *K*_ass_ = 4 × 10^2^ M^−1^) and carnitine (**77a**), which are poorly soluble in water-saturated chloroform, were taken up forming 1:1 complexes, but acetylcholine (**3**) was not. Such guests with small hydrophobic regions are accommodated with the trimethylammonium group positioned deep inside the cavity. The hydroxyl and carboxylate functions can then provide hydrogen bonding interactions with the groups at the rim. The ester group of acetylcholine (**3**) appears unable to reach such binding sites. Cavitand **125** exists as dimer or larger, kinetically unstable aggregates. With an excess of 1-adamantanol the aggregates break up and providing a sharp NMR-spectrum of a 1:1 complex. Other guests are not included or disassemble the aggregates.

**Figure 90:**
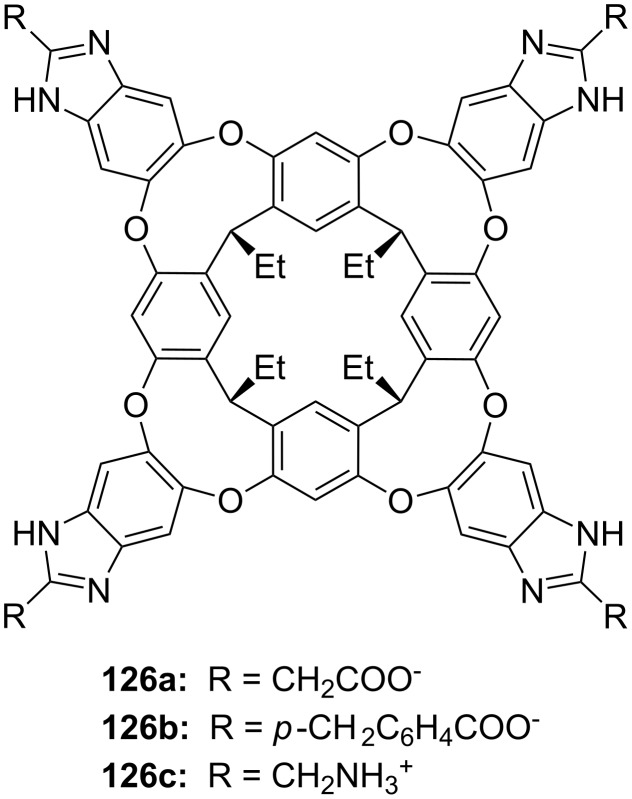
Charged cavitands **126** for tetralkylammonium ions.

Molecules of the cavitand family **126** (Figure 90) are all effective phase transfer catalysts which transport a hydrophobic guest, for example, an adamantyl residue from dichloromethane into water. If the reaction product is water soluble, it is easily released [377]. Compound **126a** forms stable 1:1 complexes with a variety of guests in water: (*S*)-nicotinium, chinuclidinium (both with *K*_ass_ > 10^4^ M^−1^), *R*-carnitine (**77**, 1.5 × 10^2^ M^−1^), choline (**76**, 2.6 × 10^4^ M^−1^) and acetylcholine (**3**, 1.5 × 10^4^ M^−1^) [378–379]. Compound **126b** shows a folded vase conformation in water and encloses cyclohexane and cycloheptane effectively (*K*_ass_ > 10^4^ M^−1^) [380]. Cavitand **126c** can distinguish between several substituted adamantyl residues [381].

Studies of **126c** with choline (**76**), acetylcholine (**3**) and carnithine (**77a**) were later extended. Binding mode and properties of these guest complexes were studied by NMR and calorimetry in water at pH 7.8 [382]. It was found, that **126c** preferably binds choline (**76**, 2.6 × 10^4^ M^−1^) over acetylcholine (**3**, 1.5 × 10^4^ M^−1^). The binding of carnithine is in comparison negligibly small (1.5 × 10^2^ M^−1^). The guest is inserted with its tetramethylammonium substituent deep in the cavity with the other end pointing to the carboxylic acid groups at the upper rim of the host.

#### 3.4. Larger structures, capsules and ditopic binders

**Figure 91:**
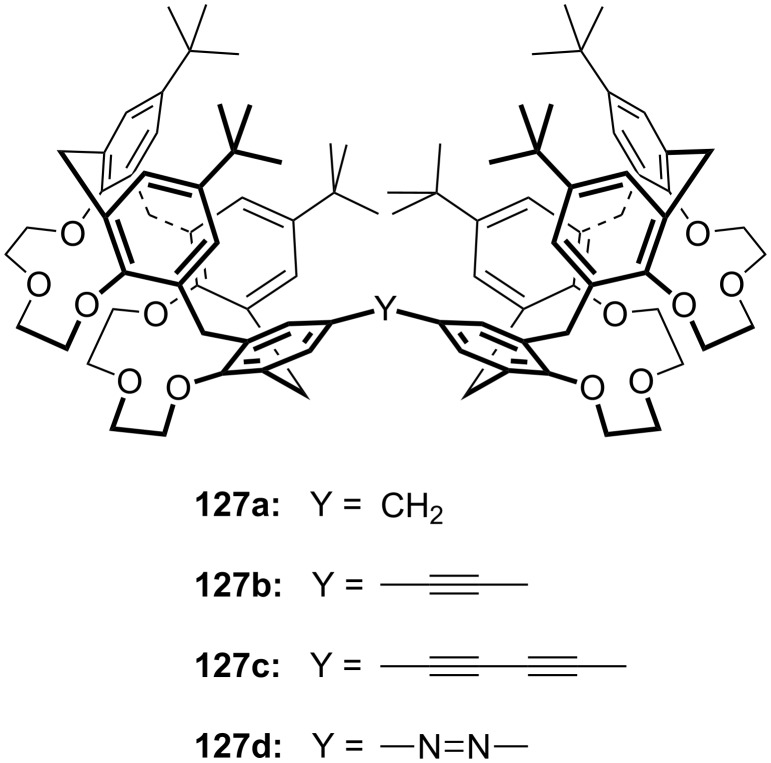
Ditopic calix[4]arene receptor **127** capped with glycol chains.

Enhancing the binding strength and the selectivity can also be achieved by adding more binding sites. Comparable to a hemicarcerand [85,383], two calixes can be connected by a suitable spacer to obtain a ditopic binder for ammonium ions (Figure 91). Using only one connection point makes the molecule sufficiently flexible to bind a bis-ammonium guest. Some recent examples of calixarenes following this concept have been published.

The binding abilities of a head-to-head linked bis-calix[4]arene-bis(crown-3) (**127**) fixed in the rigid cone conformation with bridges of different nature and length was described [384]. Tetraalkylammonium and *N*-methylpyridinium cations different in size and shape were investigated by ^1^H NMR spectroscopy in CDCl_3_ solution and in the more polar CDCl_3_/CD_3_CN solvent mixture. As a result a substantial decrease in the *K*_ass_ values was observed: association constants were generally almost an order of magnitude lower for all guests, due to CD_3_CN competing for the binding sites in the host. The double calixarenes have been found to exhibit efficiencies much higher than that of the corresponding reference cavitand calix[4]arene-bis(crown-3). The bridge present in these double calix[4]arenes dictated the orientation and distance between the two rigid caps and thus determine the efficiency and selectivity of binding. The two rigid caps could adapt in response to a potential guest and possibly co-operate in binding by forming a capsule.

**Figure 92:**
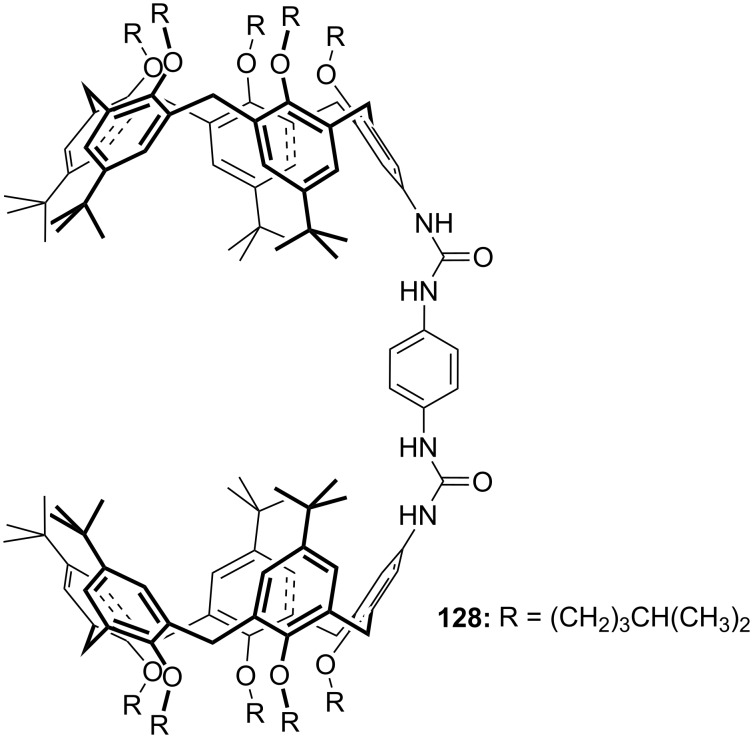
A calix[5]arene dimer for diammonium salt recognition.

Another ditopic receptor was described by the Parisis group [385]. It was developed for the binding of linear, long-chain α,ω-alkanediyldiammonium dichloride salts, combining the co-operative action of two converging calix[5]arene cavities in the encapsulation of the dication with the ability of the two ureido functions (Figure 92) to bind the relevant counter anions. Binding properties as well as the host–guest architectures, were investigated by a combination of ^1^H NMR spectroscopy in (CDCl_2_)_2_/CD_3_OD (2:1 v/v) and electrospray mass spectrometry (ESI-MS). Addition of the guest salts to a solution of **128** caused the formation of very strong inclusion complexes, whose host–guest stoichiometries (1:1 and/or 2:1) and geometries were dependent on the length of the diammonium ion and the [host]/[guest] ratio. The use of non-protic solvents showed a beneficial effect of the ureido functions by loosening the ion-paired salt and the association of the anion by formation of six-membered chelate rings with halide or picrate anions and eight-membered chelate rings with carboxylate anions. Table 7 shows the binding constants for long chain diammonium ions.

**Table 7:** Binding constants of different guests with the ditopic receptor **128**.

H_3_N^+^(CH_2_)*_n_*NH_3_^+^×2 Cl^−^	*K*_ass_ [M^−1^]^a^

*n* = 8	212
*n* = 10	163
*n* = 12	2400
*n* = 16	2600

^a^NMR titration in CDCl_3_/DMSO 3/2; 1:1 complexes; errors < 15%.

Biological molecules often possess ionic moieties as well as functional groups capable of forming hydrogen bonding interactions within the same molecule. It is quite appealing to consider ditopic cavities as binding sites based on this principle. Even larger structures can be assembled by complementary recognition of receptor parts to each other [386] – a more specialized case of recognition involving self assembly [387].

**Figure 93:**
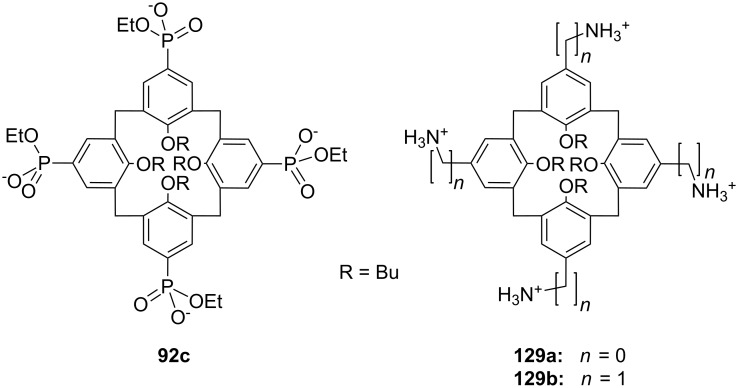
Calixarene parts **92c** and **129** for the formation molecular capsules.

In the following example the authors used the receptor structure **92c** and appropriate ammonium counterparts, for example **129a**, to form supramolecular assemblies [388] (Figure 93). From NMR titration, the stability constants *K*_ass_ of the assembly **92c** and **129a** was (7.0 ± 2.5) × 10^5^ M^−1^ in methanol, whilst for **92c** with **129b** the *K*_ass_ was (1.0 ± 0.4) × 10^4^ M^−1^ in methanol/water 4:1.

Zadmard et al. studied these capsules formed in polar solvents by two cone calix[*n*]arenes in greater detail (*n* = 4 and 6). The first was substituted at the upper rim by phosphonic ethyl ester lithium salt groups (**92c** and its calix[6]arene analogue), while the second consisted of ammonium cations (**129a** and its calix[6]arene analogue) [389] (Figure 93). Inclusion of Phe, aniline, tetramethylammonium salts and other organic molecules into the capsule cavity in methanol was investigated [390]. Since the capsules were far more stable than the complex with the guest molecule, 10^5^ vs. 10^3^ M^−1^ in methanol-*d*_4_, the authors concluded that a guest molecule was included inside the anionic half-sphere after opening the capsule.

**Figure 94:**
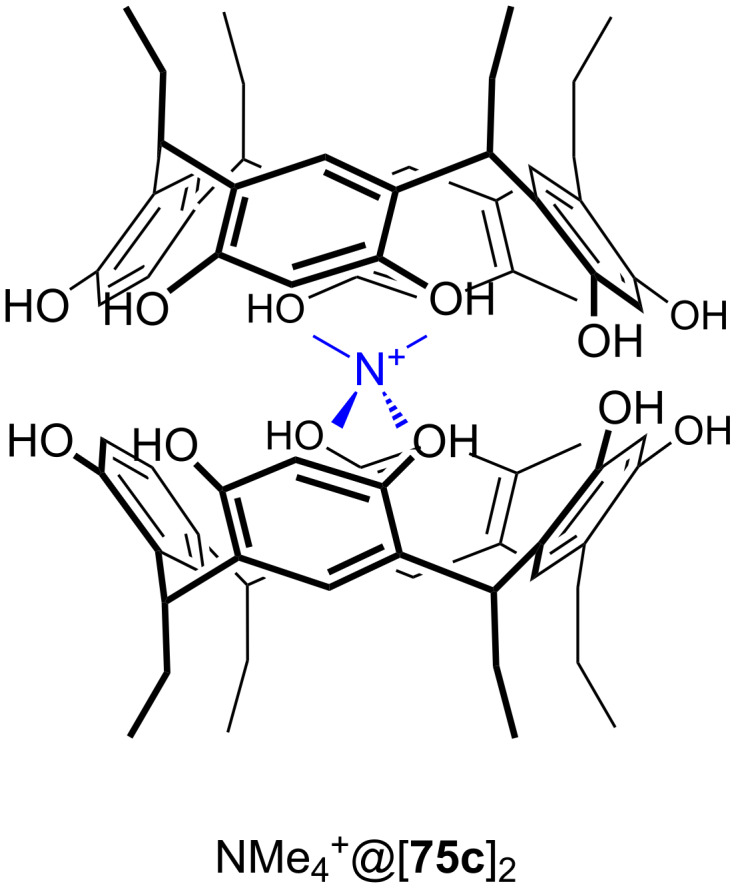
Encapsulation of a quaternary ammonium cation by two resorcin[4]arene molecules (NMe_4_^+^@[**75c**]_2_ × Cl^−^ × 6MeOH × H_2_O; for clarity, solvent molecules and counterions have been omitted).

Resorcinarene can also form dimers by a self assembling process, in which the cavity is filled [391]. For instance, the tetramethylammonium cation can be included (Figure 94). This was nicely evidenced by mass spectroscopy and by examining several crystal structures of smaller tetraalkylammonium cations with unsubstituted resorcinarenes such as **75c** with different alkyl chain lengths. Competitive mass spectrometric studies clearly indicated the preference for the tetramethylammonium cation over the tetraethylammonium cation and especially, the tetrabutylammonium cation. The two resorcinarene units are held together mediated by hydrogen-bonded networks via solvent molecules of methanol and water [392].

A tetralkylammonium ion (R = propyl to hexyl), together with one to three chloroform molecules can also be complexed and included in a capsule surrounded by six resorcinarenes stabilized by H-bonds [393].

**Figure 95:**
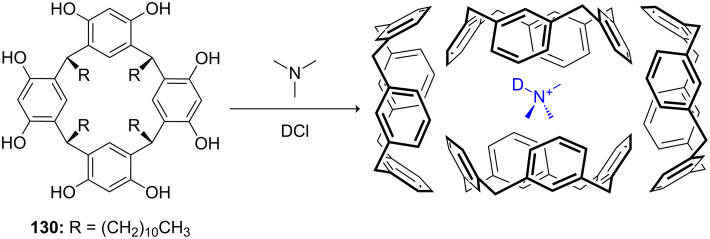
Encapsulation of a quaternary ammonium cation by six resorcin[4]arene molecules (NMe_3_D^+^@[**130**]_6_ × Cl^−^; solvent molecules, substituents and counterions are omitted for clarity; the last two resorcinarene calixes are arranged behind and in front of the scheme’s plane).

Expanding the studies, Cohen et al. demonstrated a pH dependent inclusion of quaternary ammonium salts in a hexameric structure such as **130** (Figure 95) in CDCl_3_ by NMR studies [394].

These selected, recent examples give an impression of the possibilities for ammonium recognition with calixarenes and resorcinarenes utilizing self assembly. A discussion of all options possible is beyond the scope of this review. The reader is referred to the appropriate literature [395–397]. Larger capsules for the inclusion of a variety of guests were recently described by the Rebek group [398].

The advantages of calixarenes as hosts for ammonium ion binding in comparison with other synthetic macrocycles is obvious: good accessibility, the possibility of tuning shape and size of the inner cavity and the introduction of various functional groups to address nearly any ammonium ion guest selectivity. Calixarenes are often used for the synthesis of more complicated and elaborated structures, to enclose or strongly complex larger guests with high selectivities and outstanding binding strengths.

Calixarenes often achieve selectivities in cation binding which are superior to crown ethers due to the guest inclusion being controlled by steric factors and various interactive forces of host and guest. Some calixarene-based artificial receptors show remarkable selectivities for amine isomer recognition. Especially noteworthy is their ability to complex strongly with quaternary ammonium ions, which outperforms nearly every other receptor class, except the cucurbiturils (see “4. Cucurbiturils and related structures”). This was applied in assays for such important biomolecules as acetylcholine (**3**).

A considerable number of synthetic receptors based on a calixarene framework for amino acids derivatives has been designed and studied in organic media, but only a few examples have been reported in aqueous solution. Calixarenes are able to select precisely basic or aromatic amino acids in aqueous solution. Because of this property, they can be applied even as enzyme mimetics.

### 4. Cucurbiturils and related structures

Behrend’s polymer was reported over a century ago as a by-product of aminal type polymers [399] however, the structure of the material was only fully characterized in 1981. Because of the resemblance of the barrel-shaped molecule to a pumpkin, the investigators gave the macrocyclic methylene-bridged glyconuril oligomers the name cucurbiturils, derived from the Latin name of the plant family (cucurbitacae). All have a hydrophobic cavity and two identical carbonyl-laced portals (“occuli”) in common and are readily prepared by the condensation of glyconuril with formaldehyde.

**Figure 96:**
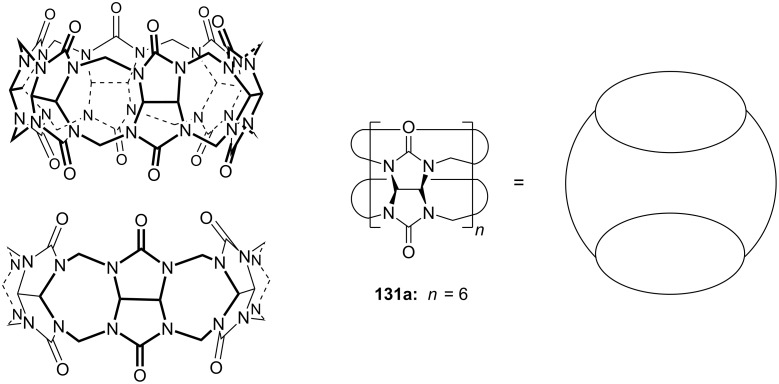
Structure and schematic of cucurbit[6]uril (CB[6], **131a**).

Cucurbit[6]uril (CB[6], **131**), a macrocycle comprising six glycoluril units connected by 12 methylene bridges (Figure 96), is the oldest and most frequenly encountered member of the host family cucurbit[*n*]uril (CB[*n*], *n* = 5–11) [400–404].

Crystalline complexes incorporating various metal salts and some dyes were observed and consequently cucurbiturils were investigated as receptors by Mock and Shih [405]. Alkylammonium ions were the first organic guests to be reported for CB[6] (**131a**) [406]. Mock [407], Buschmann and co-workers [408–409], and Kim et al. [410] further investigated the molecular-recognition properties. Cucurbiturils bind their guests by hydrogen-bonding or ion-dipole interactions in combination with the hydrophobic effect of the cavity. The rigidity of the structure enables selective recognition of hydrophobic residues or cations. The selectivity strongly depends on the inner size of the cavity and possible guest orientations therein, as in cyclodextrins and calixarenes: *para*-methylbenzylamine is bound, while the *ortho*- and *meta*-isomers are not [411]. Isaacs et al. published a crystal structure of the cucurbit[6]uril *p*-xylylenediammonium inclusion complex. The ammonium cations are symmetrically located in the centre of a ring formed by the carbonyl oxygens. The benzene ring is rotationally disordered in the cavity between two orientations [412].

The upper and the lower regions of the cucurbituril – the occuli – bear at least six urea carbonyl groups, representing an area of negative charge accumulation, co-ordinating to cationic species such as alkanediamines. The high specificity for ammonium ions is explained mainly by this electrostatic ion-dipole attraction assisted by hydrogen bonding. Proper alignment of the bound ammonium ions with the host carbonyl dipoles is critical: In the homologous series of *n*-alkane amines a clear trend in stability of the complexes was observed, reaching the maximum for *n*-butylamine: *n* = 1 < 2 < 3 < 4 > 5 > 6 > 7. α,ω-Alkanediammonium ions (H_3_N^+^–(CH_2_)*_n_*–NH_3_^+^) are bound by CB[6] (**131a**) with a preference for an alkyl chain length of *n* = 5 or 6. Substituents fitting the size of the cavity are bound with the highest strength and affinity; longer chains protrude into the second oculus of the cucurbituril, interfering with the carbonyl dipoles and their solvation sphere [413–414].

In contrast to the moderate to good water soluble related host molecules with a comparable cavity size, the cyclodextrins (**136**) [55,223,415–416], the poor solubility of CB[6] (**131a**) in common solvents and water makes it difficult to study its host–guest chemistry in solution.

During the 1990s it was discovered that CB[6] is readily soluble in aqueous solutions containing alkali or alkaline earth metal ions. Since then, such aqueous solutions have often been employed for studies on complexation properties of CB[6] (**131a**) [417–418]. Mock and Shih examined its binding affinity towards a variety of aliphatic ammonium ions in 50% (v/v) aqueous formic acid and determined binding constants (*K*) of around 10^3^–10^4^ M^−1^ for *n*-alkylammonium ions and 10^4^–10^5^ M^−1^ for α,ω-alkanediammonium ions from NMR and/or UV spectroscopy measurements [413]. Typical binding constants for ammonium guests, e.g. simple amines, diamines and aromatic amines range from 10^1^ to 10^7^ M^−1^ in H_2_O/HCOOH mixture [419]. In aqueous salt solutions, for example, 50 mM sodium chloride solution, even higher values for α,ω-alkanediammonium ions (up to 1.5 × 10^9^ M^−1^ for H_3_N^+^–(CH_2_)_5_–NH_3_^+^, cadaverin) have been reported [420].

Not only simple amines, but also many amino acids and amino alcohols have been employed as guests. Buschmann and co-workers first studied the complex formation between cucurbituril and some aliphatic amino acids by means of calorimetric titrations in aqueous formic acid (50% v/v) or aqueous solution for comparison of the interaction of cucurbituril with some aliphatic amino alcohols and aliphatic amino compounds: The complex formation of amino acids was found to be favored by enthalpic and entropic contributions. The situation changes completely in the case of amino alcohols. Reaction enthalpies and entropies are influenced by the number of methylene groups: 3-aminopropanol formed the most stable complex. With an increasing number of methylene groups the stability of the complexes decreased, which is attributed to entropic factors [421].

Paraquat and its derivatives are typical guests for cucurbit[*n*]urils [422–427]. Amino azabenzenes are bound with binding strengths in the range of 10^3^–10^6^ M^−1^ [428]. Many homologues from cucurbit[5]uril to cucurbit[10]uril, as well as derivatives, congeners and analogues are available, even exceeding the cavity size span of the cyclodextrin family. Their chemistry has been discussed in several books [429–431] and reviews [420,432–436]. In the following part, some recent examples of the molecular recognition of ammonium ions will be discussed.

Various cucurbit[*n*]uril derivatives have been synthesized by introducing alkyl groups at the equator of the molecules to improve their solubility in water and other commonly used organic solvents [437–440]. Different reactive functional groups have been introduced directly onto the surface of the cucurbit[*n*]urils to improve their solubility, and for further modification [411,441–442].

**Figure 97:**
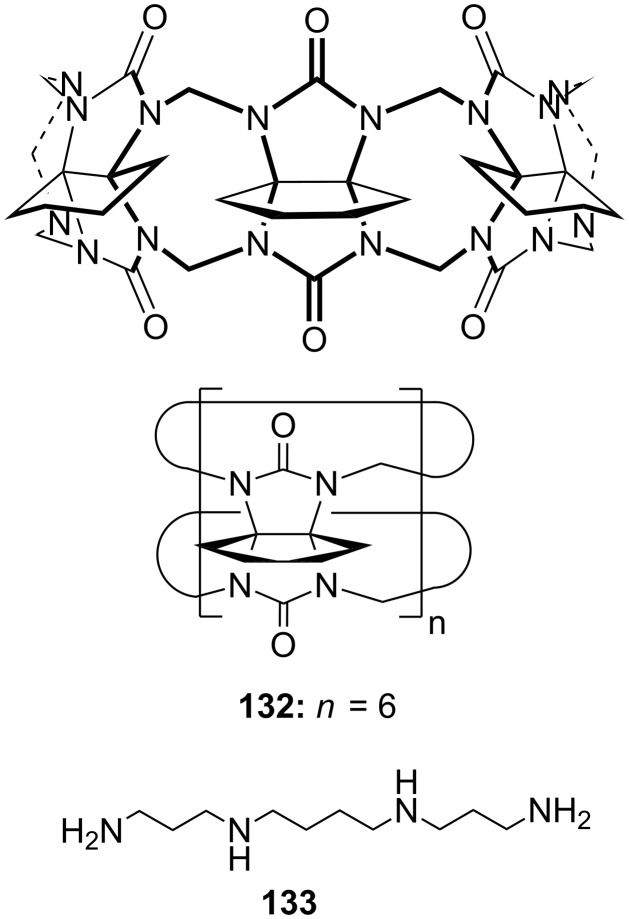
Cyclohexanocucurbit[6]uril (CB′[6], **132**) and the guest molecule spermine (**133**).

Such a water soluble example was reported with cyclohexanocucurbit[6]uril (CB × [6], **132**) (Figure 97). Complexation properties with various organic mono- and diammonium ions were studied by isothermal titration calorimetry and ^1^H NMR spectroscopy [443]. X-ray crystal structures of α,ω-alkanediammonium ions (H_3_N^+^–(CH_2_)*_n_*–NH_3_^+^, *n* = 4–8) and spermine (**133**) complexes with **132** revealed that the aliphatic chains of the guest molecules were in an extended or partially bent conformation in the cavity, depending on their length. The hexamethylene chain conformation is twisted to allow strong ion–dipole interactions between both ammonium groups and the carbonyl groups at the portals. This increases the hydrophobic interactions between the alkyl part of the guest and the inner wall of the host, which results in the largest enthalpic gain and a preference for this guest among all α,ω-alkanediammonium ions. The selectivities match with those of **131a.** The cavity dimensions are essentially the same as in CB[6] (**131a**). The binding affinities of CB′[6] (**132**) towards *n*-alkylammonium ions (10^4^–10^8^ M^−1^) and α,ω-alkanediammonium ions (10^7^–10^10^ M^−1^) in water are 3–5 and 2–3 orders of magnitude higher than those of CB[6] in 50% formic acid [407,416–417] and in 0.05 m NaCl solution [420], respectively. This was attributed mainly to the larger enthalpic gain upon complex formation in the absence of interfering ions, such as protons and Na^+^. In particular, the binding constant of spermine to CB × [6] was found to be 3.4 × 10^12^ M^−1^, which is the highest binding constant ever reported for CB[6] or its derivatives.

**Figure 98:**
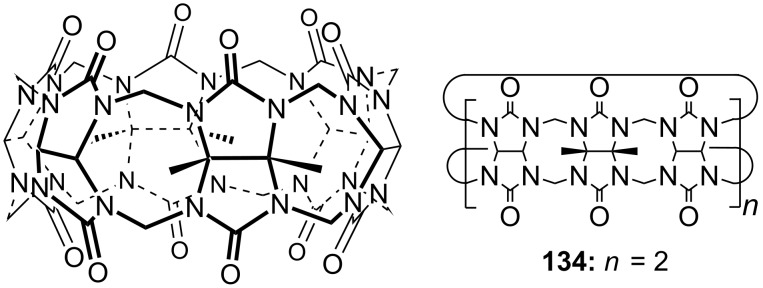
α,α,δ,δ-Tetramethylcucurbit[6]uril (**134**).

Cucurbit[*n*]urils strongly bind amino acids. A crystal structure of the inclusion complex of *S*-glutamate (*S*-Glu) in α,α,δ,δ-tetramethylcucurbit[6]uril (**134**) (Figure 98), captured by a host in a 1:1 host:guest ratio, gives more insight [444]. The protonated amino moiety is located at the portal of the host whilst the side chain carboxyl anion moiety is included in the cavity of **134**. A combination of hydrogen bonding and ion–dipole interactions of the ammonium group and the portal carbonyls of the host were seen as the driving force for the complex formation. In addition, the carboxyl moiety of the amino acid located at the portal of the host could interact with the portal carbonyl of the host through hydrogen bonding.

**Figure 99:**
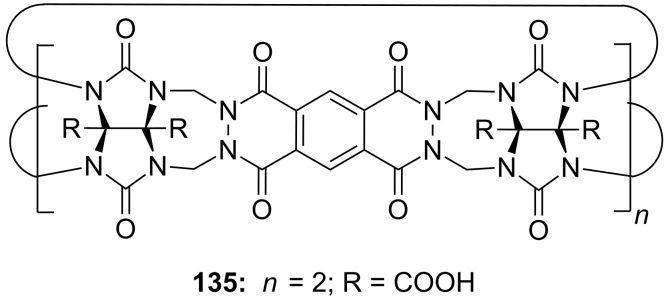
Structure of the cucurbituril-phthalhydrazide analogue **135**.

Unsubstituted cucurbiturils are not fluorescent. Issacs and co-workers described the incorporation of a fluorescent (bis)-phthalhydrazide in cucurbit[6]uril (Figure 99), which made the system accessible to monitoring by fluorescence spectroscopy [442]. This analogue (**135**) shows good molecular recognition properties for a variety of guests in aqueous sodium acetate buffer at pH 4.74: Association constants for α,ω-alkanediammonium ions (H_3_N^+^–(CH_2_)*_n_*–NH_3_^+^, *n* = 6 to 12) increase with the length of the alkane chain. The maximum binding strength was observed for *n* = 10 and 11 with 2.3 × 10^4^ M^−1^. Aromatic ammonium targets were complexed even stronger due to the additional π–π-interactions. The best examples were benzidine with an association constant of 4.6 × 10^6^ M^−1^, nile red [445] with 8.2 × 10^6^ M^−1^ and the similar dye nile blue chloride with 1.1 × 10^6^ M^−1^. The authors argue, that increasing the surface area for π–π-interactions by increasing the size of the π-system of the guest as well as increasing the co-planarity of the guest molecule significantly increases the association constant. Biologically relevant guests such as amino acids and nucleobases were bound in the cavity of **135** with *K*_ass_ values ranging from 10^3^ to 10^6^ M^−1^. Similarly, good affinities to aromatic amino acids as a result of π–π-stacking and ion-dipole interactions were observed: For *S*-phenylalanine (**81a**), *S*-tyrosine and *S*-tryptophan (**81b**) association constants of 4.2 × 10^4^, 5.7 × 10^4^ and 3.2 × 10^6^ M^−1^, respectively were determined. Due to the larger size of the indole ring compared to that of the monocyclic systems, tryptophan (**81b**) was bound more tightly.

**Figure 100:**
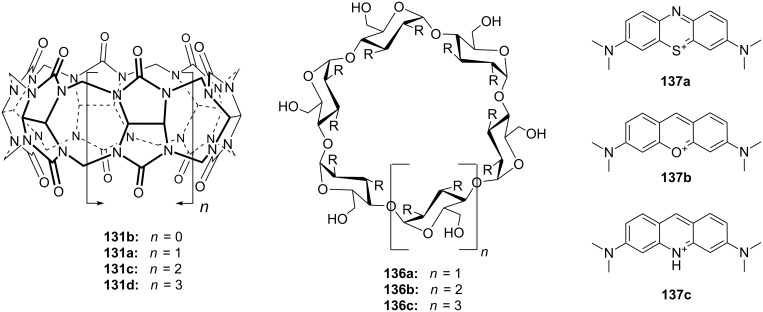
Organic cavities for the displacement assay for amine differentiation.

A dual-response colorimetric sensor array based on supramolecular host–guest complexation in cyclodextrins (α-, β- and γ-cyclodextrin, **136**) and cucurbit[*n*]urils (CB, *n* = 5–8, **131**) was used for the identification of amines in water [446] (Figure 100). The displacement of colored or fluorescent dyes such as methylene blue (**137a**), pyronine (**137b**) and acridine orange (**137c**) led to discrimination among primary, secondary, tertiary, aliphatic, aromatic, linear and branched amines by color change or by the increase in fluorescence. The combination of the images obtained from visible and UV light identified each of the 14 analytes investigated. The selectivity of the sensor array is based on the analyte’s interaction with the host–guest complex, which involves the combination of a large number of parameters, including hydrophilicity–hydrophobicity, coulombic effects, dipolar interactions and hydrogen bonding.

Nau and co-workers introduced a general supramolecular assay principle in which amino acid decarboxylase activity can be continuously monitored by measuring changes in fluorescence, which results from the competition of the enzymatic product and the dye for forming a complex with a cucurbit[*n*]uril macrocycle [447].

**Figure 101:**
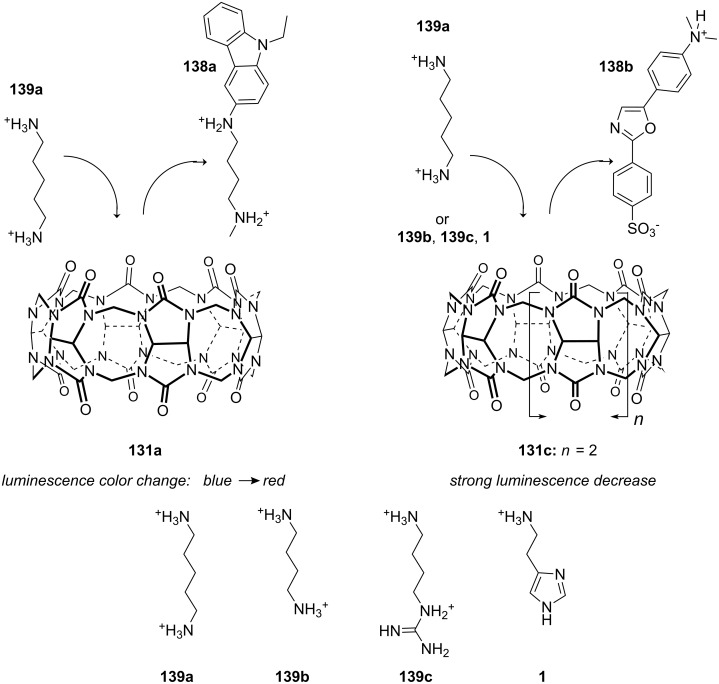
Displacement assay methodology for diammonium- and related guests involving cucurbiturils and some guests.

The combination of cucurbit[6]uril (**131a**) and the 3-amino-9-ethylcarbazole dye **138a** leads to a suitable displacement assay (Figure 101) for monitoring the enzymatic activity of lysine decarboxylase in aqueous buffer at pH 7 [448]. Due to a complexation-induced p*K*_a_ shift, a large dual fluorescence response (100-fold increase at 375 nm and 9-fold decrease at 458 nm) accompanied by a color change upon supramolecular encapsulation in cucurbit[6]uril (**131a**) is observed. The enzymatic decarboxylation of lysine (**81c**) converts the amino acid *S*-lysine (**81c**) into cadaverine (**139a**), which competes very efficiently (*K*_ass_ = 9.5 × 10^9^ M^−1^ in 10 mM NH_4_OAc buffer) and so fully reverts the fluorescence changes originally caused by the addition of the macrocycle. The binding constant of the substrate lysine (**81c**) is too low to displace the more strongly bound fluorescent dye (*K*_ass_ = 2.22 × 10^7^ M^−1^) and causes no effect.

This principle was employed in a similar manner with cucurbit[7]uril (**131c**) and the fluorescent dye Dapoxyl (**138b**) (Figure 101). It forms a strong inclusion complex with **131c** (*K*_ass_ = (2.0 ± 0.2) × 10^4^ M^−1^) in ammonium acetate buffer at pH 6, which shows up to 200 times higher emission intensity (λ_em_ = 380 nm) than the free dye [449]. Addition of amino acids has little effect on the fluorescence intensity of the CB7-Dapoxyl reporter pair. Addition of low-micromolar concentrations of amines lead to a steep decrease in fluorescence as a result of competitive binding. This allows real-time monitoring of enzymatic activity by a switch-off fluorescence response in 10 mM NH_4_OAc buffer at pH 6.0.

As demonstrated by simple titration experiments, the substrates lysine (**81c**), arginine (**81d**), histidine (**81e**) and ornithine have low affinity to **131c**, and cannot interfere with the formation of the strongly fluorescent complex (*K*_ass_ < 10^3^ M^−1^). Decarboxylation produces the corresponding amines cadaverine (**139a**), agmatine (**139c**), histamine (**1**) or putrescine (**139b**), so increases the net positive charge and thereby the affinity of the competitor by removal of the carboxylate group. These guests exist in their ammonium ion forms near neutral pH and thus have a very high affinity for **131c** (*K*_ass_ < 4.3 × 10^4^ M^−1^). This tandem assay principle has millimolar sensitivity.

The versatile approach was extended to aromatic guests and applied for enantiodiscrimination, respectively resolution [450]. Similar observations were published: The amino acids histidine (**81e**), tyrosine and tryptophan (**81b**) bind to the reporter pair **131c/138b** with approx. 1000 M^−1^, the diamines in contrast with 10^4^ to 10^6^ M^−1^ affinity in 10 mM NH_4_OAc buffer solutions (pH 6.0) and, therefore, displace the dye from the complex.

Time-dependent fluorescence response monitoring of *S*-lysine decarboxylation with varying *S*-lysine enantiomeric excess allowed accurate determination of optical purity of the amino acid over a wide range of *ee* (64–99.98%) by different kinetic fluorescence decay traces with a 2.4 nmol limiting sensitivity. Only the *S*-enantiomer is accepted by the enzyme as a substrate and is converted to the product that is responsible for the observed fluorescence signal. No response and no conversion by the enzyme are observed with the *R*-enantiomer.

**Figure 102:**
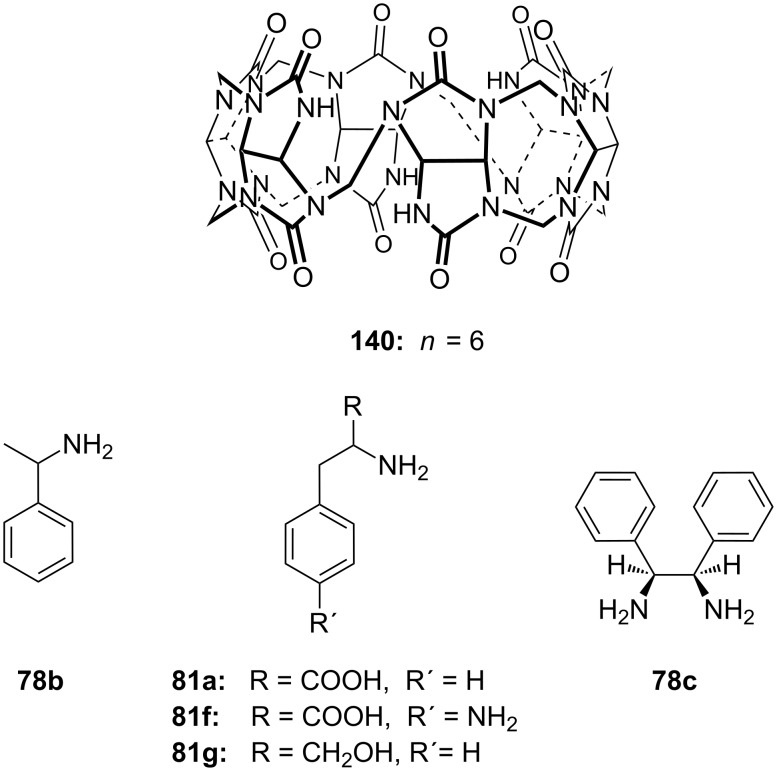
Nor-seco-Cucurbituril (±)-bis-ns-CB[6] (**140**) and guest molecules.

Recently, Isaacs et al. demonstrated the chiral recognition of some amino acids inside a novel chiral cucurbituril: nor-seco-cucurbituril (±)-bis-ns-CB[6] (**140**, Figure 102), which demonstrates enantio- and diastereoselective recognition inside its cavity [451].

The *K*_ass_ values for **140** towards diammonium guests were measured by UV–vis spectroscopic titration and ^1^H NMR spectroscopy competition experiments in water with association values mainly in the range of 10^3^ to 10^4^ M^−1^. The affinity of (±)-bis-ns-CB[6] toward 1,6-diaminohexane in its protonated form was even higher (1.3 × 10^5^ M^−1^). Conversely, this affinity is 3400-fold lower than found with CB[6] (**131a**), which presumably arises from differences in the strength of ion–dipole interactions, the degree of aqueous solvation of the C=O portals, or both.

Host **140** undergoes diastereoselective complexation (up to 88:12) with chiral amines including amino acids and amino alcohols as well as with meso-diamine **141e**. In the ^1^H NMR spectra recorded for a mixture of (±)-bis-ns-CB[6] and excess of the guest (−)-**78b**, a 72:28 ratio of the diastereomer was found. Toward amino acids **81f** (77:23) and **81a** (88:12) and amino alcohol **81g** (76:24) minimal higher values were observed. Interestingly, (±)-bis-ns-CB[6] is even able to distinguish between the enantiotopic groups of meso-compound **78c** (74:26).

**Figure 103:**
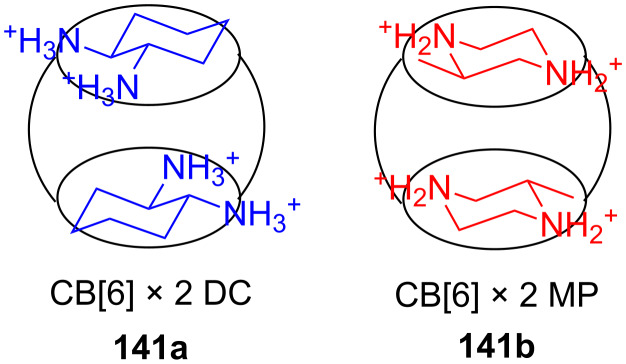
The cucurbit[6]uril based complexes **141** for chiral discrimination.

A combination of achiral host cucurbiturils and chiral inductor can also serve as a supramolecular chiral host (Figure 103). A chiral guest added to the solution of cucurbit[6]uril-based complexes with enantiopure amines can replace one of the originally bound amines achieving an enantiodifferentiation by accommodating two different chiral guests inside a self assembled achiral capsule. In this way significant enantiomeric and diastereomeric discrimination by incorporating a strong chiral binder is possible [452]. Comprehensive studies on the chiral recognition of guests were performed: Dissolving cucurbit[6]uril (CB[6]) in an aqueous solution of an enantiopure organic amine, such as (*R*)- or (*S*)-2-methylpiperazine (MP) or (*R*,*R*)- or (*S*,*S*)-*trans*-1,2-diaminocyclohexane (DC), led to the formation of the respective enantiopure complex, i.e., (*R*;*R*)- or (*S*;*S*)-[CB-[6] × 2MP]^4+^ (**141b**) or (*R*,*R*;*R*,*R*)- or (*S*,*S*;*S*,*S*)-[CB[6] × 2DC]^4+^ (**141a**). (*S*)-2-Methylbutylamine could be discriminated by these assemblies with up to 95% *ee* by formation of diastereomeric (*S*;*R*)- and (*S*;*S*)-[CB[6] × MP × MB]^3+^ ternary complexes. (*S*)-MB controls the degree of chiral supramolecular assembling of (*R*)-MP or (*S*)-MP with cucurbit[6]uril:

[CB[6] × 2((*R*)-MP)]^4+^ + (*S*)-MB^+^ → [CB[6] × (*R*)-MP × ((*S*)-MB)]^3+^ + (*R*)-MP^2+^

with a *K*_ass_ of 15000 ± 3000 M^−1^ for this process

[CB[6] × 2((*S*)-MP)]^4+^ + (*S*)-MB^+^ → [CB[6] × (*S*)-MP × ((*S*)-MB)]^3+^ + (*S*)-MP^2+^

with a *K*_ass_ of 800 ± 100 M^−1^ for this process.

The authors also found cucurbit[7]uril (**131c**) binding the diastereomeric dipeptide *S*-Phe-*S*-Leu-NH_3_^+^ up to eight times tighter than *S*-Phe-*R*-Leu-NH_3_^+^ with its larger cavity. The discrimination of dipeptides was not possible with the previously discussed system.

The cavity size of cucurbit[7]uril enables the molecule to bind ferrocenyl and adamantyl substituted amines strongly as 1:1 complexes: Rimantadin, an amino adamantyl derivative, which is used as an anti-viral drug, is included in aqueous buffer at pD 4.74 with an association constant of around 4.2 × 10^12^ M^−1^ [453].

**Figure 104:**
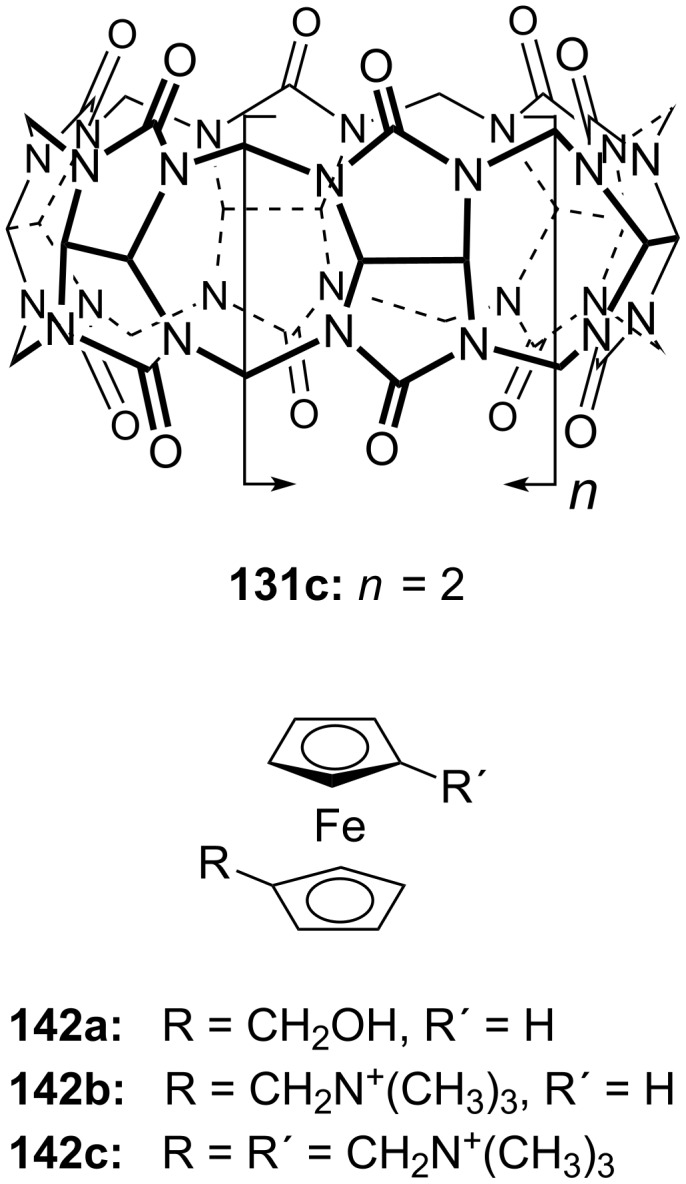
Cucurbit[7]uril (**131c**) and its ferrocene guests (**142**) opposed.

The molecular host cucurbit[7]uril (**131c**) forms an extremely stable inclusion complex with the dicationic ferrocene derivative bis(trimethylammoniumethyl)ferrocene (**142c**) in aqueous solution [454] (Figure 104). The equilibrium association constant for this host–guest pair is 3 × 10^15^ M^−1^, equivalent to that exhibited by the avidin–biotin pair.

The large association strength has been determined from serial competitive ITC binding studies (Table 8). Two different series, also giving *K*_ass_ values for other interesting ammonium guests, were pursued. All amines were protonated under the conditions of the study.

**Table 8:** Two series of binding constants for different guests to CB[7] (**131c**).

Guest	Competitor	*K*_ass_/M^−1^

*S*-Phe (**81a**)	none	(1.8 ± 0.2) × 10^6^
1,6-hexanediamine	*S*-Phe (**81a**)	(2.1 ± 0.4) × 10^9^
aminocyclohexane	1,6-hexanediamine	(1.3 ± 0.4) × 10^11^
**142c**	aminocyclohexane	(3.0 ± 1.0) × 10^15^
cyclopentanone	none	(4.2 ± 0.3) × 10^5^
spermine (**133**)	cyclopentanone	(4.8 ± 0.6) × 10^8^
*N,N*′-bis(aminoethyl)-1,6-hexanediamine	spermine (**133**)	(1.7 ± 0.4) × 10^11^
**142c**	*N,N*’-bis(aminoethyl)-1,6-hexanediamine	(3.3 ± 1.0) × 10^15^

The values for **142a** and **142b** are (3.2 ± 0.5) × 10^9^ M^−1^ and (4.1 ± 1.0) × 10^12^ M^−1^, respectively. A significant loss in the complex stability by a factor of 1400 in the *K*_ass_ value is observed upon oxidation of the ferrocene centre of **142c**, enabling a switching process of complexation/decomplexation dependent on the competitor.

The extremely large affinities of the complexes surveyed are due to a large enthalpic gain, originating from the tight fit of the ferrocene core to the rigid CB[7] cavity achieving optimal van der Waals contacts, critically assisted by the entropic gain arising from the extensive host desolvation, and largely uncompensated by losses in configurational entropy. The crystal structure of the complex shows the complete inclusion of the ferrocenyl residue in the CB[7] cavity and the almost ideal positioning of each of the trimethylammonium groups maximizing ion–dipole interactions with the carbonyl rims on each of the host portals. The ferrocene core of the guest fills 55% of the host cavity volume, approximately equal to the optimal filling fraction proposed [455].

Quaternary cations such as NMe_4_^+^, NEt_4_^+^, PMe_4_^+^, and PEt_4_^+^ are encapsulated within the cavity of CB[7] (**131c**) (Figure 104), with *K*_ass_ = (1.2 ± 0.4) × 10^5^, (1.0 ± 0.2) × 10^6^, (2.2 ± 0.4) × 10^6^, and (1.3 ± 0.3) × 10^5^ M^−1^, respectively [456].

**Figure 105:**
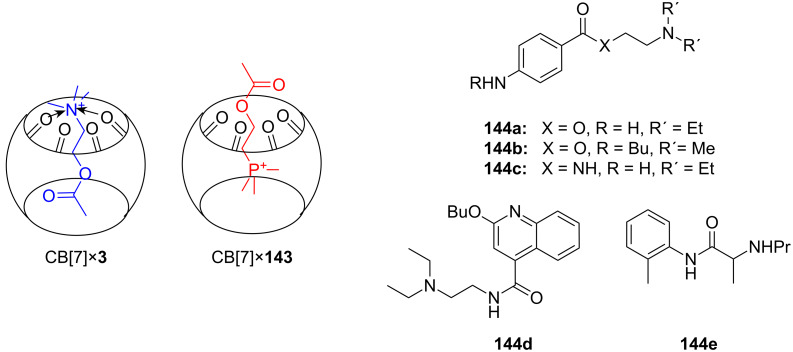
Cucurbit[7]uril (**131c**) guest inclusion and representative guests.

Consistent with these values, acetylcholine (**3**) and other cationic cholines (R_3_NCH_2_CH_2_OR′^+^), their phosphonium analogues (R_3_PCH_2_CH_2_OR′^+^) (R_3_ = Me_3_, Et_3_, or Me_2_Bz, or R_3_N = quinuclidinium, and R′ = H, COCH_3_, CO(CH_2_)_2_CH_3_, or PO_3_H) and (±)-carnithine (**77a**) form stable 1:1 host–guest complexes with cucurbit[7]uril (**131c**) in aqueous solution (*K*_ass_ in the order of magnitude 10^5^–10^6^ M^−1^) [457]. The complexation behavior has been investigated using ^1^H and ^31^P NMR spectroscopy and ESI mass spectrometry. This study is one rare example, where molecular recognition of cholines in aqueous solution is achieved with a neutral host without aromatic walls for cation–π-interactions. The acetyl-substituent is included in the cavity and the quaternary ammonium ion is co-ordinated by the carbonyl functions of **131c**. In the case of phosphonium groups, these substituents are generally included in the cavity additionally stabilized by van der Waals contacts. The acetyl substituent sits on the outside of the cavity (Figure 105).

The cucurbit[7]uril (**131c**) host molecule forms also very stable host–guest complexes with the local anaesthetics procaine (**144a**, *K*_ass_ = (3.5 ± 0.7) × 10^4^ dm^3^ mol^−1^), tetracaine (**144b**, *K*_ass_ = (1.5 ± 0.4) × 10^4^ dm^3^ mol^−1^), procainamide (**144c**, *K*_ass_ = (7.8 ± 1.6) × 10^4^ dm^3^mol^−1^), dibucaine (**144d**, *K*_ass_ = (1.8 ± 0.4) × 10^5^ dm^3^ mol^−1^) and prilocaine (**144e**, *K*_ass_ = (2.6 ± 0.6) × 10^4^ dm^3^ mol^−1^) in aqueous solution (pD = 4.75) (Figure 105) as observed by NMR studies [458]. The stability constants are 2–3 orders of magnitude higher than the values reported for binding by the comparably sized β-cyclodextrin (**136b**) host molecule. The protonated forms are bound more strongly in acidic solution. Upon protonation the cucurbit[7]uril sits around the aromatic unit of **144a**–**144c**, in the deprotonated case it includes the alkylated amine centre.

Similarly, “bolaform” guests with two cationic end groups, such as succinylcholine chloride (**145**) and α,ω-bis(trialkylammonium)alkane dications (or their phosphonium analogues) form strong host–guest complexes and [2]pseudorotaxanes with cucurbit[7]uril [459]. An analogous dimeric guest series to the amines discussed previously containing NMe_3_^+^, NEt_3_^+^, quinuclidinium (**146g**), PMe_3_^+^ and PEt_3_^+^ endgroups, was studied in aqueous solution by ^1^H and ^31^P NMR spectroscopy, as well as ESI mass spectrometry [460].

**Figure 106:**
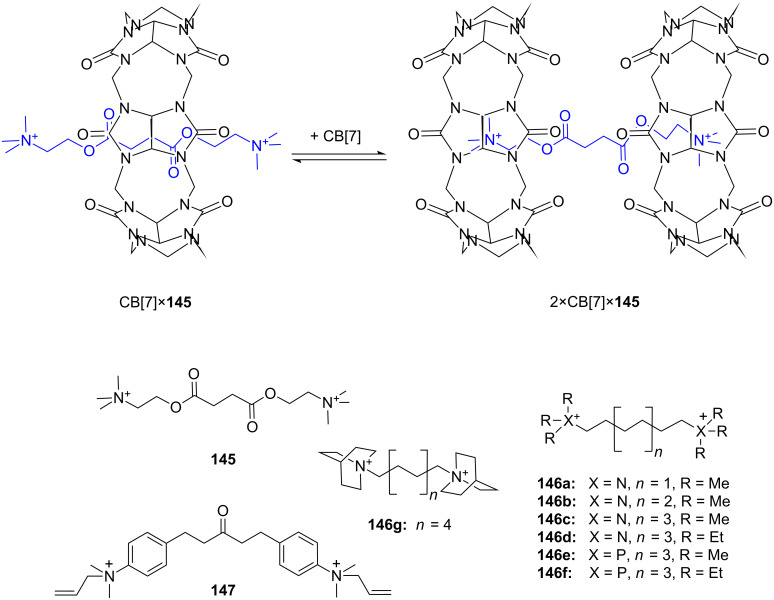
Cucurbit[7]uril (**131c**) binding to succinylcholine (**145**) and different bis-ammonium and bis-phosphonium guests.

The formation of 1:1 aggregates is assigned to a [2]pseudorotaxane structure with the NMe_3_^+^ and NEt_3_^+^ end groups outside the cavity near the carbonyl oxygens on the portal and the guest molecule located in the hydrophobic cavity (Figure 106). The 1:1 host–guest stability constants range from 8 × 10^6^ (guest **145**) to 3 × 10^10^ M^−1^ (guest **146b**) and are dependent on the nature of the end group and the length and hydrophobicity of the central linker. The stability constants for the 1:1 complexes with guests with the same decamethylene linker follows the order **146c** > **146e** > **146f** > **146d**, indicating that for threads with the same alkyl chain length, the stability constant is related to charge diffusion on the peralkylonium end group. Changing the end groups from NMe_3_^+^ to NEt_3_^+^ (**146c** to **146d**) or PMe_3_^+^ to PEt_3_^+^ (**146e** to **146f**) results in a lowering of *K*_ass_ value by one order of magnitude as the less polar triethyl-substituted groups have weaker ion-dipole interactions with the polar portals of CB[7] than the methyl analogues.

With the exceptions of the shorter [(CH_3_)_3_N(CH_2_)*_n_*N(CH_3_)_3_]^2+^ (*n* = 6, 8) dications, the addition of a second CB[7] results in the translocation of the first CB[7], such that the hydrophobic −NR_3_^+^ and −PR_3_^+^ end groups (R = Me or Et) are encapsulated in the cavities, while the central linker extends through the CB[7] portals (Figure 106). The magnitude of the stability constants for the 2:1 complexes closely follows the trend observed previously for CB[7] binding with the NR_4_^+^ and PR_4_^+^ cations.

The vast majority of host–guest complexes of CB[7] (**131c**) with cationic guests, such as paraquat [461–462], assemble with the cationic part of the guest located outside of the cavity, adjacent to the oxygens of the portal carbonyls. The remaining hydrophobic region of the guest is positioned inside the cavity.

Mohanty and co-workers have found that the fluorescent dye thioflavin T, used extensively to probe the presence of amyloid fibrils, forms 1:1 and 2:1 host–guest complexes with cucurbit[7]uril (**131c**), with binding constants in the order of magnitude of 10^5^ and 10^3^ M^−1^, respectively [463].

**Figure 107:**
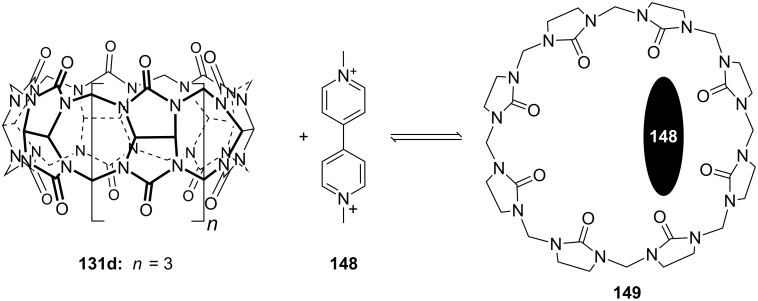
Paraquat-cucurbit[8]uril complex **149**.

By enlarging the host by one glyconuril unit to cucurbit[8]uril (**131d**) (Figure 107) a cavity comparable in size with γ-cyclodextrin (**136c**) results, which is in the position to capture and include even other macrocycles like cyclene (**6**) or cyclam (**7**) and their complexes with transition metals [464].

Kim and co-workers report that **131d** can bind to aromatic guests, such as tryptophan (**81b**), tyrosine, and dopamine (**2**) as observed by the resulting changes in visible color and in their NMR spectra [433,465].

In the crystal structures of the inclusion complexes of *S*-tyrosine (*S*-Tyr), *S*-histidine (**81e**, *S*-His), *S*-leucine (*S*-Leu) in cucurbit[8]uril (**131d**) a 1:2 host:guest ratio was found [444]. It is common, that the ammonium moiety is always located at the portal of the host, co-ordinated by hydrogen bonding and ion–dipole interaction with the carbonyl groups of the host. The host can include not only the stacked aromatic moieties, but also the alkyl moieties of the amino acids.

Consistent with these observations, cucurbit[8]uril (**131d**) is known to form 1:1:1 heteroternary complexes with paraquat (**148**) and a second aromatic guest (Figure 107): Urbach et al. describe the molecular recognition of amino acids by cucurbit[8]uril and its complex with 1,1′-dimethyl-4,4′-bipyridinium (paraquat, **148**). A comprehensive examination of the 20 genetically encoded amino acids was carried out by ^1^H NMR spectroscopy and isothermal titration calorimetry in aqueous solution [466]. The amino acid to host stoichiometry is controlled by the presence (1:1) or absence (2:1) of paraquat (**148**). Both **131d** and the complex **149** bind measurably to only tryptophan (**81b**), phenylalanine (**81a**) and tyrosine. For the 1:1 complexes with the cucurbit[8]uril-paraquat-assembly (**149**) a selectivity of Trp (**81b**, *K*_ass_ = 4.3 × 10^4^ M^−1^) with 8-fold and 19-fold specificity over Phe (**81a**, *K*_ass_ = 5.3 × 10^3^ M^−1^) and Tyr (*K*_ass_ = 2.2 × 10^3^ M^−1^), respectively, was found. The binding strengths for the 2:1 complexes of cucurbit[8]uril reach 10^8^ M^−1^ (Trp, *K*_ass_ = 6.9 × 10^7^ M^−1^ and Phe, *K*_ass_ = 1.1 × 10^8^ M^−1^).

The interaction of the host system with tryptophan (**81b**) was investigated in greater detail by using a combination of isothermal titration calorimetry, mass spectrometry, UV–visible, fluorescence, and ^1^H NMR spectroscopy methods [467], with the finding that the selectivity is mediated by the electrostatic charge in aqueous solution.

The ITC data showed that **149** binds Trp guests with ammonium group like Trp-OMe and tryptamine (*K*_ass_ ~ 5 × 10^4^ M^−1^) with approximately 20-fold selectivity over guests lacking this functionality, such as *N*-acetyl-Trp (*K*_ass_ = 2–3 × 10^3^ M^−1^). For the binding of Trp (**81b**) and its derivatives, a 1:1 binding stoichiometry was observed in all experiments. *N*-Terminal tryptophan residues are bound with higher affinity than C-terminal or internal tryptophan residues. The complex binds Trp-Gly-Gly with high affinity (*K*_ass_ = 1.3 × 10^5^ M^−1^, log *K*_ass_ = 5.1), with 6-fold specificity over Gly-Trp-Gly (log *K*_ass_ = 4.3), and with 40-fold specificity over Gly-Gly-Trp (log *K*_ass_ = 3.5).

In addition, cucurbit[8]uril (**131d**) was reported to be a remarkably synthetic host for selective recognition and non-covalent dimerization of *N*-terminal aromatic peptides in aqueous solution [468]. Cucurbiturils are known to recognize *N*-terminal tryptophan over internal and C-terminal sequence isomers. Tripeptides of the sequence X-Gly-Gly, Gly-X-Gly, and Gly-Gly-X with X being Trp, Phe, Tyr and His were studied. Compound **131d** selectively binds and dimerizes Trp-Gly-Gly and Phe-Gly-Gly with high affinity (ternary complex association constant in the range of 10^9^–10^11^ M^−1^), the binding constants for the other 10 peptides were too small to be measured by ITC. Both peptides are bound in a stepwise manner, the latter with positive co-operativity. The crystal structures revealed the structural basis for selective recognition as the inclusion of the hydrophobic aromatic side chain and chelation of the proximal *N*-terminal ammonium group by carbonyl oxygens on the cucurbituril. In view of application the authors pointed out the potential study of dimer-mediated biochemical processes and the potential use for the separation of peptides and proteins.

**Figure 108:**
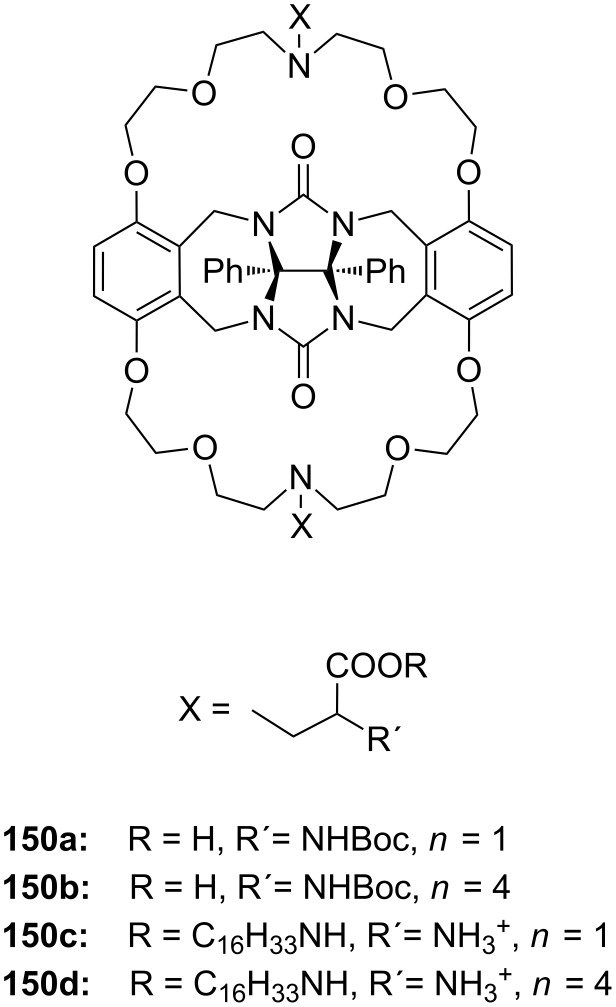
Gluconuril-based ammonium receptors **150**.

Nolte and Escuder published a series of cucurbituril related molecules, amino acid appended diphenylglycouril-based chiral molecular receptors (**150**) [469] (Figure 108). The binding of several biologically relevant guests with aromatic moieties was studied with UV–vis spectroscopy in competition experiments with 4-(4-nitrophenylazo)resorcinol (“Magneson”) and 2-(4-hydroxyphenylazo)benzoic acid (HABA) in water at pH 8 and 4.5, respectively. Compound **150b** forms thin tubules in chloroform and vesicles in water, with the possibility of surrounding the guest. Aggregates of the chiral host **150b** bind catecholamines and aromatic amino acids in water and are able to discriminate between their enantiomers. The calculated binding constants were moderate to high and a remarkable enantioselectivity for the corresponding enantiomers of *R*-tyrosine (1.6 × 10^4^ M^−1^ vs. 2 × 10^3^ M^−1^), *S*-phenylalanine (**81a**, 2.6 × 10^4^ M^−1^ vs. 1.2 × 10^4^ M^−1^) and *R*-tryptophan (**81b**, 5.6 × 10^4^ M^−1^ vs. 1.7 × 10^4^ M^−1^) was observed.

The rigid structure and capability of forming stable complexes with a wide range of molecules and ions, mediated by ammonium ion co-ordination in combination with inclusion of the side chains make cucurbit[*n*]urils very attractive not only as a synthetic receptor. As previously stated, self assembly systems is outwith the scope of this review, but it has to be mentioned since nearly as many papers as published for molecular recognition with cucurbit[*n*]urils are found using the macrocycles as building blocks for the construction of supramolecular architectures, often relying on the interaction with an ammonium species. The interested reader is referred to the large body of recent literature [470–485].

In summary, cucurbiturils and their derivatives are valuable and versatile hosts for ammonium and diammonium guests, as well as amino acids and peptides, reaching the highest binding constants of all presented receptor families in highly competitive media (up to 10^10^ to 10^12^ M^−1^). Generally, the ammonium guests are co-ordinated by the carbonyl groups of the host by electrostatic ion–dipole attraction assisted by hydrogen bonding. The non-polar part of the guest is included in the cavity. The binding is governed by hydrophobic effects and van der Waals contacts. The entropic gain upon binding additionally supports the high association constants found with cucurbiturils. Similar facts are also relevant to quaternary ammonium species, which are bound by the same interactions. Notably, cucurbit[*n*]urils are one example, where these guests are not bound by cation–π-interactions. Here the area of negative charge accumulation, represented by the carbonyl groups, co-ordinates cationic species strongly. For a more comprehensive discussion of the binding properties of the cucurbit[*n*]uril family, we recommend the recent review article by Issacs et al. [436], thermodynamic aspects of the binding process are discussed in detail in recent overviews [429,432].

### 5. Molecular clefts, tweezers, trigonal ligands, phosphonates and cyclophane structures as receptors for ammonium ions

Typical host structures for ammonium guests are macrocyclic, like calixarenes, cyclodextrins or cucurbiturils with polar functionalities organized in a circular manner. However, many suitable synthetic receptors fall in a second category: Non-cyclic compounds, with more open structures. These hosts have pockets or cavities into which a guest can fit, but is not completely encapsulated. These clefts, clips and tweezers are discussed in the following section together with tripods and suitably functionalized cyclophanes. In the topic of ammonium ion recognition, it is difficult to draw a dividing line, as both concepts – clefts and cyclophanes – function similarly or were developed in parallel for similar purposes. We will first discuss clefts, clips and tweezers, then tripods and related systems, and finally cyclophanes with ionic functionalities.

Vitally important biochemical processes involving ammonium ions rest upon the specific interactions supported by negatively charged substituents such as carbonates, sulfates, or phosphates. As demonstrated with several examples before, these charged groups contribute significantly to the substrate binding. For clefts, tweezers and cyclophane structures such substituents are of key importance to complement the ammonium ion binding by ionic and hydrogen bond interactions. In the cavities the guests can be bound utilising non-covalent bonding interactions such as hydrophobic forces, van der Waals or dispersion forces, π-stacking, hydrogen bonding, as well as metal co-ordination and electrostatic effects.

**Figure 109:**
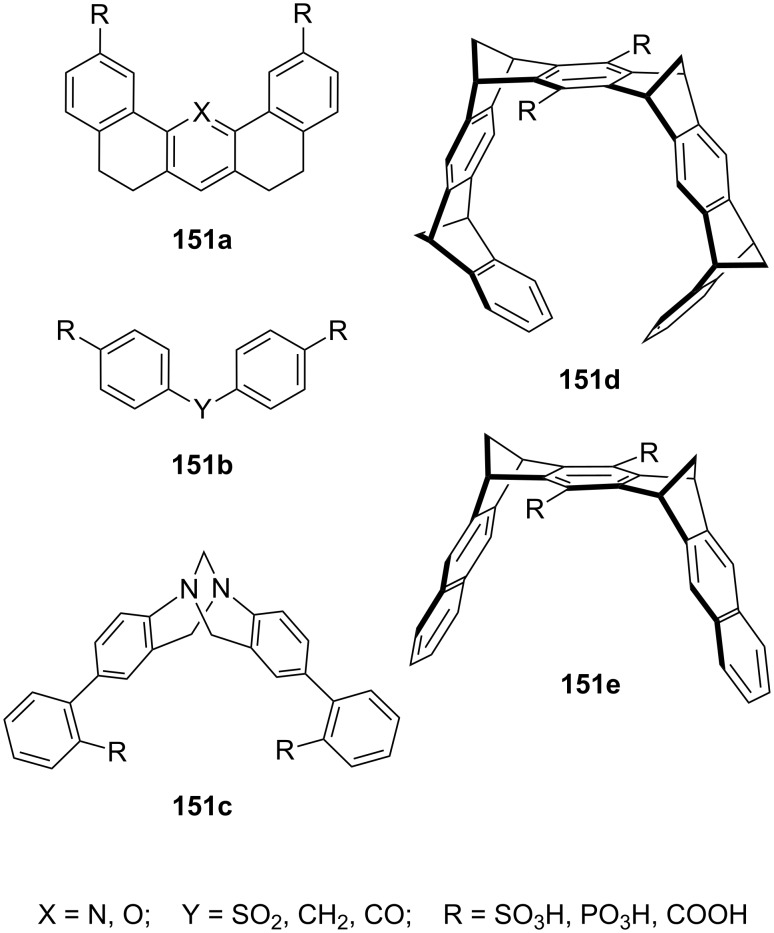
Examples of clefts (**151a**), tweezers (**151b, 151c, 151d**) and clips (**151e**).

Clefts (Figure 109) have a certain degree of flexibility, provided that the open cavity is large enough and the geometry is optimal to accommodate the desired guest molecule. Clefts organize polar functionality with hydrogen bonding or ionic co-ordination capabilities at precise distances and orientations. This conformational fixation is achieved by covalent and non-covalent constraints. Generally, acylic clefts, clips and tweezers must position functional groups on a rigid molecular scaffold, often of concave shape, to focus these inwards, to assure the desired conformation, and to prevent the collapse of the binding pocket. As in macrocycles, proper pre-organization can significantly augment binding strengths.

Molecular tweezers (Figure 109) are different examples of molecular clefts. Molecular tweezers or molecular clips can be understood as non-cyclic macrocyclic molecular complexes with open cavities bearing two “arms” that bind the guest molecule between them [486]. For ammonium ion recognition they divide into two different subtypes: Either they are characterized by convergent functional groups directed towards each other, mounted on a rigid backbone with a certain degree of freedom – the space between the functional groups provides the cleft into which a guest can bind – or the cavity of this kind of receptors is made up of two sidewalls connected to each other by a central spacer unit, which can be either flexible or rigid. The second type contains two aromatic surfaces which “pinch” aryl or more rarely an non-polar guest between them and uses an additional ionic functionality to complement the ammonium part. Molecules like Kagan’s ether or Tröger’s base (see **151c**) are employed in many examples to give the tweezer a bent shape. The synthesis and properties of such often chiral molecular clefts and tweezers have been reviewed [487].

Tweezers and similar molecules “wrapping around” their targets, namely cyclophanes and cavitands, benefit to a large extent from selective co-ordination and inclusion by charged groups. Quaternary ammonium ions can be additionally co-ordinated by the cation–π-interaction to the aromatic surfaces.

Molecular tweezers were originally developed by Whitlock [488–489] and Zimmerman [490–494]. These formed sandwich complexes with aromatic guests by π–π-interactions. Hydrophobic interactions also play a significant role in their tight binding to aromatic (bis-phenol)carboxylates in water. The tweezers constructed by Zimmerman were more rigid and showed high association constants with guests such as polynitroaromatics and 9-alkylated adenines in chloroform.

Further contributions and examples representing the different types of such molecules with open cavities were published by the groups of Vögtle [495], Rebek [496], Nolte [497], Harmata [498–499,518], Chen [500], Klärner [486,501–503] and Schrader (see the discussed example, Figure 112). Cations and some alkyl or a variety of aromatic guests, especially electron deficient aromatic systems [501,504–507] can be co-ordinated by dispersive forces such as π–π, CH–π- and cation–π-interaction. The introduction of polar functionality enables the binding of guests by additional interactions, for example, 1,3-dihydroxybenzene [497] by H-bonding or nucleosides [508–512] by ionic interactions. Similarly, ammonium ions, diamines [513–514] or chiral guests [515] can be recognized by appropriate functional groups arranged on these scaffolds.

Clips, tweezers [486,516], related V-shaped molecules [517] and their chiral analogs (e.g. Figure 109, **151c**) [518–519] have been reviewed. In the following we will discuss recent examples based on these backbones for inclusion of quaternary ammonium compounds, or, when suitably substituted, for ammonium ion recognition.

#### 5.1. Clefts for different ammonium targets

**Figure 110:**
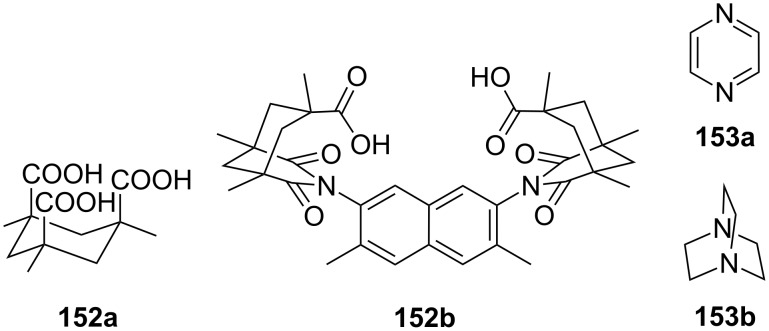
Kemp’s triacid (**152a**), on example of Rebek’s receptors (**152b**) and guests.

The ability to bind the guest by π–π-interactions and thehydrophobic effect is extended by the possibility of hydrogen bonding to the guest molecule with a receptor family developed by Rebek et al. on the basis of Kemp’s triacid (**152a**) (Figure 110). Due to the convergent carboxyl groups on the cyclohexane ring, condensation of the acid with aromatic amines – one to three aromatic rings are arranged in a linear manner – yields receptors such as **152b**, in which two carboxyl groups are pre-orientated in a convergent, optimal arrangement for the substrate binding. Rotation around the C–N bond can be prevented by a methyl group in *ortho*-position of the aromatic amine.

**Figure 111:**
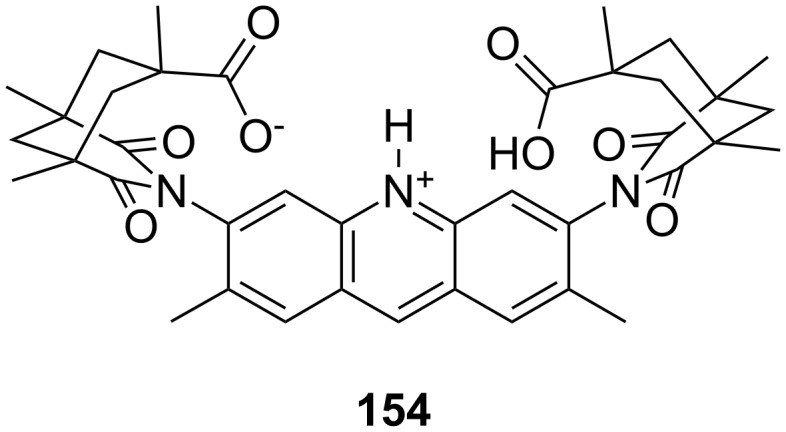
Amino acid receptor (**154**) by Rebek et al.

The largest receptor binds diamines, such as pyrazine (**153a**) or DABCO (**153b**) (Figure 110), in chloroform by salt formation. Dicarboxylic acids are linked by hydrogen bonds, similar to those found in carboxcylic acid dimers. On binding amino acids, a carboxyl group of the receptor co-ordinates to the carboxyl group of the substrate. In addition, salt formation occurs between the other carboxyl group of the receptor and the amino group of the guest [520–521]. Receptor **154** (Figure 111) is able to complex ammonium ions with its carboxylate group; the pyridinium cation binds in addition. The extended π-system allows for π-stacking [522].

The authors identified a binding preference for phenylalanine (**81a**), tyrosine and tryptophan (**81b**) by extraction experiments (water/chloroform) with unprotected amino acids. Leucine, isoleucine and valine were, however, not transported into the organic phase. Thus, the π-stacking interaction seems to result in a decisive contribution to the complex stabilization here. Phenylglycine, due to its geometry, is also not in the position to participate in π-stacking in addition to the molecular bonds of the charged parts. The mode of binding and the interactions were investigated in detail by a theoretical study and verify the results and conclusions [523].

**Figure 112:**
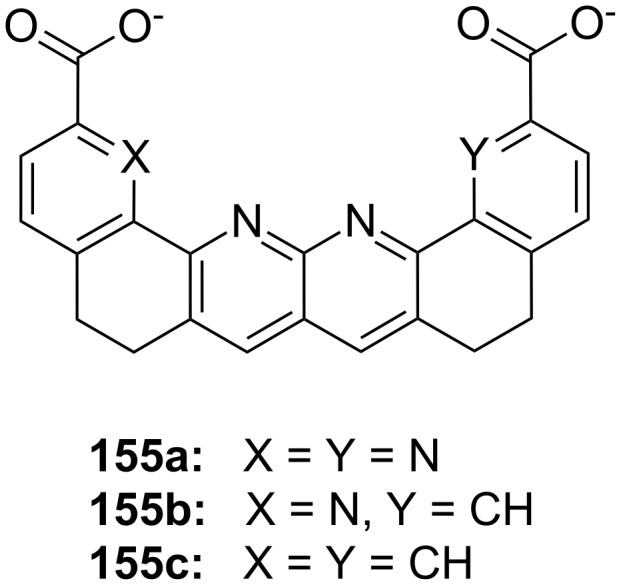
Hexagonal lattice designed hosts by Bell et al.

Because of the frequent occurance of basic amino acids (Lys, Arg, His) in biological processes, the molecular recognition of these amino acids by synthetic receptor molecules is of special interest [524–527]. Bell et al. described three receptors for guanidinium and ammonium guests [528]. These highly pre-organized clefts, bearing two carboxylate groups on a hexagonal lattice design with defined planar arrays of hydrogen-bonding groups, differ in the number of nitrogen atoms contained in their cavity (Figure 112). Complexation studies were conducted in methanol by ^1^H NMR titration for several guanidinium and ammonium ion guests. Compound **155a** bound most guests very strongly (*K*_ass_ > 100 000 M^−1^) and was selective for arginine (**81d**) more than 3-fold versus lysine (**81c**, *K*_ass_ = 29 000 M^−1^). Surprisingly, the affinity for *N*-acetyl-*S*-lysine and propylammonium chloride was also found to be very high (*K*_ass_ = 10^5^ M^−1^). Interesting for ammonium ion recognition is receptor **155b**, which bound lysine (**81c**) better than **155a**. In general, it tends to have higher affinity towards alkylammonium guests than to alkylguanidinium salts. It displayed a preference for binding primary alkylammonium guests, including *S*-lysine (**81c**), *N*-acetyl-*S*-lysine, 6-aminocaproic acid and 1-propylamine (*K*_ass_ = 10^5^ M^−1^). Among guanidinium guests, only arginine (**81d**) bound with very high affinity to **155b**. The complex of **155b** with *N*-methylguanidinium had a significantly lower stability (*K*_ass_ *=* 3900 M^−1^). This selectivity was explained in terms of energies of cavity solvation: The larger cavity of **155a** is more highly solvated prior to binding than the smaller cavity of **155b**. The compact ammonium ion with its higher charge density was expected to form stronger attractive electrostatic interactions. In contrast, the alkylguanidinium ion was able to form more H-bonds with the planar receptor **155a**.

**Figure 113:**
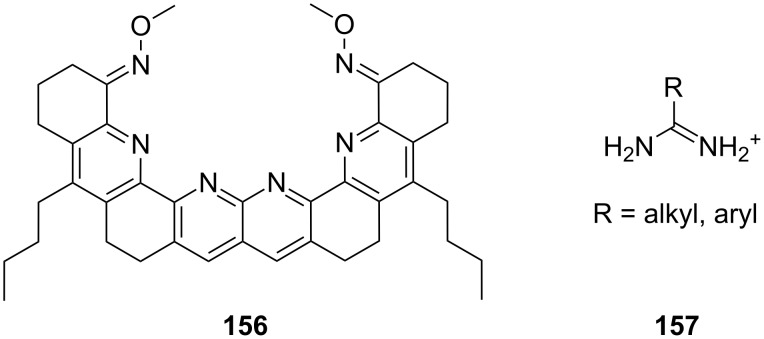
Bell’s amidinium receptor (**156**) and the amidinium ion (**157**).

The amidinium ion is closely related to the ammonium and the guanidinium ion. The amidinium functionality plays an important role in drugs targeting binding pockets for the arginine side chain. In contrast to the spherical ammonium ion, the amidinium group has to be surrounded in a half-moon-like array by at least four hydrogen bond acceptors, which are ideally pre-oriented for maximum electrostatic as well as hydrogen bond interactions for efficient binding. This was demonstrated by Bell et al., who developed a concave, highly pre-organized receptor molecule based on annulated pyridines (**156**) which binds benzamidine (**157**, R = Ph) (Figure 113) very efficiently in 10% methanolic dichloromethane (*K*_ass_ ~ 10^7^ M^−1^) [529].

The efforts of the group concerning the binding of ureas, amines and guanidines by the hexagonal lattice design receptors have been nicely summarized in an overview [530].

#### 5.2. Clips and tweezers

The interaction of carboxylates with a variety of functional groups, receptors for amino acids and nucleotides has been explained in detail in the literature [531–532], and detailed binding data for oxoanions to ammonium and guanidinium groups has been published [533].

Sulfonate groups were widely used with success for the recognition of ammonium ions in calixarenes (see chapter 4), but are of less importance for ammonium recognition with tweezers and clefts. The ammonium – phosphonate binding is by far more widely used as interaction.

The P=O double bond system features strong hydrogen bond acceptor property and weak Brønsted basicity in combination with a high dipole moment. Additional co-operative hydrogen bonds render even simple bisphosphonates highly selective [534].

Many biologically important classes of organic cations like mono- and disaccharides, amino alcohols, arginine derivatives and guanidines are bound in polar media.

**Figure 114:**
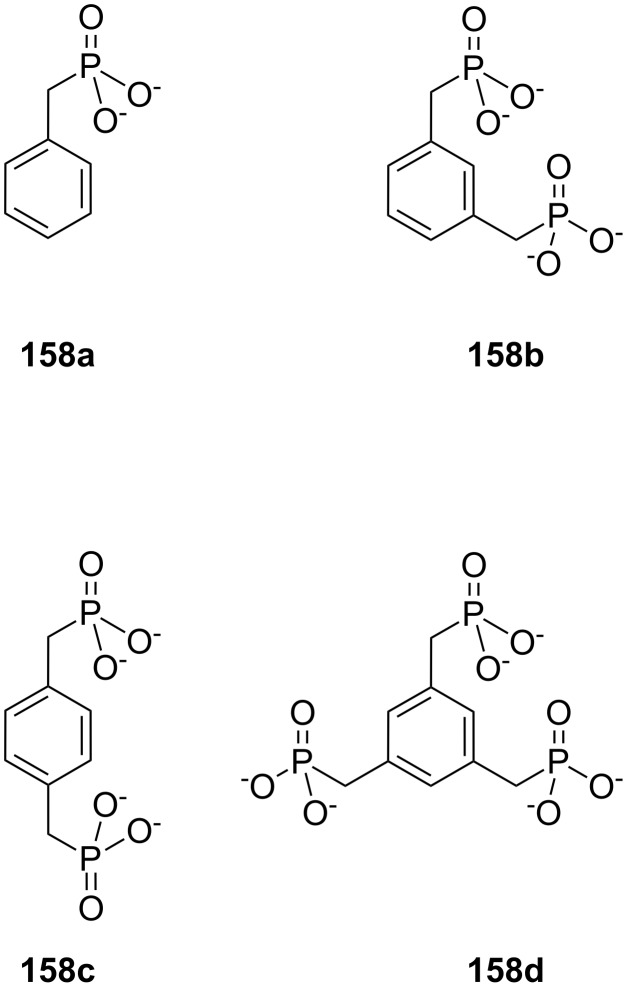
Aromatic phosphonic acids.

Phosphonic acids (Figure 114), phosphonates and their mono esters are especially employed for cation recognition. Simple representatives such as benzyl phosphonic (**158a**), *meta-* and *para-*xylene diphosphonic (**158b/c**) and mesitylene triphosphonic acid (**158d**) have shown their ability to complex selectively potassium and ammonium cations [535]. Ammonium ions were bound two to three times better than potassium in capillary electrophoresis experiments in protic media.

**Figure 115:**
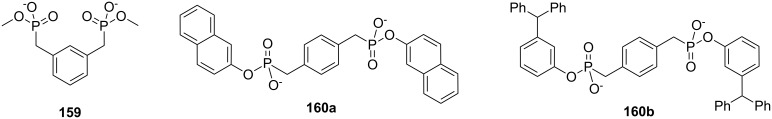
Xylene phosphonates **159** and **160a/b** for recognition of amines and amino alcohols.

In 1996 Schrader introduced a new class of artificial receptor for alkylammonium ions, i.e., xylene bisphosphonates such as **159** [536] (Figure 115). The host molecules, designed to imitate the natural adrenergetic receptor [537–538], are selective for 1,2- and 1,3-amino alcohols. In their 1:1 chelate-binding mode an almost ideal array of short, linear hydrogen bonds with the ammonium ion is created pointing towards one of the phosphonate moieties. Formation of an additional co-operative hydrogen bond between the second phosphonate anion and the hydroxyl groups provides maximum electrostatic and hydrogen-bond interactions. Biologically important amino alcohols such as glucosamine, 1-aminosorbitol, ephedrine, and the β-blocker propranolol were bound in DMSO with *K*_ass_ values of between 60 000 and 130 000 M^−1^. Secondary amines are complexed at least as strongly as primary amines; amino alcohols were bound much stronger than their simple amine counterparts. The association constants for some of the amino alcohols with 60 000 M^−1^ is five times higher than the average estimate for simple amines of 12 000 M^−1^. In addition, adrenaline model compounds were recognized by phosphonates which allow lateral recognition of the substrate by extended aromatic ester groups by π–π-interactions (**160a** and **160b)** [539] (Figure 115). Only a moderate binding of adrenaline to **159** was observed and rationalized by intermolecular competition of the catechol OH groups.

The recognition with *para-*xylene-bisphosphonates was shown with several examples of ammonium [540–541] and guanidinium [528,542] cations by Schrader et al. Similarly, the group demonstrated the recognition of the amidinium ion with the simple *m*-xylene bisphosphonate **159**.

A bifurcated hydrogen bond complex is typical for the classical amidinium binding pattern with carboxylates or phosphonates [543] with values for the association constant usually in the range of *K*_ass_ ~ 10^3^ M^−1^ in solvents such as DMSO [544]. This binding constant could be also observed for the 2:1 complex with **159**. Interestingly, when a 1:1 stoichiometry is ensured by performing dilution experiments with a surplus of **157** with respect to the amidinium ion, a far stronger co-ordination is observed in DMSO: Each amino group is bound by a phosphonate moiety of the tweezer ligand. All association constants lie two orders of magnitude higher than the classical amidinium-phosph(on)ate complexes (10^5^ M^−1^ vs. 10^3^ M^−1^). The association constants for various substituted benzamidines correlate with the electronic character of the substituents. The electron rich *p*-methoxybenzamidine is bound with *K*_ass_ = 7.6 × 10^4^ M^−1^, acetamidine and benzamidine with ~10^5^ M^−1^, and the electron deficient *m*-nitrobenzamidine even with *K*_ass_ = 2.5 × 10^5^ M^−1^.

**Figure 116:**
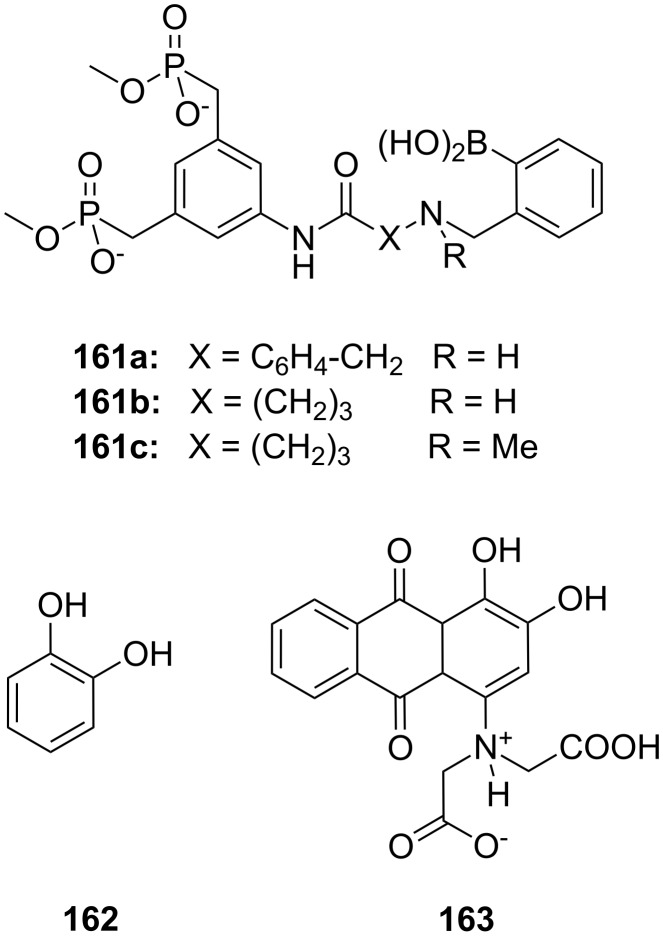
Bisphosphonate recognition motif **161** for a colorimetric assay with alizarin complexone (**163**) for catechols (**162**).

Combination of a boronic ester as recognition motif with the xylene bisphosphonate unit **159** and an appropriate spacer (Figure 116) permitted recognition of neurotransmitters [545]. For noradrenalin (**80b**) in 100 mM phosphate at pH 7.0 a strong association was found (*K*_ass_ = 190, 340 and 690 M^−1^ for **161a**, **161b** and **161c**, respectively). It was possible to evaluate the association constants for a number of catecholamines such as adrenaline (**80a**) and noradrenaline (**80b**) highlighting the importance of both the aminoalcohol and catechol motifs within the guest. Receptor **161c** as the best example bound adrenaline (**80a**, *K*_ass_ = 550 M^−1^), 3,4-dihydroxyphenethylamine (*K*_ass_ = 590 M^−1^), dopamine (**2**, *K*_ass_ = 630 M^−1^) and noradrenaline (**80b**, *K*_ass_ = 690 M^−1^) with about 2-fold selectivity over catechol (**162**, *K*_ass_ = 350 M^−1^). The receptor was then developed into a color sensor by employing the colored dye alizarin complexone in an indicator displacement assay. On binding to the receptors, the color of the dye changed from deep red to orange, permitting an association constant of *K*_ass_ = 1700 M^−1^ to be determined by ^1^H NMR titrations. Upon addition of catecholamines, displacement of the indicator and recovery of the original color were observed. Binding constants similar to those obtained by NMR spectroscopy were obtained by UV spectroscopy in water. Finally, a calibration curve for the receptor-indicator complex in the presence of varying concentrations of noradrenaline was constructed, which allowed an exact quantitative determination of the concentration of catecholamines even in complex mixtures and urine samples. On changing from water to a 3:1 mixture of methanol/water (HEPES buffer, pH 7.0), the *K*_ass_ value for alizarin complexone increased to 7000 M^−1^. A rise in noradrenaline binding could not be confirmed. All catecholamines were bound in the range of 300–400 M^−1^, catechol somewhat less tightly with 200 M^−1^ and simple amines such as phenylethylamine were not bound at all. Adrenaline was bound 2–3 times stronger than catechol.

**Figure 117:**
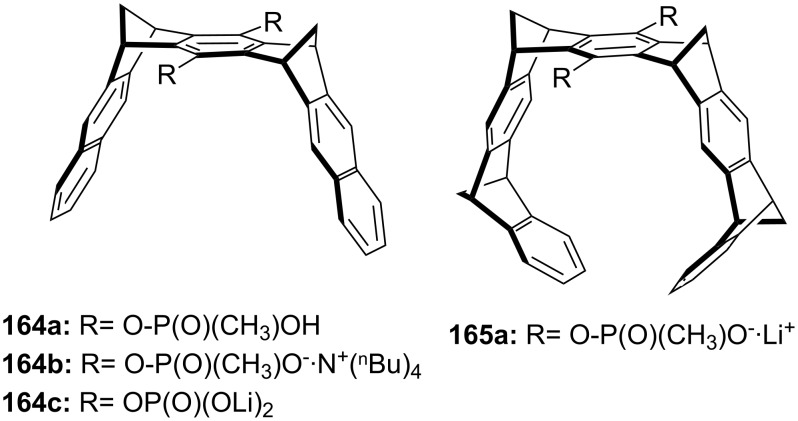
Bisphosphonate/phosphate clip **164** and bisphosphonate cleft **165**.

Klärner and Schrader introduced tweezers and clips based on an electron-rich torus-shaped cavity adorned with two peripheral anionic phosphonate and phosphate groups (Figure 117) capable of ammonium ion and amino acid recognition in water. These molecular tweezers were synthesized via repetitive Diels–Alder reactions and combine the binding properties of a non-polar aromatic cavity with the bisphosphonates. In addition, the bisphosphonate units lead to the desired solubility in polar protic solvents such as methanol and water. In water, the π–π and cation–π-interaction are coupled with the hydrophobic effect, and these are much more pronounced than in aprotic solvents and thus lead to higher binding constants. The phosphonates are fully deprotonated due to their p*K*_a_ value of 1.8 in neutral aqueous solution. Upon inclusion of a guest in the cavity, they can grab it like a pair of pincers and build ionic hydrogen bonds to the ammonium ion to support the binding.

**Figure 118:**
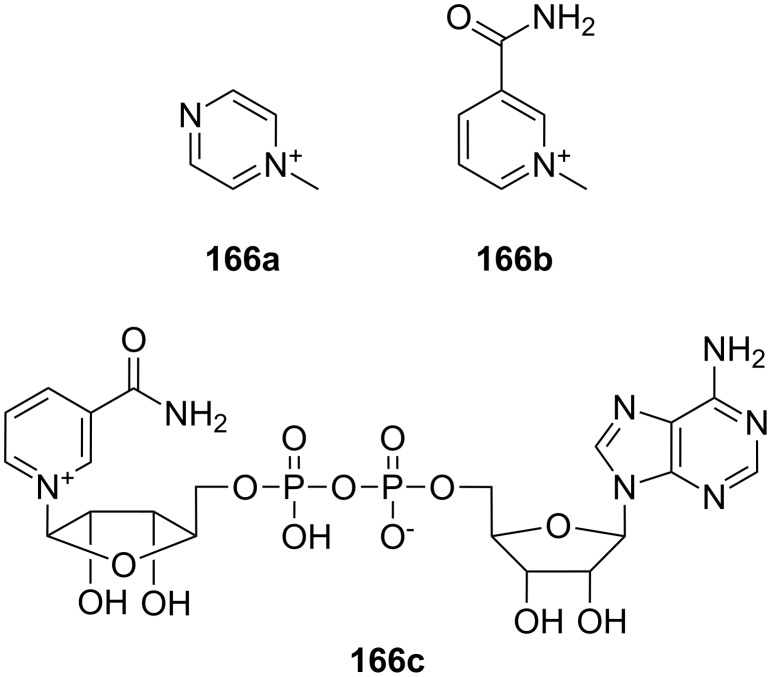
*N*-Methylpyrazine **166a**, *N*-methylnicotinamide iodide (**166b**) and NAD^+^ (**166c**).

The phosphonate substituted clip **164b** [546] selectively binds *N*-alkylpyridinium salts such as *N*-methylnicotinamide iodide (**166b,** NMNA) and NAD^+^ (**166c**) (Figure 118) in methanol and in aqueous solution. Further studies pointed to a significant contribution of the hydrophobic effect to the host–guest interaction in aqueous solution [547]. The binding constants in water are significantly higher, than those observed in methanol: for example, **166a** bound with *K*_ass_ = 9400 or 600 M^−1^ and **166b** with *K*_ass_ = 68000 or 16700 M^−1^ in water and methanol, respectively.

In the complex with NAD^+^ (**166c**, *K*_ass_ = 6500 M^−1^), one of the most important redox coenzymes in nature, a dynamic equilibrium is observed in aqueous solution. The protons of the subunits, the nicotinamide as well as the adenine moiety, are shifted upfield in the ^1^H NMR spectrum indicating that either the nicotinamide or the adenine subunit are included inside the cavit. Equilibration is rapid on the NMR time scale. A Monte Carlo conformer search, leading to the energy-minimized double-sandwich structures supported the experimental result.

Water-soluble molecular clips substituted with phosphate groups (**164c**) (Figure 117) were also investigated regarding their binding properties. Despite the similarity between the phosphonate and phosphate functional groups, the supramolecular properties of both clips are different from each other. The phosphate clip lithium salt **164c** shows self-aggregation in aqueous solution while there is no evidence of this phenomenon for the phosphonate clip dilithium salt **164a** [548]. Additionally, the binding properties of these clips in phosphate buffer solution (pH = 7.2) change dramatically from one clip to another (Table 9) as well as with the pH values of the solution. For the most guest molecules, the phosphate clip **164c** shows association constants between two and ten times larger than those of the phosphonate clip **164a**.

**Table 9:** Comparison of association constants (M^−1^) of biological relevant molecules with the phosphonate and phosphate clips in phosphate buffered aqueous solution (pH = 7.2).

Guest	Phosphonate clip **164b** *K*_ass_ [M^−1^]	Phosphate clip **164c** *K*_ass_ [M^−^^1^]

nicotinamide mononucleotide	550	1120
adenosine	1115	1400
cytidine	1070	9685
*N*-methylnicotinamide iodide (**166b**)	11270	35000
caffeine	9550	42700
NAD^+^ (**166c**)	4200	5630

*N*-Alkylated pyridinium salts are also strongly bound in the tweezer **165a**. Only *para*-substituted compounds are strongly bound, other substitution patterns do not lead to an effective inclusion in the downward shielded cavity.

In contrast to the bisphosphonate clip, the bisphosphonate tweezer also binds primary and secondary ammonium cations. The binding correlated with the steric requirements of substituents. The bulkier the substituent, the lower is the binding constant. Primary ammonium cations (*K*_ass_ up to 800–900 M^−1^ in aqueous solution) are bound more strongly than their secondary analoges. Dopamine (**2**) is bound with millimolar strength in water. Interestingly, the basic amino acids arginine (**81d**) and lysine (**81c**) are significantly better bound (up to 23000 M^−1^ for Ts-Lys-OMe in aqueous phosphate buffer) compared to 900 M^−1^ for simple amines.

The molecular cleft (**165a**) displayed comparable and also exceptionally high affinity for lysine (**81c**, *K*_ass_ = 5000 M^−1^ in neutral phosphate buffer) [549]. Selectivity for arginine (**81d**) and lysine (**81c**) is achieved by threading the whole amino acid side chain through the cavity and subsequent locking by formation of a phosphonate-ammonium/guanidinium salt bridge, reflecting a pseudorotaxane-like geometry. Thus the aggregate can be stabilized by strong electrostatic and dispersive interactions, supported by the hydrophobic effect.

The basic amino acids were effectively bound in small signaling peptides (Lys or Arg rich). These experiments confirmed the selectivity. When two lysine residues separated by other amino acids are present in the peptide, both can be individually bound by one bisphosphonate tweezer in a 2:1-complex. With two lysine residues close together, the formation of a cluster with the bisphosphonates was preferred in a water/methanol mixture. In this case it is apparently more favorable to build hydrogen bonds from the ammonium cations to the bisphosphonates, rather than trapping the lysine side chains in the cavity. This artificial lysine binder shows a one order of magnitude increased affinity compared to all other receptor molecules that have been designed for this purpose. Only Bell’s molecule (**155c**) was later identified as a selective lysine binder (*K*_ass_ > 10^5^ M^−1^ in methanol). The binding mode and strength seem to be largely governed by steric effects: bulky substituents close to the ammonium functionality prevent an effective inclusion, while a slim ethylammonium environment allows complete insertion into the host interior.

**Figure 119:**
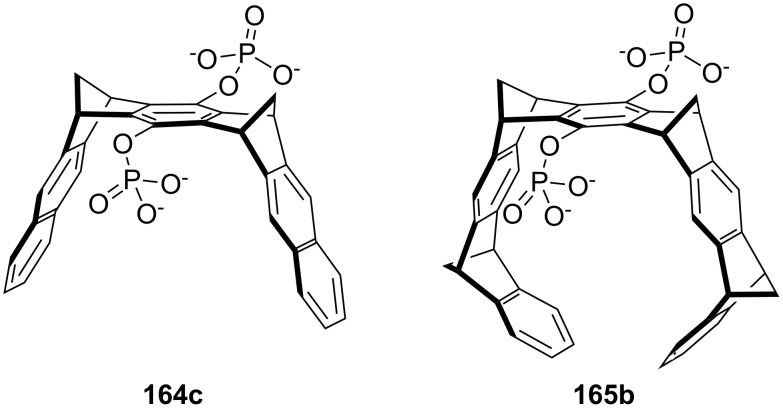
Bisphosphate cavitands.

The two corresponding water-soluble host molecules with phosphate substituents (Figure 119) designed for cofactor and amino acid recognition are able to inhibit the enzymatic activity of alcohol dehydrogenase (ADH) in vitro [550]. As previously noted, clip **164c** binds strongly to NAD^+^ (**166c**) and tweezer **165a** shows high affinity to lysine (Ac-Lys-OMe, *K*_ass_ = 5000 M^−1^) in aqueous buffer. Clip **164c** pulls out NAD^+^ (**166c**) from the Rossman fold and thereby depletes the cofactor level below a critical threshold. An excess of this molecule led to irreversible denaturation. Tweezer **165b** with its high lysine preference decorates the whole enzyme surface, especially the cofactor entrance site. While the absolute enzymatic activity was not influenced at all, 0.6 equiv of tweezer was sufficient for total enzyme shut down. Addition of lysine (**81c**) could switch on the enzyme function again in a totally reversible manner. Lineweaver-Burk plots indicated a competitive mechanism for the clip, with respect to both substrate and cofactor, while the tweezer clearly follows a non-competitive mechanism.

**Figure 120:**
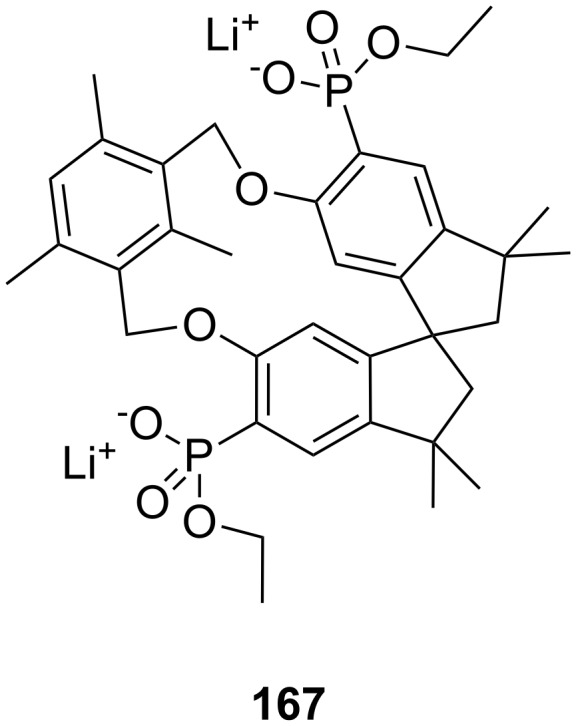
Bisphosphonate **167** of Schrader and Finocchiaro.

In 2000 a macrocyclic receptor molecule, which binds arginine (**81d**) and lysine (**81c**) in a stereoselective fashion was reported [551]. The chiral bisphosphonate **167** (Figure 120) binds ammonium and guanidinium ions by hydrogen and salt bridges. The mechanism of enantioselective recognition relies on two simultaneous cation–phosphonate interactions. The amino acid is in close contact to the surface of the chiral tether in **167** and one enantiomer is bound preferentially. The overall binding constants were only in the range of 10^4^ M^−1^ in DMSO.

In examining the binding properties by NMR titration in DMSO, the authors found out that for short diammonium guests such as *S*-histidine (**81e**), and *S*-ornithine (both as dihydrochlorides) a 1:2 (receptor: guest) stoichiometry is present, but there is no chiral discrimination. However, the complexes for lysine (**81c**, *K*_ass_ = 2.1 × 10^4^ M^−1^) and arginine (**81d**, *K*_ass_ = 9.4 × 10^3^ M^−1^) have a 1:1 molar ratio and a distinction between the enantiomers is possible. The distance between the two ammonium groups in a guest molecule must obviously be large enough to bind to both phosphonates of the receptor. The enantiomeric excess was determined to be 17% for arginine (**81d**) and 33% for lysine (**81c**).

**Figure 121:**
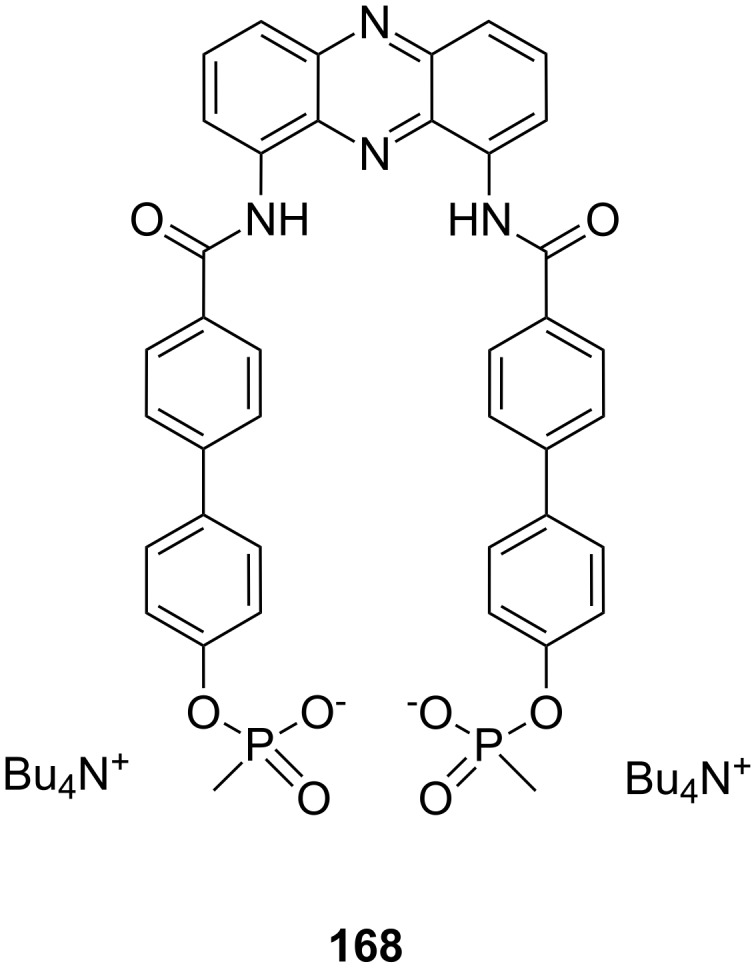
Tweezer 168 for noradrenaline (**80b**).

An artificial receptor molecule **168** with high noradrenalin specificity uses highly pre-organized stiff elements and connections (Figure 121) for a more favorable complexation entropy and improved desolvation of the included guest [552]. NMR titrations with neurotransmitters and related guests in *d*_4_-methanol revealed low micromolar affinity to *rac*-adrenaline (**80a**, 260 M^−1^), dopamine (**2**, 340 M^−1^) and aromatic amino acid esters (~200 M^−1^). Other amino acids, catechol (**162**) and phenylethylamine (**78a**) gave no response. Job’s plot analysis confirmed a 1:1 complex stoichiometry. The rigid phenazine moiety in receptor **168** strongly improves the affinity for the desired guest (*K*_ass_ = 1800 M^−1^). The effective 1:1 complex formation between (**168**) and noradrenaline (**80b**) could also be monitored by ESI-MS, producing clean mass spectra with host and aggregate ion peaks, exclusively.

Due to the highly amphiphilic structure of **168**, the receptor molecule was incorporated in a stearic acid monolayer at the air/water interface. In the Langmuir film balance, substantial shifts were produced upon subinjection of the various analytes into the aqueous sub-phase (10^−4^ M) reflecting the interaction with the embedded receptor molecule (no effects were produced with stearic acid alone). By far the largest shift is obtained from noradrenaline (**80b**), followed by much smaller shifts from adrenaline (**80a**) and dopamine (**2**).

#### 5.3. Tripodal receptors

**Figure 122:**
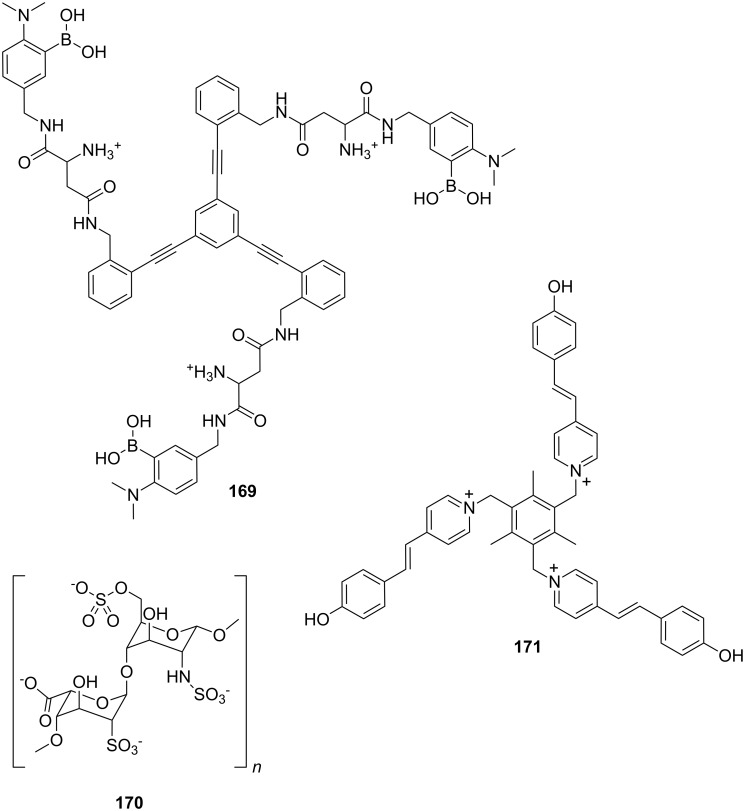
Different tripods and heparin (**170**).

Tripodal ligands are *C*_3_ symmetrical molecules related to tweezers, with three side chains on a rigid platform (Figure 122). Several of these artificial receptors have *C*_3_*_v_* symmetry [553–557]. In ammonium ion recognition with tripods, the flexible arms form three hydrogen bonds to acidic protons of the guest amine RNH_3_^+^.

The binding can benefit from this additional co-ordination site. Even more, recognition of biologically important guests often necessitates a receptor that can make multiple non-covalent contacts. This concept was nicely demonstrated with receptor **169** utilizing the threefold ammonium sulfonate/sulfate contact to recognize heparin (**170**) [558] and bind it strongly with *K*_ass_ = 1.4 × 10^8^ M^−1^ in 10 mM HEPES buffer [559].

Such a three-point co-ordinating cavity can better exclude solvent influences and enables recognition in strongly competitive solvent mixtures. For example, colorimetric discrimination between certain ω-aminoacids (H_3_N^+^–(CH_2_)*_n_*_−1_COOH) was achieved by the use of a chromogenic tripodal receptor functionalized with stilbazolium dyes (**171**) in mixed DMSO–water 90:10 v/v solutions [560]. UV-experiments revealed a preference for *n* = 4–6 (λ = 560 nm).

**Figure 123:**
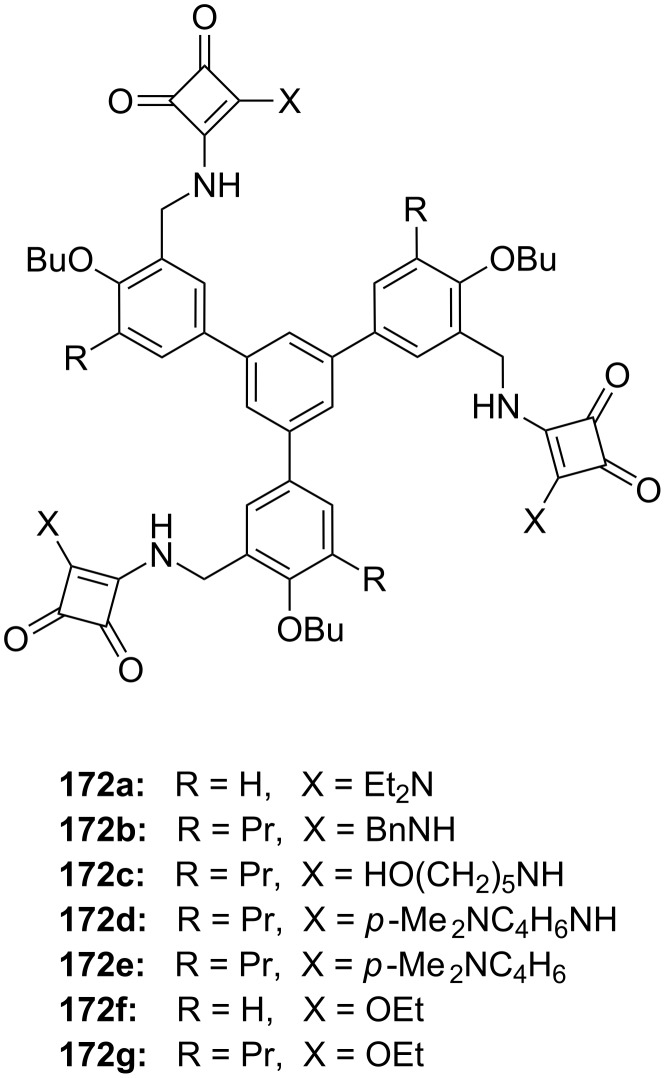
Squaramide based receptors **172**.

Quaternary ammonium ions can be co-ordinated entirely utilizing, for example, an additional cation–π-interaction with the third arm. The group of Ballester introduced squaramido rings as binding units in abiotic tripodal receptors (Figure 123), thus utilizing multiple O to C–H interactions [561]. This led to efficient receptors for tetraalkylammonium compounds such as choline (**76**), acetylcholine (**3**) and related ammonium salts. Association constants in the range 10^3^ to 10^4^ M^−1^ were determined by a ^1^H NMR titration using a 1:1 model (**172e** vs. choline (**76**) hydroiodide in CDCl_3_: *K*_ass_ = 14509 ± 1403 M^−1^). The formation of intracavity complexes was supported by intermolecular cross peaks in 2D ROESY experiments. Complexation studies carried out in 10% MeOD-*d*_4_/CDCl_3_ mixtures gave association constants that were roughly 20–25 times weaker than in CDCl_3_ alone, but the formation of the corresponding complexes was still evident.

**Figure 124:**
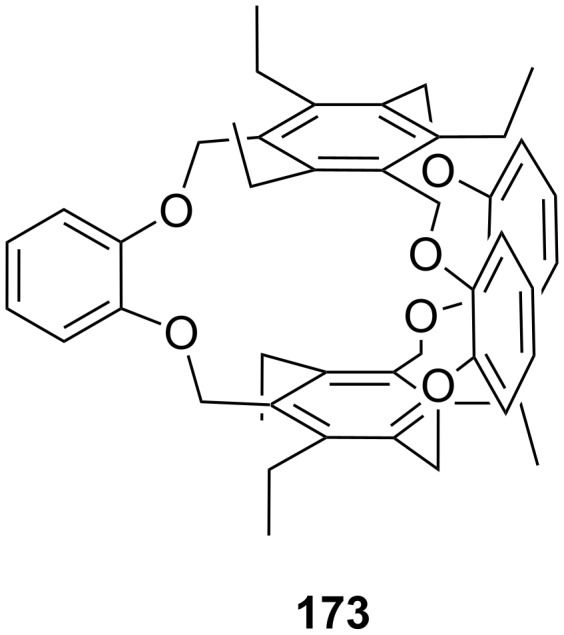
Cage like NH_4_^+^ receptor **173** of Kim et al.

The interaction with aromatic π-electron clouds plays an important role in the interaction of the synthetic NH_4_^+^ receptor (**173**) by Kim [128]. The cage like molecule (Figure 124) binds ammonium ions in addition by multiple hydrogen bonds and by cation–π-interactions.

The cavity has been calculated to be optimal for ammonium ions, but too large for lithium- and sodium ions. When used in ion selective electrodes, **173** showed a slightly higher detection limit (3.2 × 10^−6^ M) as the natural ammonium sensor nonactin (1.5 × 10^−6^ M) and an increased ammonium/potassium selectivity coefficient of log *K* (NH_4_^+^)/(K^+^) = −0.97 (Nonactin: log *K* (NH_4_^+^)/(K^+^) = −0.88). The binding constant of the ammonium ion determined by extraction experiments [188] was 3.3×10^7^ M^−1^.

**Figure 125:**
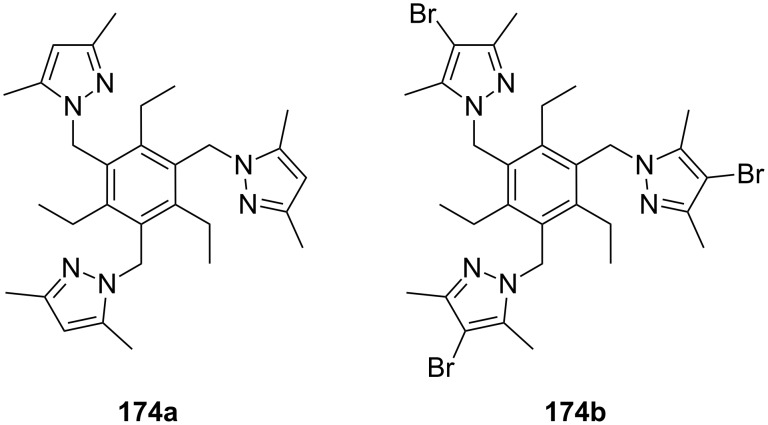
Ammonium receptors **174** of Chin et al.

Chin and co-workers synthesized 1,3,5-tri(3,5-dimethylpyrazol-1-ylmethyl)-2,4,6-triethylbenzene (Figure 125) in which the three pyrazole groups provide hydrogen-bonding sites [562]. In comparison to **173**, receptor **174a** shows an increased ammonium selectivity (log *K* (NH_4_^+^)/(K^+^) = −2.6), but the binding constant, determined by extraction experiments [189], was lower (*K*_ass_ = 1.4 × 10^6^ M^−1^). An ion selective electrode (ISE) incorporating this molecule showed improved ammonium ion over potassium ion selectivity as compared to nonactin (log *K* (NH_4_^+^)/(K^+^) = −2.6), again illustrating the importance of hydrogen bonding and symmetry. This ionophore is pre-organized into the required tetrahedral geometry for complexing ammonium ions through hydrogen bonding involving the imine nitrogen atoms. The ethyl and methyl groups provide steric interactions to force the receptor into the desired geometry and to block the ligands from binding potassium ions. Despite its high selectivity for ammonium, the limit of detection for this ionophore is two orders of magnitude higher than for nonactin, and therefore, it is not sufficiently sensitive for some applications.

To lower the binding of water and thus increase the sensitivity of the receptor, electron withdrawing groups – bromine atoms – were introduced in the pyrazole rings of the receptor (**174b**) [563] (Figure 125). This modification did indeed lead to a far lower detection limit (2.5 × 10^−5^ M) for ammonium ions in an ISE, comparable to nonactin (2.2 × 10^−5^ M). The ammonium versus potassium selectivity of this receptor was strongly enhanced compared to the unbrominated heterocycle (log *K*_NH4+/K+_ = −2.3, nonactin log K_NH4+/K+_ = −1.3).

**Figure 126:**
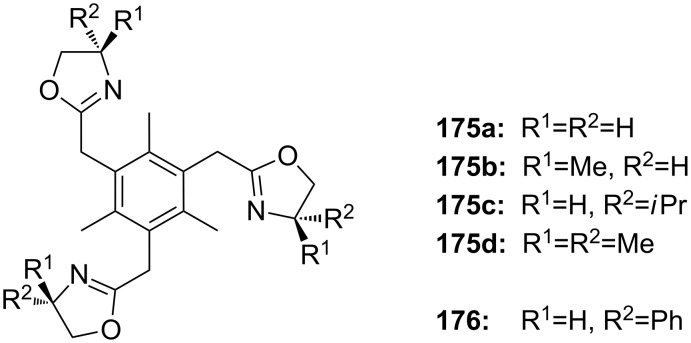
2-Oxazolin-based ammonium receptors **175a**–**d** and **176** by Ahn et al.

The further development of this structural motif, carried out by Ahn et al., led to an exchange of the weakly basic pyrazole (p*K*_a_ ≈ 2.5) with the 2-oxazoline (Figure 126) of slightly higher basicity (p*K*_a_ ≈ 5) [564].

The binding constants of the molecules **175a** – **d** towards ammonium and potassium ions were investigated by picrate extraction experiments [189] and were compared to the natural ammonium binder nonactin (Table 10).

**Table 10:** Binding constants and selectivity constants of the receptors **175a**–**d**.

	**175a**	**175b**	**175c**	**175d**	*Nonactin*

*K*_ass_ (NH_4_^+^) [M^−1^]	5.1 × 10^6^	2.5 × 10^7^	9.4 × 10^6^	3.9 × 10^6^	2.0 × 10^8^
*K*_ass_ (K^+^) [M^−1^]	3.0 × 10^4^	5.7 × 10^4^	2.4 × 10^4^	5.7 × 10^4^	6.7 × 10^7^
*K*_ass_ (NH_4_^+^)/*K*_ass_ (K^+^)	173	437	393	68	3

**Figure 127:**
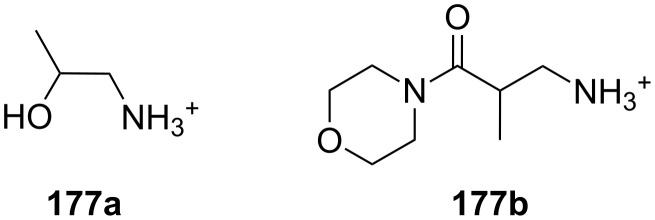
Racemic guest molecules **177**.

Due to these structural changes, the authors succeeded in further improving the binding constants (*K*_ass_ (**174**, NH_4_^+^) = 1.4 × 10^6^, *K*_ass_ (**175b**, NH_4_^+^) = 2.5 × 10^7^ M^−1^) and enhancing the NH_4_^+^/K^+^ selectivity from 398 to 437. Another advantage of oxazoline compared to the pyrazole substituents is the possibility of introducing chirality into the receptor. Ahn et al. have studied the binding of enantiomerically pure **176** towards a variety of guest molecules [565]. An increase in discrimination of the enantiomers of racemic molecules is represented by the presence of a hydrogen bridge acceptor in γ- or β-position to the ammonium ion. The authors rationalized this to the existence of a “bifurcated” H-bridge, which restricts the free rotation of the β-substituent. From ITC titration experiments in acetonitrile, the binding constants for the *R*- and *S*-enantiomers of **177a** were found to be 3.0 × 10^4^ M^−1^ or 9.2 × 10^3^ M^−1^, respectively. The enantioselectivity of the extraction is 63:37 in favor of the *R*-enantiomer. The best selectivity found for **177b** (Figure 127) was 83:17, but only an extraction of <5% was possible due to the increased water solubility of **177b**.

Theoretical studies indicated such trisoxazolines are alternatives to azacrowns for binding and sensing of ammonium and alkylammonium ions [565–567]. The importance of the *C*_3_ symmetry in chiral recognition has been pointed out [555]. Apart from Kubiks cyclo-hexapeptide (**233**) and the example **176** from Ahn et al. previously noted, there are only a few examples of enantioselective receptors for chiral ammonium ions with *C*_3_ symmetry [554,568–571].

This receptor type is built by coupling the chiral binding arms to the achiral backbone in such a way that they can organize themselves around a potential guest in a predetermined arrangement. To obtain sufficient stereoinduction, the chiral elements and the donor groups have to be arranged close to each other. An alternative design of three-armed, *C*_3_ symmetric receptors for enantiomeric discrimination is the use of chiral scaffolds to which achiral binding arms can be coupled. Here, the scaffold not only serves as a spacer but also pre-organizes the conformation of the binding arms, thus leading to an enantioselective discrimination of chiral guests.

**Figure 128:**
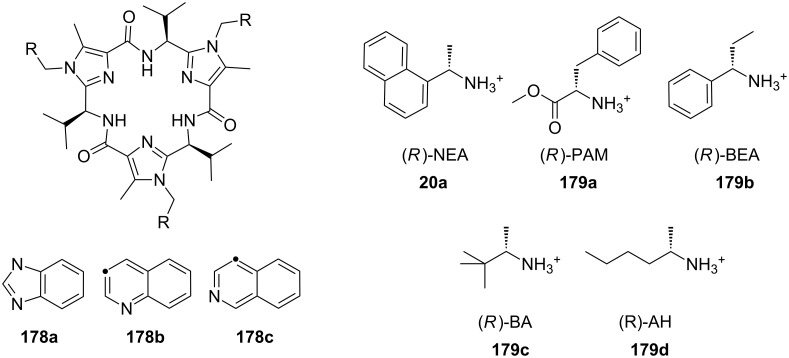
Tripods based on a imidazole containing macrocycle (**178**) and the guest molecules employed in the study (**20a**, **179a–d)**.

Only recently Schnopp and Haberhauer described *C*_3_ symmetric, imidazole-containing, macrocyclic peptides with different binding arms (Figure 128) that bind α-chiral primary organoammonium ions with up to 30,000 M^–1^ [572]. The binding constants and the selectivity ratios were estimated by standard ^1^H NMR titration techniques in CDCl_3_. The chirality of the backbone [573] and the selection of adequate receptor arms make these systems highly selective enantiodiscriminators. The receptors **178b** and **178c** showed opposite selectivities toward those organoammonium ions bound most strongly. With the isoquinoline receptor **178c**, it was possible to generate a *C*_3_ symmetric receptor with a good selectivity ratio of 87:13 for (*R*)-PEA (**20b**). The obtained binding constants were 4500 M^–1^ for (*S*)-PEA and 30,000 M^–1^ for (*R*)-PEA (**20b**).

Titrations of (*R*)-PAM (**179a**) and (*S*)-PAM with **178b** resulted in values for *K*_ass_ of 16,000 M^–1^ and 1900 M^–1^, respectively, thus reaching the high selectivity ratio of 90:10 [574]. A possible explanation for the enantioselectivity was deduced from the conformation of the complexes: They calculated the molecular structures of the energetically preferred conformers of **178c** × (*R*)-PEA and **178c** × (*S*)-PEA by density functional theory (DFT) reproducing their observations in the theoretical model by finding a less favored conformation and higher steric repulsion for the complex with (*S*)-PEA.

**Figure 129:**
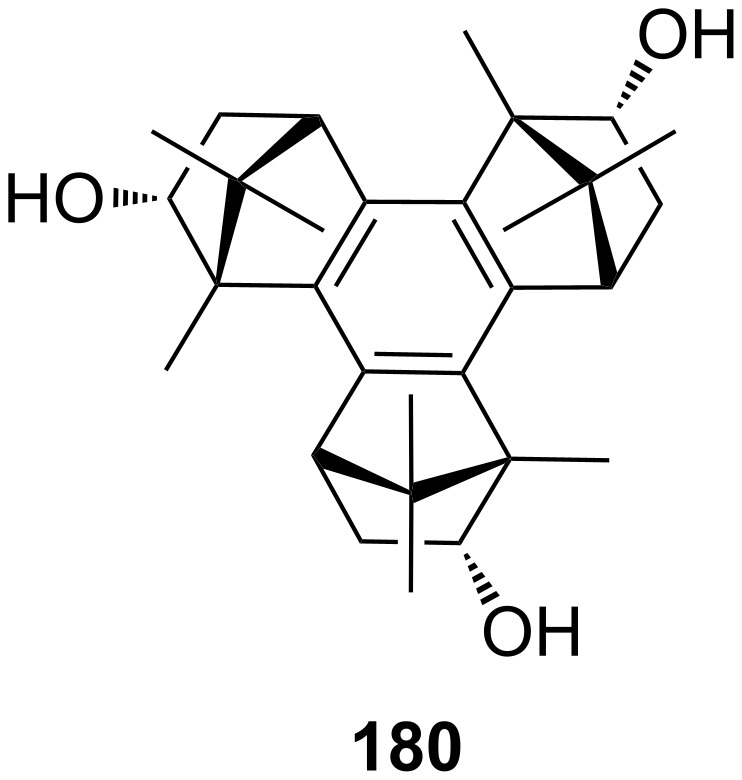
Ammonium ion receptor **180**.

The enantiopure *C*_3_ symmetric *syn*-benzotriborneol **180** (Figure 129) revealed the capability to act as host for ammonium ions, and in particular, the efficient chiral recognition of the two enantiomers of (1-phenylethyl)ammonium chloride [575]. The rigid *C*_3_ symmetric structure of triol **180** bearing three hydroxy groups on the concave side of the molecule, led to two fold better complexation capabilities of the triol *syn*-**180** with (–)-(1-phenylethyl)ammonium chloride (*K*_ass 1:1_ = 230 M^–1^, *K*_ass 1:2_ = 2380 M^–1^) with respect to the (+)-enantiomer (*K*_ass 1:1_ = 120 M^–1^, *K*_ass 1:2_ = 1220 M^–1^). The complexes were characterized in deuteriochloroform by means of ^1^H NMR titrations. The Job’s plots showed the clear formation of the 1:2 complex between the triol and the ammonium salt. The NMR titration experiments clearly showed that two different processes take place. The process that takes place at low concentrations is the complexation of the first ion pair whilst at high concentrations binding of a second ion pair for the reformation of the dimer present in solution occurs.

#### 5.4. Cyclophane structures for binding ammonium ions

Cyclophanes are well pre-organized macrocycles with several aromatic subunits [258], which usually have a large hydrophobic cavity capable of inclusion of neutral or positively charged guest molecules. Their binding properties and their solubility can be varied within a wide scope by introducing appropriate substituents.

Neutral aromatic guest molecules bind to cyclophanes over dispersive and π–π-interactions. In the complexation of organic cations the cation–π-interaction gives crucial contributions. Dougherty and co-workers [576–579] and the group of H. J. Schneider [580–581] proved cyclophane hosts to be suitable for recognition of quaternary ammonium salts: the positive charge of the guest interacts with attractive cation–π-interactions provided by the electron-rich surfaces of their aromatic rings. This fact was also verified by a theoretical study [582]. Such a charge-assisted NH–π-interaction was confirmed only recently [583].

Quaternary ammonium guests such as acetylcholine (**3**) and tetramethylammonium salts (TMA) are strongly bound mainly by cation–π-interaction [261,584–591]. Paraquat and its derivatives are also strongly included, also assisted by π–π-interaction [205,592].

Of equal importance to the properties of these cavities are their peripheral solubilising groups. Water-soluble derivatives especially have a great importance in the host–guest chemistry of cyclophanes. Water soluble cyclophanes are a well known class of receptors providing hydrophobic cavities of definite shape and size for inclusion complexes with various organic compounds in aqueous solution [593–595]. The hydrophobic effect critically assists the co-ordination to ammonium compounds by strong inclusion of the non-polar part of the guest in the cavity [580,596] and plays an important role in the complex formation in general, i.e. the release of guest molecules from the solvation shell around host and guest [597]. In addition, competitive interactions of the H-bond donor water are reduced by the apolar shielding. The synthesis [598–601] and interactions [602] of cyclophanes with typical guest molecules have been described in numerous publications.

**Figure 130:**
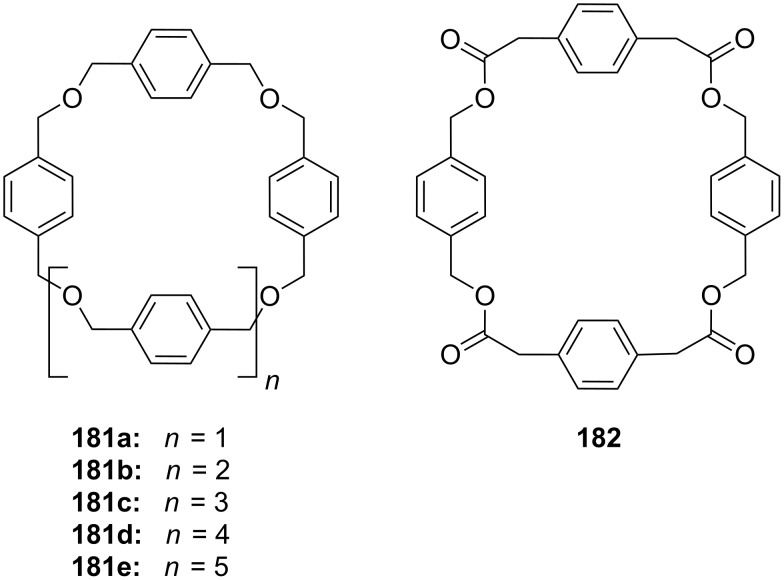
Tetraoxa[3.3.3.3]paracyclophanes **181** and a cyclophanic tetraester (**182**).

A series of oxa[3.*n*]paracyclophanes (Figure 130) was investigated with respect to their binding properties towards quaternary ammonium ions, namely tetramethylammonium and acetylcholine (**3**) with different counter ions in CDCl_3_ by ^1^H NMR titrations [603].

Association of **181a** with tetramethylammonium picrate (*K*_ass_ = 460 M^−1^) was compared to the parent tetraester **182**, the corresponding cyclophanic tetraamine, the open-chain counterpart of **181a**, and its cyclo-oligomers from the pentamer (**181b**) to the octamer (**181e**). Binding enhancements ranging from 15-fold (with respect to the tetraester and the tetraamine) to over 80-fold (with respect to the open-chain tetraether) were observed. With the appropriate choice of the anion, i.e., with a poorly inhibiting counterion (Me_2_SnCl_3_^−^), the association constant for tetramethylammonium is raised to the order of 10^3^ M^−1^, with a binding increase of over 400-fold with respect to the tetraester. Acetylcholine (**3**) was bound by **181a** with 440 M^−1^ (counterion Me_2_SnCl_3_^−^) or 360 M^−1^ (picrate salt).

Many attempts have been made to create synthetic receptor molecules for catecholamines. Most of these are monotopic: for example, dopamine selectivity has been achieved with a pyrazole containing podand [604], a homocalix[3]arene triether [605], or with a sol–gel process [606].

Boronic acids have been used in ditopic receptors for molecular recognition of the catechol ring, as shown in the example above (**161**), by the systems of Glass et al. (**247**) and with related systems in literature [607–608]. In an alternative design, the catechol has been bound by a symmetric hydrophobic cavity with peripheral carboxylate groups for dopamine (**2**) recognition [609].

**Figure 131:**
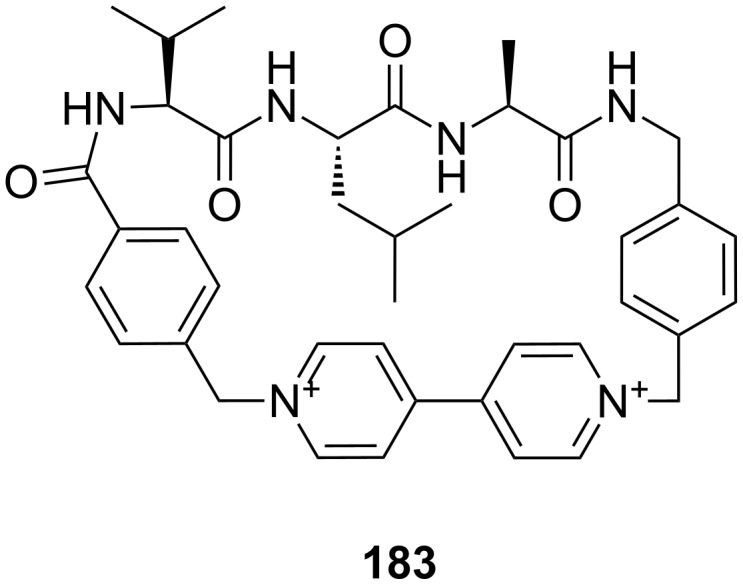
Peptidic bridged paraquat-cyclophane.

A cationic chiral cyclophane (Figure 131) was synthesized and studied as a host for chiral and racemic π-donor molecules. The cyclophane host **183** has a rigid binding cavity flanked by (*S*)-(valine-leucine-alanine) and *N,N*′-dibenzyl-4,4′-bipyridinium subunits, which allow for hydrogen-bonding and π-stacking interactions with included aromatic guest molecules [610].

^1^H NMR binding titrations were performed with several different pharmaceutically interesting guest molecules including β-blockers, NSAIDs, and amino acids and amino acid derivatives. The host–guest complexation constants were generally small for neutral and cationic guests (0–39 M^−1^ at 20 °C in water/acetone mixtures). However, an enantioselectivity ratio of 13 was found for dopamine (**2**), a strongly π-donating cationic guest. (*R*)-Dopamine showed the strongest association in 1:1 water/acetone (39 M^−1^).

Two-dimensional NOESY ^1^H NMR spectra confirm that (*R*)-dopamine binds inside the cavity of the host and that there is no measurable interaction of the cavity with (*S*)-dopamine under the same conditions.

All of these artificial host molecules are not biomimetic and not selective for catechol-amino alcohols. Schrader et al. studied the natural surroundings of such guest and published several approaches based on the imitation of the natural receptors.

In order to imitate the natural binding site, an artificial biomimetic adrenaline host should be able to provide – at least after an induced-fit process – a microenvironment with a shape complementary to the geometrical form of its guest. A high number of van der Waals contacts would help desolvation in water and lead to a strong hydrophobic attraction.

**Figure 132:**
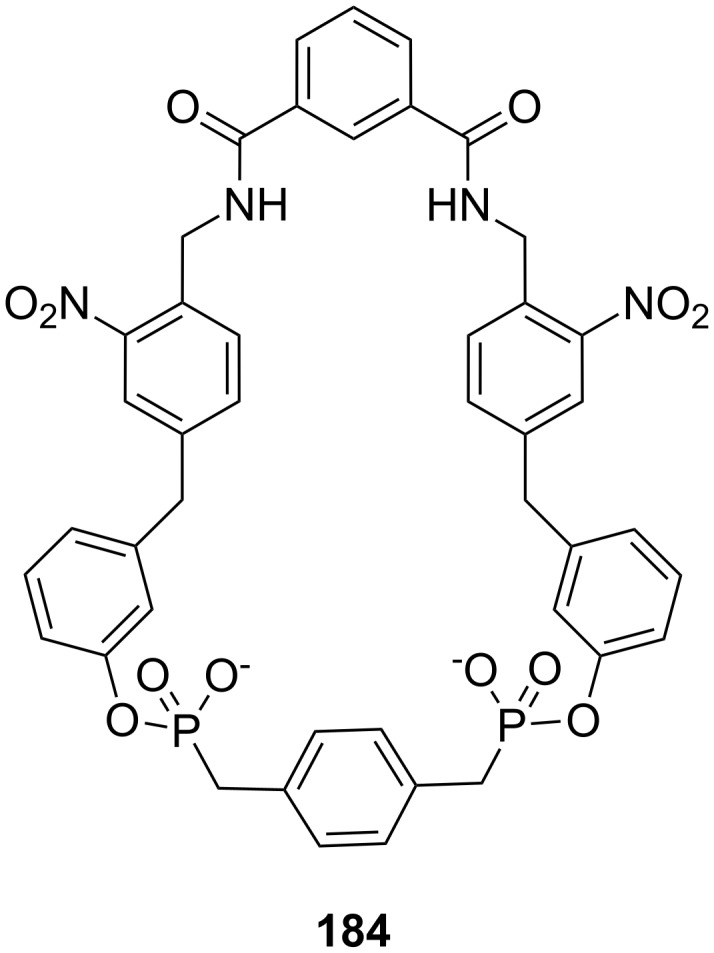
Shape-selective noradrenaline host.

A shape-selective adrenaline-inspired host was investigated [611] (Figure 132). A number of closely related biogenic amines and amino alcohols were examined in a 1:1 mixture of water and methanol by NMR to check the selectivity of the new host molecule.

Adrenaline (**80a**, *K*_ass_ = 153 M^−1^), noradrenaline (**80b**, *K*_ass_ = 215 M^−1^) and dopamine (**2**, *K*_ass_ = 246 M^−1^) were stronger bound than 2-phenylethylamine (**78a**, 102 M^−1^) and ethanolamine (54 M^−1^). The binding constant for dopamine (**2**) in water is three orders of magnitude lower than that of the natural example (10^5^ M^−1^).

The small *K*_ass_ value of ethanolamine, which is half an order of magnitude below that of noradrenaline (**80b**), shows that the receptor molecule clearly recognizes the hormone’s catechol ring. This is supported by the decrease in binding energy when the phenolic hydroxyl groups are missing from the guest structure (**78a**, 2-phenylethylamine).

All the effects discussed above confirm that the macrocyclic host **184** recognizes adrenaline derivatives in mixtures of water and methanol (1:1) by multiple non-covalent interactions including electrostatic attraction, hydrogen bonds, π-stacking, and hydrophobic forces.

The nitro-arene groups in the macrocyclic receptor molecule can undergo double π-stacking interactions with the catechol ring of adrenaline without producing any significant ring strain in the receptor molecule, whilst the isophthalic amide group is ideally pre-oriented to form hydrogen bonds to the phenolic OH groups.

**Figure 133:**
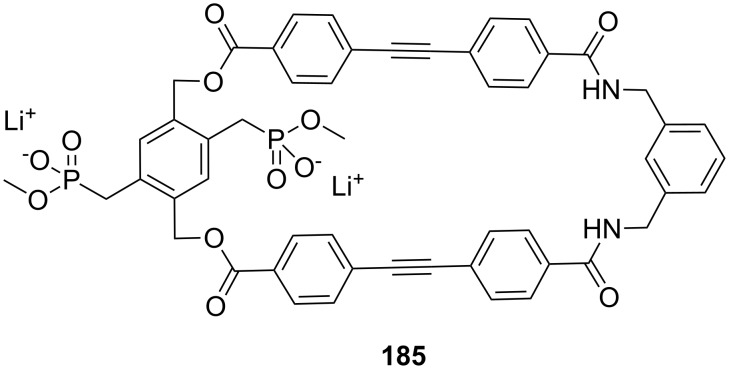
Receptor **185** for binding of noradrenaline on surface layers from Schrader et al.

Schrader et al. introduced a similar system **185** for the detection of adrenaline and related biologically important amines [612] (Figure 133). Various amines, such as ethanolamine and propranolol bind to the receptor in methanol with low selectivity. The values of the binding affinities vary between 700 and 1600 M^−1^. However, the insertion of **185** into a mono-layer of stearic acid at the air-water interface leads to selective noradrenaline (**80b**) binding (10^5^ M^−1^). The binding is monitored by changes in the pressure dependent surface area diagrams with the Langmuir film balance. The drastic change in comparison to solution is explained by the forced inclusion of the guests in the cavity of the receptor on the surface, and the formation of new hydrogen bonds between the NH of **185** and the phenolic oxygen of the noradrenaline. Other catecholamines do not show this effect.

**Figure 134:**
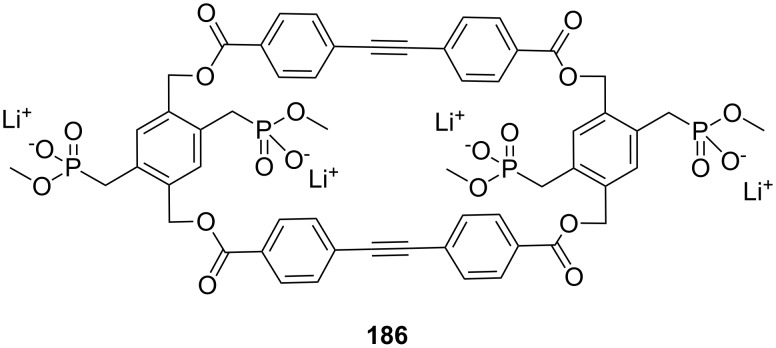
Tetraphosphonate receptor for binding of noradrenaline.

A slight variation of the receptor, the introduction of a second bisphosphonate moiety (Figure 134), resulted in high affinity towards catecholamines in water, especially for structures with extended aromatic π-faces as found in many β-blockers (up to 7 × 10^3^ M^−1^ for each single complexation step or 5 × 10^7^ M^−1^ for both steps). Job’s plot analyses showed a 2:1-stoichiometry, NMR titrations revealed no co-operativity in any case. For ease of comparison, the authors always used 1:1 association constants for each single binding step and varied the solvent polarity from pure methanol to methanol/water (1:1) to pure water. Here, the recognition profited from the amphiphilic structural design [613] and even more from the extensive self-association by the aromatic π-planes. Affinity and selectivity towards adrenergic receptor substrates was greatly enhanced if the receptor molecule **186** was transferred from water into a lipid monolayer. Above the critical micelle concentration of 3 × 10^4^ M, the host formed micelles that produce a favorable microenvironment for hydrophobic attraction of the ammonium alcohol by the phosphonate anions, combined with hydrophobic contributions between the aromatic moieties. Ionic hydrogen bonds with the polar OH or NH groups of the guest enforced the non-covalent interactions, and finally led to increased specificity. Especially β-blockers with minute structural changes can be easily distinguished from each other. A remarkable dependence of the 1:1 binding constant was revealed for noradrenaline. The binding amounts to 4000 M^−1^ in MeOD, fell to ~700 M^−1^ in MeOD/D_2_O (1:1), but increased to 1200 M^−1^ in water.

For further and more detailed discussion of the interesting topic of recognition of catecholamines with artificial receptors in aqueous solution, we refer the reader to a recent overview [614].

Bell’s receptors **155** (Figure 112) can bind free arginine (**81d**) with a *K*_ass_ value of 900 M^−1^, another binds lysine derivatives with a millimolar binding strength. The tetrasulfonate calixarene hosts (**84**) reach 1500 M^−1^ in borate buffer (see chapter 4); in calixarenes **92**, the phosphonate groups are responsible for the major contribution to binding and selectivity. Following these examples and the survey of molecules given above, this shows that by adding more phosphonate groups to a rigid scaffold, binding strength and selectivity are increased. Indeed, by virtually “dimerizing” clefts, cyclic moieties like cyclophanes result, which have suitable cavities and substitution patterns for a selective artificial ammonium ion receptor. These molecules bind strongly to bis-ammonium guests in even more polar solvents.

**Figure 135:**
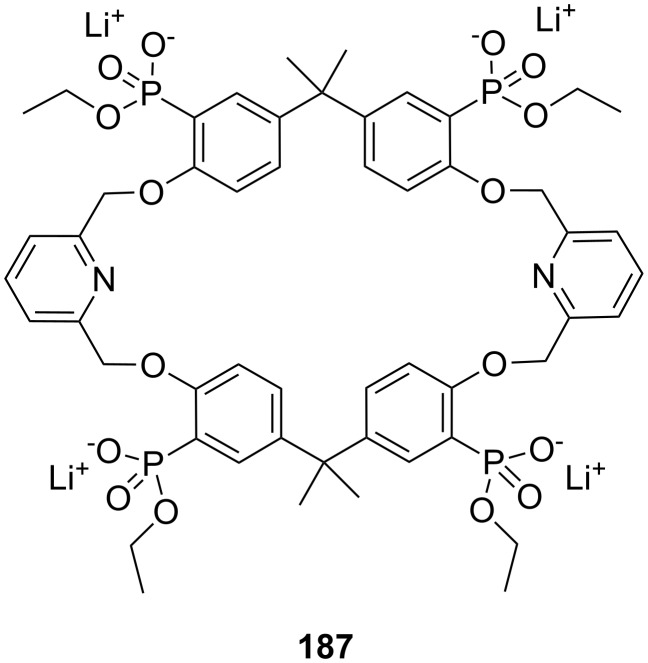
Tetraphosphonate **187** of Schrader and Finocchiaro.

The further development of receptor **167** led to the tetraphosphonate (**187**) [615] (Figure 135). By doubling the number of phosphonate groups binding increases, so that the receptor can be used in water. X-ray analysis and molecular modeling revealed that the host adopts a favorable open conformation [616]. Typical stoichiometries with diammonium amino acids are 1:2; only lysine (**81c**) forms a 1:1 complex. Table 11 summarizes the results.

**Table 11:** Binding constants for the complexes of **187** with different amino acids.

Amino acid (dihydrochlorides)	*K*_ass_ [M^−1^] (methanol)	*K*_ass_ [M^−1^] (water)	Receptor: guest stoichiometry

His	29000	650	1:2
Orn	9500	221	1:2
Arg	8800	165	1:2
Lys	21000	1200	1:1

In methanol all amino acids are bound strongly in a double chelate binding mode. The exceptionally good binding of histidine (**81e**) is explained by a chelate complex, which includes both imidazole nitrogen atoms in addition to the amino acids ammonium functionality. From methanol to water, the stoichiometry of all complexes is retained, but a 20–50 fold drop is observed in the association constants of the four investigated amino acids attributed to the competition of the water molecules. Lysine (**81c**) is complexed 5–7 times more strongly than ornithine and arginine (**81d**) and even twice as strongly as histidine (**81e**). The contribution of hydrogen bonds in water is negligible, while electrostatic interactions represent the major attractive force. It is known, that in this respect the hard ammonium ion with its high charge density is superior to the softer guanidinium and also the imidazolium ion, where the positive charge is delocalized across several atoms [617]. The electrostatic attraction exerted by the second ammonium functionality of lysine (**81c**) is stronger than that of arginine’s guanidinium ion and even histidine’s imidazolium ion. In addition, lysine (**81c**) is in the position to undergo a four-point interaction in its complex with **187** which is stronger than the two-point interaction in the related assemblies with ornithine and arginine (**81d**).

Charged clefts have previously been discussed. A similar class, quite related to the hosts presented in this chapter are cavitands or macrocycle bearing phosphate and phosphonate groups. The negative charged phosphorus derivatives are closely comparable to the carboxylate residues mentioned above. In combination with cavitands structures and/or molecular clefts e.g. tweezer backbones, they are employed with great benefits for ammonium ion recognition.

Extensive hydrophobic interactions with a self-associated or self-organized microenvironment and utilising a combination of van der Waals interactions and substantial electrostatic contributions for locking of the guest are responsible for the observed high efficiency and specificity found in clefts and cavitands. Often electrostatic interactions contribute most to the stabilization energy in the complexes. In larger cavities the loss of one hydrogen bond can be overcompensated by, e.g., hydrophobic interactions. Optimized host structures implementing elements of much higher rigidity can achieve more effective pre-organization and desolvation.

In summary, *C*_3_*_v_* symmetric tripods, tweezer ligands and pre-organized molecular clefts reach selectivities and affinities in ammonium ion binding which compete with naturally occurring recognition motifs such as nonactin or valinomycin [618].

### 6. Porphyrins and other metal complexes for ammonium ion recognition

In this part of the review we will discuss ammonium ion recognition involving metal complexes. Metal complexes are important binding sites for amines, but have even more extensively been used for amino acid recognition. In fact, the following examples typically involve simultaneous binding of ammonium and carboxylate ions. Discussion of amino acid zwitterion binding by metal complexes has been added to supplement our survey, although ammonium ion recognition is only part of the binding process.

#### 6.1. Porphyrins

Porphyrins and their metal complexes play a fundamental role in a variety of biological processes, for example, the chlorophylls as photoreaction centres in photosynthesis, haemoglobin as the oxygen carrier in blood and myoglobin for oxygen storage in muscles, cytochromes in electron-transfer processes in respiration or as important prosthetic groups and coenzymes as found in vitamin B12 [619]. They have been employed as electroactive materials for molecular electronics [620], effective photosensitizers [621] for photodynamic therapy or as supramolecular building blocks for energy conversion devices [622] and dye sensitized solar cells [623]. The synthesis and properties of porphyrins and related compounds, such as porphycenes or texaphyrins, have been extensively reviewed in several books and articles [619,624–625].

Porphyrins have been widely used for the recognition of various guest molecules [626–629]. Two reviews on their general properties and recognition scope have been published [630–631]. Articles on the related porphyrinoid [632–633], and chiral multifunctional porphyrins [634] have been reviewed. We will focus in the following section on examples of porphyrin based receptors for amines or ammonium ion recognition.

**Figure 136:**
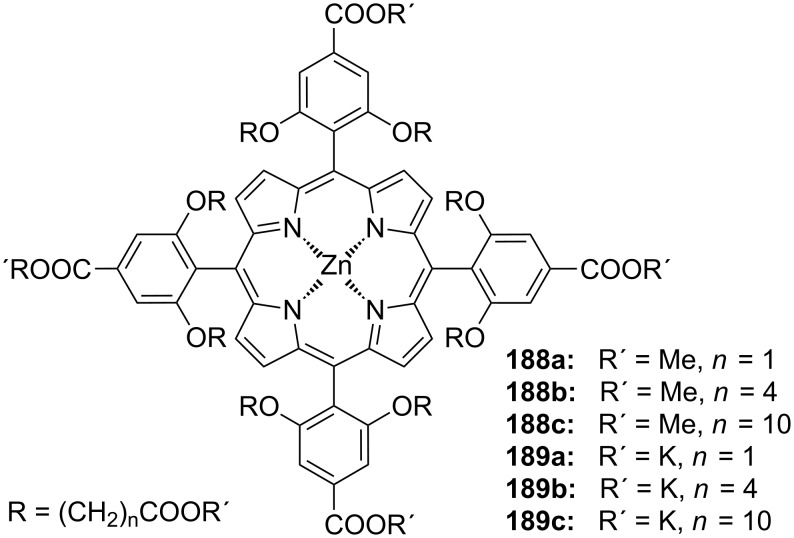
Zinc-Porphyrin ammonium-ion receptors **188 **and **189** of Mizutani et al.

Zinc porphyrin receptors bearing 12 ester groups in the meso phenyl groups [635] and the corresponding water soluble potassium carboxylates [636] (Figure 136) are selective receptors for amines, amino acid esters and oligopeptides as demonstrated by UV–vis experiments in dichloromethane and buffered aqueous medium. Using small substituents as in **188a** or the unsubstituted parent compound, butyl ammonium chloride or phenethylamine hydrochloride (up to 52700 M^−1^ in dichloromethane) bind with highest affinity. The ester groups of **188a** assist the binding of aromatic *R*-amino esters (*K*_ass_ = 8000–23000 M^−1^) in this medium and inhibited the binding of bulky aliphatic *R*-amino esters (*K*_ass_ of 460 M^−1^ for Leu-OMe). This indicated that CH–π-type interactions and steric repulsions control the selectivity. The corresponding salts **189** showed a good selectivity for binding of hydrophobic guests: **189c** binds Trp-OMe or pyridine in water with binding constants of 7000–8000 M^−1^. These anionic zinc porphyrins bind histamine (**1**) and a histidine-containing oligopeptide even more tightly. The highest binding strength for histamine was found for **189a, 189b** and **189c** in pH 8 buffer with binding constants of 157000, 31000, and 18200 M^−1^, respectively. Co-ordination of the imidazole to the zinc centre and a significant electrostatic interaction between the ammonium group of histamine and the carboxylate groups of receptor stabilizes these complexes. In a series of amino acid esters, receptor **189a** co-ordinated best to the cationic Arg-OMe, with an enthalpically driven binding of 11000 M^−1^. Strong dependence of the binding affinity on ionic strength and pH revealed that electrostatic interactions between charged functional groups are an important driving force for recognition of hydrophilic guest molecules in water. Comparisons of binding affinity between hydrophilic receptor **189a** and hydrophobic receptor **189c** revealed that the hydrophobic binding pocket of **189c** enhanced the affinity in water towards hydrophobic guests. A lower affinity of the receptors in methanol-water than in water indicated that water plays a significant role in binding energetics.

**Figure 137:**
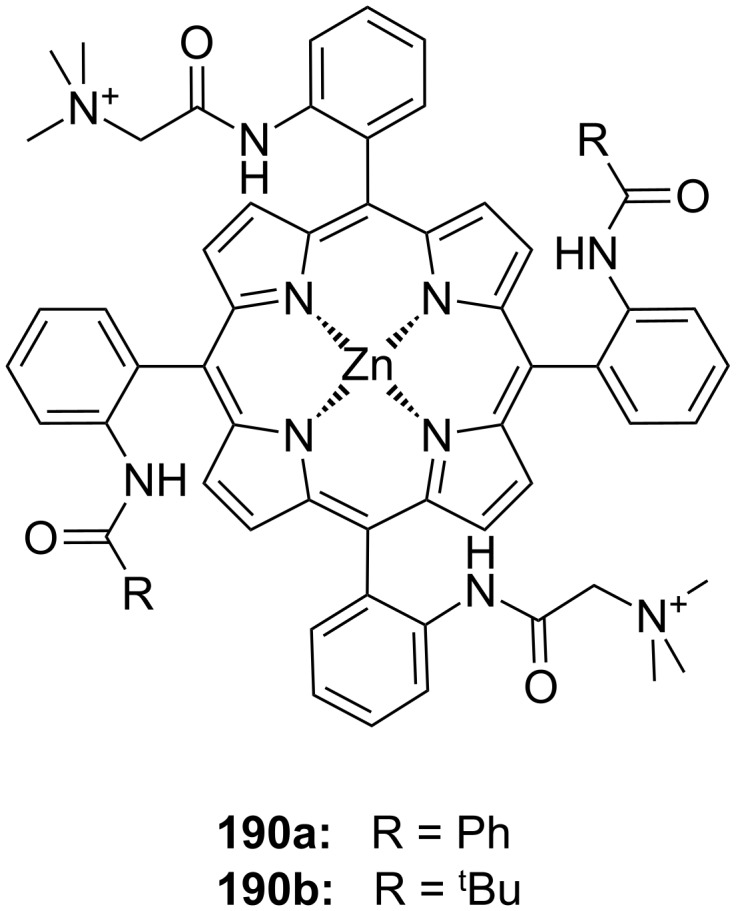
Zinc porphyrin receptor **190**.

Imai et al. also employed highly charged water-soluble zinc porphyrins (Figure 137). With an ammonium group and a phenyl or tertiary butyl group above each porphyrin plane, they recognize amino carboxylates in aqueous solution [637]. Binding constants were determined spectrophotometrically in aqueous carbonate buffer at pH = 10.4 and revealed the maximum binding strength for *rac*-tryptophan (**81b**) with 1000 M^−1^ for **190a** and 830 M^−1^ for **190b**. The authors suggest a three point recognition for amino carboxylates by co-operative co-ordinative, coulombic, and hydrophobic interactions.

**Figure 138:**
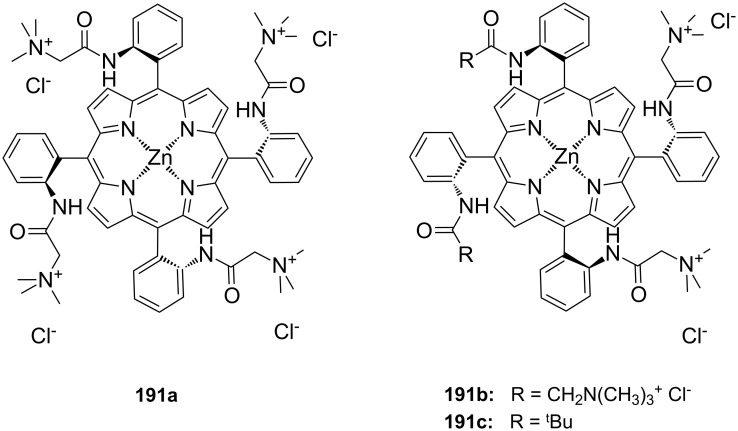
Zinc porphyrin receptors **191** capable of amino acid binding.

The binding of amino acids to water-soluble zinc porphyrins in basic aqueous solution was spectrophotometrically analyzed with similar receptors (**191**) [638] (Figure 138). The amino acids were bound to the porphyrins through the co-ordination of the N atom with the central zinc ion. Additional stabilization of the aggregate comes from coulombic interactions between the –COO^−^ anion of the amino acids and the –N^+^(CH_3_)_3_ cation of the porphyrin substituents, and the hydrophobic interactions between the porphyrin plane and the hydrophobic substituents of the amino acids. In the study, the binding of amino acids (10^2^ M^−1^) is apparently stronger than that of aminoethanol (10 M^−1^), due to additively co-operated coulombic interaction between the cation substituent(s) of porphyrins and the carboxylate anion of amino acids. This explanation is supported by the fact that the *K*_ass_ values increase as the number of possible coulombic interactions increases: the *K*_ass_ values for amino acids for **191a** and **191b** are approximately two times larger than those for **191c**, and the binding of *S*-Asp (*K*_ass,191a_ = 780 M^−1^ and *K*_ass,191b_ = 770 M^−1^) and *S*-Glu (*K*_ass,191a_ = 390 M^−1^ and *K*_ass,2_ = 540 M^−1^) is enhanced compared to that of Gly (*K*_ass,191a_ = 110 M^−1^ and *K*_ass,191b_ = 150 M^−1^). Co-ordination of the aromatic amino acids Phe (*K*_ass,191a_ = 320 M^−1^ and *K*_ass,191b_ = 180 M^−1^) and Trp (*K*_ass,191a_ = 1300 M^−1^ and *K*_ass,191b_ = 770 M^−1^) is strengthened by hydrophobic interactions between the phenyl or indole group of the amino acids and the porphyrin plane, which is also supported by observations of the relevant peak shifts by ^1^H NMR in Na_2_CO_3_ buffered D_2_O.

The coulombic interactions between dipeptides and porphyrins are comparable to those between amino acids and porphyrins. The *K*_ass_ values of Gly-*S*-Phe (*K*_ass,191a_ = 200 M^−1^ and *K*_ass,191b_ = 340 M^−1^ and *K*_ass,191c_ = 240 M^−1^) and Gly-*S*-Trp (*K*_ass,191a_ = 770 M^−1^ and *K*_ass,191b_ = 1100 M^−1^ and *K*_ass,191c_ = 780 M^−1^) are larger than those of Gly-Gly (*K*_ass_ ~ 100 M^−1^), indicating that the interactions between these dipeptides and the porphyrins are similar to those between *S*-Phe and *S*-Trp and porphyrins.

The molecular recognition of amino acid esters in CHCl_3_ was investigated by UV–vis titration with *S*-tyrosine- [639] and *S*-threonine [640] substituted chiral zinc porphyrins (**192**). The association constants of the molecular recognition reactions were all *K*_R_ > *K*_S_ and followed the order of *K*(PheOMe) > *K*(LeuOMe) > *K*(ValOMe) > *K*(AlaOMe) in host **192a** and *K*(ThrOMe) > *K*(LeuOMe) > *K*(ValOMe) > *K*(AlaOMe) > *K*(PheOMe) in host **192b**. All the results are summarized in Table 12.

**Table 12:** Binding constants and enantiomeric distinction factors of chiral porphyrin-amino-acid dipeptide receptors in chloroform at 20 °C.

Guest	*K*_ass_ [M^−1^], **192a**	*K_R_*/*K_S_*	*K*_ass_ [M^−1^], **192b**	*K_R_*/*K_S_*

*S*-Ala-OMe	320	1.4	155.2 ± 12	3.1
*R*-Ala-OMe	450		488.6 ± 20	
*S*-Val-OMe	621	1.2	175.2 ± 10	2.9
*R*-Val-OMe	713		502.2 ± 15	
*S*-Leu-OMe	1030	1.2	179.8 ± 13	4.9
*R*-Leu-OMe	1290		881.5 ± 22	
*S*-Phe-OMe	679	2.2	420.7 ± 10	1.1
*R*-Phe-OMe	1490		442.3 ± 10	
*S*-Thr-OMe	n.d.	n.d.	537.6 ± 15	2.6
*R*-Thr-OMe	n.d.		1391.3 ± 25	

**Figure 139:**
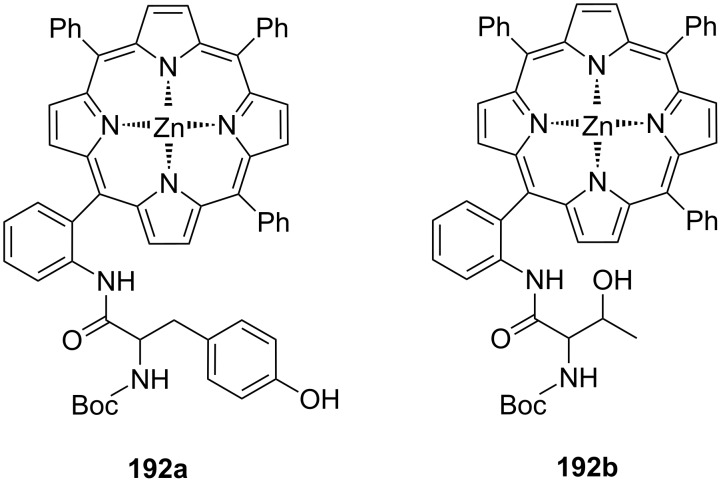
Zinc-porphyrins with amino acid side chains for stereoinduction.

A significant contribution of π–π-interaction can be observed for the binding of phenylalanine (**81a**) to receptor **192a**, as is also evident by comparison to the second system with a threonine side chain (**192b**) (Figure 139). Here the binding constant for the aromatic amino acid is the lowest in the series.

Circular dichroism spectra were used to explain chiral molecular recognition. It was found that chiral recognition arose mainly from the chiral matching between host and guest. The enthalpy–entropy compensation relationship revealed a significant conformational change during the process of chiral recognition. The induced CD spectra of the complexes exhibited characteristic Cotton effects. The authors proposed that the induced CD spectrum was caused by the coupling between the electric transition moment (the π–π*-transition) of the carbonyl group in Boc-*S*-Tyr side chain and that of the porphyrin. The molecular recognition process of this host–guest system was confirmed by quantum chemical methods. The result was a structure where the *R*-enantiomer was more tightly bound with a better steric fit to the host than its enantiomer. By comparison minimal energy conformations, it was evident that host *R*-AlaOCH_3_ has lower energy than host *S*-Ala-OCH_3_, indicating that the former was more stable than the latter.

Porphyrin dimer- or tweezer-systems have been successfully used to determine the stereochemistry of chiral amines [641–642], alcohols [643–644] and carboxylic acids [645–647].

The principle advantage of the porphyrin tweezer system resides with the non-covalent binding of the chiral guest and the stereoinduction by the two asymmetrically linked metal-co-ordination centres.

**Figure 140:**
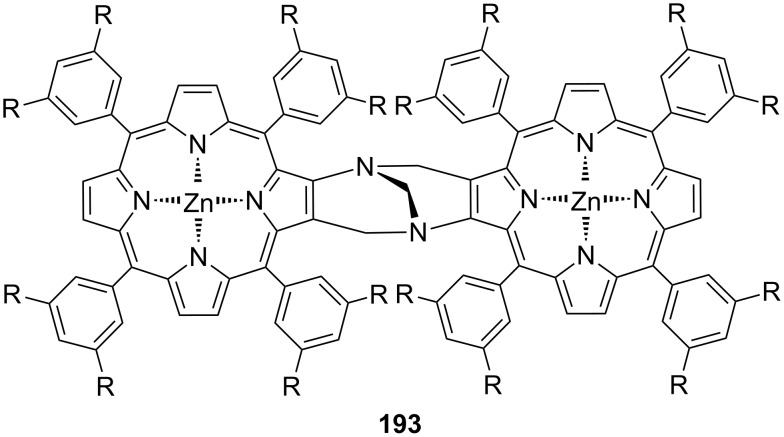
Bis-zinc-bis-porphyrin based on Tröger’s base **193**.

Crossley and his co-workers have reported a bis-zinc(II)-bis-porphyrin Tröger’s base analogue (**193**) (Figure 140) as a host molecule for diamines [648] and for the chiral recognition of histidine and lysine esters [649]. The X-ray crystal structure of the analogous palladium bis(tetraphenylporphyrinato) complex reveals a concave chiral cavity with two metal ion binding sites suitable for ditopic interactions with guest molecules.

Several α,ω-diamines (H_2_N–(CH_2_)*_n_*–NH_2_) are strongly co-ordinated with a certain preference for *n* = 2–4 and *K*_ass_ ~ 2 × 10^8^ M^−1^ as measured by spectrophotometric titrations in toluene. With increasing chain length, the affinity starts to decrease with *K*_ass_ ~ 6.1 × 10^7^ M^−1^ and *K*_ass_ ~ 3.7 × 10^7^ M^−1^ for 1,5-diaminopentane and 1,6-diaminohexane, respectively. Monoamines, such as hexylamine are less strongly bound (*K*_ass_ ~ 5.1 × 10^4^ M^−1^).

The tweezer can be resolved on a small scale by chromatography on a silica – *S*-histidine benzyl ester support [650]. Resolution of the bisporphyrin Tröger’s base analogue **193** affords homochiral clefts that tightly bind histidine esters with 80–86% *ee* and lysine benzyl ester with 48% *ee*. The histidine esters are bound in fixed conformations that can be readily detected by ^1^H NMR spectroscopy as a result of the large dispersion of proton resonances by the ring currents of the two porphyrins. The binding constants are in the same order of magnitude as observed previously for diamines.

**Figure 141:**
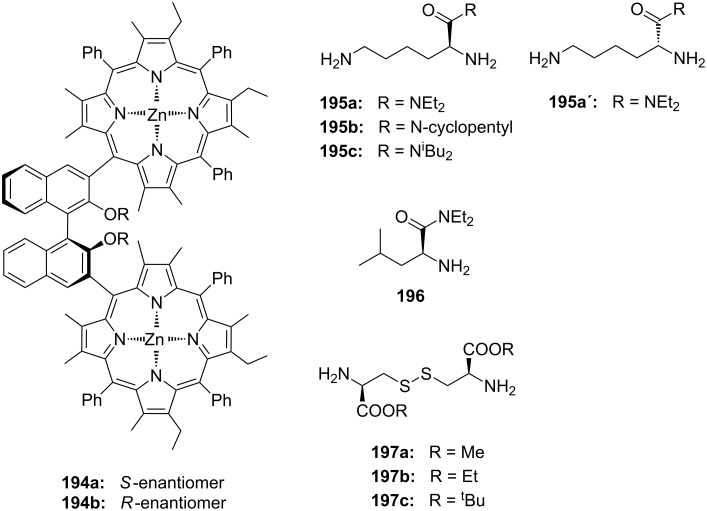
BINAP-zinc-prophyrin derivative **194** and it’s guests.

A zinc porphyrin dimer (**194**) linked by the chiral 1,1′-binaphthyl derivative (Figure 141) shows a size specific interaction with α,ω-diamines (H_2_N–(CH_2_)_n_–NH_2_) [651]: The zinc complex binds α,ω-diamines H_2_N–(CH_2_)*_n_*–NH_2_ (*n* = 6, 8, 10, 12; *K*_ass_ = 5 × 10^5^–2 × 10^6^ M^−1^ in CH_2_Cl_2_) with preference for *n* = 6 and 8. Shorter guests such as ethylenediamine or monoamines such as *n*-butylamine gave binding constants (*K*_ass_ ~ 3 × 10^3^ M^−1^) comparable to the co-ordination of alkylamine guests to the corresponding zinc porphyrin monomer (*K*_ass_ = 2.2 × 10^3^ M^−1^). These complexes gave characteristic CD spectra due to exciton coupling of the two zinc porphyrins. Their intensity depends on the length of diamine. The CD spectrum in the complex reflects the angle and flexibility of the chiral twist between two zinc porphyrin units.

The chiral zinc porphyrin dimer linked by (*R*)-2,2′-dimethoxy-1,1′-binaphthyl (**194**) (Figure 141) not only tightly binds diamines via a zinc–nitrogen co-ordinated ditopic interaction, it displays a prominent enantioselectivity for several lysine derivatives (Table 13) [652]. The enantioselectivity obtained is one of the best for chiral zinc-porphyrin recognition systems. In particular, the D/L selectivity is determined to be 11–12 for lysine derivatives, as also demonstrated by CD-spectroscopy.

**Table 13:** Binding constants and enantiomeric distinction factors for chiral porphyrin-dimers **194** in dichloromethane.

Host	Guest	*K*_ass_ [M^−1^]	*K_S_*/*K_R_*

(*S*)-**194**	**195a**	160000	12
(*R*)-**194**	**195a**	13000	
(*S*)-**194**	**195a′**	14000	11
(*R*)-**194**	**195′**	150000	
(*S*)-**194**	**195b**	120000	8.6
(*R*)-**194**	**195b**	14000	
(*S*)-**194**	**195c**	120000	11
(*R*)-**194**	**195c**	11000	
(*S*)-**194**	**196**^a^	1200	1.2
(*R*)-**194**	**196**^a^	980	

^a^for 1:1 complex formation.

**Figure 142:**
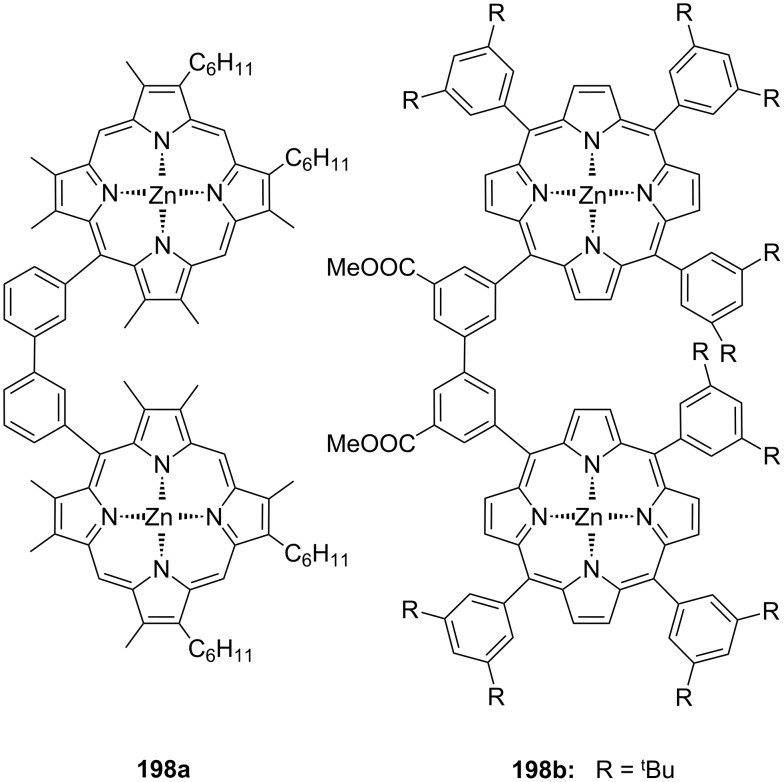
Bisaryl-linked-zinc-porphyrin receptors.

Two different achiral hosts (Figure 142) were investigated for their binding properties to the same guests in the course of the study. Titration in dichloromethane monitored by UV–vis titration demonstrated a 1:1 complexation between the zinc-porphyrin dimers and the amino acid derivatives **195** and **197**. Compared to **198a** (*K*_ass_ for **195** = 1–8 × 10^5^ M^−1^, *K*_ass_ for **197** = 1–4 × 10^5^ M^−1^), the zinc porphyrin dimer **198b** has higher affinity for cysteine derivatives. The binding constants of **198b** for **197a** and **197b** were determined to be 1.7 and 2.4 × 10^6^ M^−1^, respectively. The length of both amine-guests almost fits the Zn-to-Zn distance, leading to the strongest binding, consistent with the former study of **194** versus diamines. The other values range from 3 to 5 × 10^5^ M^−1^. The achiral zinc porphyrin dimers linked by a biphenyl unit exhibit a significantly induced CD in the Soret region in the presence of chiral diamines such as lysine amides and cysteine diesters, indicating that the chirality of the amino acid derivatives can be monitored by complexation to the achiral zinc-porphyrin dimer.

**Figure 143:**
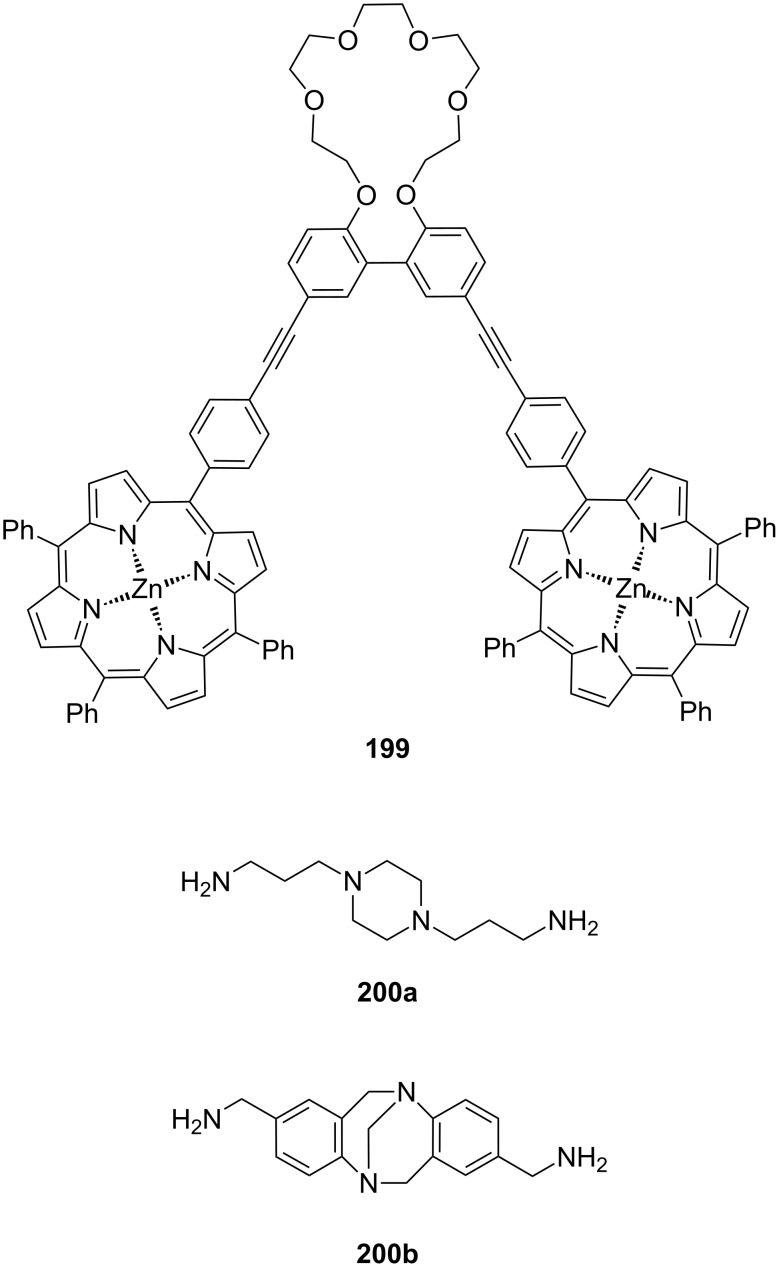
Bis-zinc-porphyrin **199** for diamine recognition and guests.

Kubo et al. developed a bis-porphyrinic system coupled with biphenyl-20-crown-6 as an allosteric spacer [653–654]. The biphenyl unit is connected by a rigid spacer to the two porphyrins and bridged with a crown-ether (Figure 143). The porphyrin centre-to-centre distance can be switched by Ba^2+^ ion complexation in the crown-ether cavity. In its concave conformer, **199** can bind a diamine guest, such as 1,4-bis(3-aminopropyl)piperazine (**200a**). UV–vis titration in CH_2_Cl_2_/CH_3_CN 9:1 confirmed 1:1 complex formation and a binding constant (*K*_ass_) of 7.9 × 10^5^ M^−1^. In addition, the chiral bis-amino guest Tröger’s base **200b** was used to probe an anti-co-operative binding event. Due to the axial chirality, **199** existed as two chiral atropisomers that rapidly interconvert at room temperature as evidenced by CD measurement. The binding of the chiral base transferred its chirality to the host upon complexation.

**Figure 144:**
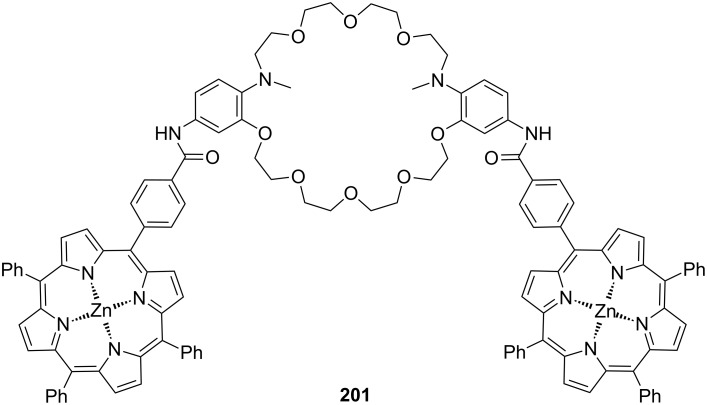
Bis-zinc-porphyrin crown ether **201**.

Another example from the same group also demonstrated this for the chiral induction with a crown-ether bis-zinc-porphyrin combination (**201**) (Figure 144). Upon complexation of a chiral sodium carboxylate by the flexible dibenzo-30-crown-10 ether, the topology was changed into a tweezers-like structure [655] and gave a ditopic chiral guest binding site. Circular dichroism (CD) spectroscopy revealed a chiral screw conformation, which interacted with various chiral diamines, for example, *N*,*N*-dimethylcyclohexane-1,2-diamine.

This chiral induction by a ditopic bound guest was employed to determine the absolute configurations of diamines, amino acids and amino alcohols by exciton-coupled circular dichroism (ECCD).

**Figure 145:**
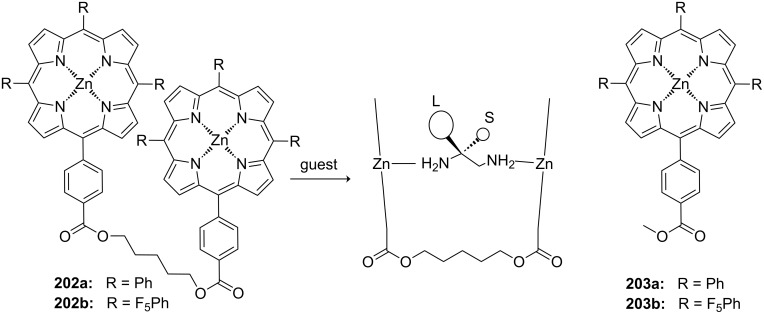
Bis-zinc-porphyrin **202** for stereodiscrimination (L = large substituent; S = small substituent).

The achiral chromophoric host porphyrin tweezer **202a** [641] or its electron deficient fluorinated analogue **202b** [656] (Figure 145) both bind to an acyclic chiral diamine through nitrogen/zinc co-ordination to form a macrocyclic host guest complex with a CD spectrum, that reflects the absolute configuration of the diamine. The exhibited exciton-coupled bisignate CD spectra reveal predictable signs based on the substituents at the chiral centre. The absolute stereochemical determination of both *threo* and *erythro* systems without the need for chemical derivatization is thus possible.

This method can be extended to amino acids and amino alcohols after simple chemical modifications. With the fluorinated system **202b**, the absolute configurations of *erythro* and *threo* diols could be also effectively determined. Binding of diols to the porphyrin tweezer system is greatly enhanced by increasing the Lewis acidity of the metalloporphyrin by the strong electron withdrawing effect of the fluorine substituents.

The binding constants to amino- and hydroxy-functionalities were determined for the monoesters (**203**) by UV–vis titration. For isopropanol as the guest *K*_ass_ = 2140 and 50 M^−1^ and for isopropylamine *K*_ass_ = 473000 and 11400 M^−1^ are observed for the fluorinated porphyrin **203b** and the triphenyl substituted compound **203a**, respectively.

**Figure 146:**
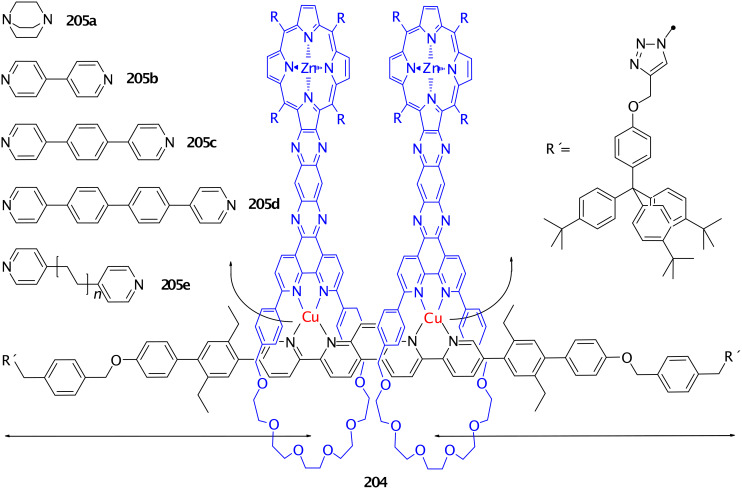
Bis-zinc-porphyrin[3]rotaxane and its copper complex and guests.

A [3]rotaxane and its copper complex (**204**) have recently been presented as a binding concept [657] (Figure 146). The properties of the system were investigated by UV spectroscopy in toluene. The complexes were also investigated and assigned by NMR DOSY experiments. In these two states of the [3]rotaxane, free and complexed with copper, the two zinc(II) porphyrins attached to the rings can bind different ditopic guests bearing pyridyl groups or amines as terminal functions.

Removal of the two Cu(I) cations releases the two rings which are now free to move along and around the thread. The metal-free [3]rotaxane is a new type of receptor by which guests of very different sizes can be trapped between the two mobile porphyrins since they can move over an 80 Å plane-to-plane distance on the thread. It is both a strong and highly adaptable receptor with high stability constants for the host/guest complexes, log *K*_ass_ being in the range of 6.3 to 7.5 for guests between 2.8 and 18 Å.

In the copper-complexed [3]rotaxane, the rings are fixed by co-ordinative bonds to the rod and the distance between the porphyrins is therefore controlled to a certain extent, leading to destabilization of the host/guest complex with long guests, due to distortions on both the guest and the porphyrin rings. The copper-complexed [3]rotaxane is a good receptor for small guests with preference for **205c** (log *K*_ass_ = 7.5) due to an entropic gain for this pre-organized molecule compared to the free [3]rotaxane.

#### 6.2. Other metal complex centres

Due to their strong complexing ability, many other co-ordinatively unsaturated metal complexes, can be employed as suitable potential binding sites for synthetic receptors, especially for molecular recognition in protic solvents [658]. Non-covalent forces are weakened in this medium with high dielectric constant, since a large number of solvent molecules interfere. The selection of the ligands is defined by the ability of their corresponding transition metal complexes to reversibly and tightly bind Lewis basic guest molecules in competing solvents, such as water. Amino acids are strongly bound by their side chains or in a bidentate complex bridging the metal. Complexes of cyclene, cyclam and related structures are widely used. The recognition with aza macrocycle complexes was recently reviewed [659].

Amino acids can be targeted by co-operative chelation between the carboxylate and the amine: The co-ordination of metal ions through the amino and carboxyl groups gives five-membered metallocycles [660]. Bipyridines (bpy) or nitrilotriacetic acid (NTA) are widely used as ligands. A typical example are [Cu(NTA)]-complexes, which co-ordinate amino acids [661–662]. Binding affinities have been determined for a variety of amino acids in aqueous medium (Table 14). The co-ordination of His to [Cu(NTA)] is a special case, containing mixtures of species in which His is co-ordinated either as an anion or in its zwitterionic form [663].

**Table 14:** Binding constants of amino acid guests to Cu[NTA].

Amino acid	log *K*_ass_^a^

Gly	5.44
Ala	5.42
Phe	4.99
Leu	5.35
Val	5.10
β-Ala	4.56
His	4.16 (monodentate) 5.73 (bidentate)

^a^Standard deviation <0.01; at 25 °C.

**Figure 147:**
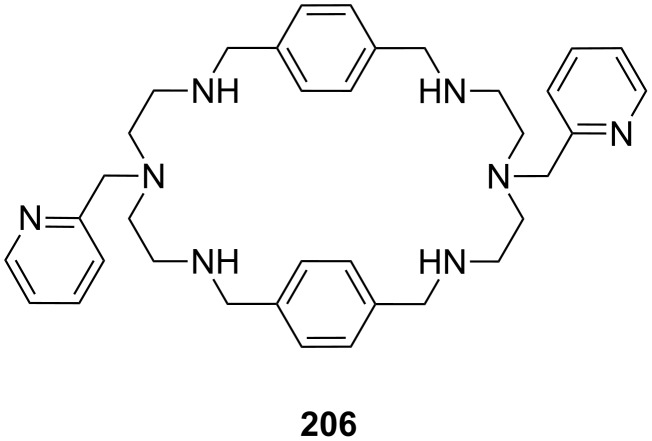
Dien-bipyridyl ligand **206** for co-ordination of two metal atoms.

Bis-dien bis-copper complexes of ligand **206** (Figure 147) bind imidazole as bridging ligand between two Cu(II) ions with the simultaneous extrusion of a proton as demonstrated by Fabbrizzi et al. [664]. A binding constant of log *K*_ass_ = 4.7 was derived by pH titration. For histamine a binding of log *K*_ass_ = 4.3 was obtained and for *S*-His the value of log *K*_ass_ was 5.5. The 1:1 complex stoichiometry was verified by spectrophotometric titrations. Later the same group reported a luminescent sensor for histidine (**81e**) based on a tridentate Zn(II)-tren complex [665].

**Figure 148:**
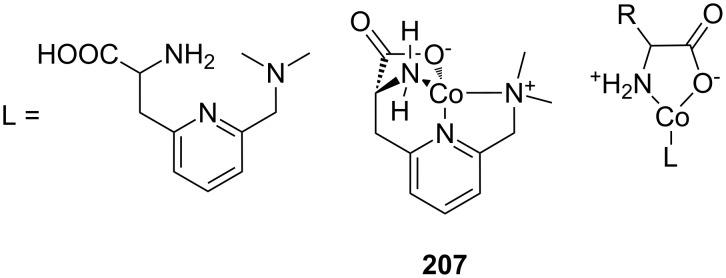
The ligand and corresponding tetradentate co-complex **207** serving as enantioselective receptor for amino acids.

The dichloro-cobalt-complex **207** (Figure 148) was reacted with glycine, *S*-alanine, *R*-alanine, *S*-phenylalanine and *R*-phenylalanine (**81a**), *S*-tryptophan and *R*-tryptophan (**81b**) [666]. Alanine forms a five-membered ring upon chelation to the metal complex. Deuteration experiments monitored by NMR showed that α-hydrogens of the three co-ordinated *R*-amino acids exchanged rapidly with little or no observable epimerization. In contrast, the α-hydrogens of the three *S*-amino acids exchanged slowly with concomitant epimerization. It was not possible to fully deuterate the *S*-amino acid complexes due to competing decomposition reactions. Thus, the *R*-enantiomer of the receptor binds the *R*-enantiomers of the amino acids more tightly and converts the *S*-enantiomers to the *R*-enantiomers.

The X-ray crystallographic and ^1^H NMR data underlined that co-ordination of alanine takes place with unprecedented regiospecicity and stereospecicity. The regiospecicity is controlled by electrostatic effects while the stereospecicity is controlled by steric effects in a highly predictive manner. This approach thus provides detailed structural insights into general separation of bidentate α-H-amino acids into *R*- and *S*-forms with a single chiral metal complex.

**Figure 149:**
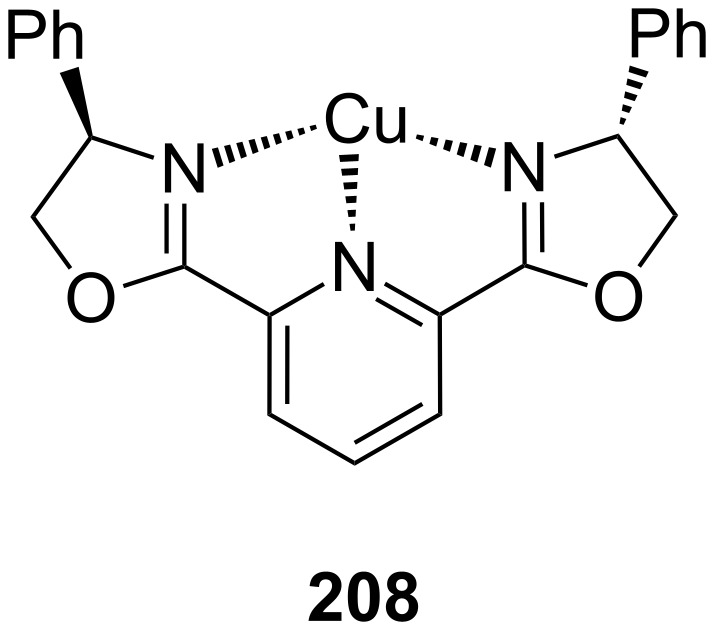
Bis(oxazoline)–copper(II) complex **208** for the recognition of amino acids in aqueous solution.

Bis(oxazolines) are widely employed in asymmetric catalysis, for example, in cyclopropanations. Besides this they are also valuable receptor moieties [667]: The enantioselective recognition of amino acids has been studied with *C*_2_ symmetric chiral pyridine bis(oxazoline)–copper(II) complex **208** (Figure 149) at physiological pH by UV–vis titration and revealed a strong binding with a submillimolar dissociation constant in aqueous solution. Moderate selectivity of up to 2:1 between *R*- and *S*-amino acids was achieved with best affinities of the *R*-host to *R*-amino acids.

Zinc–salophen complexes have also attracted much attention as receptors. Their well known capability to accept one axially co-ordinated donor species, along with their photophysical properties [668–670], make them suitable candidates for the development of amine receptors [671].

**Figure 150:**
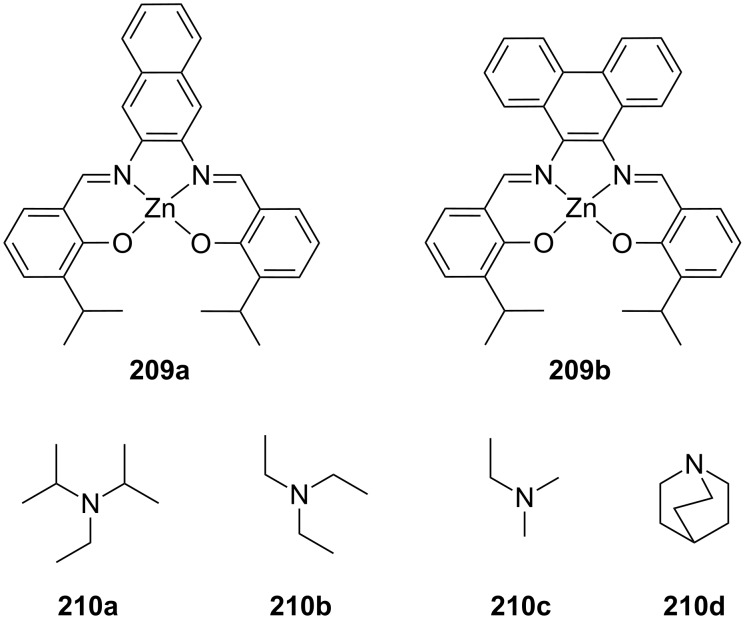
Zinc-salen-complexes **209** for the recognition tertiary amines.

Zinc–salophen compounds incorporating 2,3-diaminonaphthalene (**209a**) and 9,10-diaminophenantrene (**209b**) (Figure 150) moieties show unprecedented selectivities of quinuclidine (**210d**) vs. triethylamine (**210b**) higher than 10^5^ as investigated by UV–vis and fluorescence spectroscopy in chloroform solution [672]. The binding to the zinc–salophen compounds to tertiary amines is influenced by steric effects. The binding constants for quinuclidine (**210d**) were all larger than 10^6^ M^−1^, for triethylamine (**210b**) values of ~50 M^−1^ and smaller were recorded. Dimethylethylamine (**210c**) has an affinity of 1500 to 1900 M^−1^, while diisopropylethylamine (**210a**) gave a negligible response. The axial co-ordination of tertiary amines is in general stronger for zinc-salophen compounds than for zinc-porphyrins. X-ray diffraction showed that in the solid state compound **209a** is dimeric, but its 1:1 quinuclidine complex is monomeric. Strong indications were obtained that both free receptors and their amine adducts are monomeric in dilute chloroform solution.

**Figure 151:**
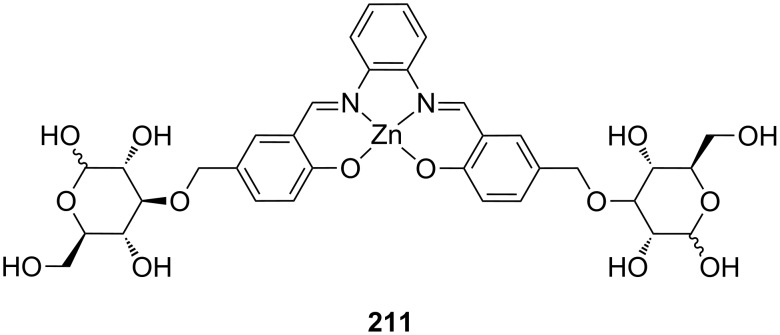
Bis(oxazoline)–copper(II) **211** for the recognition of amino acids in aqueous solution.

A “ditopic binder” recognizing ammonium ions with its side chains in water was described with a water soluble zinc-salophen complex **211** [673] (Figure 151). Its binding to carboxylate anions in water is very strong (*K*_ass_ > 10^6^ M^−1^). Amino acids are bound with associations constants ranging from *K*_ass_ = 3800 M^−1^ for glycine to *K*_ass_ < 5 M^−1^ for tryptophan (**81b**) were found from UV–vis spectrophotometric titrations. The general trend shows a gradual decrease in binding strength with increasing steric hindrance. The *K*_S_/*K*_R_ ratio of 9.6 observed for phenylalanine (**81a**, 2500 M^−1^ and 260 M^−1^, respectively) is among the highest values found for the chiral recognition of amino acids in water [674–675]. These findings led to the conclusion that amino acids are bound via zinc-carboxylate co-ordination and hydrogen bonding between the ammonium group and two oxygen atoms of one of the D-glucose moieties. This was supported by structures of the 1-glycine complex calculated at the semiempirical level (PM3).

**Figure 152:**
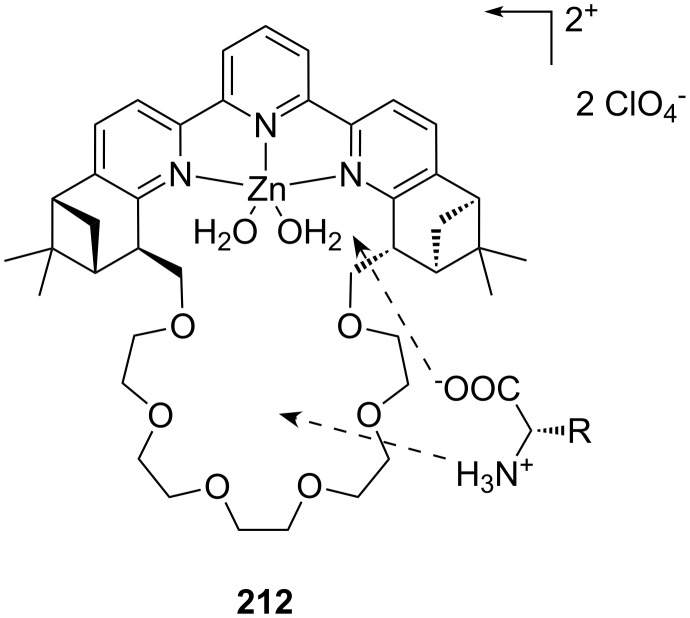
Zn(II)-complex of a *C*_2_ terpyridine crown ether.

A new fluorescence macrocyclic receptor **212** based on the Zn(II)-complex of a *C*_2_ terpyridine and a crown ether (Figure 152) has been developed for molecular recognition of zwitterionic amino acids in water/DMF solution with strong binding to *S*-aspartate (*K*_ass_ = 4.5 × 10^4^ M^−1^) and *R*-cysteine (*K*_ass_ = 2.5 × 10^4^ M^−1^) [676]. The Zn(II)-tpy subunit co-ordinates with the carboxylate group of the zwitterionic amino acids, and functions as a chromophore (λ_max_ = 348 nm) for the fluorescence sensing in aqueous solutions. The crown ether subunit binds the ammonium group of the zwitterionic amino acids. Without the crown ether subunit the binding to *S*-aspartate was about 90 times smaller, no significant change in fluorescence was observed for other amino acids. The binding properties of receptor **212** to different *S*-amino acids were studied by UV and fluorimetric titration methods. In all cases a 1:1 stoichiometry was observed and the equilibrium binding constant *K*_ass_ was estimated using the Benesi–Hildebrand equation. The binding affinity of receptor **212** to *S*-amino acids is highly dependent on the co-ordinating abilities of the side-chain chelating groups towards the Zn(II) metal (carboxylate > thiol >> amide >hydroxylammonium). *S*-Aspartate and *S*-cysteine showed the highest level of affinity to receptor **212**, which is about 4–14 times higher than *S*-asparagine and *S*-serine. *S*-Aspartate exhibited a much stronger binding (18 to 79 times greater) than the amino acids bearing an alkyl or aryl side-chain, and about 180 times higher than the cationic substrate (*S*-ornithine). The rigid *C*_2_ symmetric chiral groups in the Zn(II)-tpy subunit lead to enantioselectivity towards *R*-amino acids with *K*_R_/*K*_S_ up to 3.0 in the case of phenylglycine.

Indicator displacement assays are a popular method for converting a synthetic receptor into optical chemosensors. Amino acids are one substance class, which can be targeted by such colorimetric, fluorescent, and metal containing assays. Many examples along with their biological counterparts have been highlighted [677].

**Figure 153:**
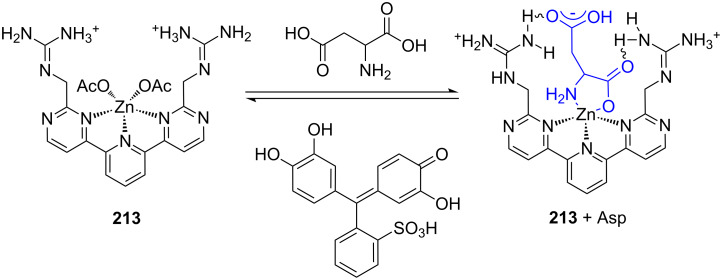
Displacement assay and receptor for aspartate over glutamate.

Anslyn et al. targeted the neurotransmitters aspartate and glutamate in a pyrocatechol violet displacement assay (Figure 153) in a water/methanol mixture (1:1; buffered with 10 mM HEPES at pH 7.4) [678]. The zinc complex was perfectly stable under these conditions. The highest affinity was found for aspartate (*K*_ass_ = 1.5 × 10^5^ M^−1^) with a seven fold stronger recognition over succinate, or glutamate, and by a factor of near 15 over the hydrophobic amino acids. The affinity of **213** is dominated by the interaction with Zn(II). In the case of aspartate the appended guanidinium groups also contributed to the binding. In addition, it was also observed that the use of metals in receptors can lead to larger color changes in indicator displacement assays. A shift in absorbance of the bound indicator that cannot be achieved with receptors which simply rely on hydrogen bonding and ion pairing for perturbing the ionization state was given as reason for this observation.

**Figure 154:**
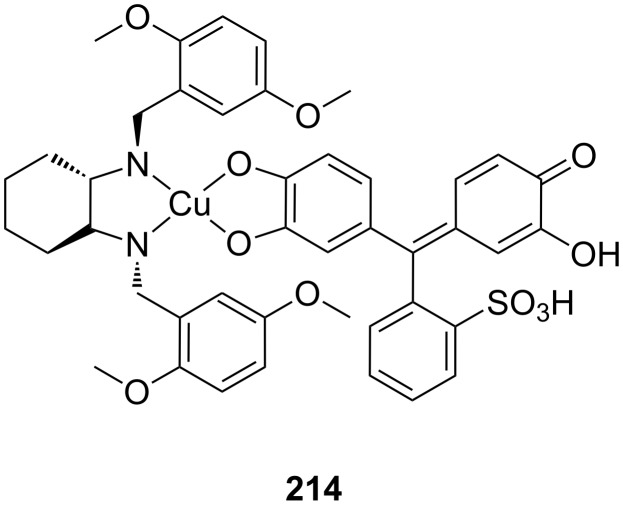
Chiral complex **214** for a colorimetric displacement assay for amino acids.

They also reported a comparable colorimetric technique for *ee* determination of non-derivatized *R*-amino acid samples in H_2_O/MeOH solutions based on a displacement assay with pyrocatechol violet (Figure 154). This instance a copper complex was used for the competitive metal co-ordination [679]. The ability of (*S*,*S*)-**214** to differentiate enantioselectively four of the hydrophobic *R*-amino acids was shown by UV–vis spectroscopy. Titration of *R*-amino acids in (*S*,*S*)-**214** resulted in a decrease of the Cu(II) absorbance. These experiments were carried out in the presence of a 10-fold excess of ligand (*S*,*S*)-**214** to discourage dissociation of (*S*,*S*)-**214**, and avoid the creation of 2:1 complexes. Valine and tryptophan (**81b**) gave the best values for their 1:1 complexes. *R*-Val and *S*-Val bound with association constants of 5.2 × 10^5^ M^−1^ and 2.0 × 10^5^ M^−1^, respectively, resulting in an enantioselectivity *K*_R_/*K*_S_ = 2.6. For *R*-Trp and *S*-Trp the values were 1.1 × 10^6^ M^−1^ and 5.0 × 10^5^ M^−1^, giving a discrimination of *K*_R_/*K*_S_ = 2.2 . Overall, the data showed a consistent preference for *R*-amino acids by about a factor of 2 to 2.6.

**Figure 155:**
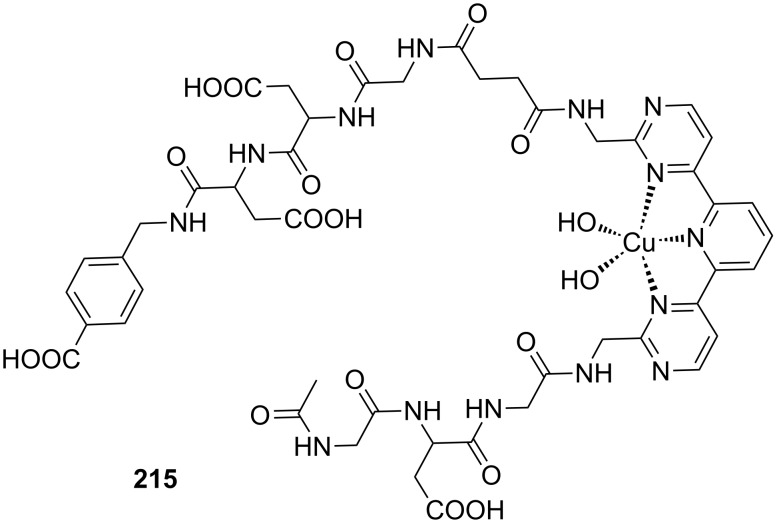
Metal complex receptor **215** with tripeptide side arms.

The insertion of strong co-ordination centres into peptides enables the construction of selective molecular receptors with complementary frameworks suitable for differentiation of amino acids and small peptides. A metal-centred receptor **215** consisting of a rigid backbone region and variable tripeptide arms [680] (Figure 155) for the recognition of tripeptides has been reported. The receptor is selective by co-operative interactions of the peptidic arms for *S*-xxx-*S*-Lys-*S*-Lys, with xxx = *S*-His, *R*-Cys, and *S*-Met with association constants near 10^6^ M^−1^. The binding studies in a water/methanol solution (1:1; buffered with 100 mM HEPES at pH 7.4) by UV–vis titration indicated from the association constants of the protected peptides, that amino acids were bound through their amino terminus. *N*-Terminal metal-chelating amino acids appended to basic amino acids bound with enhanced affinities via metal-chelating and ion pairing. *N*-Terminal His with two appended Lys showed the maximum binding with a value of 10^6^ M^−1^. The increase in affinity by a factor of near 10–30 over *R*-Cys-*S*-Lys-*S*-Lys and *S*-Met-*S*-Lys-*S*-Lys with *K*_ass_ = 3.0 × 10^5^ and 10^5^ M^−1^, respectively, was contributed to by the ion-pairing interactions possible with the guest peptide residues. In contrast, the His-, Cys-, and Met-Gly-Gly analogues affinities dropped approximately 100 fold.

**Figure 156:**
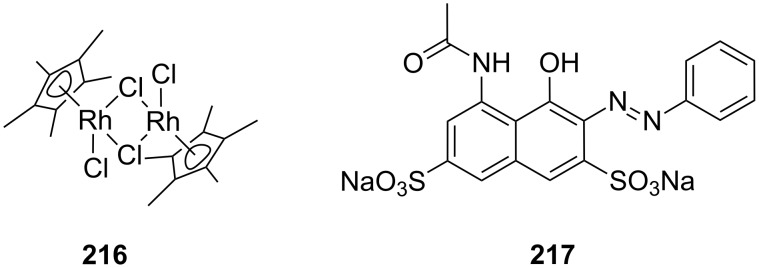
A sandwich complex **216** and its displaceable dye **217**.

Recognition of amino acids [681] and peptides [682] can be performed by a displacement assay with the rhodium sandwich complex **216** and an azo dye such as **217** (Figure 156). The aggregate distinguishes peptides with His and Met residues in position 1 or 2 at the *N*-terminus from other peptide sequences. The association constant of His-Ala, His-Gly-Gly, Leu-His-Leu or Gly-Met-Gly to **216** with values around 10^10^ M^−1^ exceed the binding strength of the dye **217** by three orders of magnitude. Peptides such as Val-Phe or Lys-Tyr compete so weakly with the dye that recognition of the former noted peptides in aqueous solution is possible even in the presence of a 100 fold excess of them. A colorimetric assay for the 20 natural amino acids in water was developed with this system [682].

**Figure 157:**
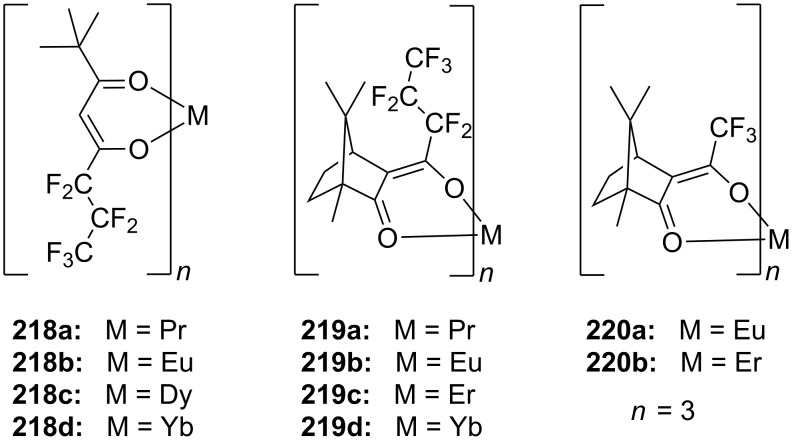
Lanthanide complexes **218**–**220** for amino acid recognition.

A recent example uses lanthanide complexes as receptors for the recognition of unprotected amino acids. Lipophilic lanthanide complexes of fluorinated diketonate ligands **218** to **220** (Figure 157) were shown by extraction experiments to bind unprotected phenylalanine (**81a**), leucine, and other amino acids under neutral conditions [683]. All tris(diketonates) formed 1:1 complexes with amino acids. The observations were verified by NMR and CD spectroscopic studies, which also suggested that the metal complexes bound the amino acid guests at two points. Their extraction, transport, and chiral recognition behaviors were significantly controlled by a combination of central lanthanide cation and co-ordinating ligand: The chiral ytterbium complex **219d** offered good enantioselectivity in the extraction of unprotected amino acids (Ph-Gly; 49% *ee*), and the related praseodymium complex **219a** provided efficient membrane transport (Phe; 62%). For receptors **218** the order of extraction from DCM to water was determined as Phe > Trp > Leu > Ph-Gly with a maximum value of 52%. Compounds **219b** and **219b** extracted Ph-Gly, Phe and Trp up to 62% under the same conditions. Complex **219b** especially exhibited excellent extraction ability for amino acids due to the effect of the electronegative fluorinated moieties [684–686] of the ligand that increase the Lewis acidity of the lanthanide tris(diketonate). This led to strong co-ordination of the carboxylate anion of the amino acid guest. In addition, fluorinated ligands enhanced the solubility of lanthanide tris(diketonates) and their ternary complexes with amino acids in the organic media [687].

Metal complexes of porphyrins, bisoxazolines, tripyridines, salens and many other ligands are valuable binding sites for amines and amino acids. By co-ordinative bonds they are able to form stable aggregates even in highly competitive media, such as water. Thus, they enable the recognition of targets such as amino acids and peptides in this challenging surrounding. Bidentate co-ordination of the guest allows enantiodiscrimination.

### 7. Other concepts: natural ionophores, (cyclo)peptidic hosts, reactive systems and more

A variety of less frequently applied concepts for ammonium ion binding have been reported in the literature, which cannot be allocated to one of the former sections: natural ionophores, their derivatives and related molecules, peptidic- and cyclopeptidic structures, and reactive groups. We discuss selected examples of these concepts in the following part.

#### 7.1. Natural ionophores

**Figure 158:**
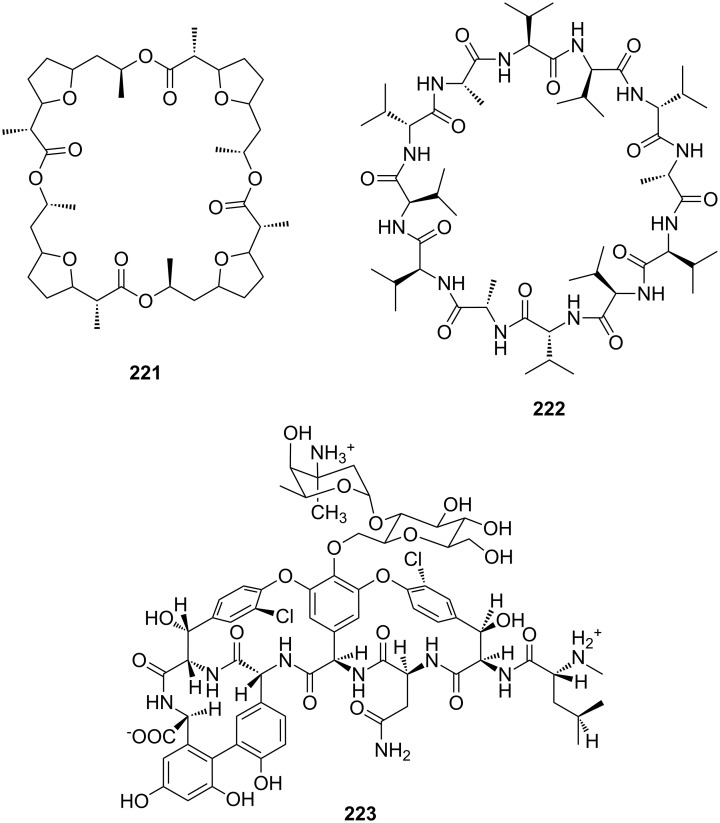
Nonactin (**221**), valinomycin (**222**) and vancomycin (**223**).

The best known naturally occurring macrocycles with ammonium ion affinity are the nonactins (**221**), valinomycin (**222**) [618] or the natural antibiotic vancomycin (**223**) [688] (Figure 158). Vancomycin (**223**) recognizes the Lys-*R*-Ala-*R*-Ala sequence and inhibits linking of these building blocks in the bacteria cell walls, thus causing cell death by osmotic overpressure [5]. For a considerable time it has been used as a reserve antibiotic, a so called “last line of defence”, because little resistance was observed [688] which is no longer the case.

Valinomycin (**222**) is a cyclodeca-depsipeptide consisting of *S*-valine, *R*-valine, *S*-lactate and *R*-hydroxyisovalerate with the repetitive structure (*S*-Lac-*S*-Val-*R*-Hiv-*R*-Val-)_3_, forming a ring of 36 atoms, with alternating amide and ester bonds. Similar to the interaction of crown ethers with cations, valinomycin guest binding is based on ion-dipole interactions between the oxygen atoms positioned along the ring and the guest [689]. The molecule is pre-organized through hydrogen bonding of its amide carbonyl groups to form a pocket with six ester carbonyl oxygens available for electrostatic stabilization of potassium ions through octahedral complexation [690]. Ammonium ions are bound in the same way. The selective transport of potassium ions by valinomycin through the cell membrane causes cells death by breakdown of the membrane potential [691–692]. The binding strength for potassium ions in aqueous media is 10^6^ M^−1^ [693–696].

Investigations of the ammonium ion complex of valinomycin in methanol by capillary electrophoresis gave an apparent stability constant of log *K*_NH4+_ of 1.52 ± 0.22 [618,697], which is in good agreement with the earlier determined value of log *K*_ass_ = 1.67 obtained from spectrophotometric measurements [698]. In comparison to the ammonium ion binding ability of 18-crown-6 (**4**) in the same solvent obtained by conductivity measurements (log *K*_ass_ = 4.1) [699], the value is two orders of magnitude lower.

The binding properties and association constants (*K*_ass_) of synthetic crown ethers with different cavity size and substituents and the natural ionophores valinomycin and nonactin versus deferriferrioxamine B, CH_3_(CH_2_)_4_NH_3_^+^, NH_4_^+^, K^+^, and Mg^2+^ in water saturated chloroform were reported (Table 15) [700].

**Table 15:** Binding values of natural ionophores compared to a 18-crown-6-derivative in chloroform.

Host	log *K*_ass_ (guest perchlorate salt)		
	Potassium	Ammonium	*n*-butylammonium

*cis*-dicyclohexano-18-crown-6^a^	8.23	7.69	6.16
valinomycin (**222**)	8.99	7.15	4.20
nonactin (**221**)	7.18	7.66	5.19

^a^= Reference [701].

These values were later confirmed by a mass spectrometric study [702]. Evaluation of the cation complexation by ^1^H or ^13^C NMR methods, in solution or solid-state, has been reported for thel ionophores: valinomycin [703–706], nonactin and tetranactin [707], and cereulide [708]. Potassium ions cause significant interference in ammonium ion detection because the potassium ion is similar in size to the ammonium ion (1.33 Å) [118].

In contrast to valinomycin, nonactin is selective for ammonium ions over potassium ions. It exceeds crown ethers in selectivity and shows excellent selectivity in NH_4_^+^ transport relative to K^+^ (NH_4_^+^/K^+^ ~ 14) [126]. In ion transfer reactions of the ammonium, potassium, and sodium ions with the ionophores dibenzo-18-crown-6, nonactin (**221**) and valinomycin (**222**) investigated at a water/1,2-dichloroethane interface, nonactin was found to be the most selective towards the ammonium ion, with a calculated association constant of 14.1 [709]. It is therefore widely employed in ion selective electrodes since it is superior to many artificial ionophores [log *K*_NH4+, K+_ = −1.0, log *K*_NH4+, Na+_ = −2.6] [126] and exhibits a detection limit for ammonium ions of 10^−6^ M [128]. Often it serves as a reference compound for the development of new ionophores for ISEs.

Nonactin (**221**) (Figure 158) is a naturally occurring ionophore, a highly symmetric meso compound with flexible conformation, when no ion is present [710]. The unbound conformation is relaxed and almost planar, possesses strong intramolecular non-bonding dipoles and lacks hydrogen bonding interactions [711]. It adopts a puckered conformation when bound to ammonium ions, pre-organized with the ion bound, leading to a good overlap of the oxygens which stabilize the charged ammonium hydrogens [712–713].

**Figure 159:**
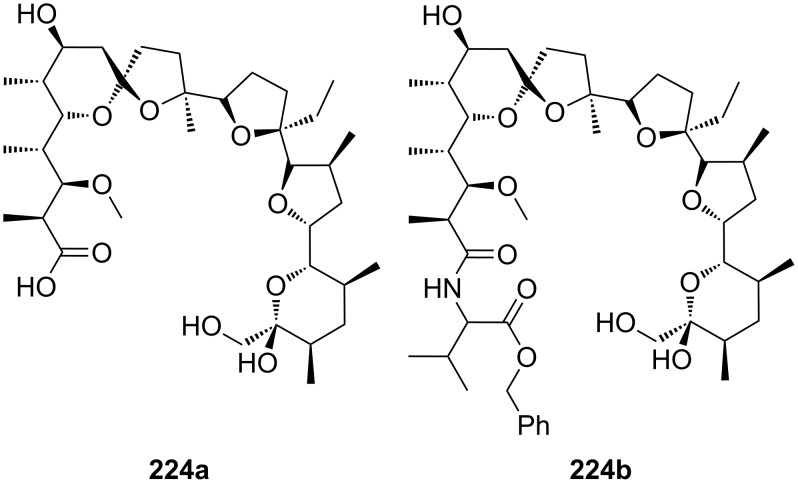
Monesin (**224a**) and a chiral analogue for enantiodiscrimination of ammonium guests (**224b**).

Monensin esters (Figure 159) are sodium ionophores, but synthetic analogs bind primary ammonium ions selectively and offer chiral recognition ability comparable to that of Cram’s binaphthyl crown ether. As demonstrated by experiments in an ion selective electrode in buffered aqueous solution and by NMR studies in chloroform, enantioselective complexation is found for chiral phenethylamine and naphthylamine salts, as well as for some amino acid esters. (*R*)-1-(l-Naphthyl)ethylammonium acetate is bound with three fold selectivity over the corresponding *S*-enantiomer by (*S*)-**224b** [714].

**Figure 160:**
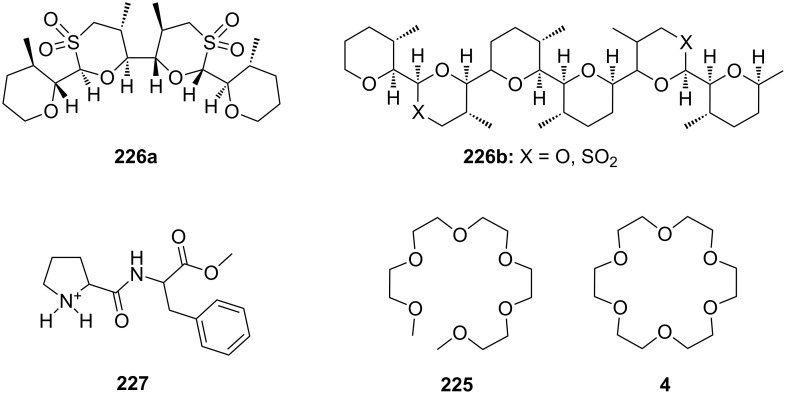
Chiral podands (**226**) compared to pentaglyme-dimethylether (**225**) and 18-crown-6 (**4**).

A variety of natural polycyclic antibiotics bear a structural resemblance to podants and they reveal often stunning selectivities and binding properties. Podands form complexes of lower stability than their corresponding macrocyclic counterparts. In the case of pentaglyme dimethylether (**225**) versus 18-crown-6 (**4**) (Figure 160), the macrocyclic ether binds the *tert*-butylammonium ion 10^4^ times more tightly [100]. The enormous difference in binding results from the macrocyclic effect. In structures such as the monesins (**224a**) and lasalocid A (**228**) this is overcome by the pre-organizing effect of the furan and pyran rings leading to a half-moon like array, as well as the possibility to build manifold contacts to the guest.

This effect can be nicely seen in the artificial systems presented by Still et al. Chiral podand analogs (**226b**) of 18-crown-6 (Figure 160), conformationally locked, reveal ionophoric properties closely related to the macrocycle. These host molecules have a cation-binding site with six oxygens with the same geometrical arrangement as found in the crystal structure of potassium 18-crown-6 [715].

The conformationally homogeneous podand receptor (**226a**) even binds proline-derived dipeptidic substrates (**227**) enantioselectively and diastereoselectively [716]. A closely related enantiomerically pure, *C*_2_ symmetric tetracyclic podand forms well-defined complexes with chiral ammonium salts. With derivatives of α-phenethylammonium hexafluorophosphate as guests, binding enantioselectivity up to 60% *ee* is achieved [136].

**Figure 161:**
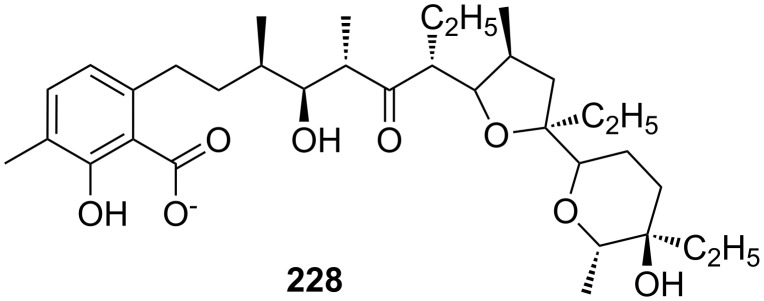
Lasalocid A (**228**).

Lasalocid A (**228**, Figure 161) binds by its OH groups and ether oxygens, and can compete with the macrocycles. It is a widely employed ionophore antibiotic that can effectively complex ammonium ions in a similar manner to crown ethers. As a drug it is used as its sodium salt. The mechanism of lasalocid activity is clearly attributed to its ionophoric properties where especially the influx of Na^+^ in the cell of Gram-positive and anaerobic bacteria causes swelling, vacuolization and finally cell death [717].

Lasalocid A can form strong complexes with biogenic amines such as dopamine (**2**), norepinephrine, 2-aminoheptane, as well as tyramine and transport them across biological membranes [718–724]. The crystal structure of a protonated amine with lasalocid shows all protons of NH_3_^+^ hydrogen bonded. The complex is stabilized, in addition, by some intramolecular hydrogen bonds [725]. In the gas, liquid and solid states lasalocid forms a very stable 1:1 complex with allylamine with its structure comparable in all states examined. Due to these interactions the outside of the complex is hydrophobic enabling ammonium transport across the biological membranes.

**Figure 162:**
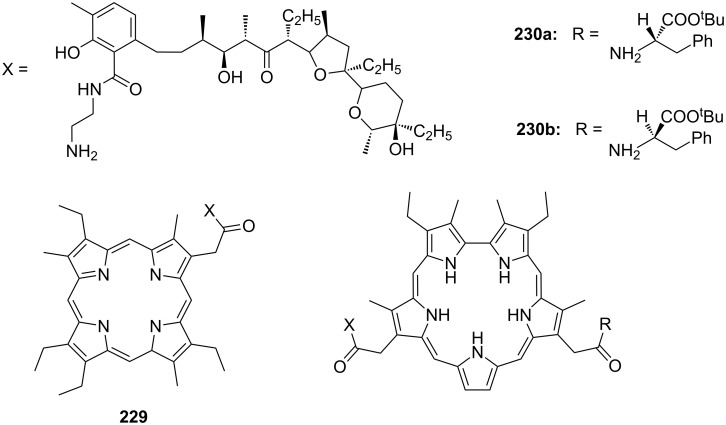
Lasalocid derivatives (**230**) of Sessler et al.

Sessler et al. reported sapphyrin ± lasalocid conjugates (**230**) which feature binding sites for both carboxylate anion complexation and ammonium group recognition (Figure 162) as efficient and selective carriers for aromatic amino acids [726]. In through-membrane model transport experiments, carrier **229** showed selectivity for phenylalanine (**81a**) over tryptophan (**81b**). Tyrosine is not transported to any significant extent. In general *S*-amino acids were transported with greater efficiency than the corresponding *R*-enantiomers by this particular carrier. The high level of amino acid carrier capability displayed by receptor **229** in dichloromethane solutions correlates well with the results of equilibrium binding studies carried out using visible-spectroscopic titrations.

By comparison two second generation sapphyrin ± lasalocid conjugates **230** were reported as carriers for the transport of Phe, Trp, and Tyr. A clear difference was observed between the free acid and the ester of **230**. The former did not affect amino acid transport, which was explained by receptor inactivation by self assembly. Depending on the chirality of the phenylalanine appendage (**230a** or **230b**) used, either *S*- or *R*-enantiomers of amino acid substrates were transported faster.

**Figure 163:**
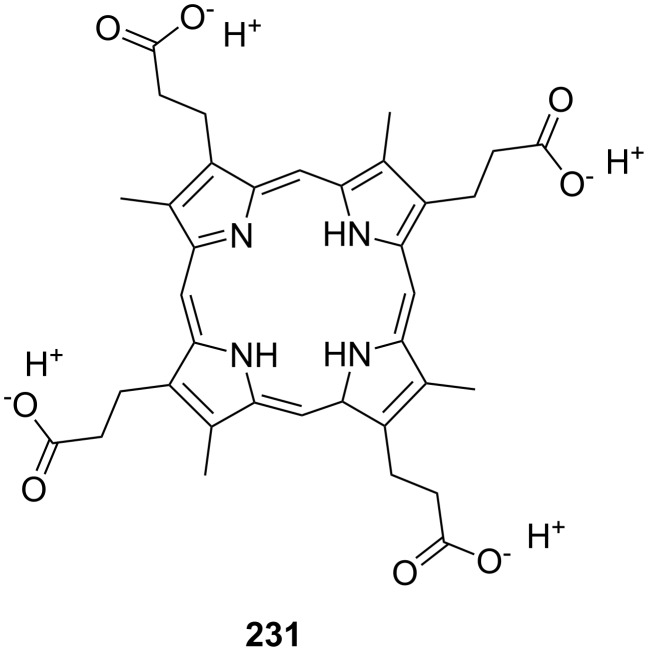
The Coporphyrin I tetraanion (**231**).

Coporphyrin I (CP, **231**, Figure 163) was employed as a host molecule [727]. As a tetraanion it binds electrostatically to the terminal ammonium groups of diammonium cations and interacts simultaneously with the hydrocarbon chain by its hydrophobic π-plane. Aliphatic diamines [H_2_N–(CH_2_)*_n_*–NH_2_, *n* = 2–8] were studied by spectrophotometry, fluorimetry and ^1^H NMR spectroscopy in the pH range 7–10 and ionic strengths 0.01–0.1 M in water. The dominant factor for binding was assigned to the ion-pair interaction. Diprotonated diammonium cations induced dimerization of CP by forming 1:1 complexes with CP, which undergo much stronger self-aggregation than free CP tetraanions. Increasing the number of methylene units connecting the ammonium groups, leads to an increase of the binding constants for the complex formation with monomeric CP (*K*_S_), but the dimerization constants of the resulting complexes were found to decrease. Even at *I* = 0.1 M the association is still fairly strong with log *K*_ass_ = 3. In the series of H_3_N^+^–(CH_2_)*_n_*–NH_3_^+^ cations, the log *K*_ass_ decreases with the increasing length of the guest by 0.1–0.3 units per methylene group.

#### 7.2. Peptidic- and cyclopeptidic ammonium hosts

Cyclic peptides are known to bind and transport metal cations in biological systems [728]. Their ease of synthesis and potential for flexible sequence modification make them good candidates for new ionophores [729].

Cyclic peptides platform structures with convergently oriented groups for ion recognition have been highlighted [730]. A review of peptide cyclization and cyclopeptides has only recently appeared [731]. Many examples for the synthesis of cyclic and bicyclic peptides can be found in the literature [732–740]. Kubik et al. published a comprehensive review about cyclopeptides as macrocyclic hosts [741]. Several cyclic peptide systems have been synthesized for ammonium complexation. We now present some representative, recent examples.

The RGD sequence is a key recognition element found in many proteins that interact with integrins on cell surfaces [742]. The combination of an integrin-binding RGD-cyclopeptide with a hexadecalysine DNA binding domain leads to peptidic minivectors for efficient gene transport [743]. The recognition of the ammonium residues by the DNA is crucial for this process.

**Figure 164:**
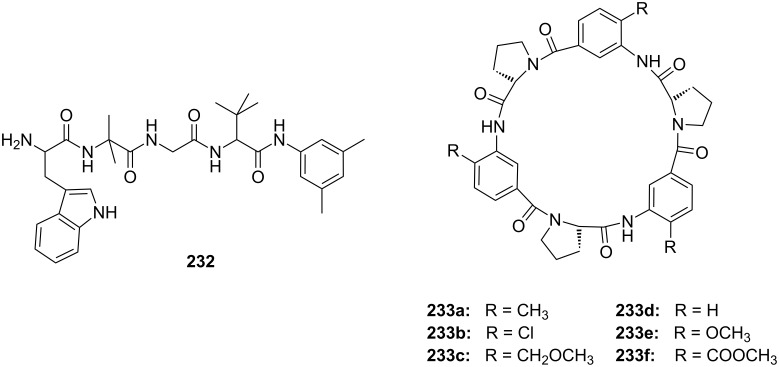
Linear and cyclic peptides for ammonium ion recognition.

The two tetrapeptide sequences Trp-Aib-Gly-Leu-NH-Ar (Aib: α-aminoisobutyric acid, 2-amino-2-methylpropanoic acid, Ar = phenyl or 3,5-dimethylphenyl) (Figure 164) bind ammonium ions by their aromatic moieties. The turn structure induced by the amino acid sequence leads to a sandwich complex of the guest between both π-systems as confirmed by 2D NMR ROESY experiments [744]. The peptide **232** bound several quaternary ammonium salts in CDCl**_3_** with the highest binding constants for benzyltrimethylammonium chloride and *N*-butylpyridinium chloride with association constants of 580 M^−1^ and 1000 M^−1^, respectively.

The chiral recognition of guest compounds by the tetrapeptides (X-Trp-Aib-Gly-Leu-NH-Ar) was also observed. The binding constants and the enantioselectivities of *N*-terminal free peptides were larger than those of peptides, which have a benzyloxycarbonyl group at the *N*-terminus [745].

Kubik et al. constructed a cyclic peptide composed of *S*-proline and three amino benzoic acids in an alternating sequence (Figure 164) that was able to bind ammonium ions with stability constants between 11000 and 42000 M^−1^ in chloroform. The series of cyclic hexapeptides contains different 4-substituted 3-aminobenzoic acid units (R = CH_3_, Cl, CH_2_OCH_3_, OCH_3_, COOCH_3_) [746]. The authors demonstrated that cyclic peptides **233** bind a variety of ammonium iodide salts with positive co-operativity in CDCl_3_. The cation complex stabilities depend on the substituents and can cover a wide range from *K*_ass_ = 140 M^−1^ for R = CH_3_ to *K*_ass_ = 10800 M^−1^ for R = COOCH_3_ (*K*_ass_ = 1260 M^−1^ for R = H) with *n*-butyltrimethylammonium picrate, for example. The peptide was found to adopt a conformation analogous to the cone conformation of a calixarene. Cations were bound by cation–*π* interactions, while the iodide counter ion co-ordinates via peptidic NH hydrogen bonds.

In a second study it was shown, that these cyclic peptides show enantiodiscrimination properties [747]. The best two examples, **233e** and **233f**, distinguish the two enantiomers of *N*,*N*,*N*-trimethyl-1-phenylethyl ammonium picrate in 0.1% DMSO–CDCl_3_ with *K_R_/K_S_* = 1.5. NMR titrations revealed binding constants (*K*_ass_) with the quaternary ammonium ion of 1550 M^−1^ for the *R*- and 1030 M^−1^ for the *S*-enantiomer binding to **233e** or 4550 M^−1^ for the *R*- and 3050 M^−1^ for the *S*-enantiomer binding to **233f** in 1:1 complexes.

The corresponding cyclic tetrapeptides composed of alternating *S*-proline and 3-aminobenzoic acid subunits possess a significantly smaller cation affinity than the hexapeptides [748]. Derivatives with suitable substituents on the aromatic subunits can be used as tweezer-type receptors.

As illustrated by the discussed examples and demonstrated by several further publications [749–751], cyclic peptides, depsipeptides and many natural ionophores selectively bind ammonium cations. Therefore, such structures can be utilized for the electrochemical analyses of such ions. The group of McGimpsey presented two approaches using cyclopeptides for ammonium ion detection in an ion selective electrode.

**Figure 165:**
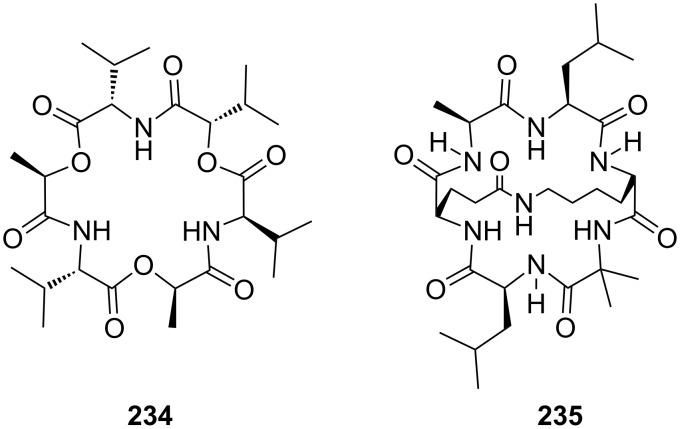
Cyclic and bicyclic depsipeptides for ammonium ion recognition.

A cyclic depsipeptide **234**, consisting of alternating amide and ester groups which is in effect half of the valinomycin structure (Figure 165), was employed as ammonium ionophore. Unlike valinomycin, this depsipeptide is too rigid to fold upon itself and therefore provides a cavity appropriately sized for ammonium ions, but not the octahedral binding geometry required by potassium ions (ionic radii: 1.43 and 1.33 Å, respectively) [752]. ISE sensors with this ionophore exhibited similar selectivity for ammonium over potassium and sodium ions compared to nonactin-based sensors (**221**) [126]. The ion selectivity follows the order of NH_4_^+^ > K^+^ > Na^+^ , Ca^2+^, Mg^2+^, Li^+^. The energy minimized structures showed the ammonium cation located within the pocket and able to hydrogen bond to at least five of the carbonyl groups. In contrast, the potassium cation adopts a position that is shifted to one side well above the plane of the disk-like structure of **234** reflecting an unfavorable binding site for potassium.

These cyclic peptides are still too flexible to bind substrates in a well-defined cavity [753], leading to lowered selectivity as sensor components. The addition of a second ring yielding bicyclic peptides was thought to increase cation binding selectivity by increasing rigidity.

The bicyclic peptide **235**, cyclo (*S*-Glu1 – *R*-Leu2 –Aib3 – *S*-Lys4 – *R*-Leu5 – *R*-Ala6)-cyclo-(1γ–4ε) (Figure 165) was introduced [754], to provide an ammonium ion complexation site in a tetrahedral geometry. The bicyclic ammonium ionophore **235** was designed for optimal size-fit/pre-organization, binding geometry and ISE membrane compatibility. A semi-rigid framework with a cavity appropriately sized for ammonium ions (ionic radius 1.43 Å) is necessary to impart high selectivity over interfering cations of other sizes [125].

The bicyclic molecule provides hydrogen bonding opportunities for the ammonium ion, primarily through the amide carbonyl groups, but also potentially through the amide nitrogen atoms. NMR measurements in CDCl_3_/CD_3_OD (1:1) indicate that four of the carbonyl groups are oriented towards the internal side of the cavity thus donating electron density upon complexation of ammonium ions. The compound shows higher selectivity for ammonium over potassium and sodium ions as determined by the downfield shifts in the carbonyl ^13^C NMR signals upon complexation.

#### 7.3. Miscellaneous concepts

Cyclodextrins were one of the first molecular receptors described to bind organic molecules and are widely used for inclusion of non-polar guests; in some cases they have been used for the recognition of quaternary ammonium ions [55,755–758]. Only recently an extensive thermodynamic study on the inclusion of quaternary ammonium surfactants was published [759].

**Figure 166:**
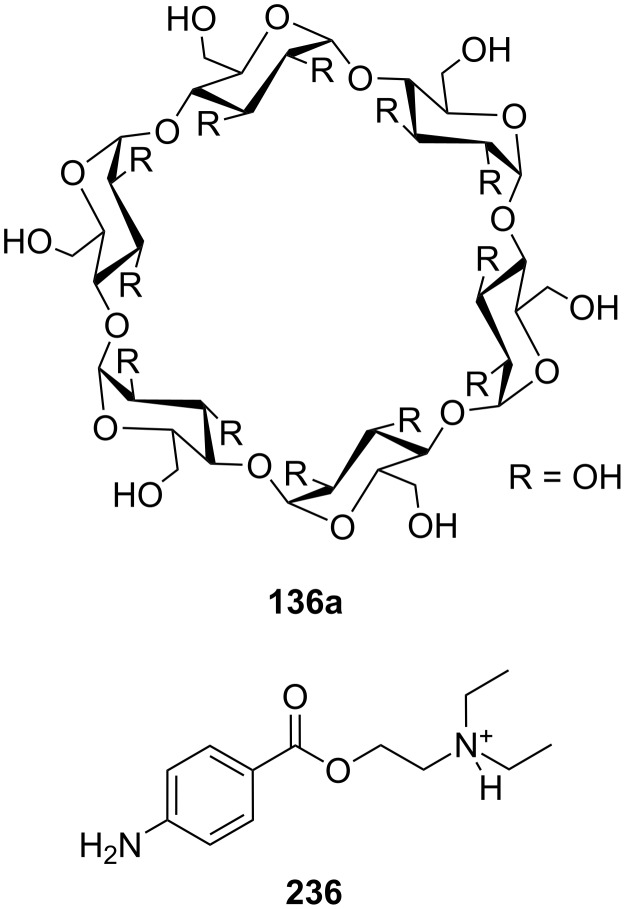
α-Cyclodextrin (**136a**) and novocaine (**236**).

The formation of inclusion complexes between α-cyclodextrin (**136a**) and the local anesthetic 2-(diethylamino)ethyl-*p*-aminobenzoate (novocaine, **236**) (Figure 166) was investigated in aqueous solution using steady-state fluorescence-, UV–vis spectroscopy and electrical conductivity measurements [760]. In addition, both the nitrosation reaction of the primary amine group in mild acid medium and the hydrolysis of the ester function in an alkaline medium have been studied. The inclusion complex formation between neutral or protonated novocaine and **136a** with a 1:1 stoichiometry was observed. However, the binding constants depend on the nature of guest and host: high affinities with an inclusion constant *K*_ass_ *=* 1500 mol^−1^ dm^3^ are observed under conditions where the novocaine and the cyclodextrin are neutral molecules.

The results obtained in this study showed that van der Waals interactions and hydrophobic interactions constitute the major driving forces for cyclodextrin complexation provided that the size and the conformation of the guest are complementary to the host cavity.

**Figure 167:**
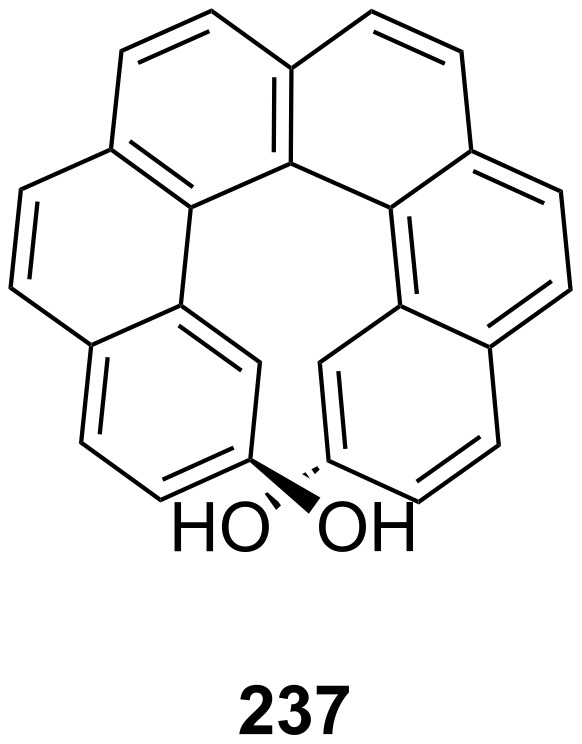
Helical diol receptor **237** by Reetz and Sostmann.

A completely different molecule has been shown to interact with various chiral amines and amino alcohols in organic solvents: the fluorescent helical diol **237** (Figure 167), reported by Reetz and Sostmann [761]. The authors suggest that the hydroxy moieties of **237** form hydrogen bonds with the amino group of the analyte, and no proton transfer is involved. Chiral discrimination was detected by differences in the fluorescence quenching observed upon binding to an amine. This chemosensor binds amines with modest stability constants.

**Figure 168:**
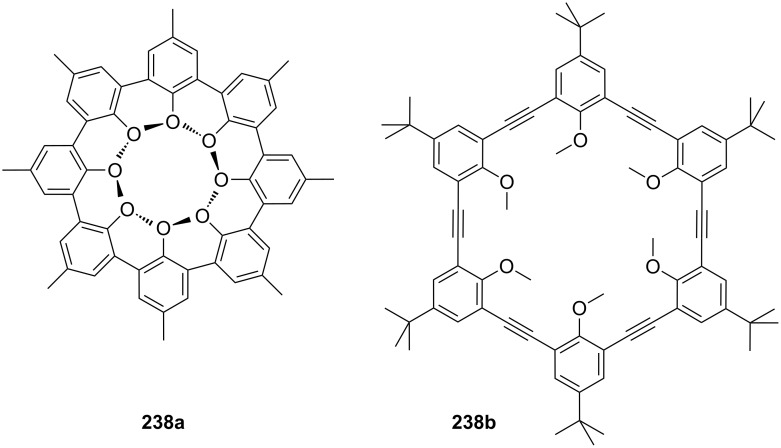
Ammonium binding spherand by Cram et al. (**238a**) and the cyclic[6]metaphenylacetylene **238b** in comparison.

Oda et al. further developed Cram’s spherands **238a** [17,762–763] to produce a better ammonium binder. They described a cyclophane (cyclic[6]metaphenylacetylene) [764] with six methoxy groups inside the cavity with acetylene units as spacers (**238b**) in a nearly planar carbon framework (Figure 168) as observed in the molecule’s crystal structure. The six methoxy groups point up and down, alternately. The cavity size is appreciably larger than the size of a caesium ion (3.4 Å). No measurable complexation with alkali metal ions in solvent extraction experiments (chloroform/aq picrate salts) was found. Compound **238b** exhibits good ionophoric selectivity for the ammonium ion in spite of its smaller size (2.86 Å) compared with a caesium ion. A plot by Shono’s method shows a straight line with a slope of approximately unity suggesting the formation of a 1:1 complex between **238b** and the ammonium ion in solution. The association constant obtained for the ammonium ion (log *K*_ass_ = 7.84) is smaller than that 18-crown-6 (log *K*_ass_ = 9.38), but larger than Cram’s cavitand **238a** (log *K*_ass_ = 6.59).

Raymond et al. reported a tetrahedral supramolecular, chiral assembly of four gallium atoms bridged by *N,N*′-bis(2,3-dihydroxybenzoyl)-1,5-diaminonaphthalene units for binding cationic guests. This cage can recognize and include monoprotonated amines in aqueous solution [765]. This allows monitoring inversion at the nitrogen atom and H-bond formation in a variety of diamines [766].

**Figure 169:**
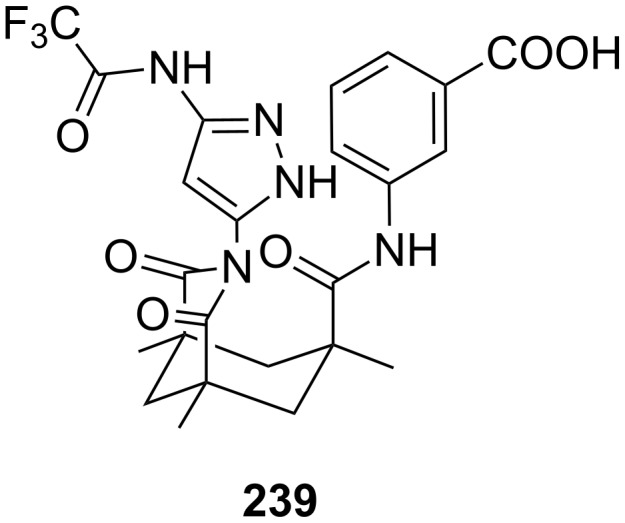
Receptor for peptide backbone and ammonium binding (**239**).

Based on the Kemp’s triacid, compound **239** (Figure 169) was developed for combined backbone and functional group recognition of peptides [767]. One molecule binds the ammonium ion side chain, as demonstrated with Ac-Orn-Ala-OMe (*K*_ass_ = 2400 M^−1^). A control experiment with *n*-propylammonium acetate gave a value of 490 M^−1^ for the salt bridge alone. Ornithine is bound with a 9:1 selectivity compared to all other amino acids employed in the dipeptide studied. All binding values were obtained by NMR titrations in chloroform; Job’s plot analysis confirmed a 1:1 stoichiometry.

**Figure 170:**
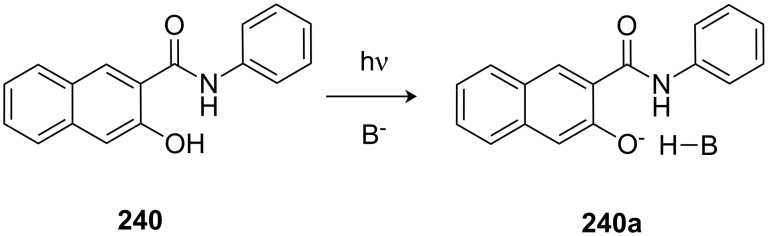
Anion sensor principle with 3-hydroxy-2-naphthanilide of Jiang et al.

The demethylated naphthol reported by Lambert et al. binds by co-ordination via H-bonds and also via the amide nitrogen [768]. The authors chose a variation of the molecule [769–770] of Jiang et al. (Figure 170), which was able to bind a variety of anions (**240**). This group used the commercially available dye naphthol AS-BI, which was developed for the cytochemical detection of alkaline phosphatase [771]. Aliphatic amines are detected through binding with 7-bromo-3-hydroxy-*N*-(2-hydroxyphenyl)naphthalene 2-carboxamide and the fluorescence of the resulting complex.

**Figure 171:**
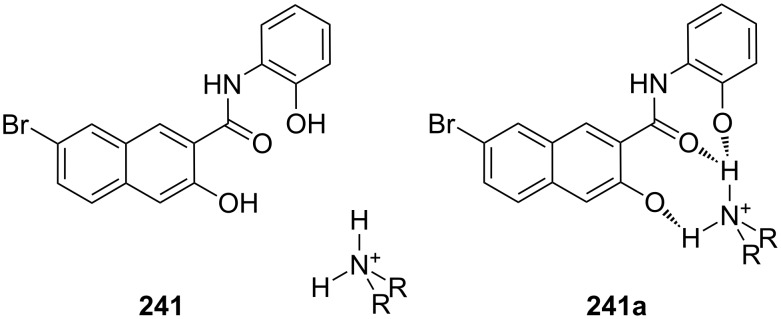
7-bromo-3-hydroxy-*N*-(2-hydroxyphenyl)naphthalene 2-carboxamide (**241**) and its amine binding.

The demethylated derivative 7-bromo-3-hydroxy-*N*-(2-hydroxyphenyl)naphthalene 2-carboxamide (**241,** Figure 171, colorless in the ground state, λ_max_ = 335 nm), emits upon excited-state complexation at 525 nm. Proton transfer is enabled by the enhanced acidity of the OH group on the naphthalene on photoexcitation. Recognition of the amine by the chemosensor **241** therefore occurs via proton transfer of the naphthalenic OH proton to the amine and is facilitated by the presence of the phenol group. Amine basicity is the primary parameter for detection and consequently poorly basic aromatic and conjugated amines such as pyridine and aniline are not detected, but almost all aliphatic amines are. Hydrogen bonding within the complex allows further differentiation of aliphatic amines in the following order of binding strength: diamines > secondary amines > primary amines > tertiary amines > aromatic amines, heterocycles. Table 16 gives an overview of the binding strengths.

**Table 16:** Binding constants for **241** in acetonitrile.

Amine	*K*_eq_ (**241**) [M^−1^]

1-propylamine	80000
1-butylamine	92000
benzylamine	7000
histamine	35000
diethylamine, diisopropylamine	150000
4-(dimethylamino)-pyridine	6900
triethylamine	28000

Diamine	*K*_eq_ (**241**) [M^−1^]

1,2-diaminoethane, 1,4-diaminobutane	160000
1,3-diaminopropane, piperidine	180000
1,5-diaminopentane, 1,7-diaminoheptane	290000
1,8-diaminooctane	310000

Although non-covalent interactions are generally weak compared to covalent bonds, biomolecules achieve strong intermolecular binding forces by using several non-covalent interactions simultaneously. In a similar fashion, naturally occurring gallate-type catechins [772] stabilize complexes with quaternary ammonium ions by using dual non-covalent interactions [773].

**Figure 172:**
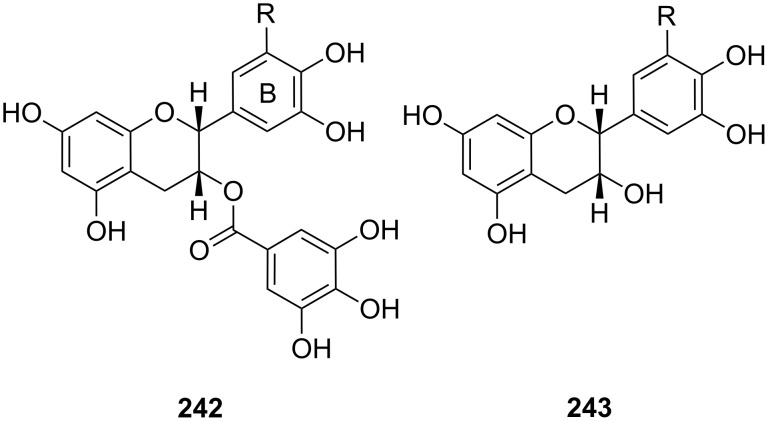
Naturally occurring catechins with affinity to quaternary ammonium ions.

Binding studies between the major catechins of green tea (Figure 172) and tetramethylammonium chloride (TMAC) [298] or benzyltrimethylammonium chloride (BMAC) were carried out by means of standard ^1^H NMR titration experiments in acetonitrile-d_3_/chloroform-d (1:1). The gallate-type catechins (for example **242**) had much higher binding ability (1300–2300 M^−1^) than the non-gallate-type catechins (200–400 M^−1^, for example **243**). This was attributed to the “biting effect” by the galloyl group and the B-ring. Compound **242** has the best binding ability of *K*_ass_ = 2300 M^−1^ towards BMAC.

**Figure 173:**
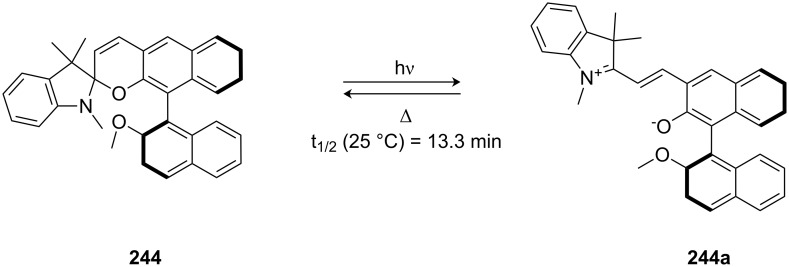
Spiropyran (**244**) and merocyanine form (**244a**) of the amino acid receptors of Fuji et al.

Fuji et al. published a system for optical distinction of enantiomers of amino acids [774]. The authors used the thermo- and photochromic, colorless spiropyran **244**. On treatment with UV light the colored merocyanine is formed (Figure 173): The zwitterionic species **244a** binds to amino acids by ionic and hydrogen-bond interactions. This complex formation in turn stabilizes the colored merocyanine state and so the bleaching observed under dark conditions is slowed down.

Due to the binaphthyl system diastereomeric complexes arise with chiral amino acids, which are distinguished by their decoloration rates (Table 17). The best stabilization of **244a** was achieved with ammonium acetate (t^1/2^ = 122 min).

**Table 17:** Dependency of the decoloring rate of **244a** in the presence of different *R*- and *S*-amino acids and ammonium acetate.

Guest	t_1/2_ (*R*, *S*) [min]

none	13.3
alanine	24.1, 23.4
valine	32.5, 28.1
tryptophan	20.2, 17.0
phenylalanine	30.4, 26.8
ammonium acetate	122

#### 7.4. Recognition by covalent bond formation

The ammonium ion is always in equilibrium with it’s corresponding amine. Thus, the possibility of nucleophilic attack can be used for recognition, simply binding the guest as imine or aminal. Such concepts are now presented in the last part of this review.

**Figure 174:**
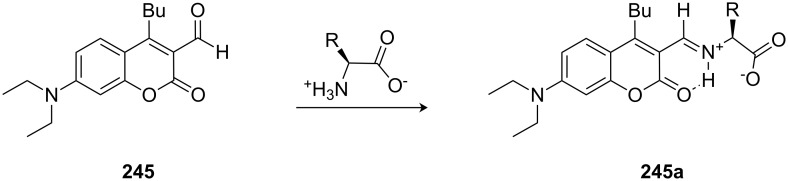
Coumarin aldehyde (**245**) and its iminium species with amino acid bound (**245a**) by Glass et al.

A covalent approach for the detection of ammonium ions was applied by Glass et al. Their coumarin derivative **245** forms iminium salts with ammonium ions (**245a**) [775] (Figure 174). The iminium formation can be monitored by UV spectroscopy using the resulting redshift of the long wavelength absorption band of approximately 440 nm to approximately 480 nm, as well as by a substantial (up to 45-fold) increase in the fluorescence intensity. As the main reason for the spectroscopic changes, the authors considered, the electronic effects caused by the formation of a hydrogen bond between the iminium hydrogen and the lactone carbonyl oxygen. The measurements were conducted under physiological conditions. Similar receptors based on hydrogen bond interaction show usually no affinity under these conditions. So, the equilibrium constants, e.g. for lysine (**81c**) *K*_eq_ = 6.5 M^−1^ for the retention of amino acids are certainly noteworthy.

**Figure 175:**
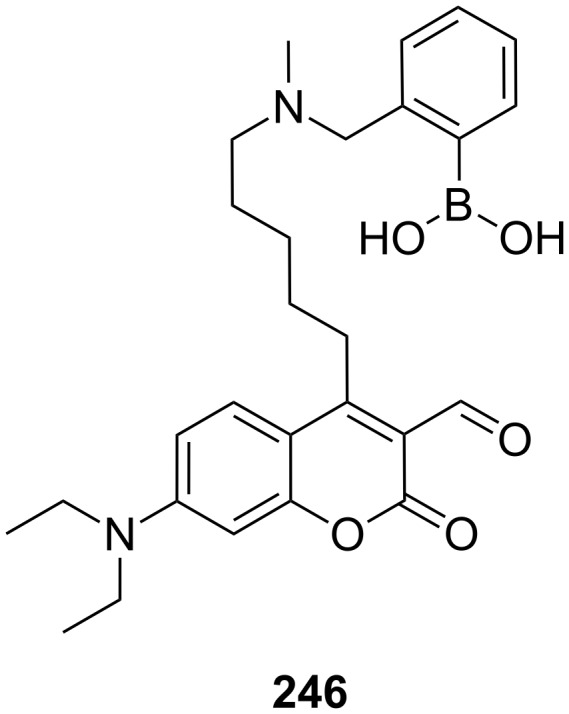
Coumarin aldehyde appended with boronic acid.

Later the group reported a dopamine (**2**) receptor based on the same principle: A boronic acid-containing coumarin aldehyde was designed (**246**) [776] (Figure 175). The sensor binds to catecholamines such as dopamine (**2**) and norepinephrine by forming an iminium ion with the amine as well as a boronate ester with the catechol. It acts as an effective colorimetric sensor for dopamine (**2**, *K*_ass_ = 3400 M^−1^, Δλ_max_ = 30 nm) and norepinephrine (*K*_ass_ = 6500 M^−1^, Δλ_max_ = 24 nm) with excellent selectively over epinephrine (*K*_ass_ = 5000 M^−1^, Δλ_max_ = 0 nm), amino acids, and glucose (*K*_ass_ = 5–7 M^−1^). The sensor responds differentially to catechol amines over simple amines, giving a fluorescence decrease in response to catechol-containing compounds (40–60% decrease) and a fluorescence increase with other amines (up to 50 fold for tyramine). The fluorescence quenching effect was found to be directly related to the catechol group. The electron-rich catechol is likely acting as a photoinduced electron transfer (PET) quencher of the coumarin under these conditions.

Other valuable binders for dopamine (**2**) have of course been described: Cyclophanes have been quite useful for selective dopamine recognition [777], including a recent example that displays shape-selective recognition with only non-covalent interactions [778]. For more examples the reader is referred to section six of this review.

**Figure 176:**
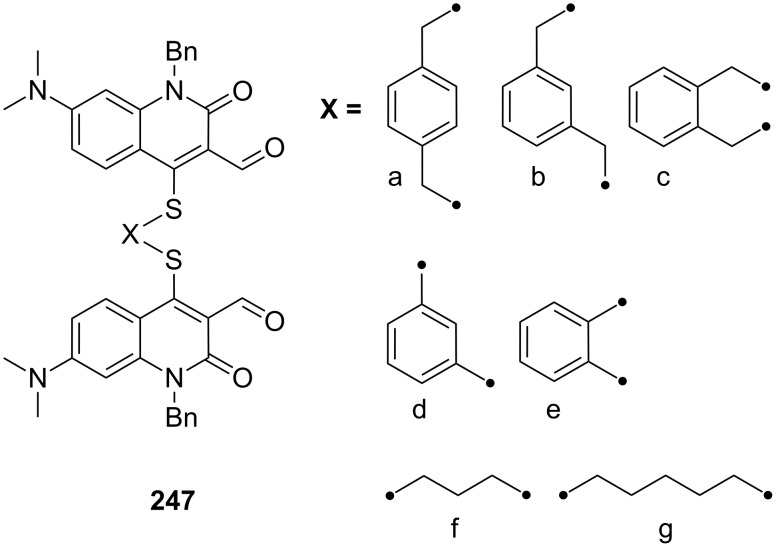
Quinolone aldehyde dimers by Glass et al.

A series of ditopic receptors (**247**) for diamines using dimers of a quinolone aldehyde chromophore (Figure 176) was explored by a combination of NMR, absorption and fluorescence spectroscopy [779]. It was shown that the dimeric sensors bound the diamine guests by formation of a bis-iminium ion, which produced large changes in the fluorescence of the quinolone core. Spectroscopic analysis was carried out in a 1:1 methanol–buffer solution system. The absorption spectra showed trends similar to those observed with the coumarin analogs in which a large red shift in absorption maximum was observed upon addition of diamines to the sensors. Diaminopropane was the best guest for all systems, with the highest binding to **247g** with a binding constant of 6700 M^−1^ which was 3–4 fold stronger compared to diamino-butane/pentane and 2.5 fold compared to ornithine/lysine (**81c**) with a maximum fluorescence increase at saturation (*I*_sat_/*I*_0_) of 6.6-fold. It bound lysine (**81c**) with 2800 M^−1^ and a fluorescence increase of 30 fold. The second best binder was **247d**. A shift in absorbance up to 28 nm was observed, consistent with a shift from aldehyde to iminium ion forms. The red shift in absorption has been attributed to the hydrogen bond between the formed iminium ion and the carbonyl group of the chromophore. By exciting the chromophore at 495 nm, a large increase in fluorescence was observed upon titration with the diamine: Up to 160 fold better binding for diamines compared to butylamine. The mode of binding and the 1:1 stoichiometry were confirmed by NMR experiments in chloroform.

**Figure 177:**
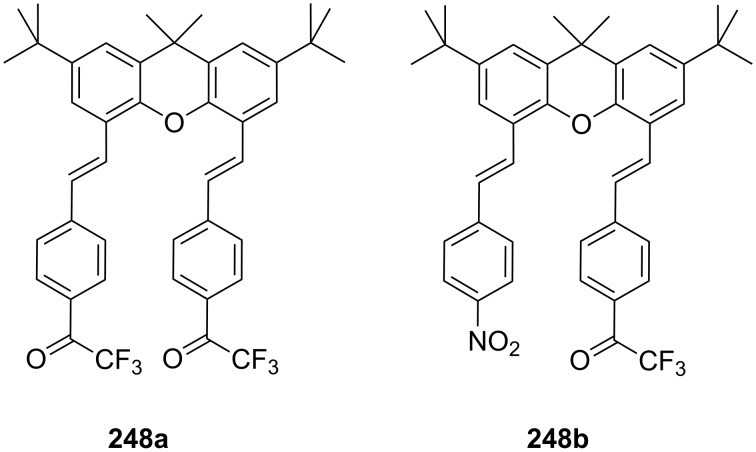
Chromogenic ammonium ion receptors with trifluoroacetophenone recognition motifs.

Reversible covalent binding of an amino, e.g. forming a hemiaminal, has been realized in two chemosensor dyes with either one or two trifluoroacetophenone recognition moieties (Figure 177). As amines 1-propylamine, diethylamine, triethylamine, and aliphatic diamines of different chain length were used [780]. Their conversion into a hemiaminal or a zwitterion leads to a change in the electron delocalization within the dye molecule and subsequently to a shift in absorbance to shorter wavelengths. Comparing the interaction of **248a** and **248b** with amines in homogenous solution it was found, that for their reaction with diamines the *K*_eq_ values are significantly increased. The highest value was observed for 1,2-diaminoethane and the lowest for 1,4-diaminobutane. Table 18 compares the results.

**Table 18:** Binding constants of amines to compounds **248a** and **248b** in ethyl acetate.

Amine	*K*_eq_ (**249a**) [M^−1^]	*K*_eq_ (**249b**) [M^−1^]

1-propylamine	195	210
1,2-diaminoethane	30000	5000
1,3-diaminopropane	26000	3500
1,4-diaminobutane	13000	700

**Figure 178:**
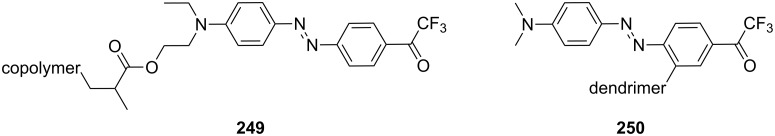
Chromogenic ammonium ion receptor with trifluoroacetophenone recognition motif bound on different matrices.

The response and sensitivity towards monoamines was comparable, because only one functional group in **248a** can react with amines. The dyes embedded in thin layers of plasticized PVC (Figure 178) showed clear changes in absorbance on exposure to aliphatic amines.

Similarly, the chromogenic functional dye **249** (Figure 178) shows a significant color change in the presence of amines in organic solvents, with high sensitivity [781]. The cross-linked polymer sensor membranes allow a fast and reversible chemical reaction with solutions of primary aliphatic amines in most organic solvents. The equilibrium constants varied, depending on the solvent and analyte molecule, the sensor layers typically exhibited equilibrium constants of 100 M^−1^ for *n*-butylamine in chloroform, 1300 M^−1^ for 1,4-diaminobutane and 20,000 M^−1^ for tris(2-aminoethyl)amine in toluene. A change in selectivity due to the size or polarity of the analyte could not be observed. The reaction rate of the membranes with secondary and tertiary amines as well as with alcohols is slower than the rate with primary aliphatic amines, which gave the opportunity to distinguish ammonium guests by structure.

Zimmerman et al. have prepared receptors for diamines by incorporating trifluoromethyl ketones into a dendrimer (**250**) with success [782–784]. Such receptors showed, for example, selectivity for α,ω-diamines (H_3_N^+^–(CH_2_)*_n_*–NH_3_^+^) versus aromatic and cycloaliphatic diamine, amines, amino alcohols and diols. Complexation studies in THF by visible spectroscopy and NMR afforded an apparent association constant (*K*_ass_) of 2.7 × 10^4^ M^−1^ for *n* = 3 that was ca. 200-fold higher than that for *n*-butylamine (140 M^−1^). The association constant for *n* = 4 was even 10–20% higher. Longer and shorter diamines bound less strongly [785].

## Conclusion

We have presented various approaches for the detection and binding of ammonium ions and amino acids ranging from metal-complexing agents or reactive molecules via different inclusion compounds to weakly co-ordinating systems, such as crown ethers. A large number of molecular receptors of varying sizes, shapes and functionalities have been discussed in their interaction with the guests.

The synthetic hosts require complementarity to the ammonium guests in size, shape, and molecular interactions [786]. Typical interactions observed in the complexes of primary and secondary ammonium cations are ionic and dipolar interactions, dispersive forces such as van der Waals or hydrogen bonds. Cation–π- and ionic-interactions, often assisted by the hydrophobic effect and dispersive forces determine the binding of quaternary ammonium ions.

Binding an organic ammonium ion in solution three aspects have to be considered:

An organic ammonium ion never exists as a sole cation, an anion is always associated with it. Depending on the polarity and hydrogen donor/acceptor abilities of the solvent, the association strength is different [787]. The strength of the electrostatic interaction in solution, despite the solvation [788–789] of host and guest, influences the binding to an artificial receptor. Strongly co-ordinating counterions such as chloride generally lead to weaker binding constants upon recognition of the associated cation as compared to when large, soft and weakly co-ordinating counterions such as iodide (tetrafluoroborate, hexafluorophosphate or perchlorate) are employed [790–792].

The binding of primary, secondary and tertiary ammonium ions to the most receptor structures relies on H-bonding to a large extent. The complex stability depends on the number of H-bonds possible between host and guest [793], but also on the acidity of the ammonium ion. The more acidic an ammonium ion is, the stronger are the H-bonds with a particular donor site. For instance, primary, secondary and tertiary ammonium ions have p*K*_b_-values between three and four and therefore stabilize a complex to a larger extent compared to an anilinium ion with a p*K*_b_-value of nine to ten.

The third fact of importance is the steric bulk present in the guest (and the host). The better an ammonium ion can be placed in the recognition motif and the less interference is present in the complex, the stronger the association (assuming no additional co-ordination of the substituents can take place).

The many different examples reported in literature show that crown ethers are one of the most versatile classes of synthetic receptors for the recognition of ammonium ions. Crown ethers recognize ammonium-ions typically by hydrogen-bond interactions. Therefore only ammonium ions of primary and secondary amines are typical guests and quaternary ammonium ions are not bound. The crown-ether ammonium ion recognition motif has been extended to multitopic receptors allowing an analytical discrimination of diamines of different length, combined with anion recognition for the binding of amino acids. Many examples of transport and effective enantioselective recognition of amino acids, as esters or in zwitterionic form have been described. Crown ether amino acid building blocks for synthetic receptors were developed by Voyer [187] (Figure 29) and König [192–193] (Figure 31 and Figure 32). Such systems allow the easy assembly of larger structures such as membrane channel mimics, which are of fundamental interest in medicine and biochemistry [794–795].

Substituted calixarenes can bind primary and secondary ammonium ions by ion–ion-, ion–dipole- and H-bond-interactions, and quaternary ammonium guests by ion–ion-, cation–π- and hydrophobic interactions. The molecular geometry of calixarenes is adjustable via their conformation, allowing a fine tuning of their selectivity for shape and size of the guest. This is not possible to the same extent with crown ethers. In addition, calixarenes often achieve binding selectivities exceeding those achieved with crown ethers due to guest inclusion being controlled by steric factors and various interactive forces of host and guest. Therefore, they can show remarkable selectivities in the discrimination of ammonium ion isomers. Especially noteworthy is their ability to complex strongly with quaternary ammonium ions.

Molecular tweezers and clips (Figure 109) serve as selective receptors for electron-deficient aromatic and aliphatic substrates. Cavity or clefts affect the thermodynamic stability and the binding kinetics; addition of side arms may enhance lipophilicity (long alkyl chains) or encourage interaction with some external entity, which makes these systems especially interesting for ammonium binding. Assisted by the hydrophobic effect of the cavity, van der Waals interactions and substantial electrostatic contributions for locking of the guest are responsible for the observed high efficiency and specificity found in clefts and cavitands. Water-soluble clips form stable complexes with *N*-alkylpyridinium, phenethylammonium ions, catechols and basic amino acids, which are often more stable in aqueous solution than in methanol due to a positive contribution of the hydrophobic effect to the receptor-substrate binding processes. *C*_3_*_v_* symmetric tripods, tweezer ligands and pre-organized molecular clefts reach ammonium ion binding selectivities that compete with naturally occurring recognition systems such as nonactin or valinomycin [618].

Cucurbiturils often reveal remarkably high affinity for alkanediammonium ions, size, shape, and functional group selectivity as a consequence of ion–dipole and hydrophobic interactions and have the highest binding constants of all presented receptor families in aqueous media (up to 10^10^ to 10^12^ M^−1^). Generally, ammonium guests are co-ordinated by the carbonyl groups of the moieties by electrostatic ion–dipole attraction assisted by hydrogen bonding. The non-polar part of the guest is included in the cavity. The binding is governed by hydrophobic effects and van der Waals contacts. The entropic gain upon binding additionally supports the high association constants found with cucurbiturils. Together with cyclodextrins, a wide range of host cavities for ammonium ions with different shape, solubility, and chemical functionality is available.

Lewis-acidic metal centres in combination with carboxylate, trimethylammonium or H-bond donors bind guests with a high degree of selectivity and affinity. Amines and amino acids are preferred guests. Ionic interactions in combination with hydrogen bonds and the hydrophobic effect are the main contributions for their complex stabilization. The strong co-ordination of the metal centre allows guest binding even in competitive media like water.

Porphyrins in particular provide a useful framework for artificial receptors. The conjugated system facilitates the detection of interactions by UV–vis, fluorescence or circular dichroism measurements. It also provides a planar structure for the design of well-defined binding pockets with recognition groups attached in several distinct positions. The types of interactions utilized in these receptors include hydrophobic interaction, hydrogen bonding and, in most cases, co-ordinative bonds, taking advantage of the Lewis acidity of a metal, typically zinc [796]. Dimer structures based on metal-porphyrins allow for the enantiodiscrimination of diamines, amino acids, peptides and amino alcohols.

The rules how synthetic receptors interact with ammonium ion guests become clearer, which paves the way for a rational design of biomimetic devices, non-covalent synthesis and responsive host–guest systems. The study of synthetic ammonium ion receptors has certainly contributed to a better understanding of intermolecular interactions in various fields including drug design, DNA processing, enzyme interactions or approaches for the inhibition of protein–protein interactions [797–798]. Applications of ammonium ion recognition may be envisaged in many areas: Drug design, photo switching, separation, or motion and transport [799–800], self assembly in solution, and in the solid state.
